# Author Correction: The isocyanide S_N_2 reaction

**DOI:** 10.1038/s41467-023-42066-z

**Published:** 2023-10-06

**Authors:** Pravin Patil, Qiang Zheng, Katarzyna Kurpiewska, Alexander Dömling

**Affiliations:** 1 Institute of Molecular and Translational Medicine, Faculty of Medicine and Dentistry and Czech Advanced Technology and Research Institute, Palackӯ University in Olomouc, Olomouc, Czech Republic; 2 Department of Drug Design, University of Groningen, Groningen, The Netherlands; 3 Department of Crystal Chemistry and Crystal Physics Faculty of Chemistry, Jagiellonian University, 30-387 Kraków, Poland

**Keywords:** Synthetic chemistry methodology, Sustainability, Synthetic chemistry methodology

Correction to: *Nature Communications* 10.1038/s41467-023-41253-2, published online 19 September 2023

In this article the author name Katarzyna Kurpiewska was incorrectly written as Katarzyna Kurpiewsk. The original article has been corrected.

