# Supplementary material for: The isocyanide SN2 reaction
Source: Nat Commun. 2023 Sep 19;14:5807. doi: 10.1038/s41467-023-41253-2 (PMC10509164; doi:10.1038/s41467-023-41253-2)
Supplement: Supplementary file 1 — Supplementary Information [file 41467_2023_41253_MOESM1_ESM.pdf]

Supplementary Information

The isocyanide S_N2 reaction

Pravin Patil[1,2#], Qiang Zheng[2#], Katarzyna Kurpiewsk[3], Alexander Dömling[1,2*]

[1] Institute of Molecular and Translational Medicine, Faculty of Medicine and Dentistry and Czech Advanced Technology and Research Institute, Palacky University in Olomouc, Olomouc, Czech Republic

[2] Department of Drug Design, University of Groningen, Groningen, The Netherlands

[3] Department of Crystal Chemistry and Crystal Physics Faculty of Chemistry, Jagiellonian University, 30-387 Kraków, Poland

[#] Author contributed equally

correspondence: alexander.domling@upol.cz

Table of contents

1	General information	S3
2	Method development	S4
3	Effect of bases, PTC, temperature, solvents	S6
4	Cheminformatic calculation results	S10
5	Price comparison Vs chemical space	S12
6	Effect of nucleophile concentration on overall reaction yield	S14
7	Chiral analysis of the reaction of methyl ester of (S)-2-bromobutyric acid with ada-mantly isocyanide under standard S_N2 reaction condition	S18
8	General procedure A for compound **1a** -**61a**	S20
9	Synthesis of compound **1b**: procedure B	S20
10	Synthesis of compound **1c**: procedure C	S20
11	Synthesis of compound **1d**: procedure D	S20
12	General procedure for the scale-up synthesis of compound **15a**	S21
13	General procedure for the control experiment	S21
14	Supporting information analytical data	S22
15	Supporting Information Spectral data (1H-NMR and C-13 Spectra)	S41
16	List of molecules which were ineffectively synthesized by this method	S106
17	Crystal structure determination	S108
18	Supplementary References	S111

1. General Information.

Reagents were available from commercial suppliers and used without any purification unless otherwise noted. All isocyanides were made *in house* by performing the Ugi procedure (R-NH_2 => R-NHCHO => R-NC or based on our recently published Leukart-Wallach procedure (ACS Comb. Sci. 2015, 17, 9, 493–499). Other reagents were purchased from Sigma Aldrich, ABCR, Acros, Fluorochem and AK Scientific and were used without further purification. Nuclear magnetic resonance spectra were recorded on a Bruker Avance 500 spectrometer. Chemical shifts for 1H NMR were reported relative to TMS (δ 0 ppm) or internal solvent peak ($CDCl_3$ δ 7.26 ppm, CD_3OD δ 3.31 ppm or D_2O δ 4.79 ppm) and coupling constants were in hertz (Hz). The following abbreviations were used for spin multiplicity: s = singlet, d = doublet, t = triplet, dt = double triplet, ddd = doublet of double doublet, m = multiplet, and br = broad. Chemical shifts for ^{13}C NMR reported in ppm relative to the solvent peak ($CDCl_3$ δ 77.23 ppm, DMSO δ 39.52 ppm, CD_3OD δ 49.00 ppm). Flash chromatography was performed on a Grace Reveleris X2 using Grace Reveleris Silica columns (12g) and a gradient of petroleum ether/ethyl acetate (0–100%) or dichloromethane/methanol (0–20%) was applied. Thin layer chromatography was performed on Fluka pre-coated silica gel plates (0.20 mm thick, particle size 25 µm). Mass spectra were measured on a Waters Investigator Supercritical Fluid Chromatograph with a 3100 MS Detector (ESI) using a solvent system of methanol and CO_2 on a Viridis silica gel column (4.6 × 250 mm, 5 µm particle size) and reported as (m/z). High resolution mass spectra (HRMS) were recorded using an LTQ-Orbitrap-XL (Thermo Fisher Scientific; ESI pos. mode) at a resolution of 60000@m/z400. Melting points were obtained on a melting point apparatus and were uncorrected.

2. Method development:

As a model reaction we choose *p*-chlorobenzyl isocyanide and benzyl bromide, a good electrophile in S_N2 reactions and with good visibility of the educts and product in TLC (Supplementary Figure 1). Initially, we run the model reaction in acetonitrile (0.5M) using triethylamine as a base at room temperature, we observed the traces of product formation in TLC-MS analysis. Furthermore, we run the reaction in toluene (0.5M) and dichloroethane (0.5M), but we didn't observe the product formation in TLC-MS. However, we observe the nitrilium ion and imidoyl bromide intermediate formation in the SFC-MS analysis (Supplementary Figure 2) instead of the corresponding amide.

We added water to the reaction, we added different amounts of water (0.1, 05, 1.0, 2.0, 5.0, 10.0 equiv.), and we found that excess of water was harmful for the reaction as isocyanide hydrolysis observed. Overall, with 1.0 equivalent of water in the reaction, we observe 1.0 % product formation and starting material was unchanged even after 3 days in room temperature.

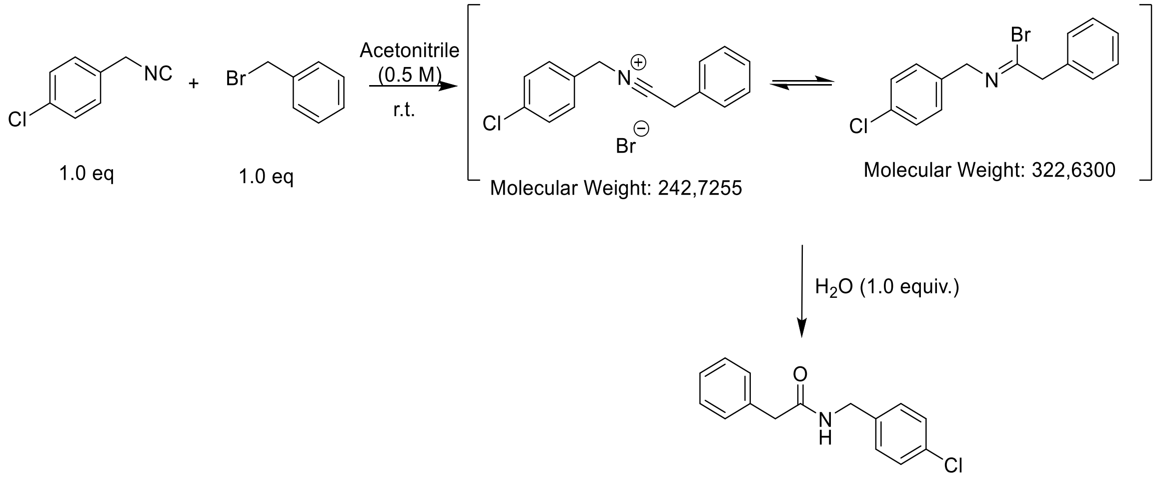

Supplementary Figure 1: The reaction between *p*-chlorobenzyl isocyanide and benzyl bromide in acetonitrile at room temperature without addition of water.

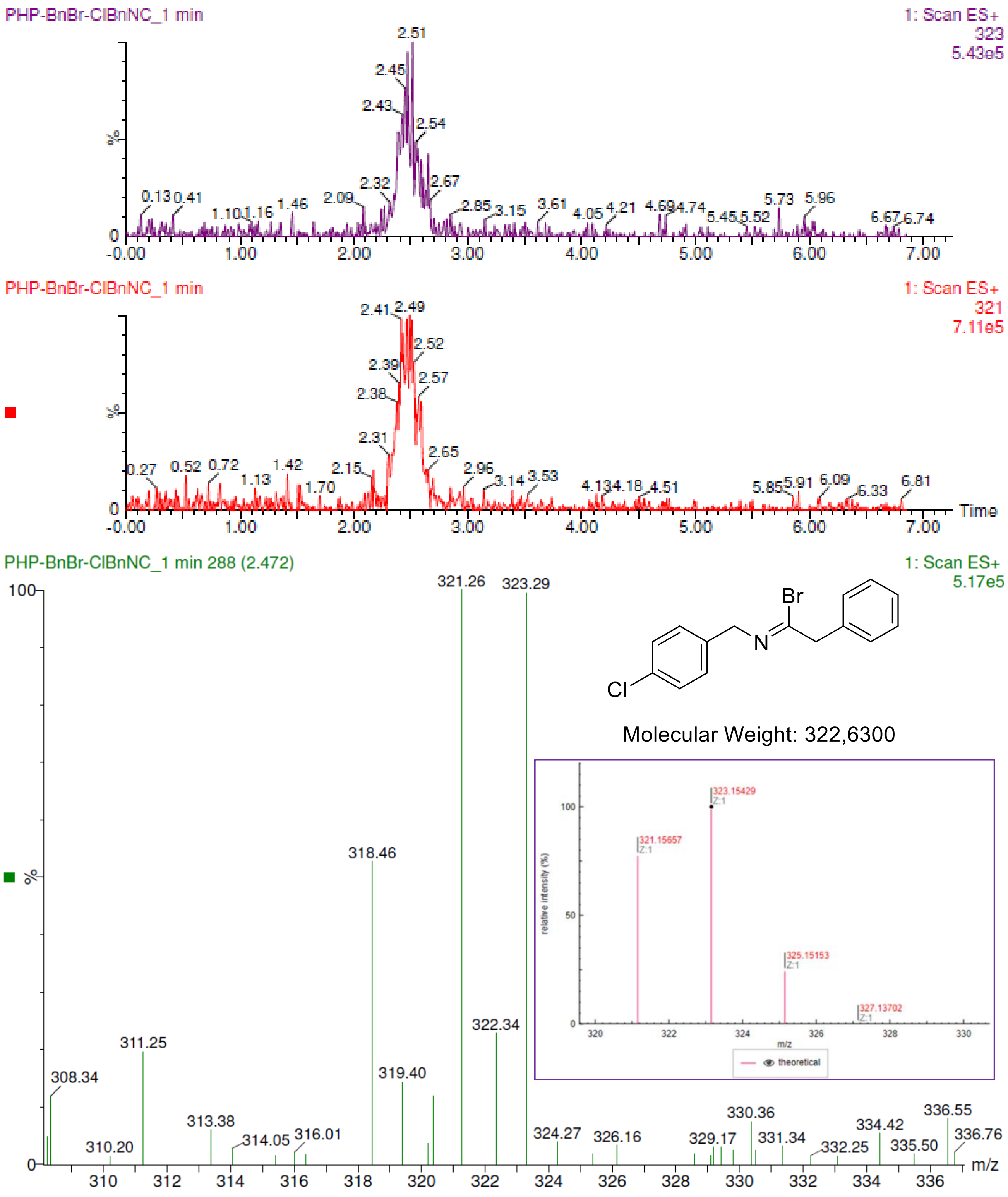

Supplementary Figure 2: SFC-MS analysis electrospray spectra with mass peaks of the reaction between *p*-chlorobenzyl isocyanide and benzyl bromide in acetonitrile at room temperature without addition of water. The measured and calculated (blue inserted box) mol peak region is shown.

3. **Effect of bases, PTC, temperature, solvents**:

In our initial studies we observed that more or less than one equivalent water negatively affect the overall yield of the reaction. For this reason, we use one equitant of water in our next all attempt to improve the overall reaction yield. Further, we tested the addition of different bases: KOH, NaOH, NaOAc, LiOH, Cs_2CO_3, $Ba(OH)_2$, K_2CO_3, $NaHCO_3$, *tert*-BuOK, DBU, DIPEA, Et_3N, DMAP, in combination with three solvents toluene, acetonitrile and dichloroethane, at room temperature as well as at 55 °C (Supplementary Figure 3). In these 60 different reaction we observed product formation of less than 3 % using inorganic bases KOH, NaOH, NaOAc, $Ba(OH)_2$ and organic bases DBU, DIPEA, Et_3N, DMAP. Surprisingly, in the case of K_2CO_3 and $NaHCO_3$ we observed higher 5% and 4% isolated yields. Furthermore, we investigated modifications of conditions by using K_2CO_3 as a base in the reaction in combination of toluene, acetonitrile, and dichloroethane as a solvent **(Supplementary Table 1)** at various temperatures 55 °C, 82 °C and various phase transfer catalyst (PTC) (listed below).

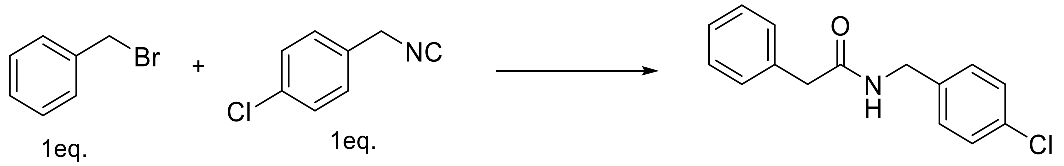

Supplementary Figure 3: General reaction used for method development in 96 well plate with different reaction conditions.

List of phase transfer catalyst tested:

1. Tetrabutylammonium fluoride solution 1.0 M in THF, CAS: 429-41-4
2. Tetrabutylammonium hydroxide solution 40% in water, Cas: 2052-49-5
3. Benzyltributylammonium bromide, CAS: 25316-59-0
4. Tetrabutylammonium bromide, CAS: 1643-15-2
5. Tetrabutylammonium fluoride trihydrate, CAS: 87749-50-6
6. Tetrabutylammonium iodide, CAS: 311-28-4
7. Benzyltriethylammonium chloride, CAS: 56-37-1
8. Tetramethylammonium chloride, CAS: 75-57-0
9. Benzyltrimethylammonium hydroxide solution 40 wt. % in water, CAS: 100-85-6
10. Tetrabutylammonium hydrogensulfate, CAS: 32503-27-8
11. Tetrabutylammonium tribromide CAS: 38932-80-8

Experiment No	Solvent	Base	catalyst	Temperature/ time
Entry 1 To Entry 12	Toluene 0.5 ml + water 10 ml	K_2CO_3	Total 11 PTC + blank	70°C /2days
Entry 13 To Entry 24	Acetonitrile 0.5 ml + water 10 ml	K_2CO_3	Total 11 PTC + blank	70°C /2days
Entry 25 To Entry 36	DCE 0.5 ml + water 10 ml	K_2CO_3	Total 11 PTC + blank	70°C /2days

Supplementary Table 1: Reaction conditions for 36 glass vials on 96-well metal bock.

We used a setup in metal 96-well plate equipped with glass vial. Each vial was equipped with 1:1 stoichiometric amount of benzyl bromide (0.5 mmol, 1 equiv.), *p*-chlorobenzyl isocyanide (0.5 mmol, 1.0 equiv.), K_2CO_3 (1 mmol, 2.0 equiv.) in solvent (0.5 M) as showing in **Supplementary Figure 4**. We run these reactions at room temperature for 48 hr; unfortunately, we didn't observe any improvement as compare to the reaction without PCT. Therefore, next the reactions run at 70 °C; but we didn't observe any difference in the reaction performance at room temperature or 70 °C. Note that, cooling of the reaction in glass vials was accomplished by circulating cold water through the water tubing as shown in **Supplementary Figure 4a**.

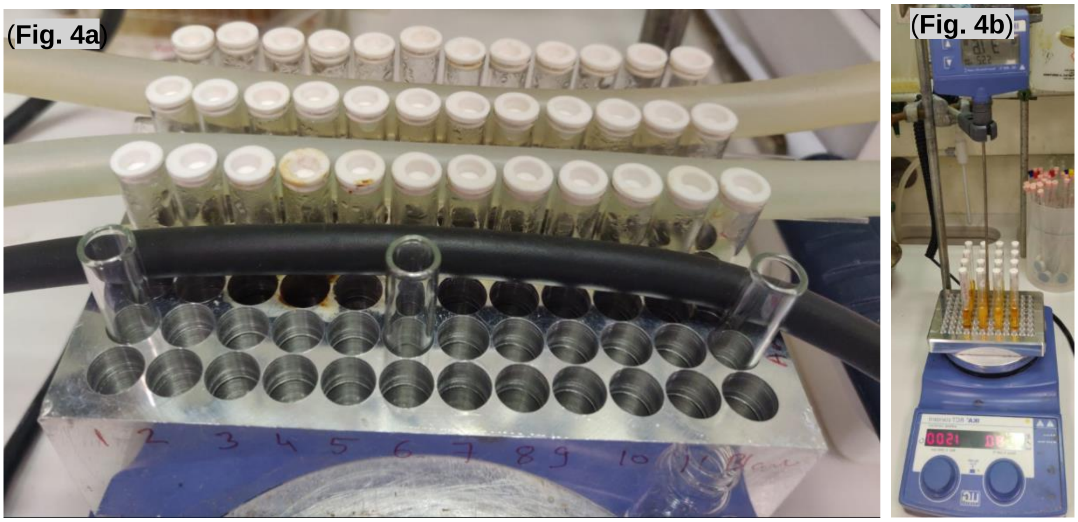

Supplementary Figure 4: High throughput experimentation. Reaction setup for the 11 different PTC's and one reaction without PTC in each set of solvent like toluene, acetonitrile, and dichloroethane (total: 36

reactions at room temperature (**Supplementary Figure 4b**), and then similar reaction in another set of 36 reactions at 82 °C (**Supplementary Figure 4a**).

Qualitative analysis of the reactions was done by TLC; we observed that there was small difference in the reaction yields (less that 1-3 % in acetonitrile), acetonitrile was the best solvent for the reaction; when we used tetrabutylammonium iodide (vial no 6) in acetonitrile at 82 °C we isolated the product in 10% yield. Multiple reaction where check with TLC (**Supplementary Figure 5)** as well as TLC-MS analysis (**Supplementary Figure 6)** .

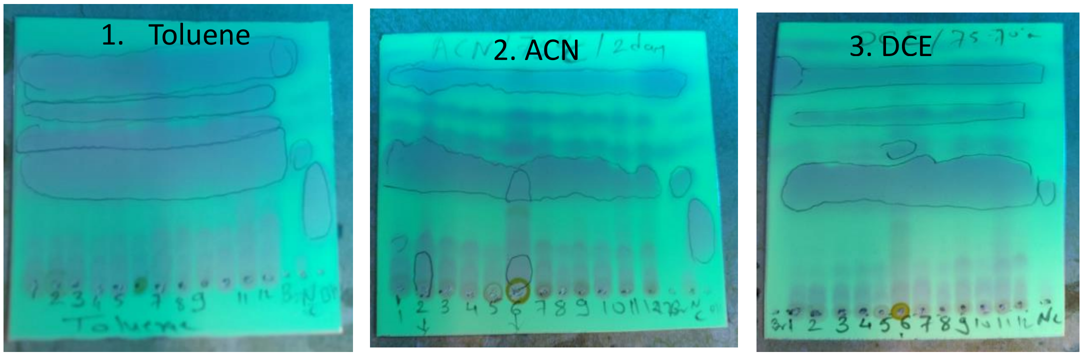

Supplementary Figure 5: HT qualitative TLC analysis. TLC of 36 reactions at 70 °C using 11 PTC's and without PTC in solvents 1st TLC of reactions using toluene as a solvent; 2nd TLC of reactions using acetonitrile as a solvent, in this TLC spot of 6th vial is with tetrabutylammonium iodide (10% yield); 3rd TLC of reactions using dichloroethane as a solvent.

Next, we screened other solvent such as acetone, DMF, DMSO. Unfortunately, we observed hydrolysis product of isocyanide in case of DMF and DMSO. Acetone gave little less yield (7%) as compare to acetonitrile. When we run the reaction with potassium iodide (KI) in 10% mole amount in the reaction instead of PTC we observed a slight yield increase to 12%. When testing various amount of KI in the reaction ranging from 1%, 5%, 10%, 20%, 50%, 1.0 equiv., and 2.0 equiv.), we found that reaction using 20% KI gave better yields (22%).

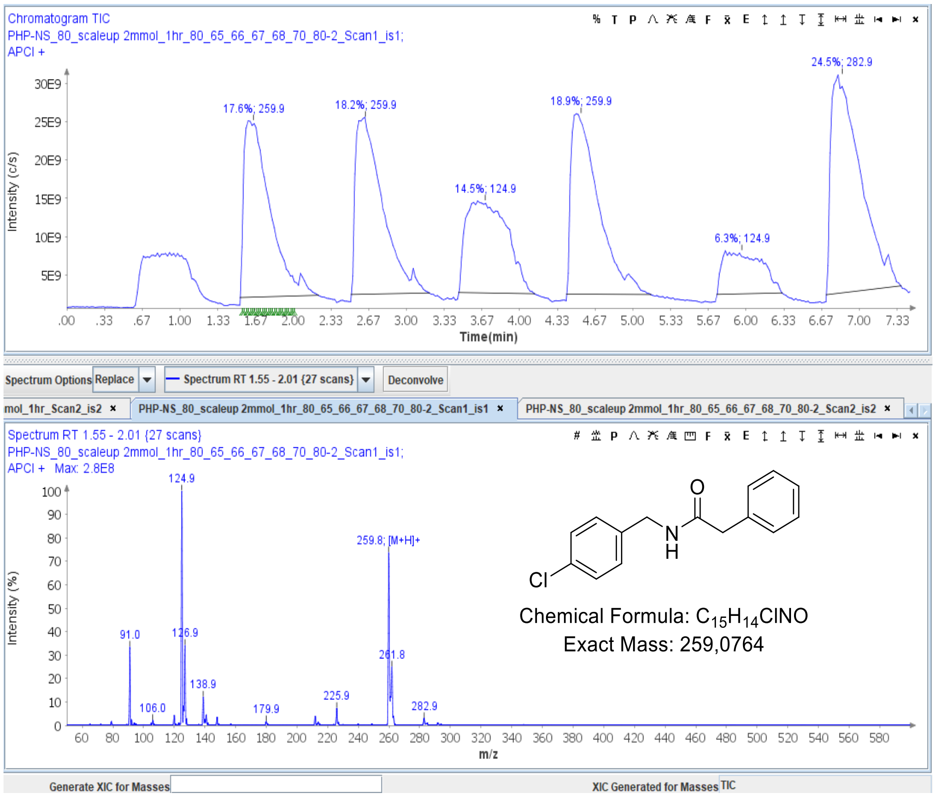

Supplementary Figure 6: HT TLC-MS analysis. Reaction progress was checked with TLC-MS for multiple samples.

Furthermore, we investigated the reaction under microwave conditions and found generally an increase of the % yield of reaction. For this purpose, we run the reaction in 0.5 mmol scale using the benzyl bromide (1.0 equiv.), *p*-chlorobenzyl isocyanide, (1.0 equiv.), K_2CO_3 (2.0 equiv.), KI (20 %), water (1.0 equiv.) in acetonitrile (1 ml) in microwave at 150 °C for 15 min and could isolate the product in 32% yield. While increasing the reactions time to 30 min, 1 hr, and 2 hr, we observed that the vials were either damaged or in some cases the microwave stop running due to sudden excess pressure formation in the vial due to the HBr liberation from imidoyl bromide which reacts vigorously: HBr reacts with the K_2CO_3 forming CO_2. To avoid this problem, we run the reaction with lowering the microwave temperature from 150 °C to lower temperatures. For example, we run reactions at 120 °C, 110 °C, and 105 °C. for 3 hrs. We observed that in these all cases from 150-110 °C excess pressure was observed, and microwave stop in between the reaction progress. Surprisingly, at 105 °C microwave run smoothly without any problem and this gave us 47% yield of the corresponding product. Furthermore, we changed the stoichiometric amounts of the *p*-chlorobenzyl isocyanide from 1.0 to 3.0 equivalent, which did not help to increase the % yield of the product, but instead gave side reactions mostly hydrolyzed isocyanide. However, when we changed the

stoichiometric amounts benzyl bromide, we observed better yield. When we used 2.0 equivalent of benzyl bromide, we obtained 60% yield of the product. (**Supplementary Figure 7**). However, further increasing the stoichiometric amount from 2 equivalents to 3 or 4 equivalents of benzyl bromide was not helpful to increase the % yield of the product. Instead, it gave hydrolyzed product of benzyl bromide to benzyl alcohol as a side product, and excess remaining benzyl bromide is troublesome during reaction workup as it is lachrymatory.

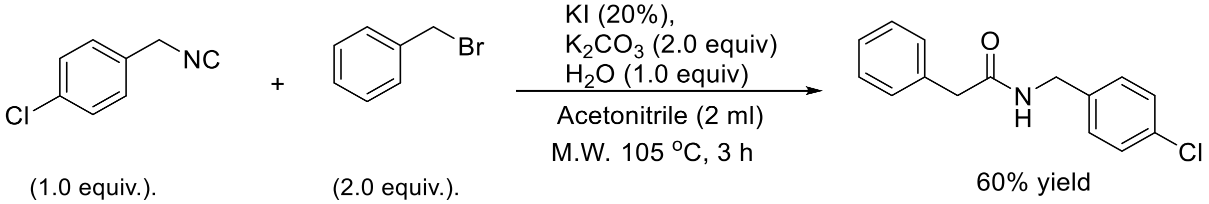

Supplementary Figure 7: Final general optimized reaction condition: *p*-chlorobenzylisocyanide (1.0 equiv.), benzyl bromide (2.0 equiv.), potassium iodide (20 mole %), K_2CO_3 (2.0 equiv.), water (1.0 equiv.), acetonitrile (0.5 M), microwave temperature 105°C, time of reaction 3 hr.

4. Cheminformatic analysis:

Cheminformatic analysis was done using ChemAxon's software's. ChemAxon's REACTOR software[1] used for the virtual reaction. InstantJChem[2] and OpenBabel software[3] were used to remove the duplicate structures from the libraries.

We compared the classical method to form amides from carboxylic acids and amines with our new method obtaining amides from the isocyanides and alkyl halides (RCH_2-X, X= Cl, Br, I); Isocyanides were synthesized from amines (Ugi or Hoffman method), aldehyde, and ketones (Leuckart–Wallach) (Supplementary Figure 8).

Using the TCI America catalog, we extracted all carboxylic acids (1963), primary amines (1951), aldehydes and ketones (719). We excluded bifunctional building blocks, and high molecular weight peptides.

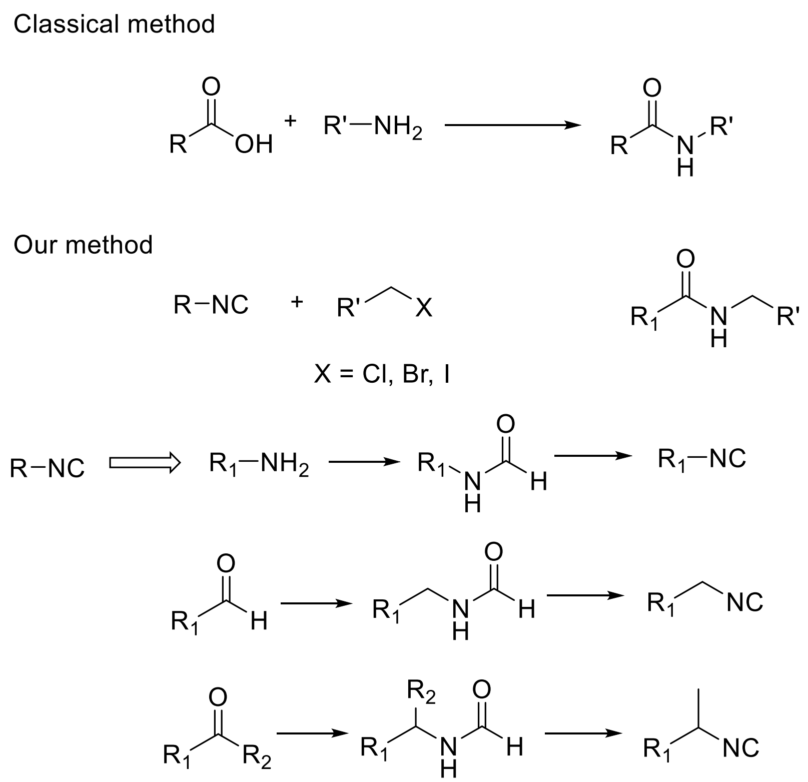

Supplementary Figure 8: Comparison of the classical amide coupling with our method. In our method isocyanide could be obtained from primary amines, anilines, aldehydes and ketones. This gives a versatile chemical space.

Using the REACTOR software (ChemAxon), 1963 carboxylic acids reacting with 1951 amines yielded 3829813 unique amides. Isocyanide synthesis generated novel unique isocyanides (Supplementary Figure 8), from amines (1951), aldehydes (719), and ketones (1990) giving a total of 4660 isocyanides excluding duplicates using the TCI America catalog. These 4660 isocyanide molecules were combined with 921 alkyl halides (RCH_2-X, X= Cl, Br, I) from the TCI America catalog in virtual reaction software REACTOR, giving 4291860 unique amides. Using open babel software both sets (4291860+3829813 = 8121673 molecules) checked for common amides: only 1061736 molecules were in common in both libraries. Thus, only 25% molecules where common in the both libraries. And 75% of the molecules were different. For the synthesis of complicated, novel amides our method is more suitable due to the lack of the starting material needed for the classical amide coupling (see Supplementary figure 9).

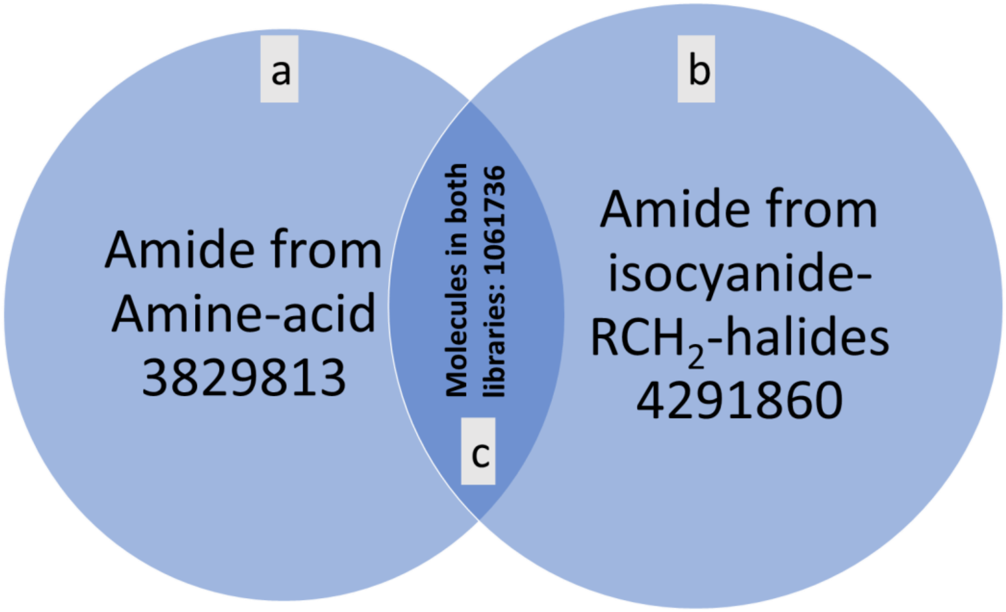

Supplementary Figure 9: Possible synthase building blocks from the TCI America catalog. **a**: number of amide (3829813) formation by classical reaction between acid (1963) and amines (1951) from TCI America catalog: **b**: number of amide (4291860) formation from RCH_2-X (921), and isocyanides which could synthesized from amine (1951), aldehydes (719), and ketones (1990) from the TCI America catalog. **c**: Only 1061736 molecules (~25%) were common in both libraries, this indicates that both libraries gave nearly 75% different chemical space.

5. **Price comparison vs Chemical space**:

Investigating the overlapping and unique chemical space with 200 random amide examples, we surprisingly found that in 104 cases (52%) the corresponding carboxylic acids were not commercially available at all. In the remaining 48%, the average price of the carboxylic acid is 95.4 $/g, which is 2.4 times more expensive than the average price of the corresponding chlorides (40.6 $/g) (**Supplementary Figure 10**).

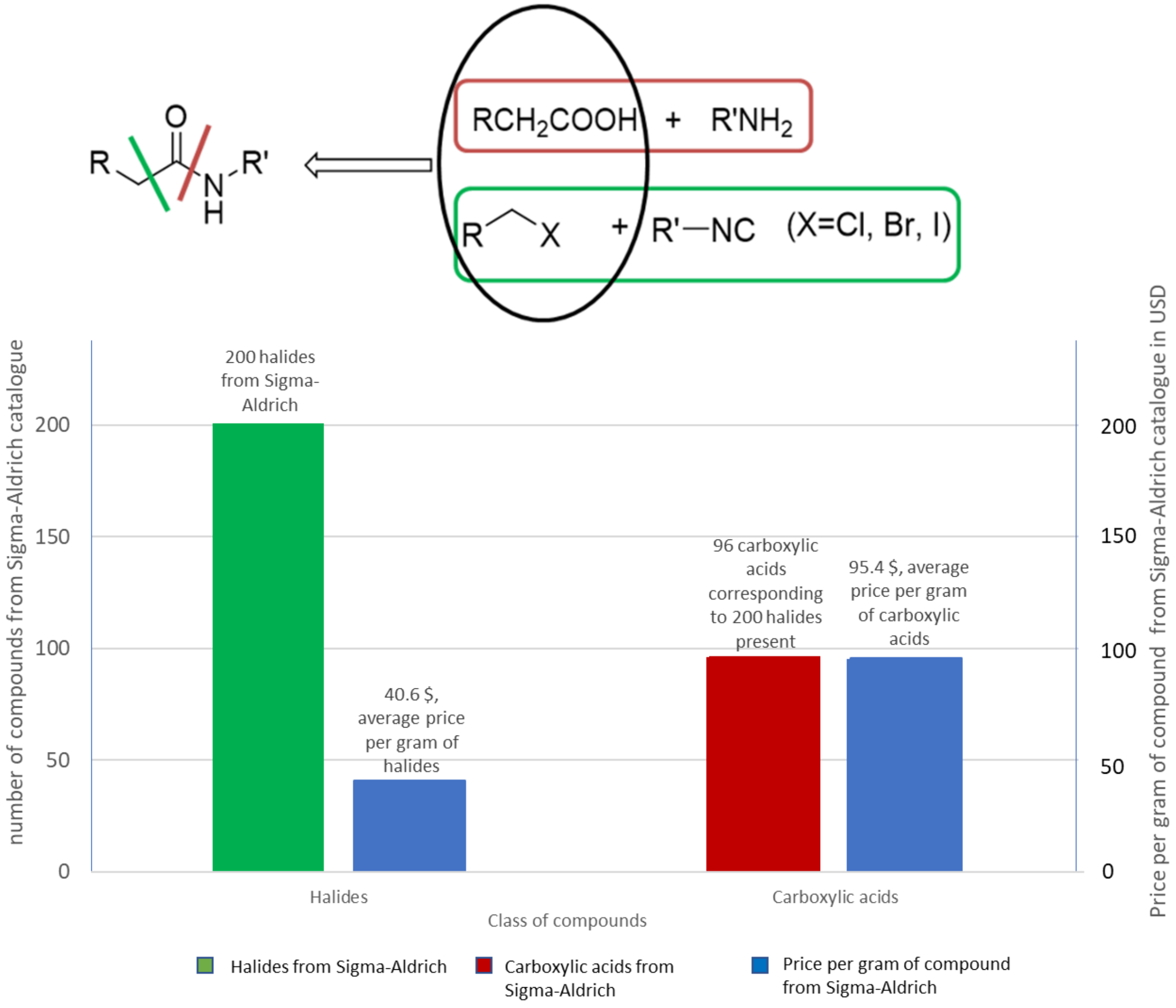

Supplementary Figure 10: Synthesizing the same target amide by both methods, the new S_N2 method is in general more economical. 200 Halides (green color) and 96 corresponding carboxylic acids (Red color) from Sigma-Aldrich, inc. were compared for their prize (blue color). The average price for the carboxylic acids is 135% higher than the corresponding halides.

6. **Effect of nucleophile concentration on overall reaction yield**

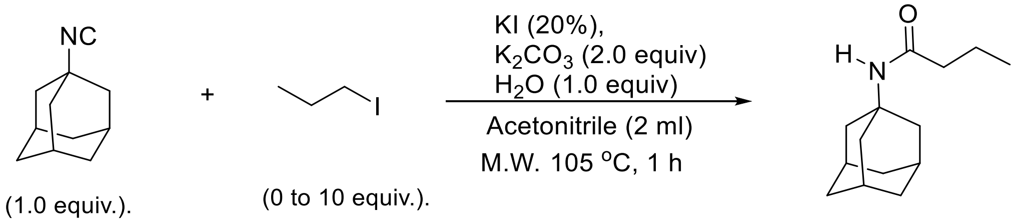

Supplementary figure 11: Reaction between adamantly isocyanide with various concentration of propyl iodide at standard reaction condition for 1hr.

To test the concentration effect of the substrate, we reacted under standard conditions while keeping the adamantly isocyanide concentration constant and measured product formation at different concentration of propyl iodide (Supplementary Figure 11) by ^{1}H NMR. We tested the various concentration of propyl iodide ranging from 0, 0.5, 0.75, 1.0, 2.0, 4.0, 6.0 and 10.0 equivalence. We selected propyl iodide as the reactant because of the low boiling point of propyl iodide and ease to remove unreacted propyl iodide from the reaction mixture under vacuum. After removing the reaction solvents under vacuum, we recorded the proton NMR crude reaction mixtures and compared with the corresponding pure product **17a** (Supplementary Figure 12). We found that rising the concentration of the reactant propyl iodide results in increase of the overall yield of the corresponding product.

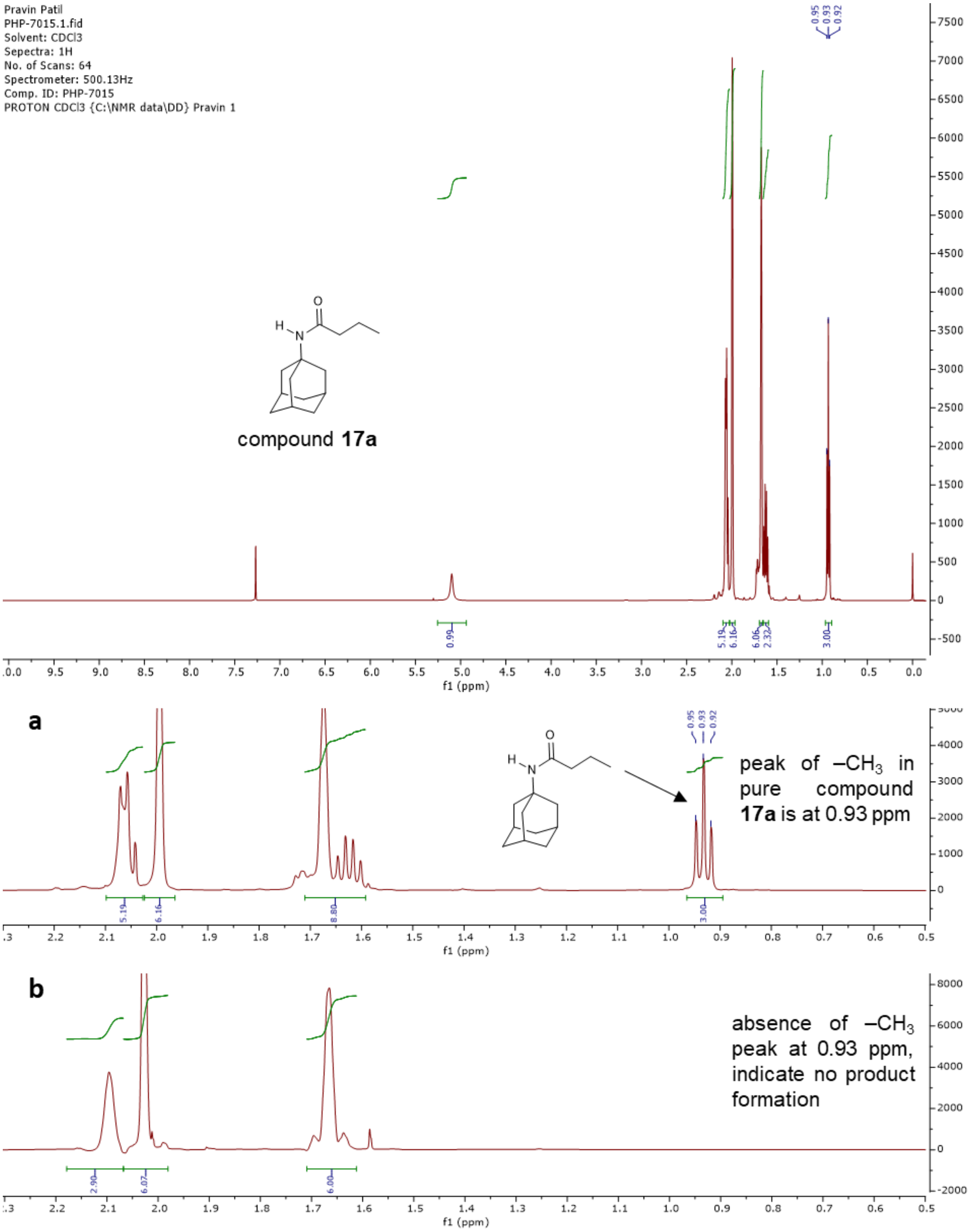

Supplementary Figure 12: Proton NMR of pure isolated product **17a** in the full range of 0.0 to 10.0 ppm and its comparison with proton NMR of reaction without propyl iodide. **a**) Proton NMR of pure isolated

product **17a** in the range of 0.5 to 2.3ppm, indicating the peak of CH_3 of propyl group at 0.93 as triplet. **b**) Proton NMR in the range of 0.5 to 2.3ppm of the crude reaction mixture after 1 hour in microwave without propyl iodide as a nucleophile, it shows that absence of product peak of CH_3 of propyl group at 0.93 as triplet, indicating 0% product formation.

A suitable characteristic product peak is the CH_3 group of the propyl at 0.93 ppm (Supplementary Figure 12a), a triplet which was used to clearly identify product in the crude reaction mixture. Excess unreacted propyl iodide is volatile and was evaporated in vacuum to not interfere with the product peaks.

The crude reaction after 1 hour in microwave without any nucleophile, it shows that absence of product peak of CH_3 of propyl group at 0.93 as triplet, indicating 0% product yield (Supplementary Figure 12b). Similarly, when we run the reaction only for 1 hour in various concentrations of propyl iodide ranging from 0.5, 0.75, 1.0, 2.0, 4.0, 6.0 and 10.0 equivalence, we observed 1%, 9%, 12%, 22%, 34%, 35% and 38% yields respectively (Supplementary Figure 13).

Thus, the NMR study of crude reaction mixture with different halide concentration of reactants show that rising the concentration of halide resulted in to the higher yields in each case, but after a certain concentration side reaction take place. Thus, increasing the concentration of the propyl iodide is rising the overall % yield of the reaction while keeping all other parameter constant. This indicates that this reaction between isocyanide with alkyl halide follows S_N2 reaction characteristics.

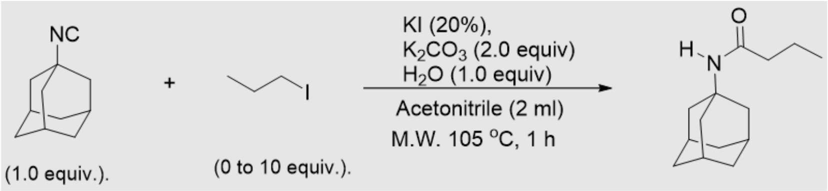

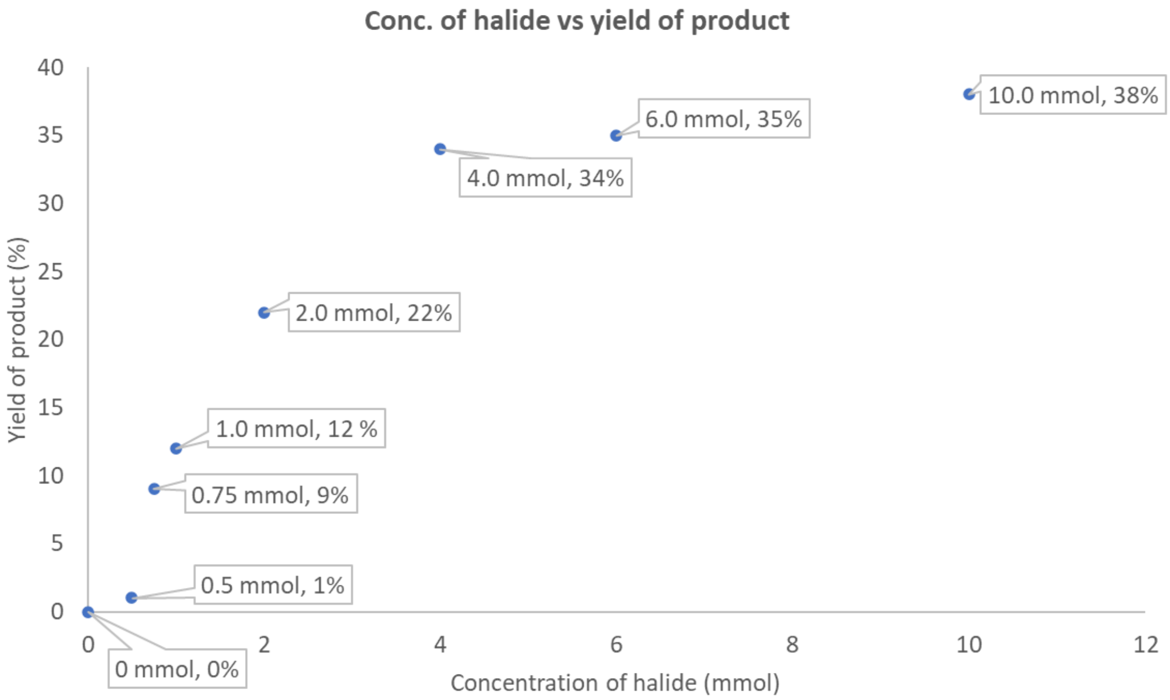

Supplementary Figure 13: Scatter plot of concentration of propyl halide vs % yield of product after 1-hour reaction in the microwave keeping all other reaction parameter constant (standard reaction condition).

7. Chiral analysis of the reaction of methyl ester of (S)-2-bromobutyric acid with adamantly isocyanide under standard S_N2 reaction condition

SFC analysis was performed on a Waters SFC Prep 150 AP system equipped with a 2998 Photodiode Detector (220-300nm) using a Chiralpack IG chiral column 4.0x250mm, 5µm particle size. 100µL of a 1mg/mL sample was injected and eluted with CO2/MeOH 20% isocratic with a flow rate of 4mL/min at 40 °C and 120 bar. Mass was detected with a Waters 3100 Mass Detector using ESI in positive mode.

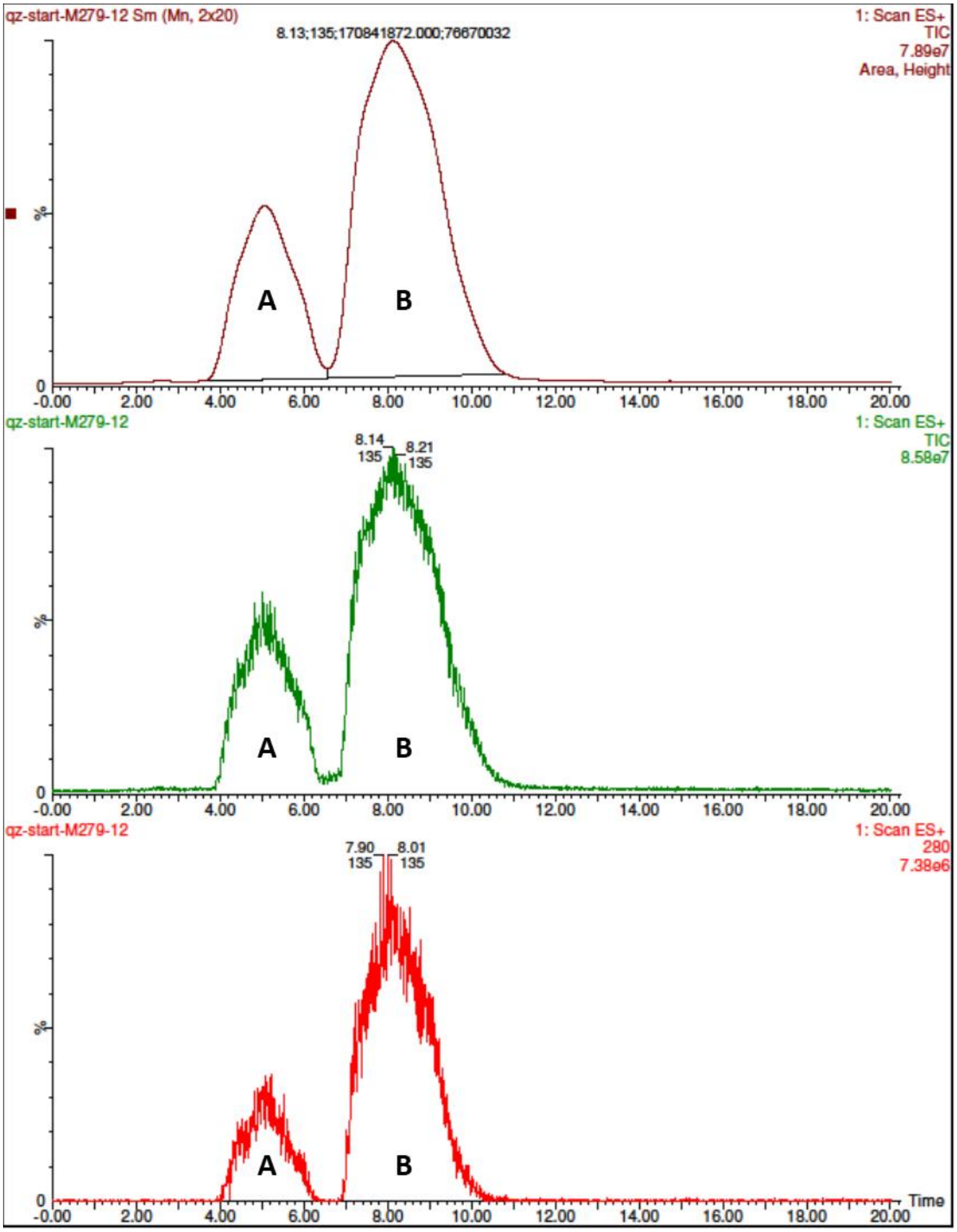

Supplementary Figure 14: Chiral SFC-MS analysis spectra of compound **62a**

	Elution time (min)	Peak Height	Peak Area	Ratio
**Enantiomer A**	5.045	39677912	62768468	0.27
**Enantiomer B**	8.128	76670032	170841872	0.73
**Total Area**			233610340	

Supplementary Table 2: Result of chiral SFC-MS analysis spectra of methyl ester of (S)-2-bromobutyric acid (**62a**)

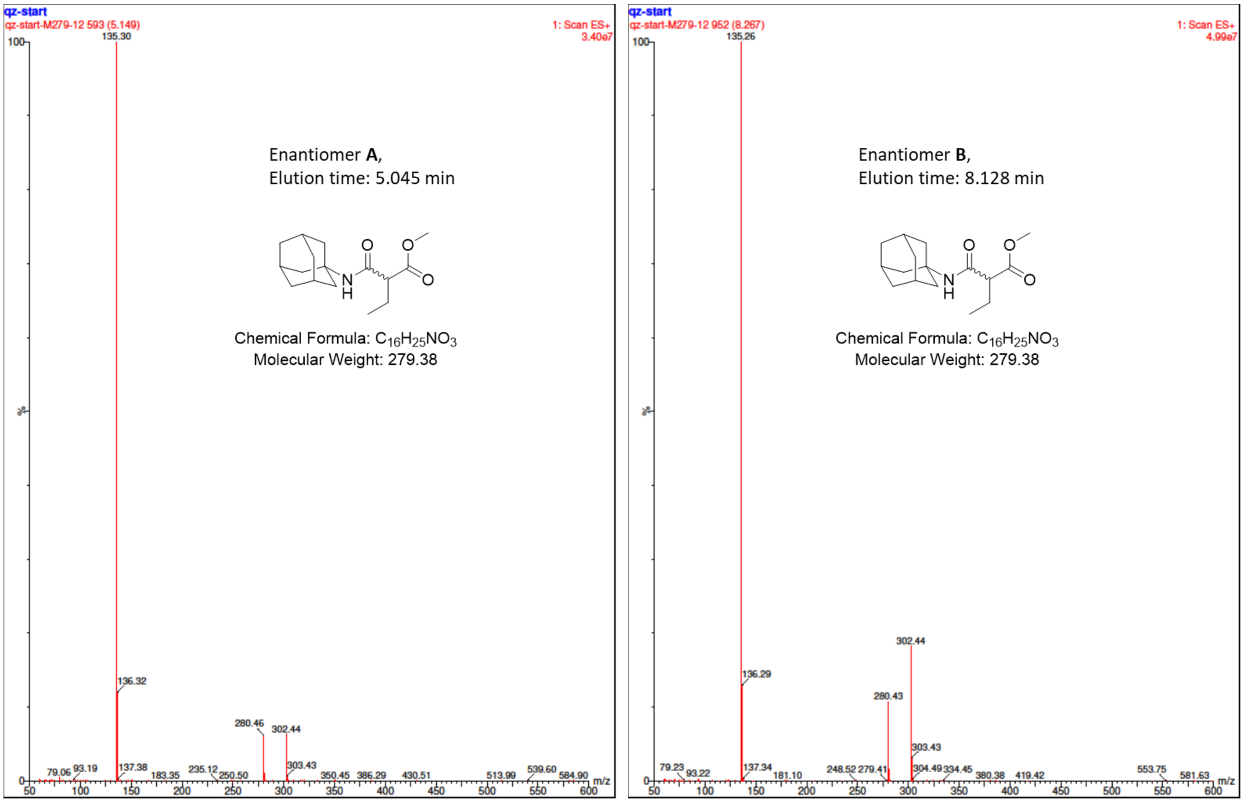

Supplementary Figure 15: Chiral SFC-MS analysis mass peaks of both enantiomers of compound **62a**

8. **General procedure A for compound 1a - 61a**

To a microwave vial equipped with a magnetic stir bar containing isocyanide (1.0 mmol), alkyl methyl halide (2.0 mmol), potassium iodide (0.2 mmol), potassium carbonate (2.0 mmol), H_2O (1.0 mmol) and acetonitrile (0.5 M) were added. The reaction vessel was sealed and irradiated in the cavity of the microwave reactor at the set temperature of 105 °C for 3h. Upon completion of the reaction, the vial was cooled to room temperature. Reaction mixture was diluted with dichloro-methane 5ml and filter off to remove the inorganic solids. Solvents were evaporated under vacuum to give crude reaction product. The crude product was purified by flash chromatography on silica gel to obtained the pure product.

9. **Synthesis of compound 1b: procedure B:**

The mixture of aldehyde (**15a,** 1.0 mmol), *tert*-butyl isocyanide (1.0 mmol), aniline (1.0 mmol) and but-2-ynoic acid (1.0 mmol) in 1.0 mL of methanol was stirring under room temperature for 12 h. The crude product was purified by chromatography on silica gel (petroleum ether/ ethyl acetate, 3:1) to get product **1b** (392 mg, 72%).

10. Synthesis of compound 1c: procedure C:

To a microwave vial equipped with a magnetic stir bar containing pyridine-2-amine (1.0 mmol), cyclohexyl isocyanide (1.0 mmol), aldehyde (**15a,** 1.0 mmol), methanol (1.0 ml) and scandium triflate (0.1 mmol) were added. The reaction vessel was sealed and irradiated in the cavity of the microwave reactor at the set temperature of 100 °C for 2h. Upon completion of the reaction, the vial was cooled with a stream of air. The resulting reaction mixture was diluted with ethyl acetate (50 mL), thoroughly washed with 1M HCl (2×50 mL)2 and saturated Na_2CO_3 (50 mL) aqueous solutions, dried over Na_2SO_4 and concentrated under reduced pressure. The crude product was dissolved in ethyl acetate (5 ml) and subjected to the flash chromatography with silica and ethyl acetate / pentane (3:7) as eluent to give pure **1c** (175 mg, 36%).

11. Synthesis of compound 1d: procedure D:

The mixture of aldehyde (**15a**, 1.0 mmol), isocyanide (1.0 mmol), aniline (1.0 mmol) and $TMSN_3$ (1.0 mmol) in 1.0 mL of methanol was stirring under room temperature for 12 h. The crude product

was purified by chromatography on silica gel (petroleum ether/ ethyl acetate, 3:1) to get product **1d** (221 mg, 39%).

12. General procedure for the scale-up synthesis of compound 15a:

To a microwave vial equipped with a magnetic stir bar containing adamantyl isocyanide (10.0 mmol), aldehyde (10.0 mmol), potassium iodide (1.0 mmol), potassium carbonate (20 mmol), H_2O (10.0 mmol) and acetonitrile (8.0 ml) were added. The reaction vessel was sealed and irradiated in the cavity of the microwave reactor at the set temperature of 105 °C for 5h. Upon completion of the reaction, the vial was cooled under room temperature. The crude product was purified by chromatography on silica gel (petroleum ether/ ethyl acetate, 3:1) to get product **15a** (1.57 g, 51%)

13. General procedure for the control experiment ruling out a radical mechanism

To a microwave vial equipped with a magnetic stir bar containing adamantyl isocyanide (1.0 mmol), 2-(bromomethyl)-1,3-dichlorobenzene (1.0 mmol), potassium iodide (0.2 mmol), potassium carbonate (2.0 mmol), TEMPO (2.0 mmol), H_2O (1.0 mmol) and acetonitrile (2.0 ml) were added. The reaction vessel was sealed and irradiated in the cavity of the microwave reactor at the set temperature of 105 °C for 5h. Upon completion of the reaction, the vial was cooled under room temperature. The crude product was purified by chromatography on silica gel (petroleum ether/ ethyl acetate, 3:1) to get product **1a** (198mg, 59%).

14. Supporting information analytical data:

1a: *N*-(adamantan-1-yl)-2-(2,6-dichlorophenyl)acetamide

The product was obtained using general procedure **A** (X= Br), 1 mmol scale 222 mg, 66% yield

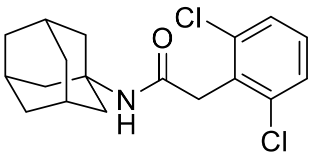

as white solid, M.P.= 196-198 °C; ^{1}H NMR (500 MHz, $CDCl_3$) δ 7.34 (d, *J* = 8.0 Hz, 2H), 7.17 (t, *J* = 8.1 Hz, 1H), 5.18 (s, 1H), 3.85 (s, 2H), 2.05 – 2.02 (m, 3H), 1.97 – 1.94 (m, 6H), 1.66 – 1.63 (m, 6H). ^{13}C NMR (126 MHz, $CDCl_3$) δ 167.1, 136.2, 132.3, 128.8, 128.3, 52.2, 41.5, 39.9, 36.3, 29.4. HRMS (ESI) m/z: $[M + H]^+$ calcd for $C_{18}H_{22}Cl_2NO$, 338.1073; found, 338.1073.

2a: *N*-(adamantan-1-yl)-2-(benzyloxy)acetamide

The product was obtained using general procedure **A** (X= Cl), 1 mmol scale 117 mg, 39% yield

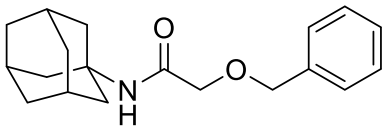

as white solid, M.P.= 176-178 °C; ^{1}H NMR (500 MHz, $CDCl_3$) δ 7.72 – 6.98 (m, 5H), 6.29 (s, 1H), 4.57 (s, 2H), 3.88 (s, 2H), 2.12 – 2.09(m, 3H), 2.04 – 1.98 (m, 6H), 1.74 – 1.65 (m, 6H). ^{13}C NMR (126 MHz, $CDCl_3$) δ 168.4, 137.0, 128.6, 128.2, 127.9, 73.6, 70.1, 51.5, 41.6, 36.3, 29.4. HRMS (ESI) m/z: $[M + H]^+$ calcd for $C_{19}H_{26}NO_2$, 300.1958; found, 300.1957.

3a: *N*-((3*s*,5*s*,7*s*)-adamantan-1-yl)-2-(2-(trifluoromethyl)phenyl)acetamide

The product was obtained using general procedure **A** (X= Br), 1 mmol scale 182 mg, 54% yield

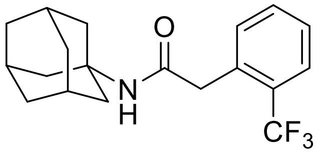

as white solid, M.P.= 161-163 °C; ^{1}H NMR (500 MHz, $CDCl_3$) δ 7.68 (d, *J* = 7.8 Hz, 1H), 7.55 (t, *J* = 7.6 Hz, 1H), 7.49 (d, *J* = 7.7 Hz, 1H), 7.40 (t, *J* = 7.6 Hz, 1H), 5.10 (s, 1H), 3.65 (s, 2H), 2.06 – 2.05 (m, 3H), 1.99 – 1.95 (m, 6H), 1.70 – 1.66 (m, 6H). ^{13}C NMR (126 MHz, $CDCl_3$) δ 168.6, 133.8 (d, *J* = 1.9 Hz), 132.5, 132.1, 128.6 (q, *J* = 29.7 Hz), 127.2, 126.1 (q, *J* = 5.6 Hz), 124.4 (d, *J* = 273.8 Hz), 52.1, 41.5, 41.4, 36.3, 29.4. HRMS (ESI) m/z: $[M + H]^+$ calcd for $C_{19}H_{23}F_3NO$, 338.1726; found, 338.1723.

4a: *N*-(adamantan-1-yl)-2-(3,5-difluorophenyl)acetamide

The product was obtained using general procedure **A** (X= Br), 1 mmol scale 143 mg, 47% yield

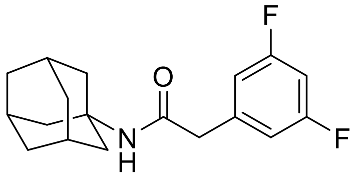

as white solid, M.P.= 163-165 °C; ^{1}H NMR (500 MHz, $CDCl_3$) δ 7.00 – 6.54 (m, 2H), 6.75 – 6.70 (m, 1H), 5.27 (s, 1H), 3.43 (s, 2H), 2.07 – 2.05 (m, 3H), 2.01 – 1.94 (m, 6H), 1.71 – 1.62 (m, *6*H). ^{13}C NMR (126 MHz, $CDCl_3$) δ 168.4, 164.0 (d, *J* = 12.8 Hz), 162.1 (d, *J* = 12.9 Hz), 139.1 (t, *J* = 9.4 Hz), 113.2 – 111.3 (m), 102.6(t, *J* = 25.2 Hz), 52.3, 44.3, 41.5, 36.3, 29.4. HRMS (ESI) m/z: $[M + H]^+$ calcd for $C_{18}H_{22}F_2NO$, 306.1664; found, 306.1655.

5a: *N*-((3*s*,5*s*,7*s*)-adamantan-1-yl)but-3-enamide

The product was obtained using general procedure **A** (X= Br), 1 mmol scale 111 mg, 51% yield

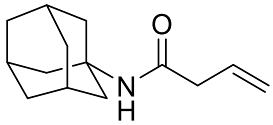

as brown solid, M.P.= 194-196 °C; ^{1}H NMR (500 MHz, $CDCl_3$) δ 5.95 – 5.87 (m, 1H), 5.34 (s, 1H), 5.20 – 5.15 (m, 2H), 2.91 (d, *J* = 7.2 Hz, 2H), 2.11 – 2.01 (m, 3H), 1.98 – 1.94(m, 6H), 1.67 – 1.60 (m, 6H). ^{13}C NMR (126 MHz, $CDCl_3$) δ 169.6, 132.1, 119.2, 51.8, 42.8, 41.5, 36.3, 29.4. HRMS (ESI) m/z: $[M + H]^+$ calcd for $C_{14}H_{22}NO$, 220.1696; found, 220.1692.

6a: *N*-((3*s*,5*s*,7*s*)-adamantan-1-yl)-2-(4-bromophenyl)acetamide

The product was obtained using general procedure **A** (X= Br), 1 mmol scale 146 mg, 42% yield

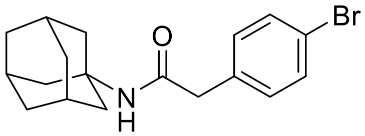

as yellow solid, M.P.= 181-183 °C; ^{1}H NMR (500 MHz, $CDCl_3$) δ 7.46 (d, *J* = 8.1 Hz, 2H), 7.14 (d, *J* = 8.1 Hz, 2H), 5.20 (s, 1H), 3.41 (s, 2H), 2.16 – 1.96 (m, 3H), 1.94 – 1.91 (m, 6H), 1.66– 1.62 (m, 6H). ^{13}C NMR (126 MHz, $CDCl_3$) δ 169.4, 134.5, 131.9, 130.9, 121.0, 52.1, 44.1, 41.5, 36.3, 29.4.

HRMS (ESI) m/z: $[M + H]^+$ calcd for $C_{18}H_{23}BrNO$, 348.0958; found, 348.0953.

7a: *N*-((3*s*,5*s*,7*s*)-adamantan-1-yl)-2-(1H-benzo[d]imidazol-2-yl)acetamide

The product was obtained using general procedure **A** (X= Cl), 1 mmol scale 65 mg, 21% yield as

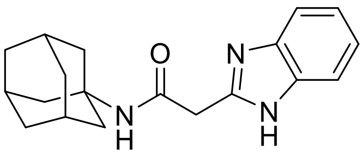

brown solid, M.P.= 160-162 °C; ^{1}H NMR (500 MHz, $CDCl_3$) δ 10.58 (s, 1H), 8.55 (s, 1H), 7.83 – 7.32 (m, 2H), 7.23 (dd, *J* = 6.1, 3.2 Hz, 2H), 4.80 (s, 2H), 2.22 – 2.08 (m, 3H), 2.00 – 1.91 (m, 6H), 1.77 – 1.55 (m, 6H). ^{13}C NMR (126 MHz, $CDCl_3$) δ 163.1, 152.2, 122.5, 119.1, 112.2, 57.9,

41.8, 38.4, 35.7, 29.3. HRMS (ESI) m/z: $[M + H]^+$ calcd for $C_{19}H_{24}N_3O$, 310.1914; found, 310.1908.

8a: *N*-((3*s*,5*s*,7*s*)-adamantan-1-yl)-2-(1,3-dimethyl-1H-pyrazol-5-yl)acetamide

The product was obtained using general procedure **A** (X= Cl), 1 mmol scale 103 mg, 36% yield

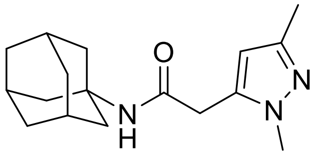

as yellow solid, M.P.= 148-150 °C; ^{1}H NMR (500 MHz, $CDCl_3$) δ 5.91 (s, 1H), 5.22 (s, 1H), 3.72 (s, 3H), 3.45 (s, 2H), 2.22 (s, 3H), 2.09 – 1.98 (m, 3H), 1.96 – 1.87(m, 6H), 1.69 – 1.58 (m, 6H). ^{13}C NMR (126 MHz, $CDCl_3$) δ 167.0, 147.6, 136.9, 105.9, 52.1, 41.4, 36.2, 36.0, 35.1, 29.3, 13.4. HRMS (ESI) m/z: $[M + H]^+$ calcd for $C_{17}H_{26}N_3O$, 288.2070; found, 288.2067.

9a: *N*-(adamantan-1-yl)-2-(5-hydroxy-3*H*-1,2,4-triazol-3-yl)acetamide

The product was obtained using general procedure **A** (X= Cl), 1 mmol scale 50 mg, 18% yield as

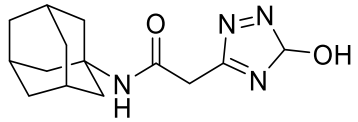

yellow solid, M.P.= 153-155 °C; ^{1}H NMR (500 MHz, DMSO-d_6) δ 11.16 (s, 1H), 7.57 (s, 1H), 3.24 (d, J = 1.0 Hz, 2H), 3.18 (t, J = 7.6 Hz, 1H), 2.03 – 2.00 (m, 3H) 1.96 – 1.88 (m, 6H), 1.66 – 1.58 (m, 6H). ^{13}C NMR (126 MHz, DMSO-d_6) δ 166.0, 156.6, 143.4, 51.5, 41.4, 36.4, 35.1, 29.3. HRMS (ESI) m/z: $[M + H]^+$ calcd for $C_{14}H_{21}N_4O_2$, 277.1659; found, 277.1658.

10a: *N*-((3*s*,5*s*,7*s*)-adamantan-1-yl)-4-methylpent-3-enamide

The product was obtained using general procedure **A** (X= Br), 1 mmol scale 128 mg, 52% yield

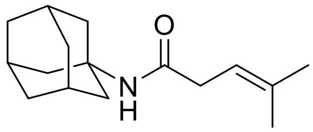

as yellow solid, M.P.= 155-157 °C; ^{1}H NMR (500 MHz, $CDCl_3$) δ 5.36 (s, 1H), 5.31 – 5.27 (m, 1H), 2.87 (d, J = 7.5 Hz, 2H), 2.08 – 2.02 (m, 3H), 2.01 – 1.96 (m, 6H), 1.79 (d, J = 1.5 Hz, 3H), 1.73 – 1.68 (m, 6H), 1.66 (d, J = 1.2 Hz, 3H). ^{13}C NMR (126 MHz, $CDCl_3$) δ 170.4, 137.1, 117.5, 51.6, 41.6, 37.2, 36.4, 29.4, 25.7, 17.9. HRMS (ESI) m/z: $[M + H]^+$ calcd for $C_{16}H_{26}NO$, 248.2009; found, 248.2006.

11a: *N*-(adamantan-1-yl)-2-(7-methoxy-2-oxo-2*H*-chromen-4-yl)acetamide

The product was obtained using general procedure **A** (X= Br), 1 mmol scale 128 mg, 35% yield

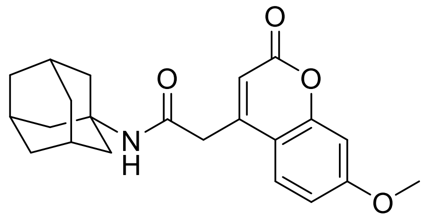

as yellow solid, M.P.= 157-159 °C; ^{1}H NMR (500 MHz, $CDCl_3$) δ 7.61 (d, *J* = 9.1 Hz, 1H), 6.95 – 6.85 (m, 1H), 6.83 (d, *J* = 3.1 Hz, 1H), 6.22 (s, 1H), 5.39 (s, 1H), 3.89 (s, 3H), 3.59 (s, 2H), 2.22 – 2.02 (m, 3H), 2.01 – 1.81 (m, 6H), 1.73 – 1.53 (m, 6H). ^{13}C NMR (126 MHz, $CDCl_3$) δ 166.4, 162.9, 161.0, 155.6, 150.1, 126.0, 113.0, 112.6, 112.5, 101.0, 55.8, 52.6, 41.9, 41.4, 36.2, 29.3. HRMS (ESI) m/z: $[M + H]^+$ calcd for $C_{22}H_{26}NO_4$, 368.1856; found, 368.1859.

12a: *N*-((3*s*,5*s*,7*s*)-adamantan-1-yl)-4-(1,3-dioxoisoindolin-2-yl)butanamide

The product was obtained using general procedure **A** (X= Br), 1 mmol scale 66 mg, 18% yield as

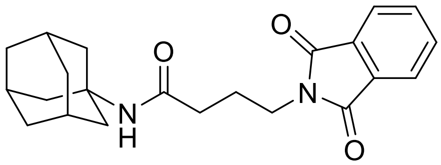

yellow solid, M.P.= 159-161 °C; ^{1}H NMR (500 MHz, $CDCl_3$) δ 7.85 (dd, *J* = 5.4, 3.1 Hz, 2H), 7.74 (d, *J* = 3.0 Hz, 1H), 7.73 (d, *J* = 3.0 Hz, 1H), 5.63 (s, 1H), 3.75 (t, *J* = 6.4 Hz, 2H), 2.13 (t, *J* = 7.0 Hz, 2H), 2.10 – 2.02 (m, 3H), 2.02 – 1.94 (m, 8H), 1.76 – 1.64 (m, 6H). ^{13}C NMR (126 MHz, $CDCl_3$) δ 170.9, 168.7, 134.0, 132.1, 123.3, 51.9, 41.6, 37.3, 36.4, 34.8, 29.4, 25.0. HRMS (ESI) m/z: $[M + H]^+$ calcd for $C_{22}H_{27}N_2O_3$, 367.2016; found, 367.2011.

13a: *N*-((3*s*,5*s*,7*s*)-adamantan-1-yl)-2-(4-(chloromethyl)phenyl)acetamide

The product was obtained using general procedure **A** (X= Cl), 1 mmol scale 101 mg, 32% yield

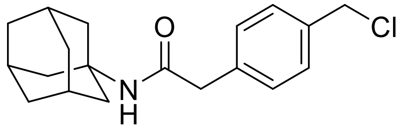

as yellow solid, M.P.= 146-148 °C; ^{1}H NMR (500 MHz, $CDCl_3$) δ 7.40 – 7.34 (m, 2H), 7.26 (d, *J* = 8.1 Hz, 2H), 5.16 (s, 1H), 4.60 (s, 2H), 3.48 (s, 2H), 2.14 – 1.97 (m, 3H), 1.95 – 1.92 (m, 6H), 1.67 – 1.63 (m, 6H). ^{13}C NMR (126 MHz, $CDCl_3$) δ 169.6, 136.3, 135.9, 129.6, 129.1, 52.0, 45.9, 44.5, 41.5, 36.3, 29.4. HRMS (ESI) m/z: $[M + H]^+$ calcd for $C_{19}H_{25}ClNO$, 318.1616; found, 318.1619.

14a: *N*-((3*s*,5*s*,7*s*)-adamantan-1-yl)-4-cyanobutanamide

The product was obtained using general procedure **A** (X= Br), 1 mmol scale 84 mg, 34% yield as white solid, M.P.= 142-144 °C; ^{1}H NMR (500 MHz, $CDCl_3$) δ 5.33 (s, 1H), 2.45 (t, *J* = 6.9 Hz, 2H), 2.27 (t, *J* = 7.0 Hz, 2H), 2.13 – 2.07 (m, 3H), 2.02 –1.98 (m, 6H), 1.97 – 1.93 (m, 2H), 1.71 – 1.67 (m, 6H). ^{13}C NMR (126 MHz, $CDCl_3$) δ 169.9, 119.5, 52.2, 41.6, 36.3, 35.0, 29.4, 21.1, 16.5. HRMS (ESI) m/z: $[M + H]^+$ calcd for $C_{15}H_{23}N_2O$, 247.1805; found, 247.1800.

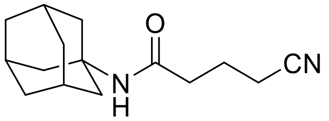

15a: *N*-((3*s*,5*s*,7*s*)-adamantan-1-yl)-2-(5-formylthiophen-3-yl)acetamide

The product was obtained using general procedure **A** (X= Cl), 1 mmol scale 154 mg, 51% yield as yellow solid, M.P.= 143-145 °C; ^{1}H NMR (500 MHz, $CDCl_3$) δ 9.89 (s, 1H), 7.75 (s, 1H), 7.56 (s, 1H), 5.41 (s, 1H), 3.50 (s, 2H), 2.16 – 2.02 (m, 3H), 2.03 – 1.94 (m, 6H), 1.71 – 1.62 (m, 6H). ^{13}C NMR (126 MHz, $CDCl_3$) δ 183.0, 168.5, 143.9, 137.5, 137.1, 132.5, 52.3, 41.5, 38.7, 36.3, 29.4. HRMS (ESI) m/z: $[M + H]^+$ calcd for $C_{17}H_{22}NO_2S$, 304.1366; found, 304.1366.

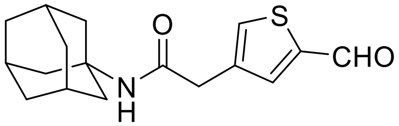

16a: *N*-((3*s*,5*s*,7*s*)-adamantan-1-yl)-3-phenylpropanamide

The product was obtained using general procedure **A** (X= Br), 1 mmol scale 147 mg, 52% yield as yellow solid, M.P.= 149-151 °C; ^{1}H NMR (500 MHz, $CDCl_3$) δ 7.34 – 7.27 (m, 2H), 7.25 – 7.19 (m, 3H), 5.03 (s, 1H), 2.95 (t, *J* = 7.6 Hz, 2H), 2.40 (t, *J* = 7.6 Hz, 2H), 2.11 – 2.06 (m, 3H), 1.99 – 1.91 (m, 6H), 1.74 – 1.65 (m, 6H). ^{13}C NMR (126 MHz, $CDCl_3$) δ 171.2, 141.1, 128.5, 126.1, 51.8, 41.6, 39.5, 36.3, 31.9, 29.42. HRMS (ESI) m/z: $[M + H]^+$ calcd for $C_{19}H_{26}NO$, 284.2009; found, 284.2009.

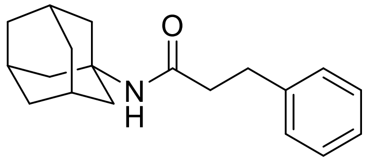

17a: *N*-((3*s*,5*s*,7*s*)-adamantan-1-yl)butyramide

The product was obtained using general procedure **A** (X= I), 1 mmol scale 179 mg, 81% yield as white solid, M.P.= 109-111 °C; ^{1}H NMR (500 MHz, $CDCl_3$) δ 5.10 (s, 1H), 2.10 – 2.03 (m, 5H), 2.02 – 1.96 (m, 6H), 1.70 – 1.65 (m, 6H), 1.62 (q, *J* = 7.4 Hz, 2H), 0.93 (t, *J* = 7.4 Hz, 3H); 1^{3}C NMR (126 MHz,

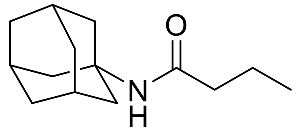

$CDCl_3$) δ 172.1, 51.5, 41.5, 39.5, 36.2, 29.3, 19.1, 13.5; HRMS (ESI) m/z calcd for $C_{14}H_{24}NO$ $[M+H]^+$: 222.1852; found: 222.1851.

18a: *N*-((3*s*,5*s*,7*s*)-adamantan-1-yl)-2-(4-formylphenyl)acetamide

The product was obtained using general procedure **A** (X= Br), 1 mmol scale 50 mg, 17% yield as

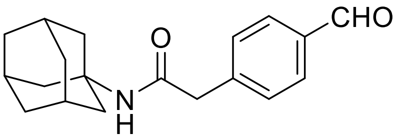

yellow solid, M.P.= 144-146 °C; ^{1}H NMR (500 MHz, $CDCl_3$) δ 10.01 (s, 1H), 7.87 (d, *J* = 7.4 Hz, 2H), 7.45 (d, *J* = 7.8 Hz, 2H), 5.22 (s, 1H), 3.55 (s, 2H), 2.07 – 2.06 (m, 3H), 1.98 – 1.96 (m, 6H), 1.69 – 1.66 (m, 6H); ^{13}C NMR (126 MHz, $CDCl_3$) δ 191.8, 168.7, 142.6, 135.2, 130.2, 129.9, 52.3, 44.9, 41.5, 36.3, 29.4. HRMS (ESI) m/z: $[M + H]^+$ calcd for $C_{19}H_{24}NO_2$, 298.1802; found, 298.1800.

19a: *N*-((3*s*,5*s*,7*s*)-adamantan-1-yl)-2-(quinolin-8-yl)acetamide

The product was obtained using general procedure **A** (X= Br), 1 mmol scale 105 mg, 33% yield

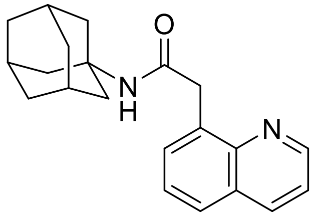

as brown solid, M.P.= 139-141 °C; ^{1}H NMR (500 MHz, $CDCl_3$) δ 8.99 – 8.89 (m, 1H), 8.28 – 8.17 (m, 1H), 7.77 (dd, *J* = 6.8, 3.5 Hz, 2H), 7.57 – 7.50 (m, 1H), 7.50 – 7.43 (m, 1H), 7.23 (s, 1H), 4.09 (s, 2H), 2.04 – 1.97 (m, 3H), 1.92 – 1.84 (m, 6H), 1.67 – 1.55 (m, 6H). ^{13}C NMR (126 MHz, $CDCl_3$) δ 170.6, 149.2, 146.4, 136.9, 134.9, 130.7, 128.7, 127.1, 126.8, 121.1, 51.5, 42.1, 41.5, 36.4, 29.4. HRMS (ESI) m/z: $[M + H]^+$ calcd for $C_{21}H_{25}N_2O$, 321.1961; found, 321.1960.

20a: *N*-((3*s*,5*s*,7*s*)-adamantan-1-yl)-2-phenylacetamide

The product was obtained using general procedure **A** (X= Br), 1 mmol scale 94 mg, 35% yield as

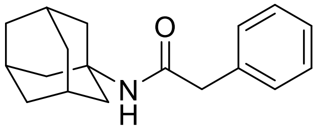

yellow solid, M.P.= 151-153 °C; ^{1}H NMR (500 MHz, $CDCl_3$) δ 7.39 – 7.34 (m, 2H), 7.31 – 7.25 (m, 3H), 5.11 (s, 1H), 3.50 (s, 2H), 2.11 – 1.99 (m, 3H), 1.97 – 1.92 (m, 6H), 1.69 – 1.63 (m, 6H).^{13}C NMR (126 MHz, $CDCl_3$) δ 170.1, 135.6, 129.3, 128.9, 127.1, 51.9, 45.0, 41.5, 36.3, 29.4.
HRMS (ESI) m/z: $[M + H]^+$ calcd for $C_{18}H_{24}NO$, 270.1852; found, 270.1853.

21a: *N*-((3*s*,5*s*,7*s*)-adamantan-1-yl)acetamide

The product was obtained using general procedure **A** (X= I), 1 mmol scale 126 mg, 65% yield as

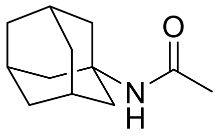

white solid, M.P.= 143-145 °C; ^{1}H NMR (500 MHz, $CDCl_3$) δ 5.13 (s, 1H), 2.08 – 2.03 (m, 3H), 2.00 – 1.96 (m, 6H), 1.90 (s, 3H), 1.68 – 1.65 (m, 6H); ^{13}C NMR (126 MHz, $CDCl_3$) δ 169.2, 51.6, 41.4, 36.2, 29.3, 24.5; HRMS (ESI) m/z calcd for $C_{12}H_{20}NO$ [M+H]+: 194.1539; found: 194.1540.

22a: *N*-((3s,5s,7s)-adamantan-1-yl)-3-(benzyl(1-phenoxypropan-2-yl)amino)propanamide

The product was obtained using general procedure **A** (X= Cl), 1 mmol scale 178 mg, 40% yield

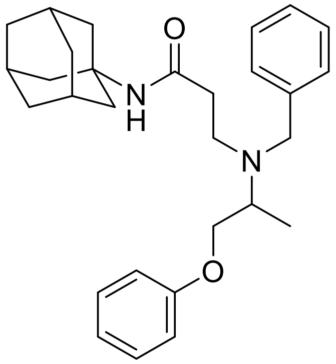

as brown solid, M.P.= 146-148 °C; ^{1}H NMR (500 MHz, $CDCl_3$) δ 7.40 – 7.33 (m, 4H), 7.32 – 7.30 (m, 3H), 6.97 (t, *J* = 7.4 Hz, 1H), 6.90 (d, *J* = 8.1 Hz, 2H), 6.88 (s, 1H), 4.16 – 4.04 (m, 1H), 3.95 – 3.86 (m, 1H), 3.81 (d, *J* = 13.8 Hz, 1H), 3.70 (d, *J* = 13.9 Hz, 1H), 3.38 – 3.31 (m, 1H), 3.05 – 2.76 (m, 2H), 2.29 (t, *J* = 6.0 Hz, 2H), 2.10 –2.02 (m, 3H), 2.00 – 1.94 (m, 6H), 1.77 – 1.57 (m, 6H), 1.22 (d, *J* = 6.8 Hz, 3H). ^{13}C NMR (126 MHz, $CDCl_3$) δ 171.5, 158.7, 139.8, 129.5, 128.9, 128.4, 127.2, 120.8, 114.5, 70.0, 54.8, 53.7, 51.6, 47.2, 41.6, 36.4, 35.5, 29.5, 12.5. HRMS (ESI) m/z: $[M + H]^+$ calcd for $C_{29}H_{39}N_2O_2$, 447.3006; found, 447.2999.

23a: *N*-(4-chlorobenzyl)acetamide

The product was obtained using general procedure **A** (X= I), 1 mmol scale 119 mg, 67% yield as

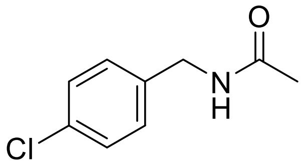

white solid, M.P.= 132-134 °C; ^{1}H NMR (500 MHz, $CDCl_3$) δ 7.29 (dd, *J* = 8.2, 2.0 Hz, 2H), 7.20 (dd, *J* = 8.2, 2.0 Hz, 2H), 5.94 (s, 1H), 4.38 (d, *J* = 5.6 Hz, 2H), 2.01 (s, 3H); ^{13}C NMR (126 MHz, $CDCl_3$) δ 170.2, 136.8, 133.0, 128.9, 128.6, 42.7, 22.9; HRMS (ESI) m/z calcd for $C_9H_{11}ClNO$ $[M+H]^+$: 184.0524; found: 184.0523.

24a: *N*-(4-fluorobenzyl)acetamide

The product was obtained using general procedure **A** (X= I), 1 mmol scale 105 mg, 63% yield as

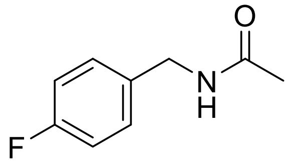

white solid, M.P.= 88-90°C; ^{1}H NMR (500 MHz, $CDCl_3$) δ 7.30 – 7.16 (m, 2H), 7.07 – 6.94 (m, 2H), 5.88 (s, 1H), 4.39 (d, *J* = 5.8 Hz, 2H), 2.02 (s, 3H); ^{13}C NMR (126 MHz, $CDCl_3$) δ 170.13, 161.98 (d, *J* = 245.4 Hz), 134.08 (d, *J* = 3.2 Hz), 129.27 (d, *J* = 8.1 Hz), 115.30 (d, *J* = 21.4 Hz), 42.74, 22.95; HRMS (ESI) m/z calcd for $C_9H_{11}FNO$ $[M+H]^+$: 168.0819; found: 168.0819.

25a: *N*-(4-methylbenzyl)acetamide

The product was obtained using general procedure **A** (X= I), 1 mmol scale 86 mg, 53% yield as

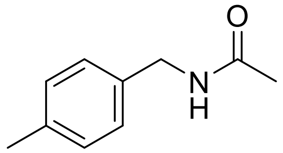

white solid, M.P.= 110-113 °C^{1}H NMR (500 MHz, $CDCl_3$) δ 7.18 (d, *J* = 8.2 Hz, 2H), 7.14 (d, *J* = 8.2 Hz, 2H), 5.66 (s, 1H), 4.39 (d, *J* = 5.6 Hz, 2H), 2.34 (s, 3H), 2.01 (s, 3H); ^{13}C NMR (126 MHz, $CDCl_3$) δ 170.0, 137.2, 135.3, 129.4, 127.9, 43.5, 23.2, 21.1; HRMS (ESI) m/z calcd for $C_{10}H_{14}NO$ $[M+H]^+$: 164.1070; found: 164.1070.

26a: *N*-(thiophen-2-ylmethyl)acetamide

The product was obtained using general procedure **A** (X= I), 1 mmol scale 70 mg, 45% yield as liquid; ^{1}H

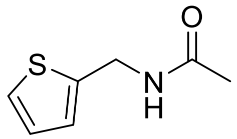

NMR (500 MHz, $CDCl_3$) δ 7.21 (dd, *J* = 5.0, 1.1 Hz, 1H), 6.97 – 6.92 (m, 2H), 6.00 (s, 1H), 4.58 (d, *J* = 5.6 Hz, 2H), 1.99 (s, 3H); ^{13}C NMR (126 MHz, $CDCl_3$) δ 170.1, 141.0, 126.6, 125.7, 124.8, 38.0, 22.7; HRMS (ESI) m/z calcd for $C_7H_{10}NOS$ $[M+H]^+$: 156.0478; found: 156.0479.

27a: *N*-phenethylacetamide

The product was obtained using general procedure **A** (X= I), 1 mmol scale 95 mg, 58% yield as

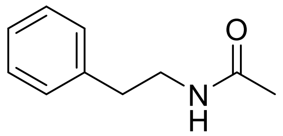

yellow oil; ^{1}H NMR (500 MHz, $CDCl_3$) δ 7.34 – 7.29 (m, 2H), 7.25 – 7.21 (m, 1H), 7.21 – 7.17 (m, 2H), 5.63 (s, 1H), 3.51 (q, *J* = 6.5 Hz, 2H), 2.81 (t, *J* = 7.0 Hz, 2H), 1.93 (s, 3H); ^{13}C NMR (126 MHz, $CDCl_3$) δ 170.1, 138.8, 128.6, 128.4, 126.3, 40.6, 35.5, 23.0; HRMS (ESI) m/z calcd for $C_{10}H_{14}NO$ $[M+H]^+$: 164.1070; found: 164.1070.

28a: *N*-(furan-2-ylmethyl)acetamide

The product was obtained using general procedure **A** (X= I), 1 mmol scale 95 mg, 68% yield as white solid; M.P. = 166-168 °C; ^{1}H NMR (500 MHz, $CDCl_3$) δ 7.34 (d, *J* = 2.0 Hz, 1H), 6.32 (t, *J* = 3.3, 2.0 Hz, 1H), 6.23 (d, *J* = 3.3 Hz, 1H), 5.86 (s, 1H), 4.42 (d, *J* = 5.6 Hz, 2H), 2.00 (s, 3H); ^{13}C NMR (126 MHz, $CDCl_3$) δ 170.3, 151.2, 141.8, 110.2, 107.1, 36.2, 22.6; HRMS (ESI) m/z calcd for $C_2H_{10}NO_2$ $[M+H]^+$: 140.0706; found: 140.0704.

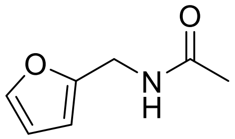

29a: *N*-(3-phenoxybenzyl)acetamide

The product was obtained using general procedure **A** (X= I), 1 mmol scale 157 mg, 65% yield as semisolid; ^{1}H NMR (500 MHz, $CDCl_3$) δ 7.36 – 7.31 (m, 2H), 7.29 – 7.25 (m, 1H), 7.11 (d, *J* = 7.4 Hz, 1H), 7.06 – 6.97 (m, 3H), 6.95 – 6.86 (m, 2H), 5.84 (s, 1H), 4.40 (d, *J* = 5.8 Hz, 2H), 2.02 (s, 3H; ^{13}C NMR (126 MHz, $CDCl_3$) δ 170.2, 157.3, 156.8, 140.3, 129.8, 129.6, 123.2, 122.2, 118.8, 117.8, 117.4, 43.1, 22.9; HRMS (ESI) m/z calcd for $C_{15}H_{16}NO_2$ $[M+H]^+$: 242.1176; found: 242.1174.

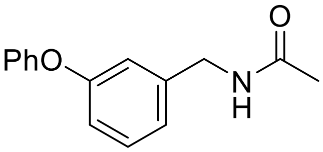

30a: *N*-(2-(benzyloxy)benzyl)acetamide

The product was obtained using general procedure **A** (X= I), 1 mmol scale 79 mg, 31% yield as white solid, M.P.= 96--98 °C; ^{1}H NMR (500 MHz, $CDCl_3$) δ 7.44 – 7.38 (m, 4H), 7.38 – 7.33 (m, 1H), 7.29 (d, *J* = 7.3 Hz, 1H), 7.28 – 7.23 (m, 1H), 6.97 – 6.91 (m, 2H), 5.97 (s, 1H), 5.11 (s, 2H), 4.47 (d, *J* = 5.8 Hz, 2H), 1.91 (s, 3H); ^{13}C NMR (126 MHz, $CDCl_3$) δ 169.7, 156.5, 136.7, 129.6, 128.7, 128.6, 128.0, 127.2, 126.6, 120.9, 111.6, 69.9, 39.3, 23.1; HRMS (ESI) m/z calcd for $C_{16}H_{18}NO_2$ $[M+H]^+$: 256.1334; found: 256.1332

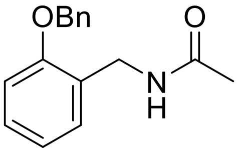

31a: *N*-(4-isopropylphenyl)acetamide

The product was obtained using general procedure **A** (X= I), 1 mmol scale 73 mg, 41% yield as white solid, M.P.= 117-119 °C; ^{1}H NMR (500 MHz, $CDCl_3$) δ 7.40 (d, *J* = 8.2 Hz, 2H), 7.25 – 7.01 (m, 3H), 2.98 – 2.71 (m, 1H), 2.16 (s, 3H), 1.24 (d, *J* = 6.9 Hz, 6H); ^{13}C NMR (126 MHz, $CDCl_3$) δ 168.6, 144.9, 135.6, 126.7, 120.2, 33.5, 24.3, 23.9; HRMS (ESI) m/z calcd for $C_{11}H_{16}NO$ $[M+H]^+$: 178.1226; found: 178.1226.

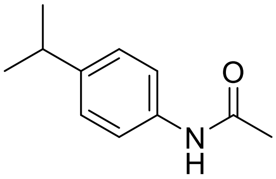

32a: *N*-(6-methoxypyridin-3-yl)acetamide

The product was obtained using general procedure **A** (X= I), 1 mmol scale 60 mg, 36% yield as

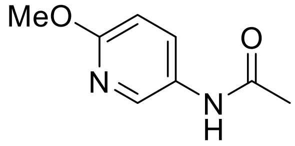

white solid, M.P.= 80-90 °C; ^{1}H NMR (500 MHz, $CDCl_3$) δ 8.22 (t, *J* = 2.8 Hz, 1H), 7.34 – 7.18 (m, 1H), 6.55 – 6.38 (m, 1H), 3.55 (s, 3H), 2.14 (s, 3H); ^{13}C NMR (126 MHz, $CDCl_3$) δ 169.0, 161.3, 159.2, 135.7, 130.3, 119.8, 38.1, 23.7; HRMS (ESI) m/z calcd for $C_8H_{11}N_2O_2$ $[M+H]^+$: 167.0815; found: 167.0814.

33a: *N*-(3,5-dimethoxyphenyl)acetamide

The product was obtained using general procedure **A** (X= I), 1 mmol scale 131 mg, 67% yield as white

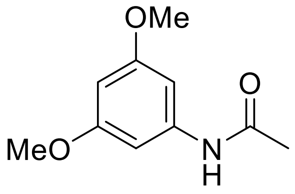

solid, M.P.= 146-148 °C; ^{1}H NMR (500 MHz, $CDCl_3$) δ 7.45 (s, 1H), 6.76 (d, *J* = 2.2 Hz, 2H), 6.23 (d, *J* = 2.2 Hz, 1H), 3.76 (s, 6H), 2.15 (s, 3H); ^{13}C NMR (126 MHz, $CDCl_3$) δ 168.6, 160.9, 139.8, 105.4, 98.1, 96.5, 55.3, 24.6; HRMS (ESI) m/z calcd for $C_{10}H_{14}NO_3$ $[M+H]^+$: 16.0968; found: 196.0968.

34a: *N*-(naphthalen-1-yl)acetamide

The product was obtained using general procedure **A** (X= I), 1 mmol scale 103 g, 56% yield as

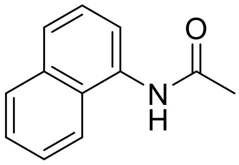

white solid, M.P.= 140-142 °C; ^{1}H NMR (500 MHz, $CDCl_3$) δ 7.94 – 7.84 (m, 3H), 7.63 – 7.41 (m, 5H), 2.32 (s, 3H); ^{13}C NMR (126 MHz, $CDCl_3$) δ 169.3, 134.0, 132.3, 128.5, 126.7, 126.1, 125.9, 125.9, 125.5, 121.6, 121.1, 24.0; HRMS (ESI) m/z calcd for $C_{12}H_{12}NO$ $[M+H]^+$: 186.0913; found: 186.0914.

35a: *N*-(4-phenoxyphenyl)acetamide

The product was obtained using general procedure **A** (X= I), 1 mmol scale 91 mg, 40% yield as white solid,

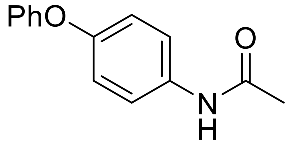

M.P.= 127-129 °C; ^{1}H NMR (500 MHz, $CDCl_3$) δ 7.48 (d, *J* = 8.5 Hz, 3H), 7.34 (t, *J* = 7.8 Hz, 2H), 7.11 (t, *J* = 7.4 Hz, 1H), 7.04 – 6.96 (m, 4H), 2.19 (s, 3H); ^{13}C NMR (126 MHz, $CDCl_3$) δ 168.8, 157.4, 153.3, 133.5, 129.6, 123.0, 121.8, 119.4, 118.3, 24.2; HRMS (ESI) m/z calcd for $C_{14}H_{14}NO_2$ $[M+H]^+$: 228.1019; found: 228.1018.

36a: *N*-(3,4-dichlorophenyl)acetamide

The product was obtained using general procedure **A** (X= I), 1 mmol scale 132 mg, 65% yield as white solid, M.P.= 111-113 °C; ^{1}H NMR (500 MHz, $CDCl_3$) δ 7.77 (d, *J* = 2.2 Hz, 1H), 7.69 (s, 1H), 7.41 – 7.32 (m, 2H), 2.20 (s, 3H); ^{13}C NMR (126 MHz, $CDCl_3$) δ 169.2, 137.5, 132.5, 130.3, 127.2, 121.6, 119.2, 24.3; HRMS (ESI) m/z calcd for $C_8H_8Cl_2NO$ $[M+H]^+$: 203.9977; found: 203.9978.

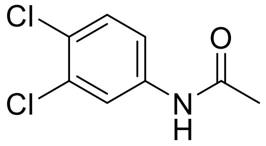

37a: *N*-(2-chloro-4-fluorophenyl)acetamide

The product was obtained using general procedure **A** (X= I), 1 mmol scale 103 mg, 65% yield as white solid, M.P.= 108-110 °C; ^{1}H NMR (500 MHz, $CDCl_3$) δ 8.34 – 8.25 (m, 1H), 7.50 (s, 1H), 7.13 (d, *J* = 8.1 Hz, 1H), 7.05 – 6.94 (m, 1H), 2.24 (s, 3H); ^{13}C NMR (126 MHz, $CDCl_3$) δ 168.3, 158.4 (d, *J* = 247.4 Hz), 13105 (d, *J* = 3.4 Hz), 123.3 (d, *J* = 8.3 Hz), 116.1 (d, *J* = 25.9 Hz), 114.4 (d, *J* = 21.7 Hz), 24.4; HRMS (ESI) m/z calcd for C_8H_8ClFNO $[M+H]^+$: 188.0273; found: 188.0274.

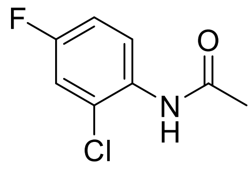

38a: *N*-(3-(diethylamino)propyl)acetamide methyl idiodide salt

The product was obtained using general procedure **A** (X= I), 1 mmol scale 82 mg, 26% yield as liquid; ^{1}H NMR (500 MHz, MeOD) δ 3.53 – 3.48 (m, 4H), 3.45 – 3.40 (m, 2H), 3.37 (d, *J* = 7.1 Hz, 2H), 3.13 (s, 3H), 2.14 – 2.00 (m, 5H), 1.48 – 1.42 (m, 6H); ^{13}C NMR (126 MHz, MeOD) δ 173.6, 59.7, 57.9, 37.3, 23.5, 8.3; HRMS (ESI) m/z calcd for $C_9H_{21}NO_2$ $[M+H]^+$: 173.1648; found: 173.1649.

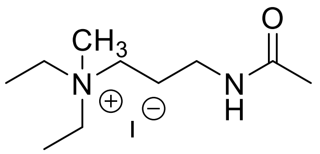

39a: *N*-(3-morpholinopropyl)acetamide methyliodide salt

The product was obtained using general procedure **A** (X= I), 1 mmol scale 236 mg, 72% yield as liquid; ^{1}H NMR (500 MHz, MeOD) δ 2.81 – 2.68 (m, 4H), 2.35 – 2.21 (m, 6H), 2.03 (ddd, *J* = 7.0, 4.7, 1.5 Hz, 2H), 1.99 (s, 3H), 0.81 – 0.73 (m, 2H), 0.71 (s, 3H); ^{13}C NMR (126 MHz, MeOD) δ 173.3, 64.2, 61.6, 61.1, 47.9, 37.1, 23.2, 22.9; HRMS (ESI) m/z calcd for $C_{10}H_{21}N_2O_2$ $[M+CH_3]^+$: 201.1598; found: 201.1596.

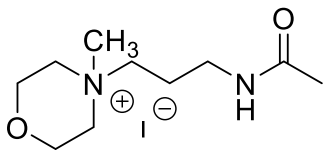

40a: Methyl 2-acetamido-2-methylpropanoate

The product was obtained using general procedure **A** (X= I), 1 mmol scale 113 mg, 71% yield as liquid; ^{1}H NMR (500 MHz, $CDCl_3$) δ 6.06 (s, 1H), 3.75 (s, 3H), 1.98 (s, 3H), 1.55 (s, 6H); ^{13}C NMR (126 MHz, $CDCl_3$) δ 174.9, 169.6, 55.8, 52.1, 24.6, 22.8; HRMS (ESI) m/z calcd for $C_7H_{14}NO$ $[M+H]^+$: 160.0968; found: 160.0968.

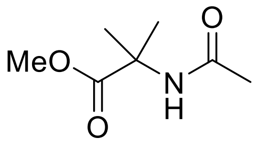

41a: Methyl acetyl leucinate

The product was obtained using general procedure **A** (X= I), 1 mmol scale 65 mg, 35% yield as liquid; ^{1}H NMR (500 MHz, $CDCl_3$) δ 6.10 (d, *J* = 8.5 Hz, 1H), 4.64 (dd, *J* = 8.5, 4.8 Hz, 1H), 3.73 (s, 3H), 2.02 (s, 3H), 1.74 – 1.59 (m, 2H), 1.59 – 1.48 (m, 1H), 0.94 (dd, *J* = 6.3, 2.7 Hz, 6H); ^{13}C NMR (126 MHz, $CDCl_3$) δ 173.7, 169.9, 52.1, 50.6, 41.4, 24.7, 22.9, 22.6, 21.8; HRMS (ESI) m/z calcd for $C_9H_{18}NO_3$ $[M+H]^+$: 188.1281; found: 188.1282.

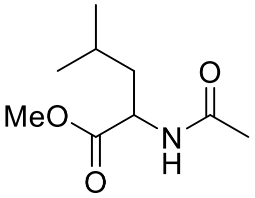

42a: *N*-(2,2-diphenylethyl)acetamide

The product was obtained using general procedure **A** (X= I), 1 mmol scale 120 mg, 50% yield as semisolid; ^{1}H NMR (500 MHz, $CDCl_3$) δ 7.32 – 7.27 (m, 5H), 7.23 – 7.18 (m, 5H), 5.55 (d, *J* = 5.8 Hz, 1H), 4.18 (d, *J* = 8.0 Hz, 1H), 3.87 (dd, *J* = 8.0, 5.8 Hz, 2H), 1.85 (s, 3H); ^{13}C NMR (126 MHz, $CDCl_3$) δ 170.1, 141.8, 128.5, 127.8, 126.5, 50.3, 43.7, 22.9; HRMS (ESI) m/z calcd for $C_{16}H_{18}NO$ [M+H]+: 240.1383; found: 240.1383.

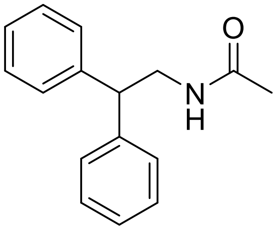

43a: *N*-(2-chloro-6-fluorobenzyl)butyramide

The product was obtained using general procedure **A** (X= I), 1 mmol scale 124 mg, 54% yield as white solid, M.P.= 116-118 °C; ^{1}H NMR (500 MHz, $CDCl_3$) δ 7.28 – 7.20 (m, 2H), 7.06 – 7.00 (m, 1H), 5.74 (s, 1H), 4.66 (d, *J* = 5.6 Hz, 2H), 2.18 (d, *J* = 7.5 Hz, 2H), 1.73 – 1.61 (m, 2H), 0.94 (d, *J* = 7.4 Hz, 3H); ^{13}C NMR (126 MHz, $CDCl_3$) δ 172.5, 161.6 (d, *J* = 250.4 Hz), 135.5 (d, *J* = 5.5 Hz), 129.6 (d, *J* = 9.6 Hz), 125.4 (d, *J* = 3.6 Hz), 123.9 (d, *J* = 18.1 Hz), 114.3 (d, *J* = 22.9 Hz), 38.4, 34.8 (d, *J* = 4.0 Hz), 19.0, 13.7; HRMS (ESI) m/z calcd for $C_{11}H_{14}ClFNO$ $[M+H]^+$: 230.0742; found: 230.0741.

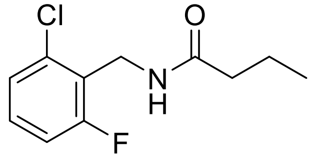

44a: *N*-(3,5-dimethoxyphenyl)butyramide

The product was obtained using general procedure **A** (X= I), 1 mmol scale 120 mg, 54% yield as semi solid; ^{1}H NMR (500 MHz, $CDCl_3$) δ 7.42 (s, 1H), 6.79 (d, *J* = 2.3 Hz, 2H), 6.22 (t, *J* = 2.3 Hz, 1H), 3.76 (s, 6H), 2.32 (d, *J* = 7.4 Hz, 2H), 1.74 (p, *J* = 7.4 Hz, 2H), 0.99 (t, *J* = 7.4 Hz, 3H); ^{13}C NMR (126 MHz, $CDCl_3$) δ 171.81, 160.8, 139.9, 97.9, 96.4, 55.1, 39.4, 18.9, 13.6; HRMS (ESI) m/z calcd for $C_{12}H_{18}NO_3$ $[M+H]^+$: 224.1281; found: 224.1283.

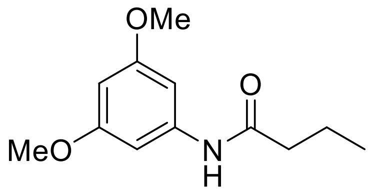

45a: *N*-(4-chlorobenzyl)butyramide

The product was obtained using general procedure **A** (X= I), 1 mmol scale 85 mg, 40% yield as yellow solid, M.P.= 68-70 °C; ^{1}H NMR (500 MHz, $CDCl_3$) δ 7.28 (d, *J* = 8.3 Hz, 2H), 7.20 (d, *J* = 8.3 Hz, 2H), 5.84 (s, 1H), 4.39 (d, *J* = 5.9 Hz, 2H), 2.19 (t, *J* = 7.5 Hz, 2H), 1.72 – 1.63 (m, 2H), 0.95 (t, *J* = 7.4 Hz, 3H); ^{13}C NMR (126 MHz, $CDCl_3$) δ 173.0, 137.1, 133.0, 128.9, 128.6, 42.6, 38.4, 19.1, 13.7; HRMS (ESI) m/z calcd for $C_{11}H_{14}ClNO$ $[M+H]^+$: 212.0837; found: 212.0832.

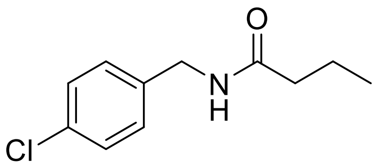

46a: *N*-(4-chlorobenzyl)pentanamide

The product was obtained using general procedure **A** (X= I), 1 mmol scale 70 mg, 31% yield as pale yellow liquid; ^{1}H NMR (500 MHz, $CDCl_3$) δ 7.26 (d, *J* = 8.5 Hz, 2H), 7.17 (d, *J* = 8.5 Hz, 2H), 6.28 (s, 1H), 4.35 (d, *J* = 5.9 Hz, 2H), 2.19 (d, *J* = 7.7 Hz, 2H), 1.66 – 1.53 (m, 2H), 1.33 (h, *J* = 7.4 Hz, 2H), 0.91 (d, *J* = 7.5 Hz, 3H); ^{13}C NMR (126 MHz, $CDCl_3$) δ 173.2, 137.1, 133.0, 128.9, 128.6, 42.6, 36.3, 27.7, 22.3, 13.7; HRMS (ESI) m/z calcd for $C_{12}H_{16}ClNO$ $[M+H]^+$: 226.0993; found: 226.0991.

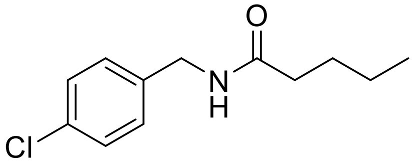

47a: *N*-(1-(3-fluorophenyl)cyclopropyl)-2-(4-formylphenyl)acetamide

The product was obtained using general procedure **A** (X= Br), 1 mmol scale 107 mg, 36% yield as red solid, M.P.= 156-158 °C; ^{1}H NMR (500 MHz, $CDCl_3$) δ 10.01 (s, 1H), 7.87 (d, *J* = 8.1 Hz, 2H), 7.46 (d, *J* = 7.9 Hz, 2H), 7.23 – 7.19 (m, 1H), 6.98 – 6.74 (m, 3H), 3.64 (s, 2H), 1.24 (dt, *J* = 7.3, 2.4 Hz, 4H). ^{13}C NMR (126 MHz, $CDCl_3$) δ 191.8, 170.1, 162.9 (d, *J* = 245.3 Hz), 144.9 (d, *J* = 7.1 Hz), 141.7, 135.5, 130.3, 129.9, 129.8, 120.6 (d, *J* = 2.9 Hz), 113.3 (d, *J* = 21.1 Hz), 112.5 (d, *J* = 22.8 Hz), 43.8, 34.9 18.3. HRMS (ESI) m/z: $[M + H]^+$ calcd for $C_{18}H_{17}FNO_2$, 298.1238; found, 298.1236.

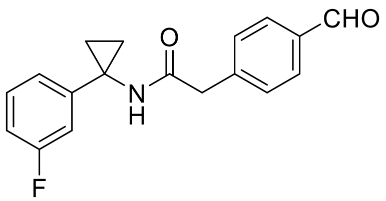

48a: *N*-(4-chlorobenzyl)-2-phenylacetamide

The product was obtained using general procedure **A** (X= Br), 1 mmol scale 155 mg, 60% yield as pale yellow solid, M.P.= 108-110 °C; ^{1}H NMR (500 MHz, $CDCl_3$) δ 7.38 – 7.33 (m, 2H), 7.33 – 7.29 (m, 1H), 7.28 – 7.23 (m, 4H), 7.10 (d, *J* = 8.4 Hz, 2H), 6.22 (t, *J* = 6.0 Hz, 1H), 4.33 (d, *J* = 6.0 Hz, 2H), 3.59 (s, 2H); ^{13}C NMR (126 MHz, $CDCl_3$)

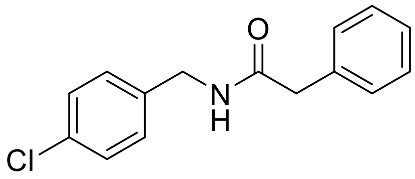

δ 171.0, 136.7, 134.7, 129.2, 128.9, 128.7, 128.6, 127.3, 43.5, 42.7; HRMS (ESI) m/z calcd for $C_{15}H_{15}ClNO$ $[M+H]^+$: 260.0837; found: 260.0836.

49a: *N*-(4-chlorobenzyl)but-3-enamide

The product was obtained using general procedure **A** (X= Br), 1 mmol scale 61 mg, 29% yield as

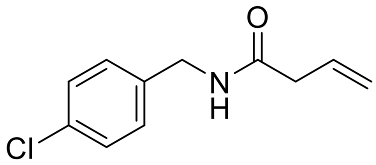

white solid, M.P.= 89-91 °C; ^1H NMR (500 MHz, $CDCl_3$) δ 7.33 (d, *J* = 8.5 Hz, 2H), 7.23 (d, *J* = 8.5 Hz, 2H), 6.02 – 5.92 (m, 2H), 5.31 – 5.21 (m, 2H), 4.42 (d, *J* = 5.9 Hz, 2H), 3.08 (d, *J* = 7.0 Hz, 2H); ^{13}C NMR (126 MHz, $CDCl_3$) δ 170.5, 136.7, 133.2, 131.1, 129.0, 128.7, 119.8, 42.8, 41.4; HRMS (ESI) m/z calcd for $C_{11}H_{13}ClNO$ $[M+H]^+$: 210.0680; found: 210.0682.

50a: *N*-(*tert*-butyl)-2-phenylacetamide

The product was obtained using general procedure **A** (X= Br), 1 mmol scale 78 mg, 41% yield as liquid;

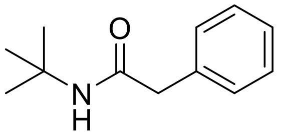

^1H NMR (500 MHz, $CDCl_3$) δ 7.42 – 7.34 (m, 3H), 7.33 – 7.27 (m, 2H), 5.22 (s, 1H), 3.51 (s, 2H), 1.31 (s, 9H); ^{13}C NMR (126 MHz, $CDCl_3$) δ 170.2, 135.4, 129.2, 128.9, 127.1, 51.2, 44.9, 28.6; HRMS (ESI) m/z calcd for $C_{12}H_{18}NO$ $[M+H]^+$: 192.1383; found: 192.1384.

51a: *N*-(2-chloro-6-fluorobenzyl)but-3-enamide

The product was obtained using general procedure **A** (X= Br), 1 mmol scale 109 mg, 48% yield as white

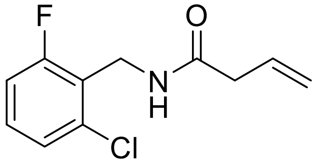

solid, M.P.= 92-94 °C; ^1H NMR (500 MHz, $CDCl_3$) δ 7.26 – 7.18 (m, 2H), 7.04 – 6.99 (m, 1H), 5.96 – 5.88 (m, 2H), 5.28 – 5.14 (m, 2H), 4.63 (d, *J* = 5.6 Hz, 2H), 3.01 (dd, *J* = 7.1, 1.3 Hz, 2H); ^{13}C NMR (126 MHz, $CDCl_3$) δ 170.04, 161.46 (d, *J* = 250.3 Hz), 135.43 (d, *J* = 5.4 Hz), 131.12, 129.57 (d, *J* = 9.7 Hz), 125.30 (d, *J* = 3.6 Hz), 119.52, 114.23 (d, *J* = 22.9 Hz), 41.18, 34.80 (d, *J* = 4.0 Hz); HRMS (ESI) m/z calcd for $C_{11}H_{12}ClFNO$ $[M+H]^+$: 228.0586; found: 228.0586.

52a: *N*-(2,6-dimethylphenyl)-2-phenylacetamide

The product was obtained using general procedure **A** (X= Br), 1 mmol scale 69 mg, 29% yield as solid,

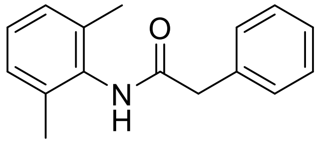

M.P.= 143-145 °C; ^1H NMR (500 MHz, $CDCl_3$) δ 7.49 – 7.40 (m, 4H), 7.40 – 7.35 (m, 1H), 7.12 – 7.07 (m, 1H), 7.07 – 7.00 (m, 2H), 6.59 (s, 1H), 3.82 (s, 2H), 2.14 (s, 6H); ^{13}C NMR (126 MHz, $CDCl_3$) δ 169.4, 135.3,

135.1, 133.6, 129.5, 129.2, 128.1, 127.6, 127.3, 44.0, 18.2; HRMS (ESI) m/z calcd for $C_{16}H_{18}NO$ $[M+H]^+$: 240.1383; found: 240.1381.

53a: 2-(benzyloxy)-*N*-(3-(2,3-dihydrobenzo[*b*][*1,4*]dioxin-6-yl)-2-methylphenyl)acetamide

The product was obtained using general procedure **A** (X= Cl), 1 mmol scale 85 mg, 22% yield as

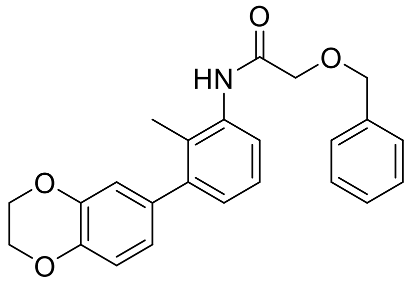

brown solid, M.P.= 167-169 °C; ^{1}H NMR (500 MHz, $CDCl_3$) δ 8.42 (s, 1H), 7.96 (dd, *J* = 8.2, 1.3 Hz, 1H), 7.47 – 7.31 (m, 5H), 7.26 (d, *J* = 7.8 Hz, 1H), 7.08 (dd, *J* = 7.5, 1.3 Hz, 1H), 6.92 (d, *J* = 8.2 Hz, 1H), 6.82 (d, *J* = 2.1 Hz, 1H), 6.77 (dd, *J* = 8.2, 2.1 Hz, 1H), 4.72 (s, 2H), 4.36 – 4.29 (m, 4H), 4.21 (s, 2H), 2.17 (s, 3H). ^{13}C NMR (126 MHz, $CDCl_3$) δ 167.6, 143.1, 142.7, 142.5, 136.7, 135.3, 135.1, 128.7, 128.4, 127.8, 126.9, 126.5, 126.1, 122.5, 121.4, 118.2, 116.9, 73.7, 69.9, 64.5, 64.4, 14.8. HRMS (ESI) m/z: $[M + H]^+$ calcd for $C_{24}H_{24}NO_4$, 390.1700; found, 390.1696.

54a: 4-methyl-*N*-((1*S*,4*R*)-1,7,7-trimethylbicyclo[*2.2.1*]heptan-2-yl)pent-3-enamide

The product was obtained using general procedure **A** (X= Br), 1 mmol scale 72 mg, 29% yield as

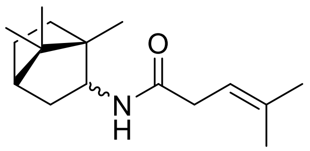

white solid, M.P.= 158-160 °C; product 54a contains two diastere-omers, dr = 3:1; ^{1}H NMR (500 MHz, $CDCl_3$) δ 5.88 (s, 1H), 5.35 – 5.27 (m, 1H), 3.90 – 3.85 (m, 1H), 3.03 – 2.89 (m, 2H), 1.85 – 1.82 (m, 1H), 1.81 (s, 3H), 1.75 (t, *J* = 4.4 Hz, 2H), 1.68 (s, 3H), 1.56 – 1.52 (m, 1H), 1.33 – 1.27 (m, 2H), 1.19 – 1.14 (m, 1H), 0.86 (s, 3H), 0.84 (s, 3H), 0.81 (s, 3H).^{13}C NMR (126 MHz, $CDCl_3$) δ 170.2, 138.4, 117.2, 56.4, 48.3, 47.1, 44.9, 39.3, 36.1, 27.0, 25.6, 20.2, 19.9, 17.9, 11.7. HRMS (ESI) m/z: $[M + H]^+$ calcd for $C_{16}H_{28}NO$, 250.2165; found, 250.2162.

55a: *N*-(2-ethylphenyl)-2-(4-(hydroxymethyl)phenyl)acetamide

The product was obtained using general procedure **A** (X= Cl), 1 mmol scale 43 mg, 16% yield as

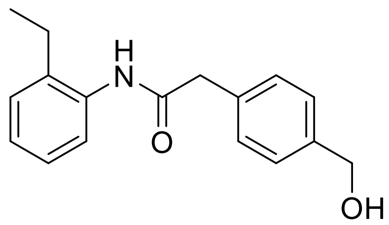

white solid, M.P.= 156-158 °C; ^{1}H NMR (500 MHz, $CDCl_3$) δ 7.88 (d, *J* = 8.2 Hz, 1H), 7.43 (d, *J* = 7.8 Hz, 2H), 7.37 (d, *J* = 7.8 Hz, 2H), 7.19 (t, *J* = 7.6 Hz, 1H), 7.12 – 7.02 (m, 2H), 6.95 (s, 1H), 4.74 (s, 2H), 3.79 (s, 2H), 2.24 (q, *J* = 7.7 Hz, 2H), 1.84(s, 1H), 0.92 (t, *J* = 7.6 Hz, 3H). ^{13}C NMR (126 MHz, $CDCl_3$) δ 169.0, 140.7, 134.8, 134.2, 133.9, 129.9, 128.7, 127.9, 126.8, 125.3, 122.7, 64.8, 44.6, 24.4, 13.9. HRMS (ESI) m/z: $[M + H]^+$ calcd for $C_{17}H_{20}NO_2$, 270.1489; found, 270.1487.

56a: 2-(5-chlorothiophen-2-yl)-*N*-octadecylacetamide

The product was obtained using general procedure **A** (X= Cl), 1 mmol scale 102 mg, 24% yield

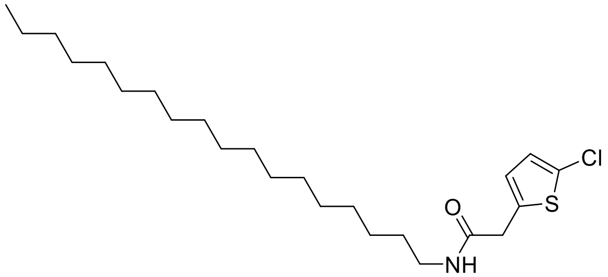

as yellow solid, M.P.= 144-146 °C; ^{1}H NMR (500 MHz, $CDCl_3$) δ 6.82 (d, *J* = 3.8 Hz, 1H), 6.73 (d, *J* = 3.8 Hz, 1H), 5.62 (s, 1H), 3.68 (s, 2H), 3.25 (q, *J* = 6.8 Hz, 2H), 1.51 – 1.43 (m, 2H), 1.28 – 1.23 (m, 30H), 0.90 (t, *J* = 6.8 Hz, 2H). ^{13}C NMR (126 MHz, $CDCl_3$) δ 169.0, 135.2, 129.6, 126.6, 126.2, 39.9, 38.0, 31.9, 29.7, 29.6, 29.5, 29.4, 29.2, 26.8, 22.7, 14.2. HRMS (ESI) m/z: $[M + H]^+$ calcd for $C_{24}H_{43}ClNOS$, 428.2748; found, 428.2746.

57a: *N*-(3,4-dichlorophenyl)butyramide

The product was obtained using general procedure **A** (X= I), 1 mmol scale 51 mg, 22% yield as oil; ^{1}H

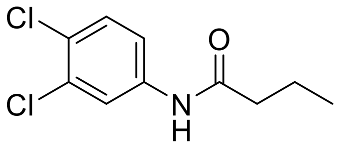

NMR (500 MHz, $CDCl_3$) δ 7.76 (d, *J* = 2.1 Hz, 1H), 7.45 (s, 1H), 7.38 – 7.31 (m, 2H), 2.32 (t, *J* = 7.4 Hz, 2H), 1.80 – 1.68 (m, 2H), 0.99 (t, *J* = 7.4 Hz, 3H); ^{13}C NMR (126 MHz, $CDCl_3$) δ 171.4, 137.3, 132.7, 130.4, 127.3, 121.5, 118.9, 39.5, 18.9, 13.7; HRMS (ESI) m/z calcd for $C_{10}H_{12}Cl_2NO$ $[M+H]^+$: 232.090; found: 232.0291.

58a: *N*-(9*H*-fluoren-9-yl)-4-phenoxybutanamide

The product was obtained using general procedure **A** (X= Br), 1 mmol scale 220 mg, 64% yield as semisolid; ^{1}H NMR (500 MHz, $CDCl_3$) δ 7.67 (d, *J* = 7.6 Hz, 2H), 7.63 (d, *J* = 7.6 Hz, 2H), 7.47 – 7.42 (m, 2H), 7.40 – 7.35 (m, 2H), 7.25 – 7.19 (m, 2H), 6.91 (td, *J* = 7.4, 1.2 Hz, 1H), 6.76 (d, *J* = 8.5 Hz, 2H), 3.77 (d, *J* = 6.3 Hz, 2H), 2.50 – 2.43 (m, 2H), 1.61 – 1.55 (m, 2H); ^{13}C NMR (126 MHz, $CDCl_3$) δ 158.5, 156.3, 143.4, 139.4, 129.7, 129.3, 128.4, 123.5, 120.7, 120.3, 114.2, 66.6, 38.2, 23.7; HRMS (ESI) m/z calcd for $C_{23}H_{22}NO_2$ $[M+H]^+$: 343.1645; found: 343.1645.

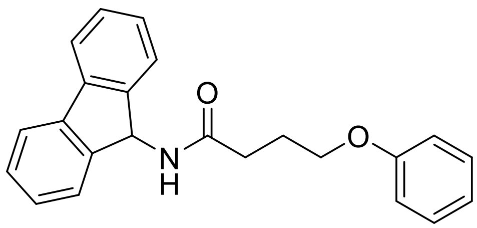

59a: *N*-(5-methoxy-1,2,3,4-tetrahydronaphthalen-1-yl)butyramide

The product was obtained using general procedure **A** (X= I), 1 mmol scale 20 mg, 8% yield as semisolid; ^{1}H NMR (500 MHz, $CDCl_3$) δ 9.21 (s, 1H), 7.25 – 7.19 (m, 2H), 6.96 (dd, *J* = 7.5, 1.8 Hz, 1H), 3.87 (m, 4H), 3.49 (q, *J* = 6.7 Hz, 2H), 2.77 – 2.68 (m, 2H), 2.35 – 2.26 (m, 2H), 1.70 – 1.62 (m, 2H), 1.59 – 1.55 (m, 2H), 0.96 (t, *J* = 7.4 Hz, 3H); ^{13}C NMR (126 MHz, $CDCl_3$) δ 186.6, 159.6, 130.5, 126.0, 120.4, 112.5, 60.4, 55.7, 48.9, 24.5, 23.4, 22.5, 11.3; HRMS (ESI) m/z calcd for $C_{15}H_{22}NO_2$ $[M+H]^+$: 248.1645; found: 248.1646.

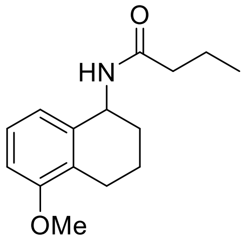

60a: (*Z*)-4,8-dimethyl-*N*-octadecylnona-3,7-dienamide

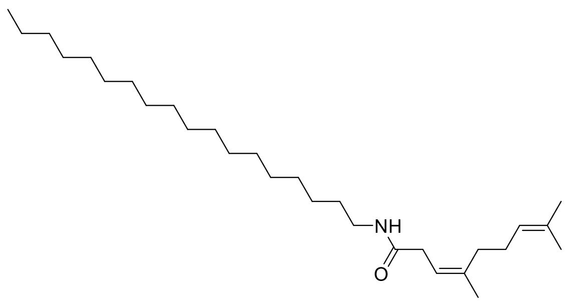

The product was obtained using general procedure **A** (X= Cl), 1 mmol scale 112 mg, 26% yield as yellow solid, M.P.= 139-141 °C; ^{1}H NMR (500 MHz, $CDCl_3$) δ 5.77 (s, 1H), 5.32 (t, *J* = 7.6 Hz, 1H), 5.10 (t, *J* = 6.6 Hz, 1H), 3.25 – 3.21 (m, 2H), 2.98 (d, *J* = 7.6 Hz, 2H), 2.28 – 2.05 (m, 4H), 1.72 (s, 3H), 1.66 (s, 3H), 1.64 (s, 3H), 1.37 – 1.18 (m, 30H), 0.91 – 0.88 (m, 5H). ^{13}C NMR (126 MHz, $CDCl_3$) δ 171.1, 141.5, 132.1, 123.7, 116.9, 39.6, 39.6, 35.9, 31.9, 29.7, 29.6, 29.5, 29.4, 29.3, 26.9, 26.4, 25.7, 22.7, 17.8, 16.1, 14.1. HRMS (ESI) m/z: $[M + H]^+$ calcd for $C_{29}H_{56}NO$, 434.4356; found, 434.4350.

61a: 2-(2,6-dichlorophenyl)-*N*-octadecylacetamide

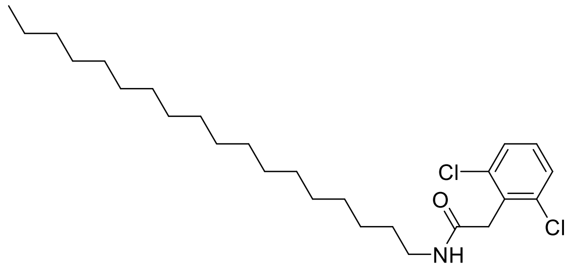

The product was obtained using general procedure **A** (X= Br), 1 mmol scale 141 mg, 31% yield as yellow solid, M.P.= 136-138 °C; ^{1}H NMR (500 MHz, $CDCl_3$) δ 7.38 (dd, *J* = 8.1, 1.8 Hz, 1H), 7.23 – 7.20 (m, 1H), 5.36 (s, 1H), 3.95 (s, 2H), 3.25 (d, *J* = 6.7 Hz, 2H), 1.37 – 1.17 (m, 30H), 0.92 – 0.88 (m, 5H). ^{13}C NMR (126 MHz, $CDCl_3$) δ 167.9, 136.3, 131.8, 129.1, 128.4, 39.8, 39.1, 31.9, 29.7, 29.6, 29.5, 29.4, 29.2, 26.8, 22.7, 14.1. HRMS (ESI) m/z: $[M + H]^+$ calcd for $C_{26}H_{44}Cl_2NO$, 456.2794; found, 456.2792.

1b: *N*-(1-(4-(2-(((3s,5s,7s)-adamantan-1-yl)amino)-2-oxoethyl)thiophen-2-yl)-2-(tert-butylamino)-2-oxoethyl)-*N*-phenylbut-2-ynamide

The product was obtained using procedure **B**, 1 mmol scale 392 mg, 72% yield as yellow solid,

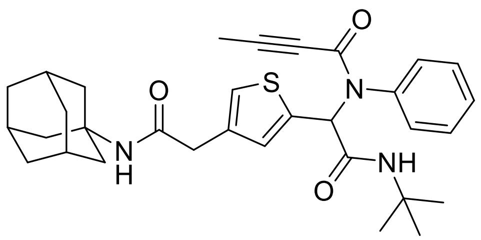

M.P.= 139-141 °C; ^{1}H NMR (500 MHz, $CDCl_3$) δ 7.28 – 7.20 (m, 3H), 7.19 – 7.09 (m, 2H), 7.00 (d, *J* = 1.4 Hz, 1H), 6.90 (d, *J* = 1.5 Hz, 1H), 6.24 (s, 1H), 6.13 (s, 1H), 5.18 (s, 1H), 3.34 (s, 2H), 2.09 – 2.04 (m, 3H), 2.04 (s, 1H), 1.95 – 1.91 (m, 6H), 1.68 (s, 3H), 1.66 – 1.61 (m, 6H), 1.37 (s, 9H). ^{13}C NMR (126 MHz, $CDCl_3$) δ 169.3, 167.0, 154.7, 139.3, 136.5, 134.5, 130.9, 129.9, 128.6, 128.5, 125.6, 91.9, 73.7, 60.2, 51.9, 51.8, 41.5, 39.3, 36.3, 29.4, 28.6, 3.9. HRMS (ESI) m/z: $[M + H]^+$ calcd for $C_{32}H_{40}N_3O_3S$, 546.2785; found, 546.2779.

1c: *N*-((3s,5s,7s)-adamantan-1-yl)-2-(5-(3-(cyclohexylamino)imidazo[*1,2-a*]pyridin-2-yl)thiophen-3-yl)acetamide

The product was obtained using procedure **C**, 1 mmol scale 175 mg, 36% yield as yellow solid,

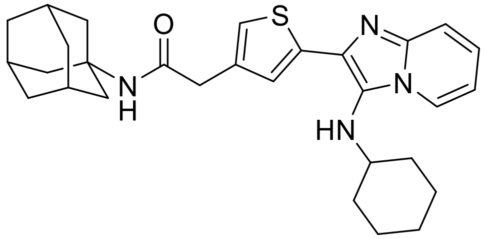

M.P.= 141-143 °C; ^{1}H NMR (500 MHz, $CDCl_3$) δ 8.08 (d, *J* = 6.8 Hz, 1H), 7.51 (d, *J* = 9.2 Hz, 1H), 7.49 (s, 1H), 7.29 (s, 1H), 7.14 (dd, *J* = 9.0, 6.7 Hz, 1H), 7.10 (s, 1H), 6.79 (t, *J* = 6.7 Hz, 1H), 5.32 (s, 1H), 3.53 (s, 2H), 3.21 – 2.95 (m, 1H), 2.12 – 2.00 (m, 3H), 1.98 – 1.94 (m, 6H), 1.92 – 1.85 (m, 2H), 1.80 – 1.73 (m, 2H), 1.70 – 1.57 (m, 8H), 1.40 – 1.30 (m, 2H), 1.21 –

1.16 (m, 2H). ^{13}C NMR (126 MHz, $CDCl_3$) δ 169.6, 141.7, 138.3, 136.1, 132.4, 125.2, 124.4, 124.1, 122.8, 122.4, 117.2, 111.8, 57.0, 51.9, 41.5, 39.7, 36.3, 34.3, 29.4, 25.7, 24.9. HRMS (ESI) m/z: [M + H]+ calcd for $C_{29}H_{37}N_4OS$, 489.2683; found, 489.2678.

1d: *N*-((3s,5s,7s)-adamantan-1-yl)-2-(5-((1-(2-methoxybenzyl)-1*H*-tetrazol-5-yl)(phenylamino)methyl)thiophen-3-yl)acetamide

The product was obtained using procedure **D**, 1 mmol scale 221 mg, 39% yield as yellow solid,

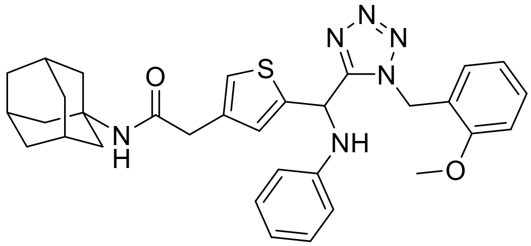

M.P.= 147-149 °C; ^{1}H NMR (500 MHz, $CDCl_3$) δ 7.35 (dd, *J* = 7.8, 1.6 Hz, 1H), 7.18 – 7.09 (m, 3H), 7.05 (s, 1H), 6.95 (t, *J* = 7.5 Hz, 1H), 6.90 (d, *J* = 8.3 Hz, 1H), 6.79 – 6.74 (m, 1H), 6.70 (d, *J* = 1.2 Hz, 1H), 6.55 (d, *J* = 8.0 Hz, 2H), 6.24 (d, *J* = 8.4 Hz, 1H), 5.57 (s, 2H), 5.19 (s, 1H), 4.95 (d, *J* = 8.4 Hz, 1H), 3.78 (s, 3H), 3.32 (s, 2H), 2.08 – 2.04 (m, 3H), 1.91 – 1.87 (m, 6H), 1.66 – 1.60 (m, 6H). ^{13}C NMR (126 MHz, $CDCl_3$) δ 169.0, 156.7, 154.9, 145.1, 141.7, 135.6, 130.8, 130.3, 129.4, 127.5, 123.6, 121.2, 121.1, 119.4, 114.0, 110.9, 55.5, 52.0, 48.8, 46.4, 41.4, 39.4, 36.3, 29.4. HRMS (ESI) m/z: $[M + H]^+$ calcd for $C_{32}H_{37}N_6O_2S$, 569.2693; found, 569.2684.

62a: Methyl (R)-2-(((3S,5S,7S)-adamantan-1-yl)carbamoyl)butanoate

This product was obtained using general procedure **A** (X = Br), 1mmol scale 60mg, 22% yield as

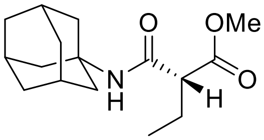

white solid, M.P.= 161-163 °C; ^{1}H NMR (500 MHz, $CDCl_3$) δ 6.20 (s, 1H), 3.75 (s, 3H), 3.10 – 2.93 (m, 1H), 2.14 – 2.06 (m, 3H), 2.03 – 2.01 (m, 6H), 1.94 – 1.89 (m, 2H), 1.70 – 1.66 (m, 6H), 0.98 – 0.95 (m, 3H). ^{13}C NMR (126 MHz, $CDCl_3$) δ 173.0, 167.0, 55.3, 52.3, 52.0, 41.4, 36.3, 29.4, 24.6, 11.7; HRMS (ESI) m/z: $[M + H]^+$ calcd for $C_{16}H_{26}NO_3$, 280.1907; found, 280.1904.

15. Supporting Information Spectral data:

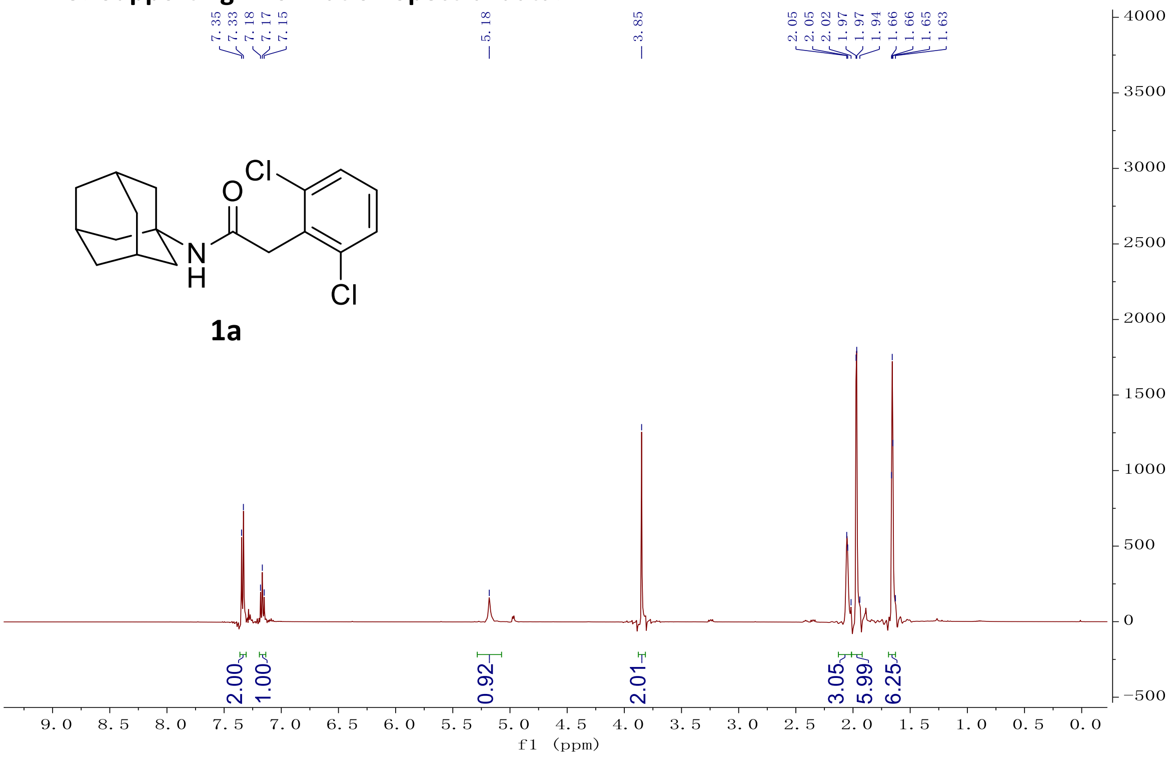

Supplementary Figure 16. ^{1}H NMR (400 MHz, $CDCl_3$) of compound **1a**

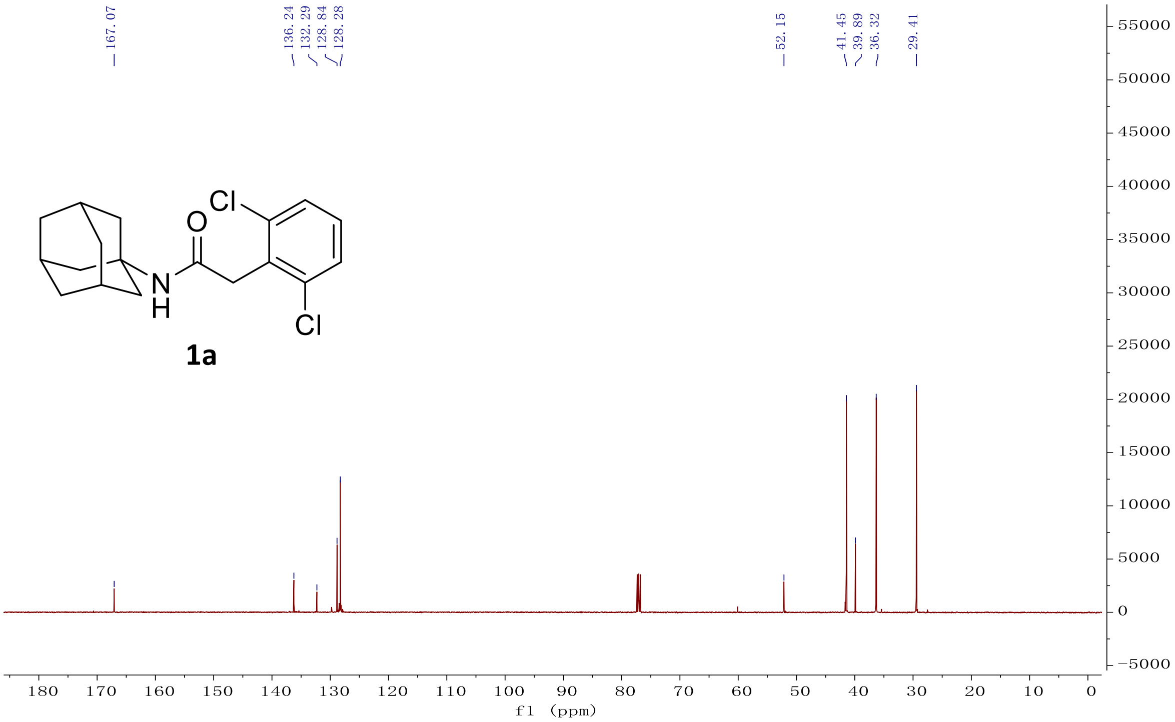

Supplementary Figure 17. ^{13}C NMR (126 MHz, $CDCl_3$) of compound **1a**

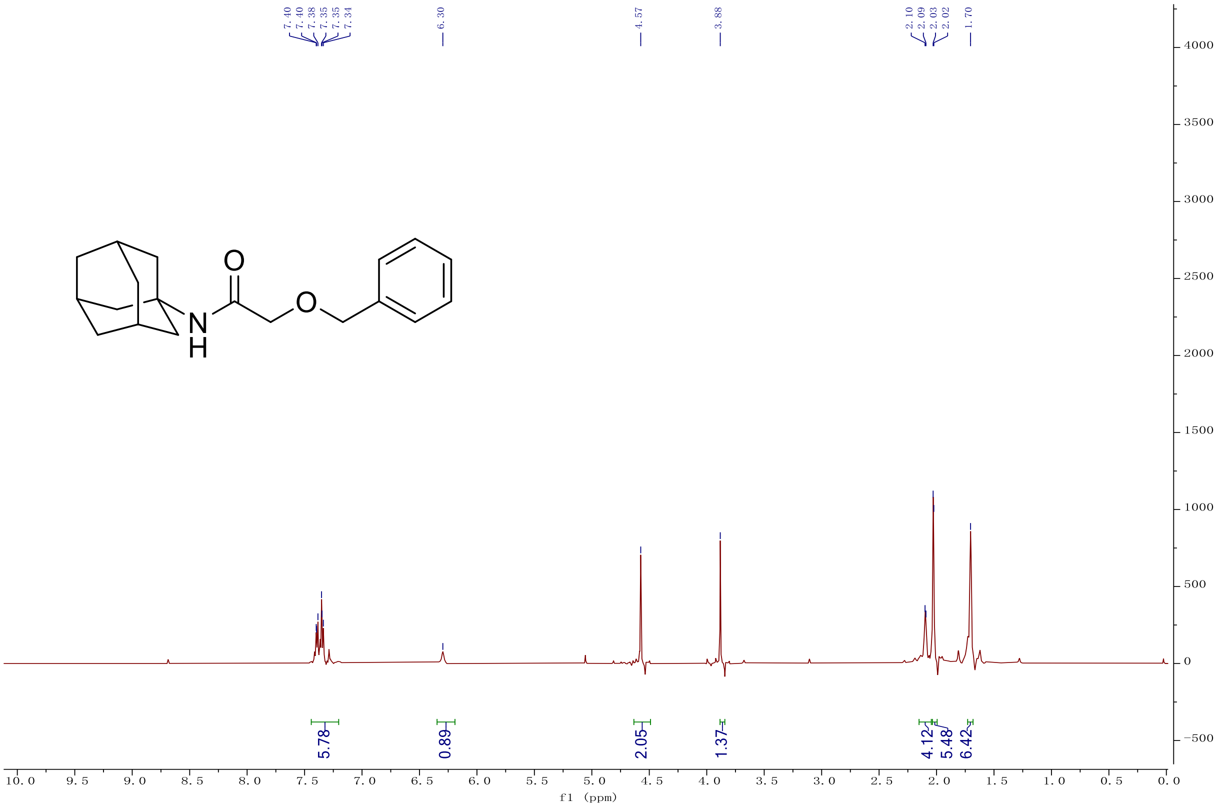

Supplementary Figure 18. ^{1}H NMR (400 MHz, $CDCl_3$) of compound **2a**

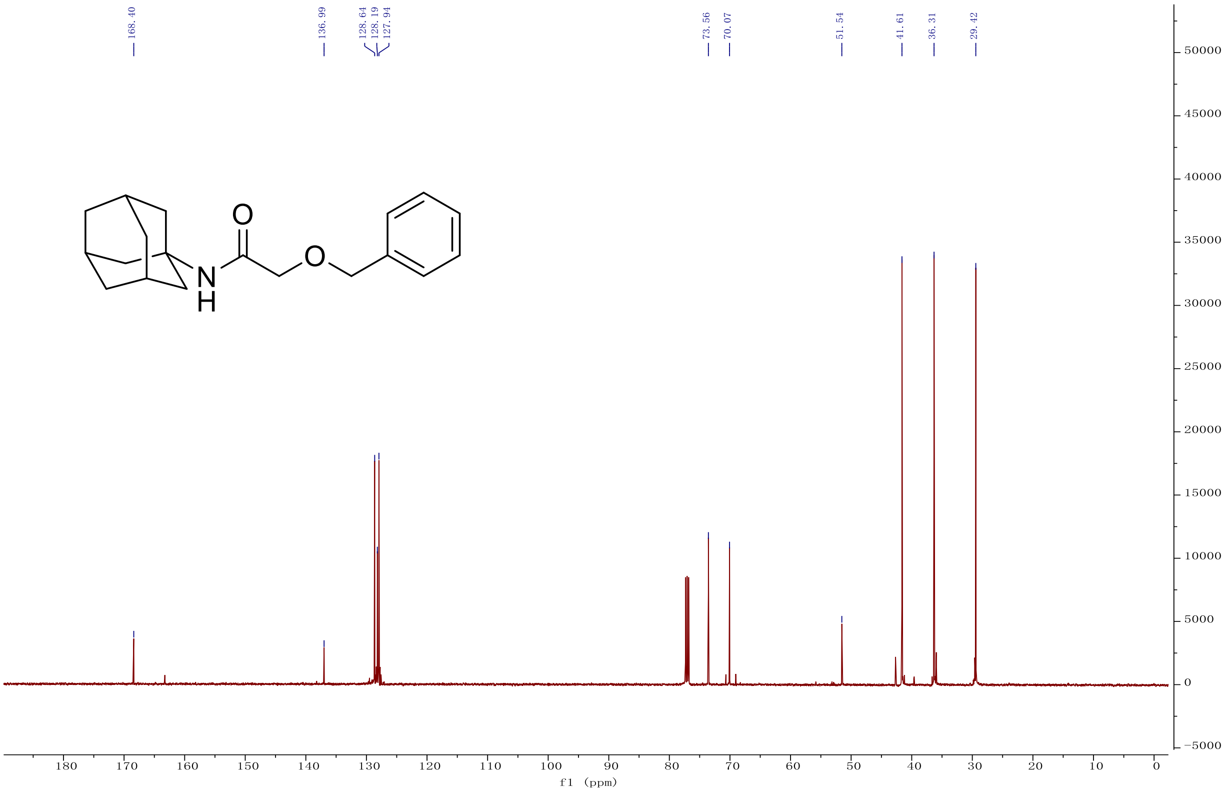

Supplementary Figure 19. ^{13}C NMR (126 MHz, $CDCl_3$) of compound **1a**

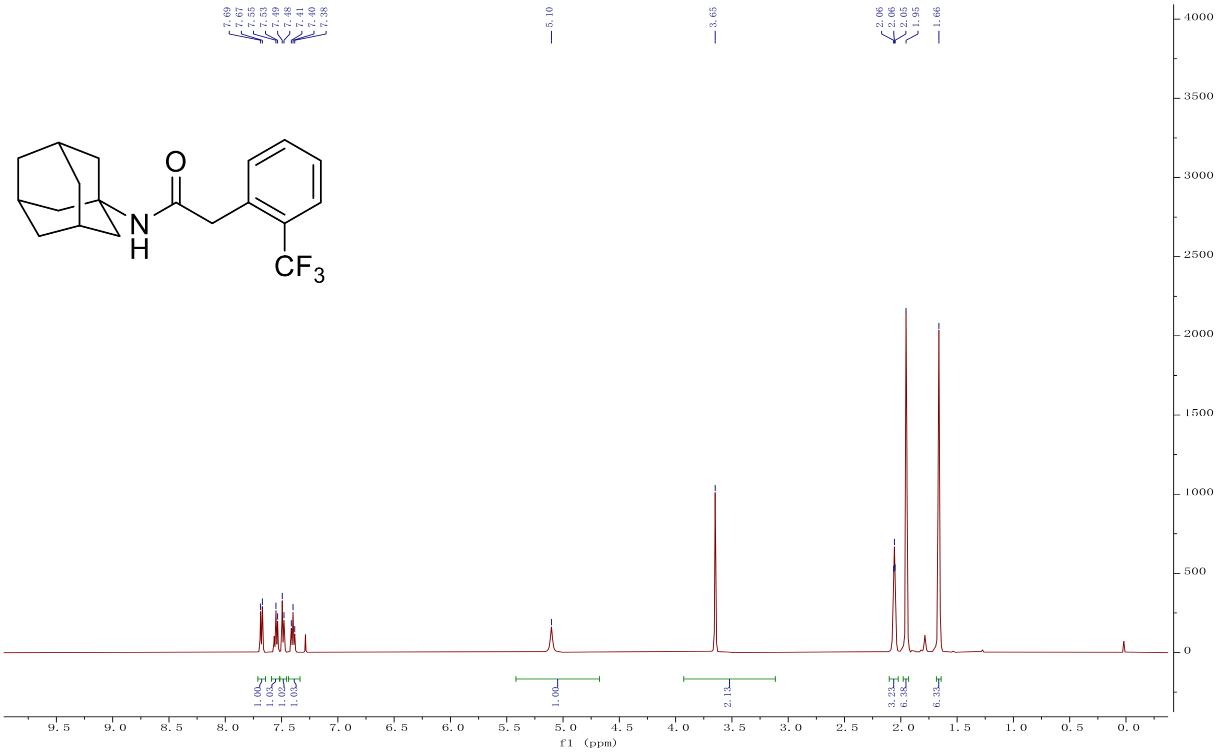

Supplementary Figure 20. ^{1}H NMR (400 MHz, $CDCl_3$) of compound **3a**

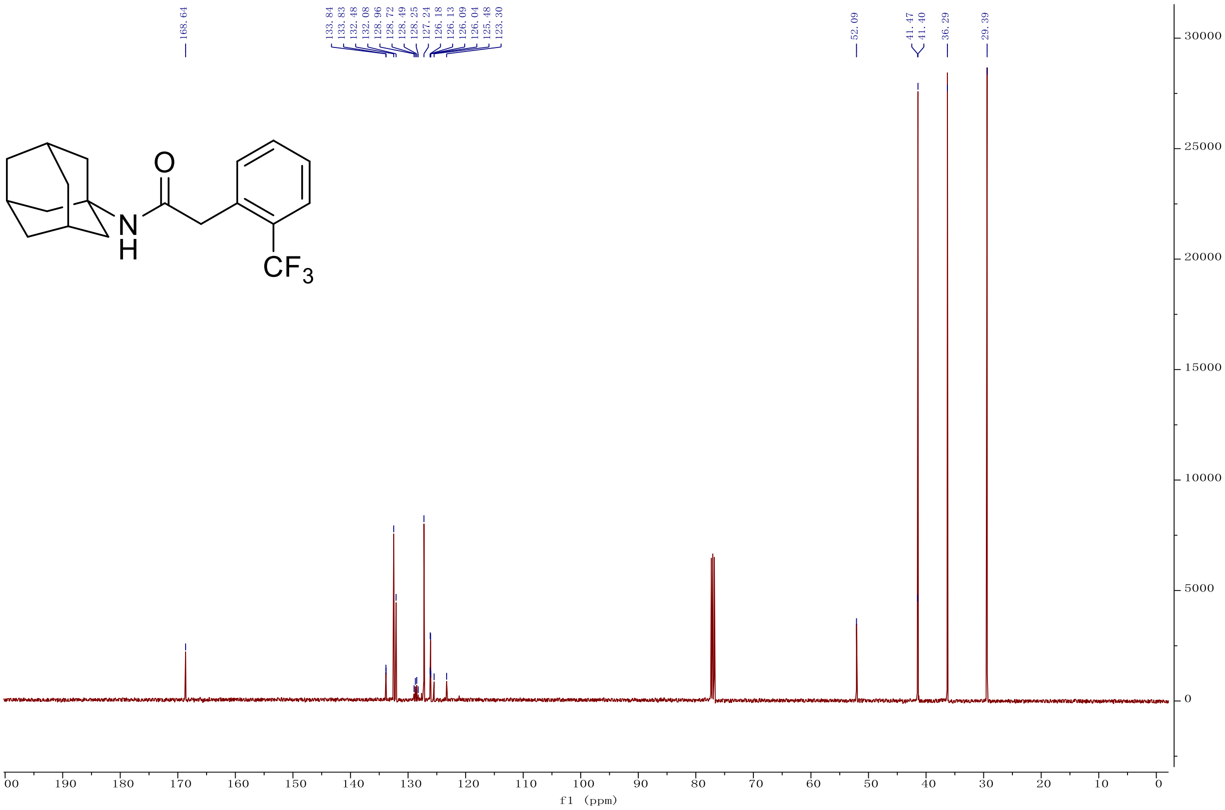

Supplementary Figure 21. ^{13}C NMR (126 MHz, $CDCl_3$) of compound **3a**

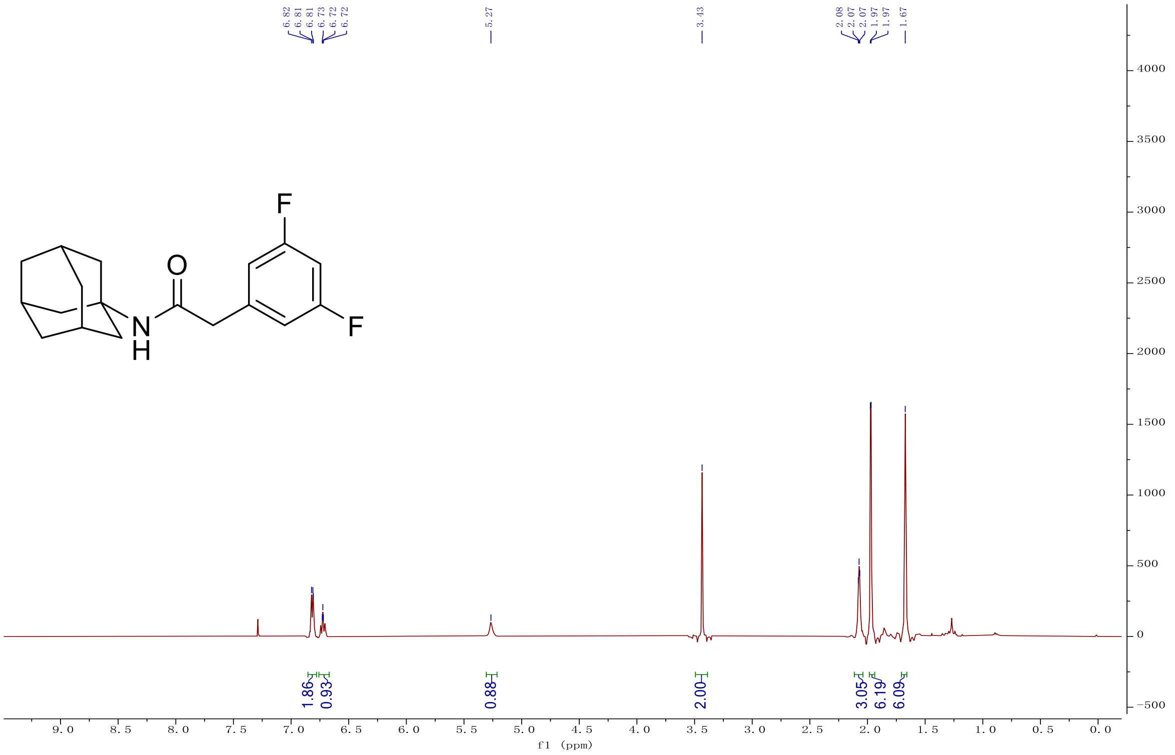

Supplementary Figure 22. ^{1}H NMR (400 MHz, $CDCl_3$) of compound **4a**

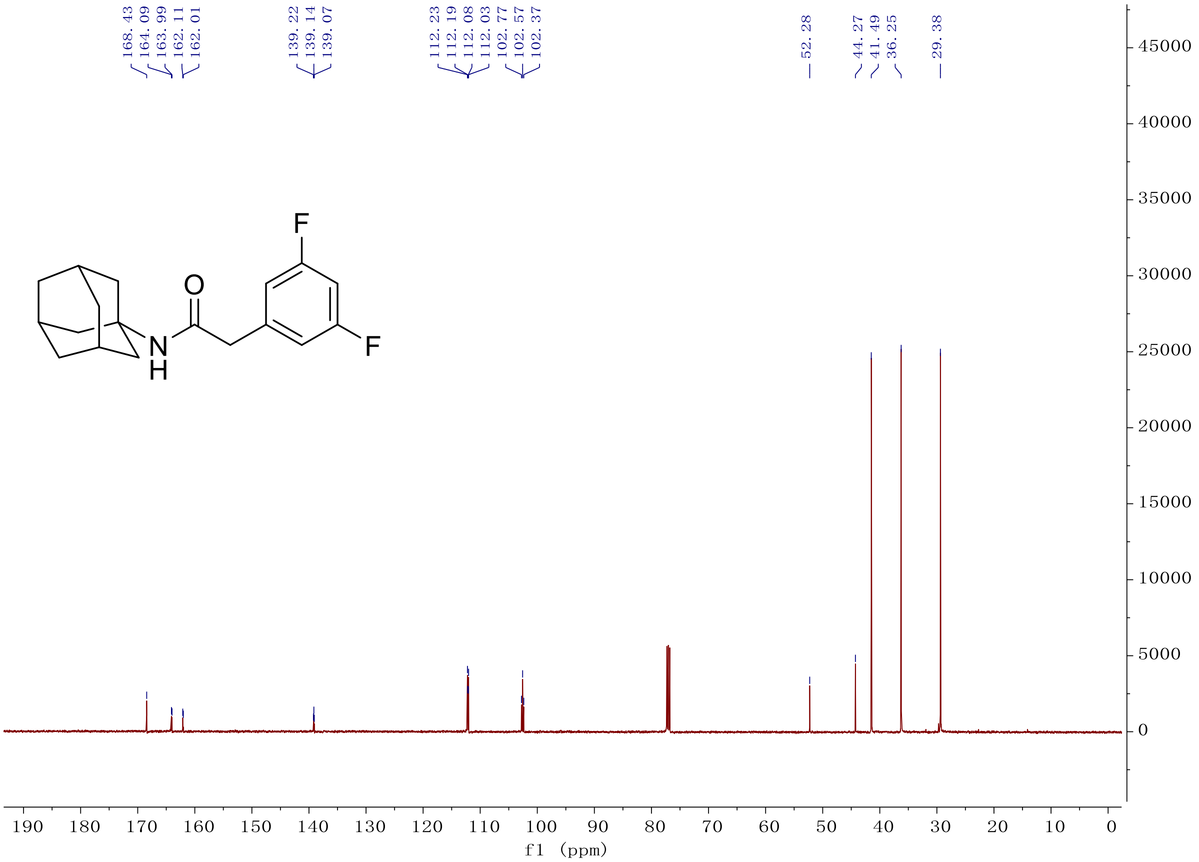

Supplementary Figure 23. ^{13}C NMR (126 MHz, $CDCl_3$) of compound **4a**

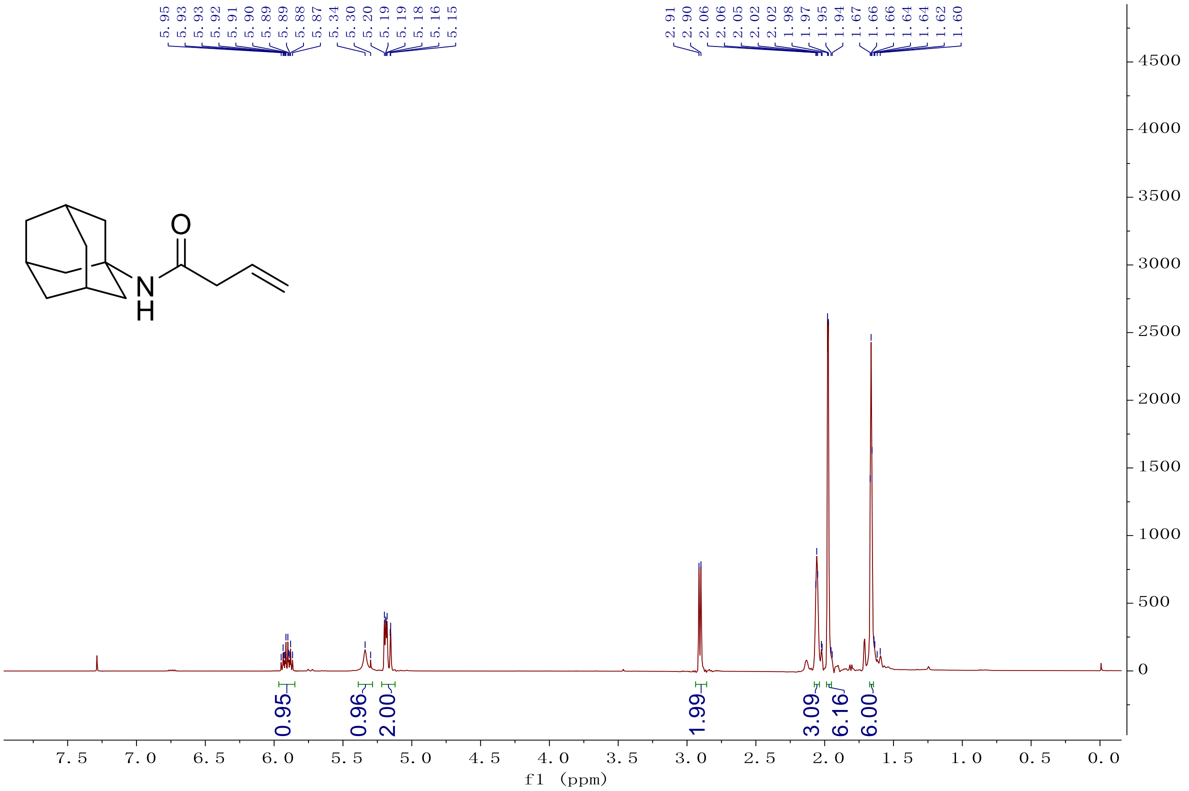

Supplementary Figure 24. ^{1}H NMR (400 MHz, $CDCl_3$) of compound **5a**

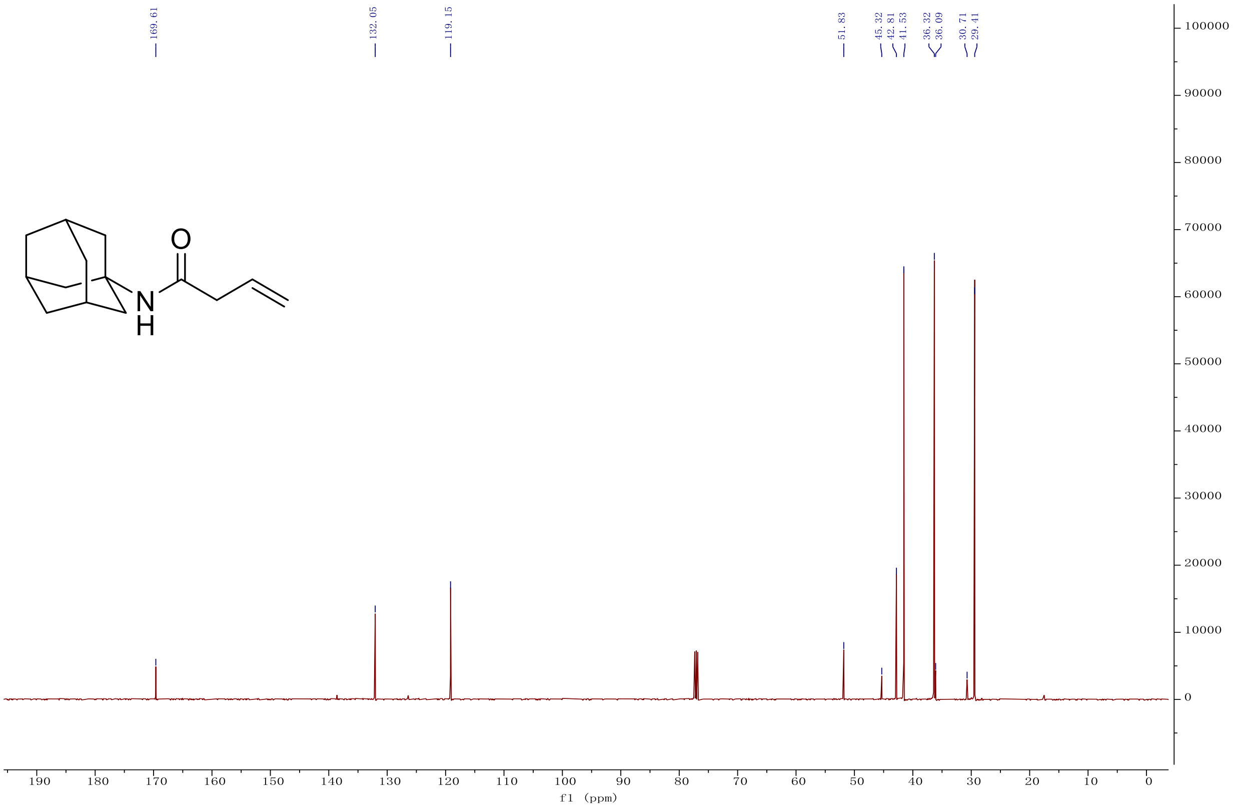

Supplementary Figure 25. ^{13}C NMR (126 MHz, $CDCl_3$) of compound **5a**

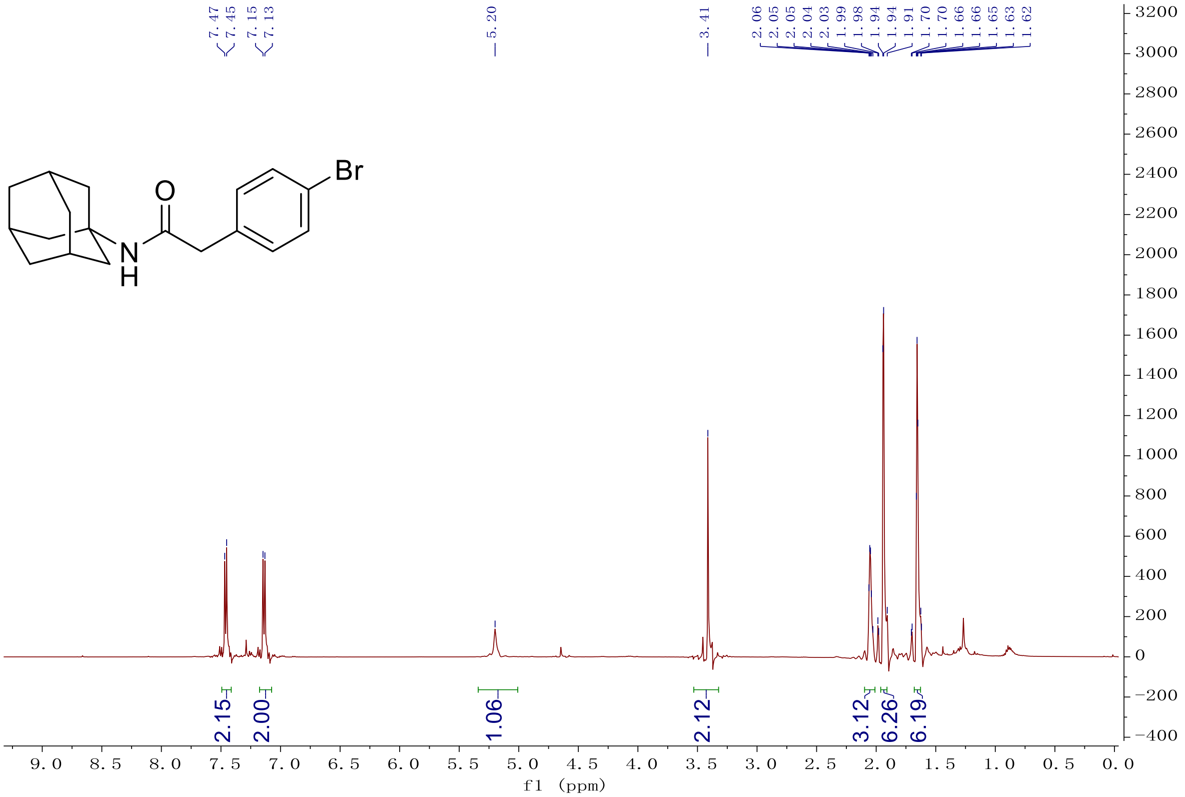

Supplementary Figure 26. ^{1}H NMR (400 MHz, $CDCl_3$) of compound **6a**

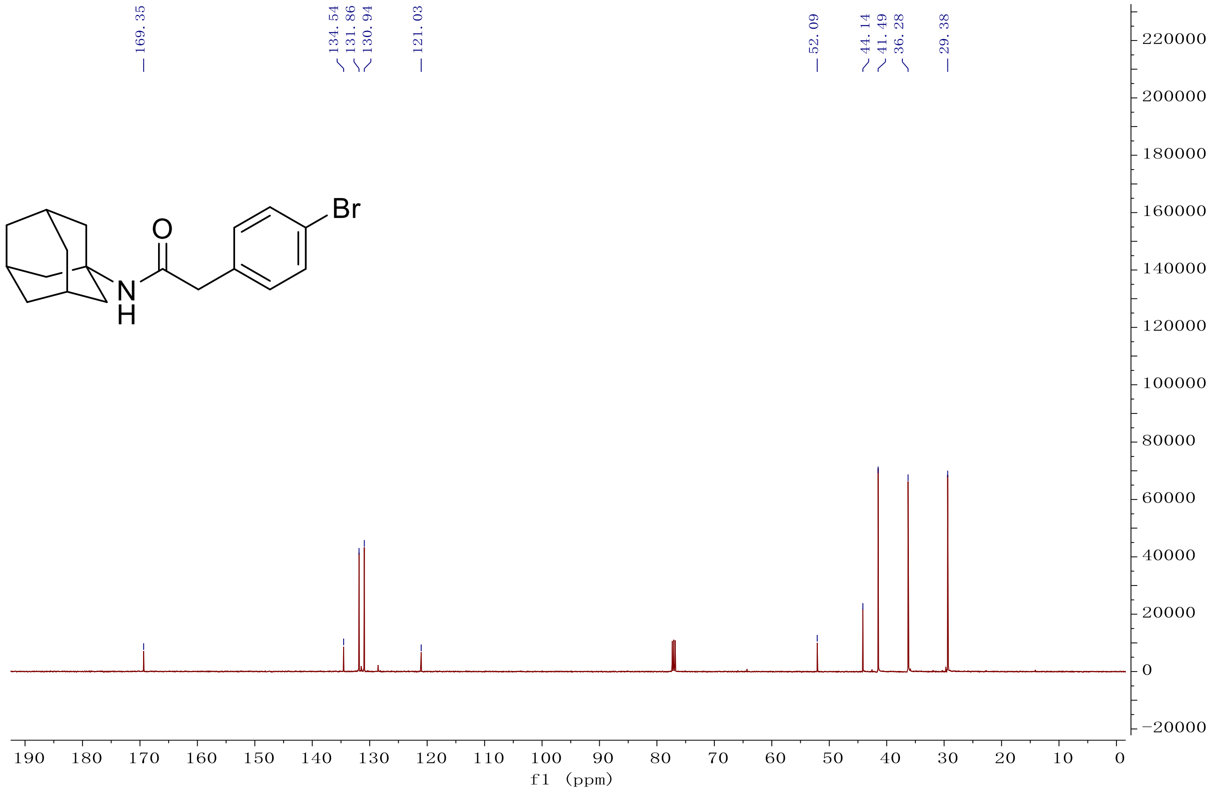

Supplementary Figure 27. ^{13}C NMR (126 MHz, $CDCl_3$) of compound **6a**

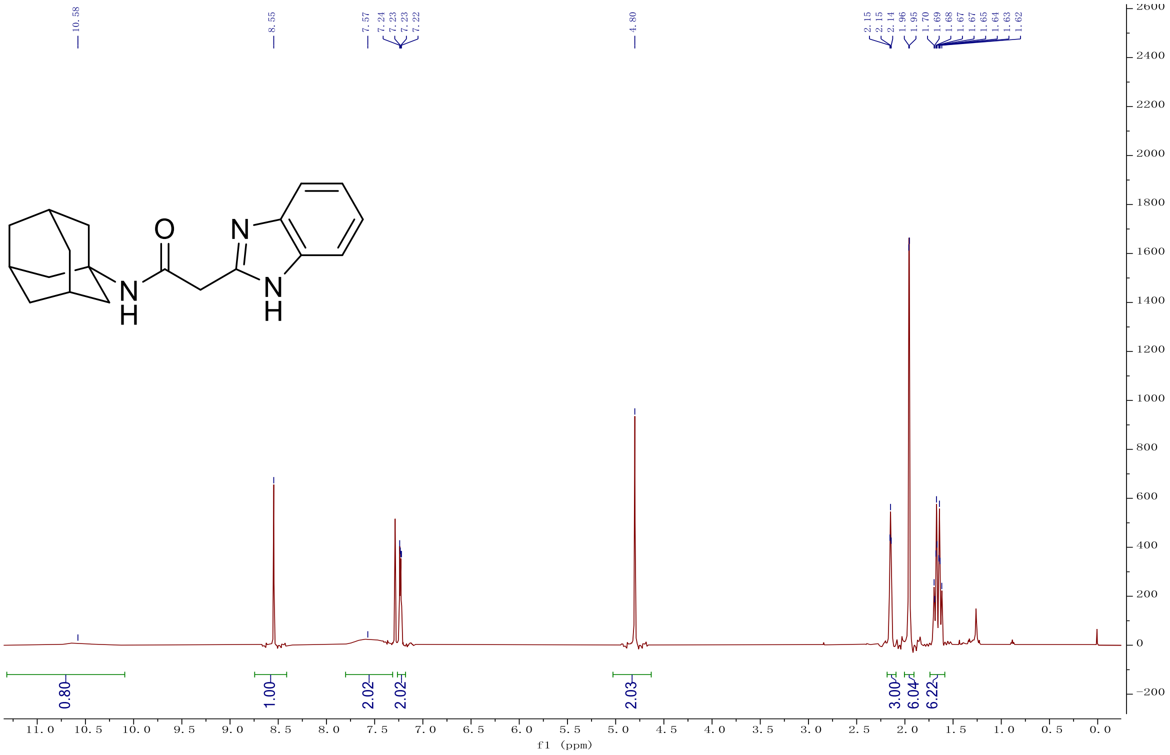

Supplementary Figure 28. ^{1}H NMR (400 MHz, $CDCl_3$) of compound **7a**

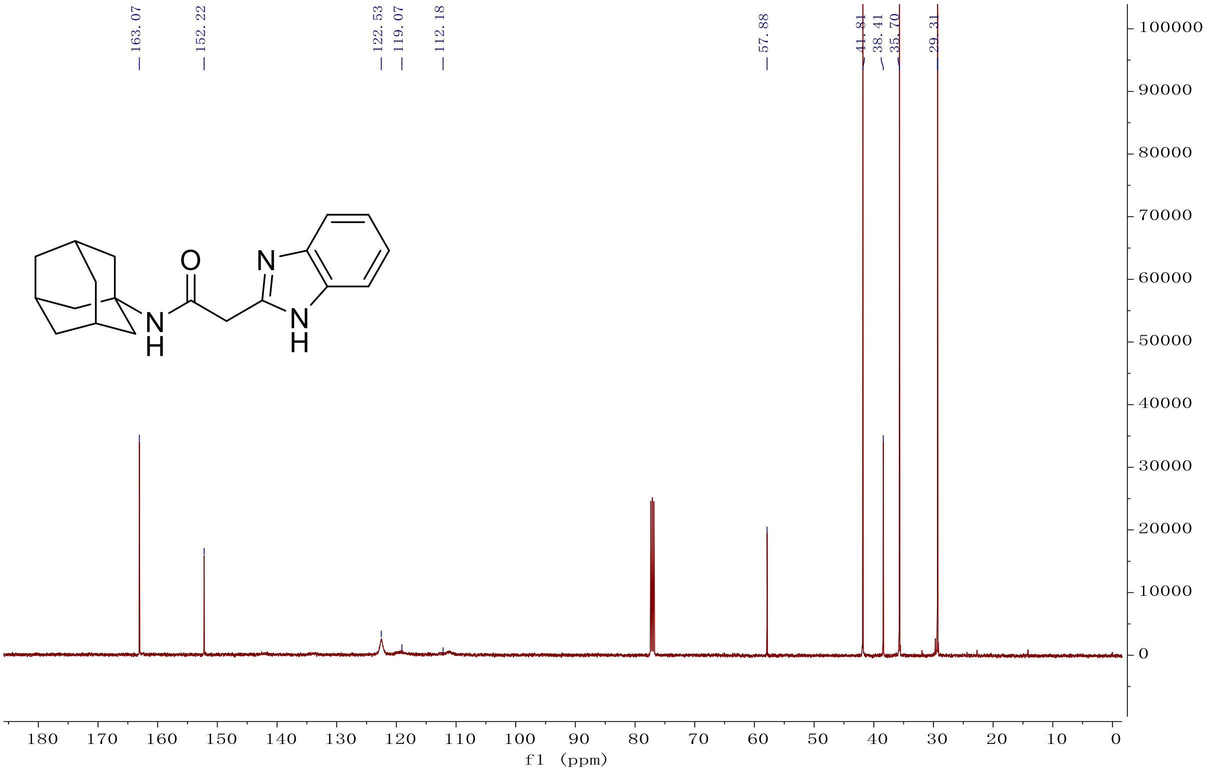

Supplementary Figure 29. ^{13}C NMR (126 MHz, $CDCl_3$) of compound **7a**

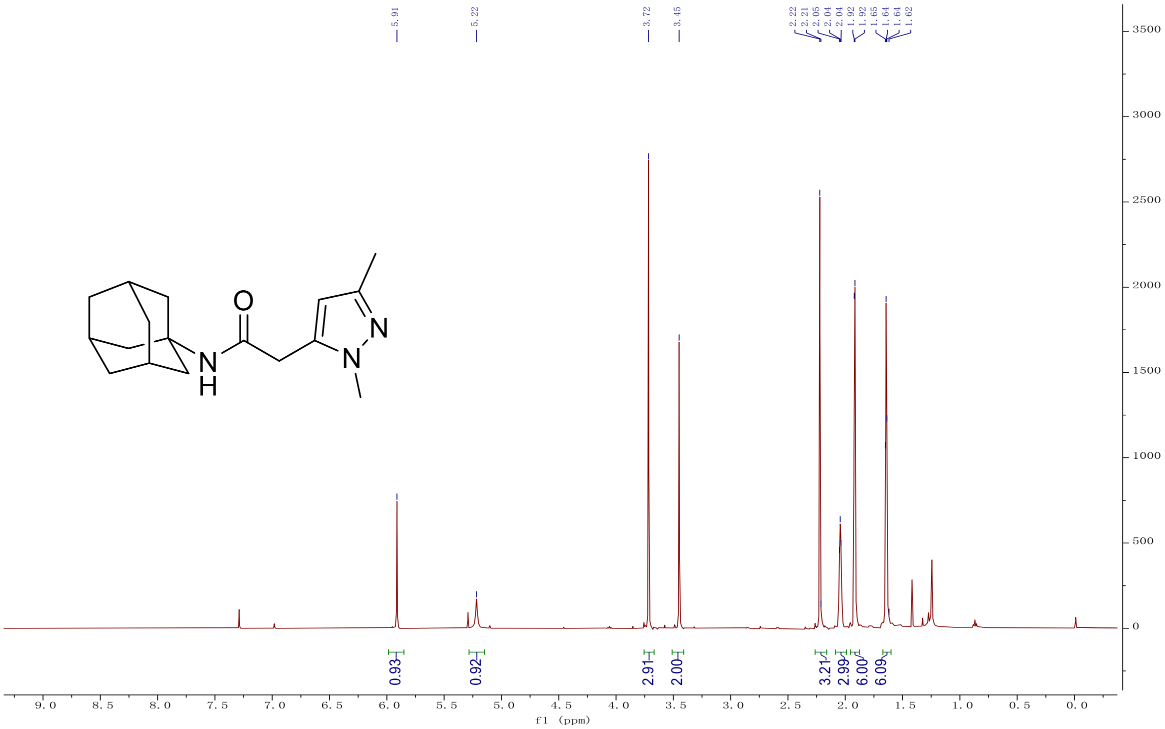

Supplementary Figure 30. ^{1}H NMR (400 MHz, $CDCl_3$) of compound **8a**

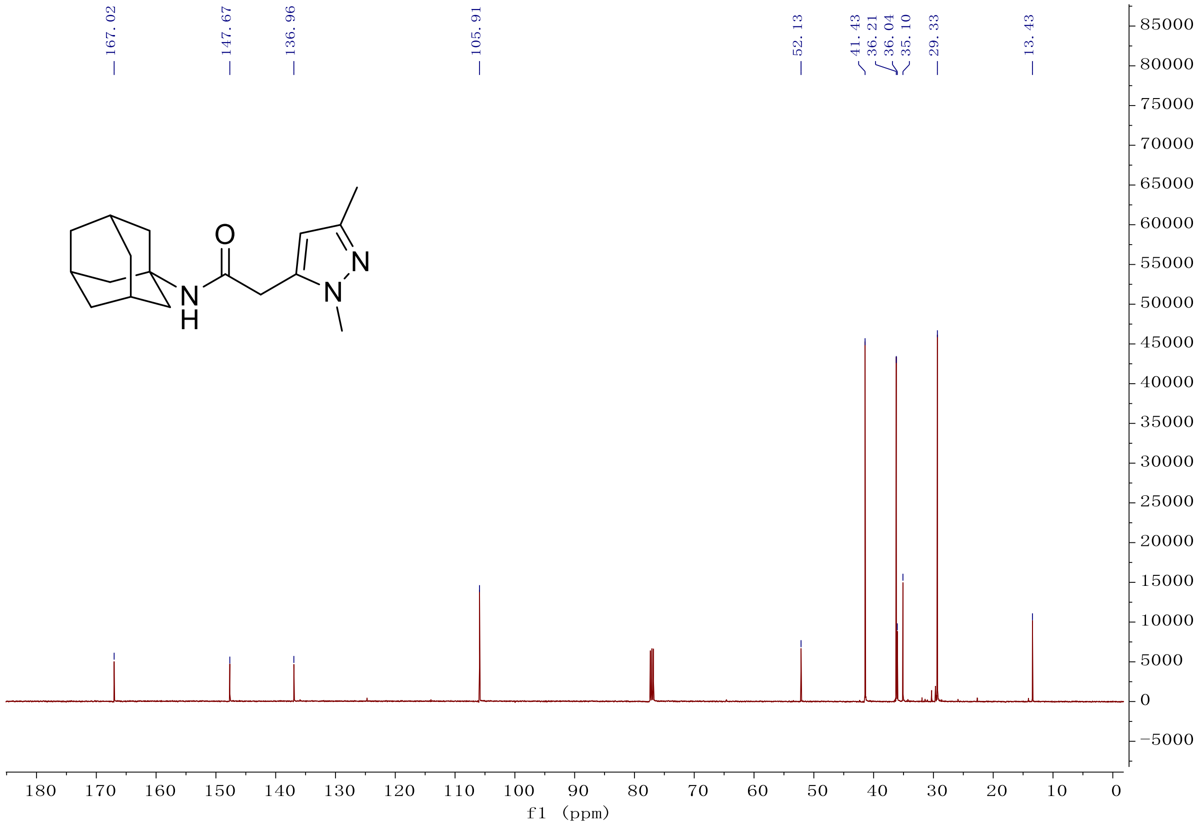

Supplementary Figure 31. ^{13}C NMR (126 MHz, $CDCl_3$) of compound **8a**

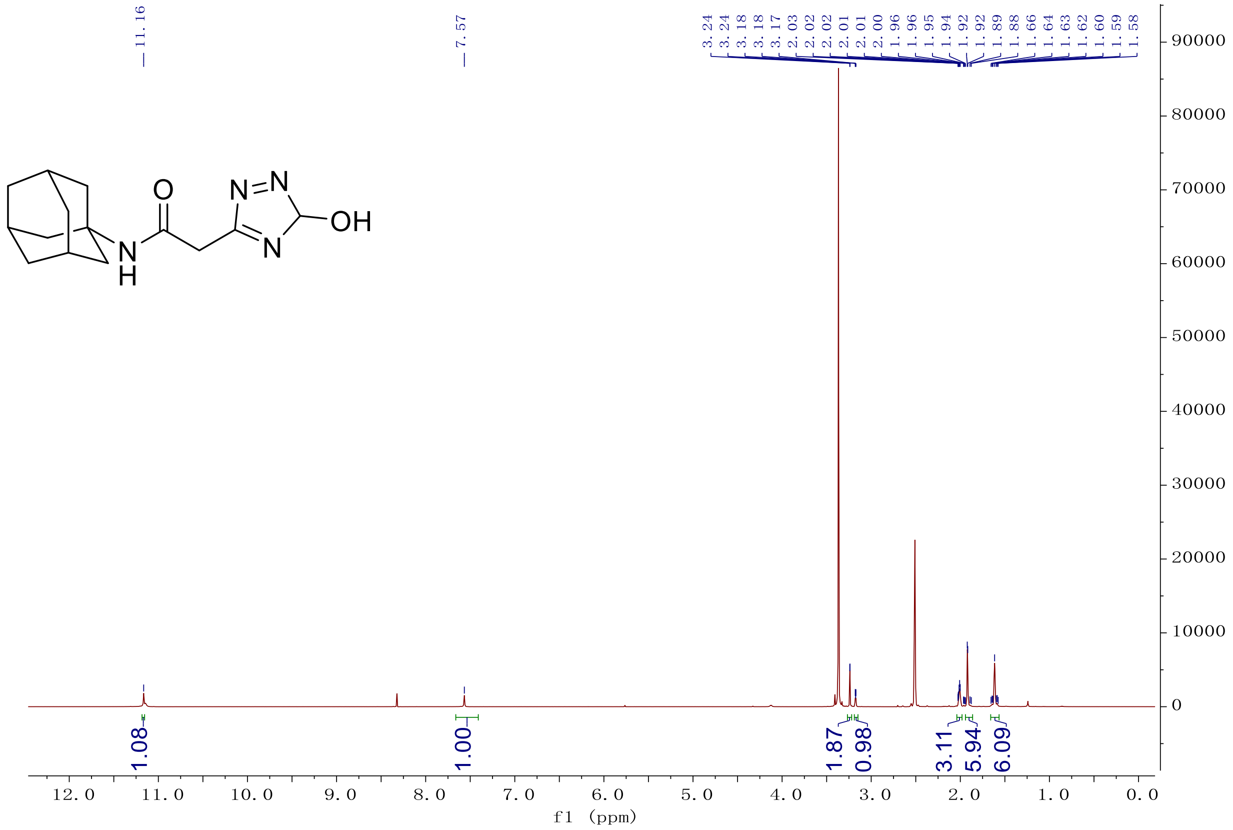

Supplementary Figure 32. ^{1}H NMR (400 MHz, DMSO-*d*6) of compound **9a**

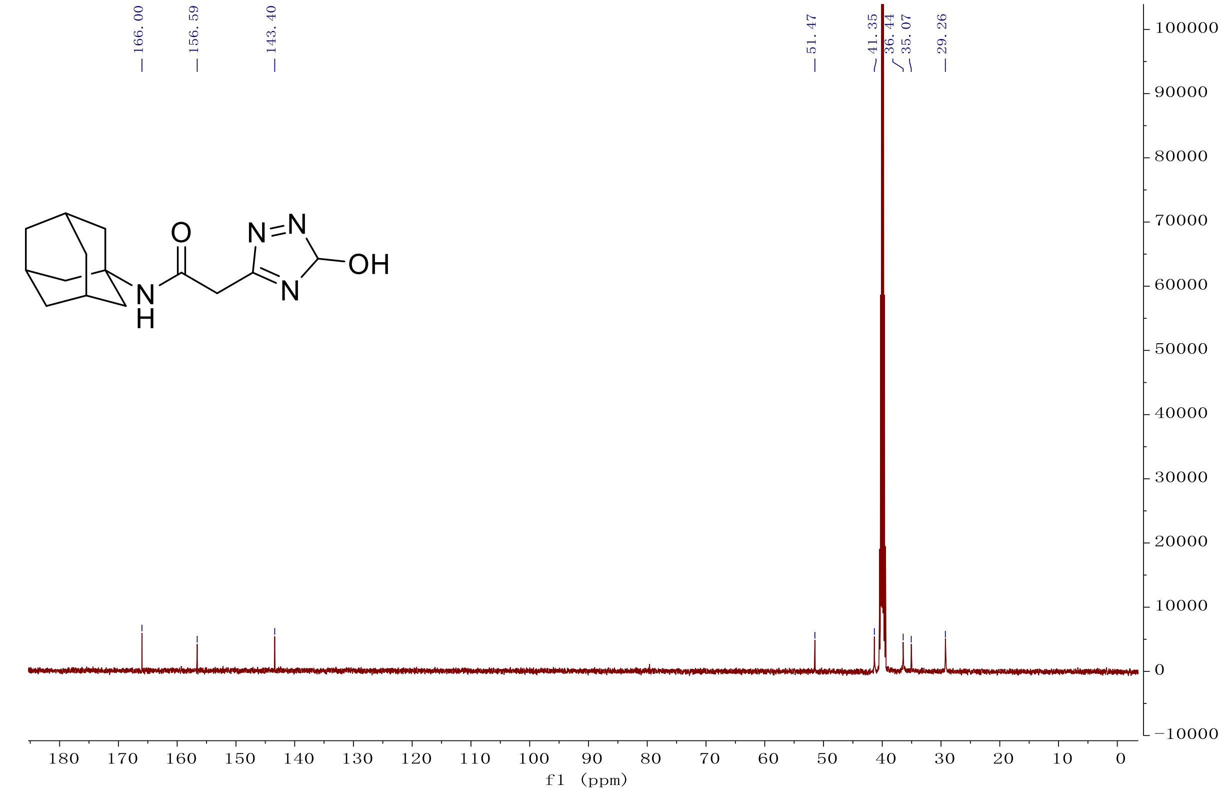

Supplementary Figure 33. ^{13}C NMR (126 MHz, DMSO-*d*6) of compound **9a**

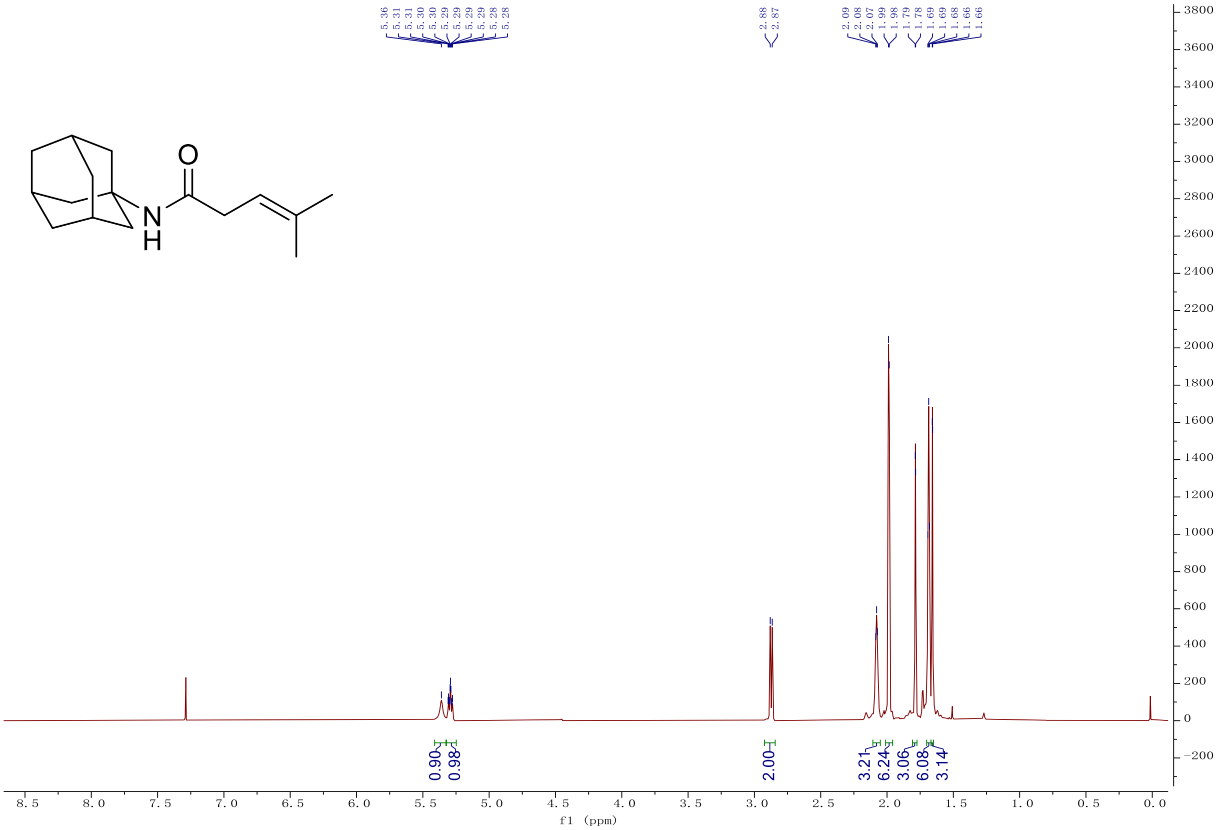

Supplementary Figure 34. ^{1}H NMR (400 MHz, $CDCl_3$) of compound **10a**

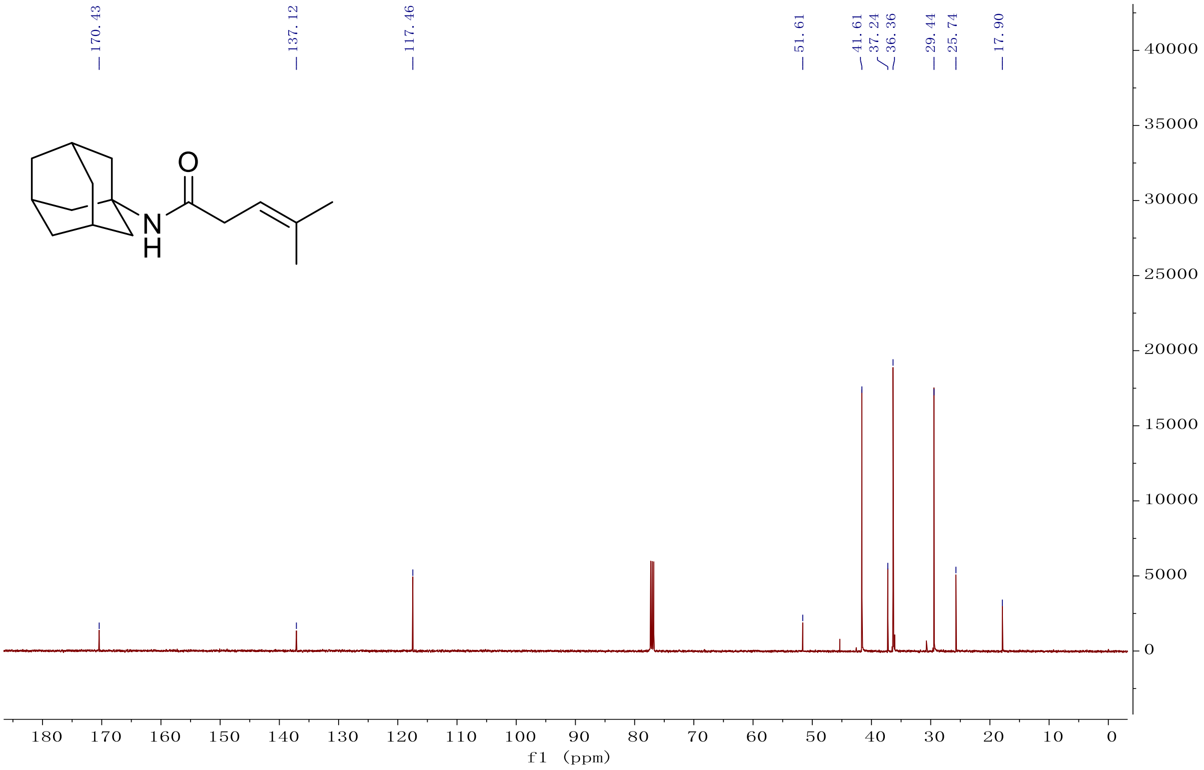

Supplementary Figure 35. ^{13}C NMR (126 MHz, $CDCl_3$) of compound **10a**

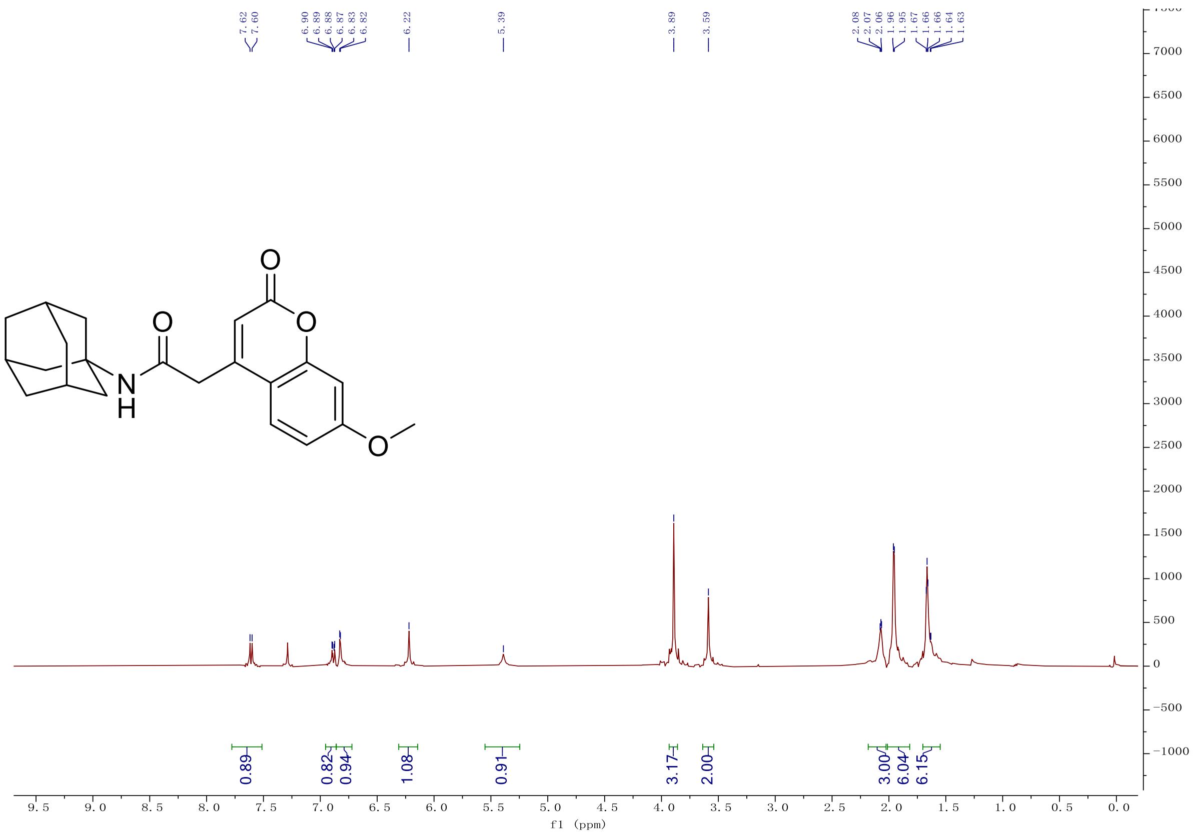

Supplementary Figure 36. ^{1}H NMR (400 MHz, $CDCl_3$) of compound **11a**

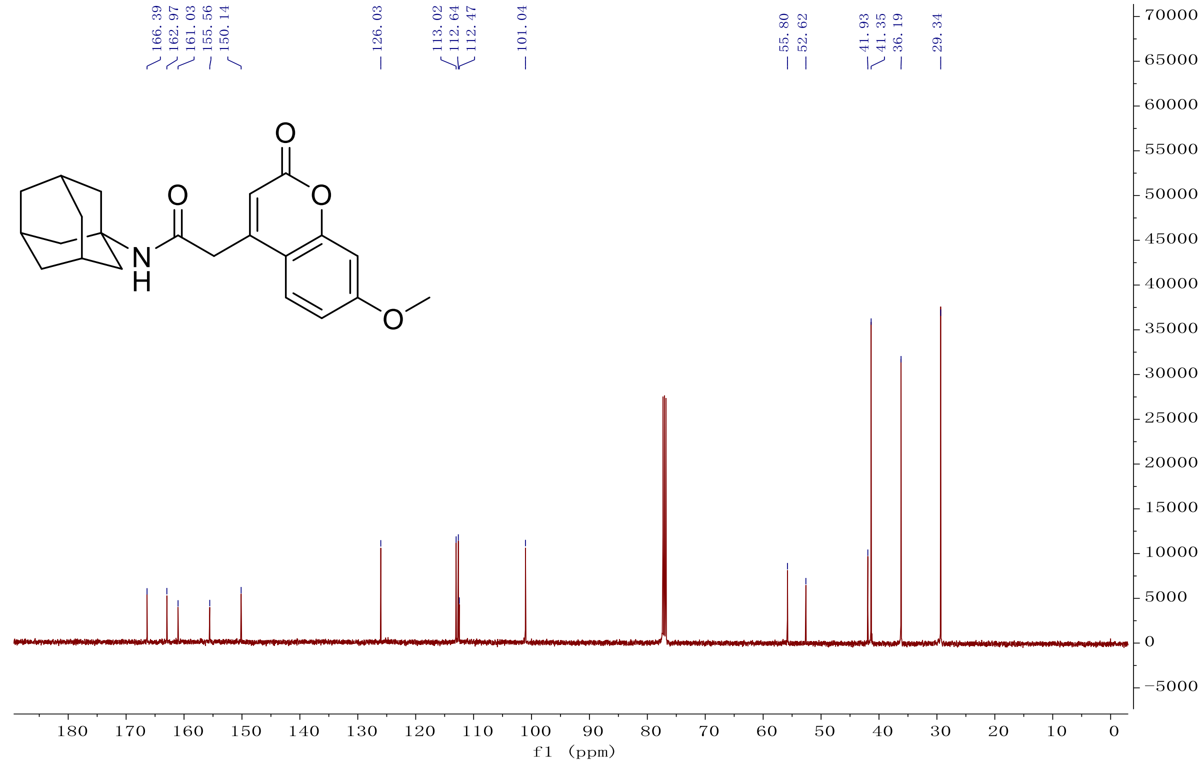

Supplementary Figure 37. ^{13}C NMR (126 MHz, $CDCl_3$) of compound **11a**

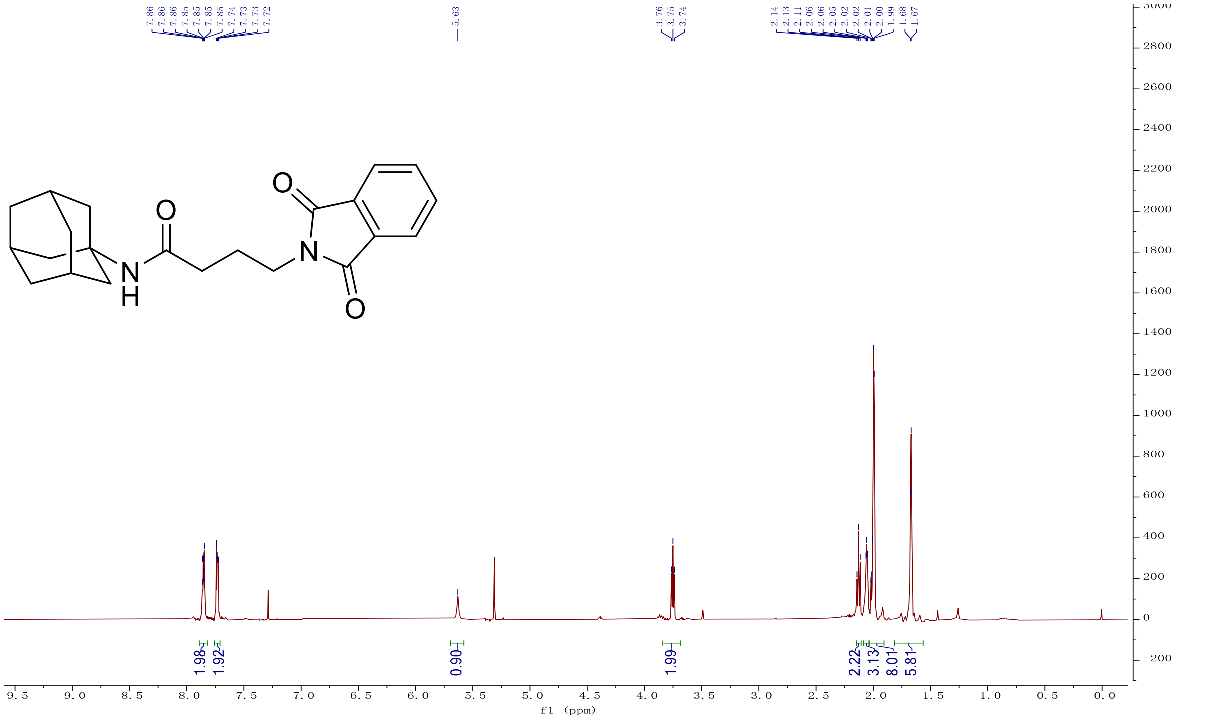

Supplementary Figure 38. ^{1}H NMR (400 MHz, $CDCl_3$) of compound **12a**

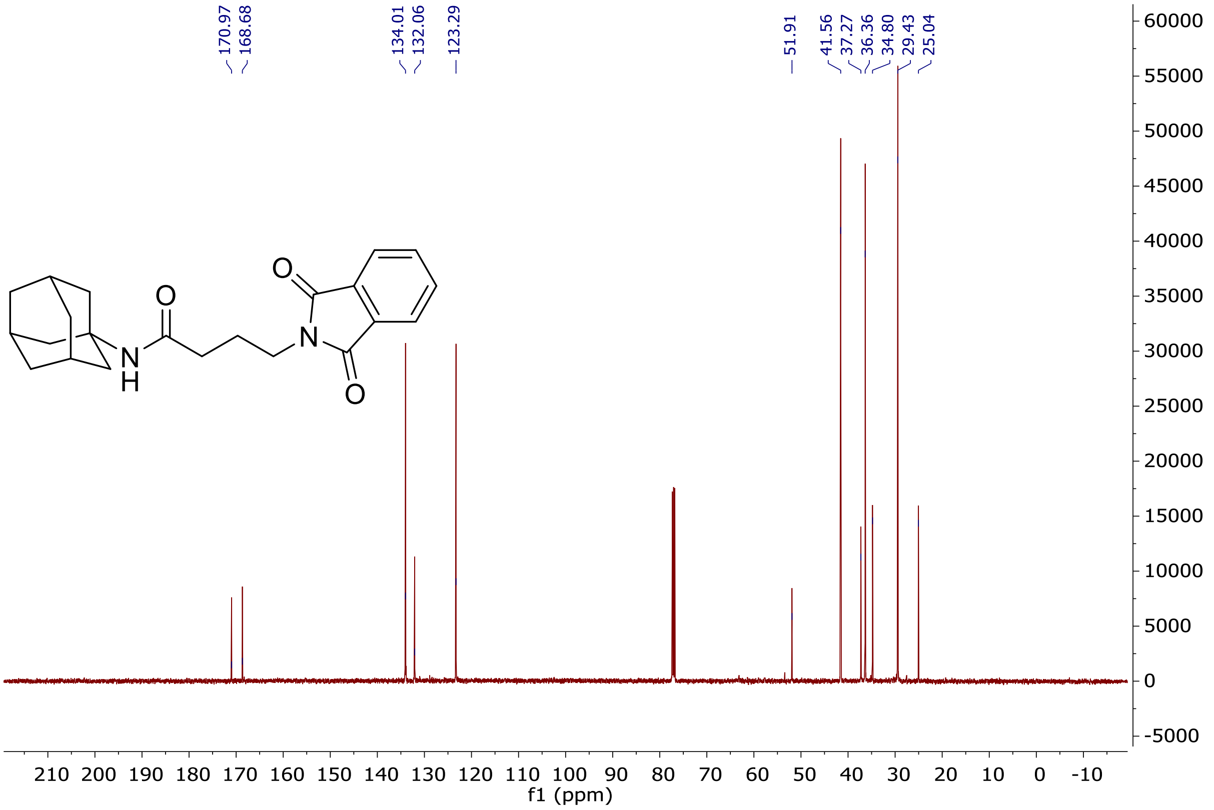

Supplementary Figure 39. ^{13}C NMR (126 MHz, $CDCl_3$) of compound **12a**

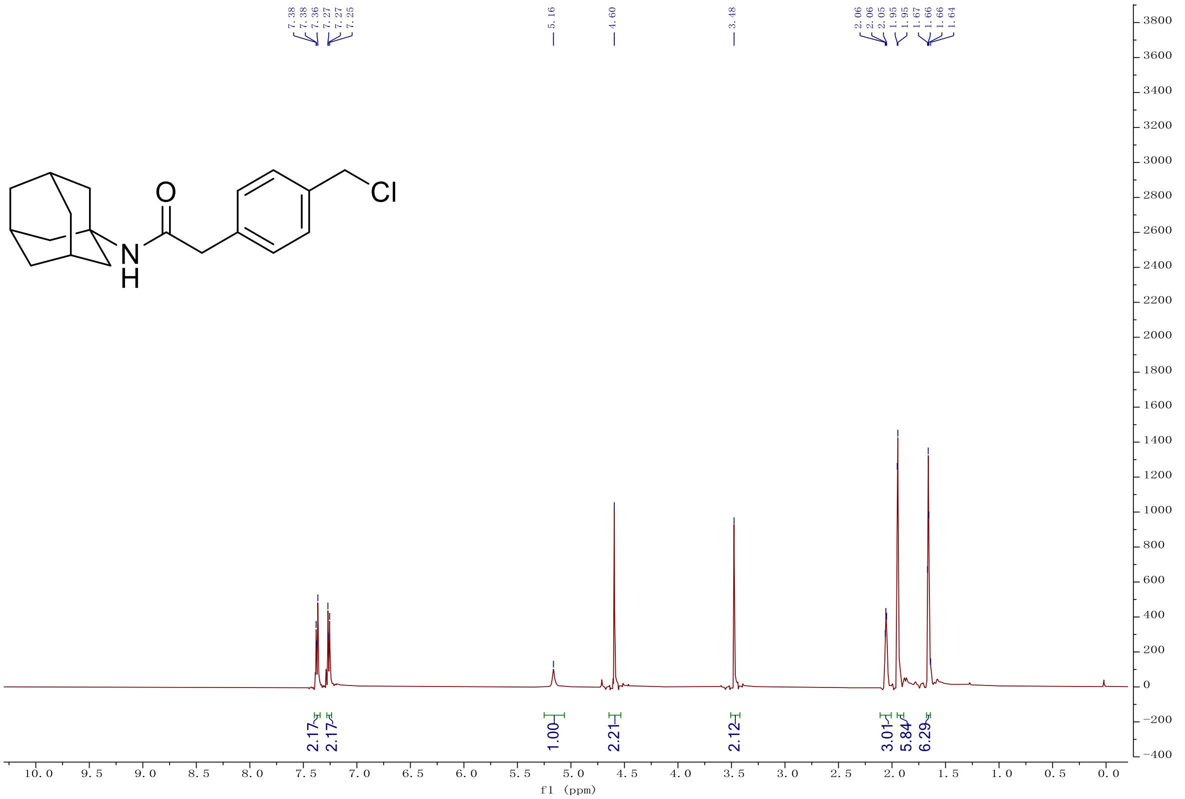

Supplementary Figure 40. ^{1}H NMR (400 MHz, $CDCl_3$) of compound **13a**

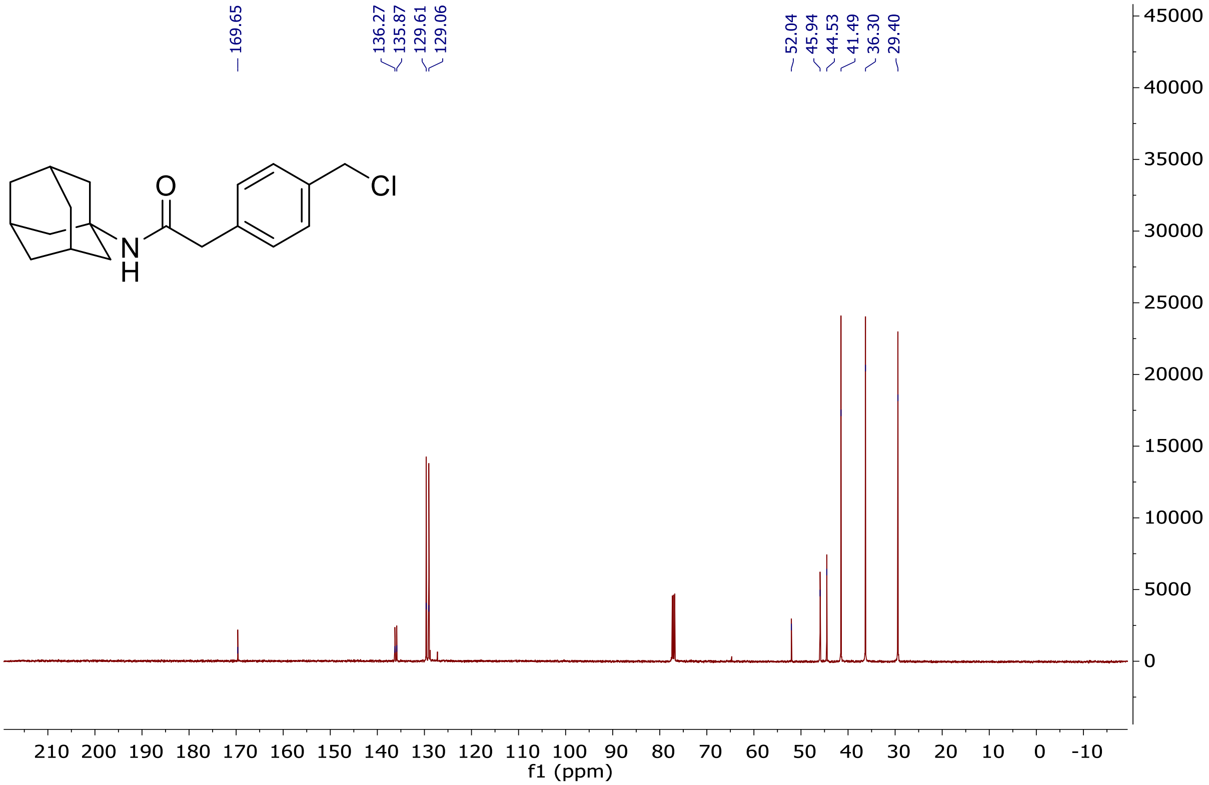

Supplementary Figure 41. ^{13}C NMR (126 MHz, $CDCl_3$) of compound **13a**

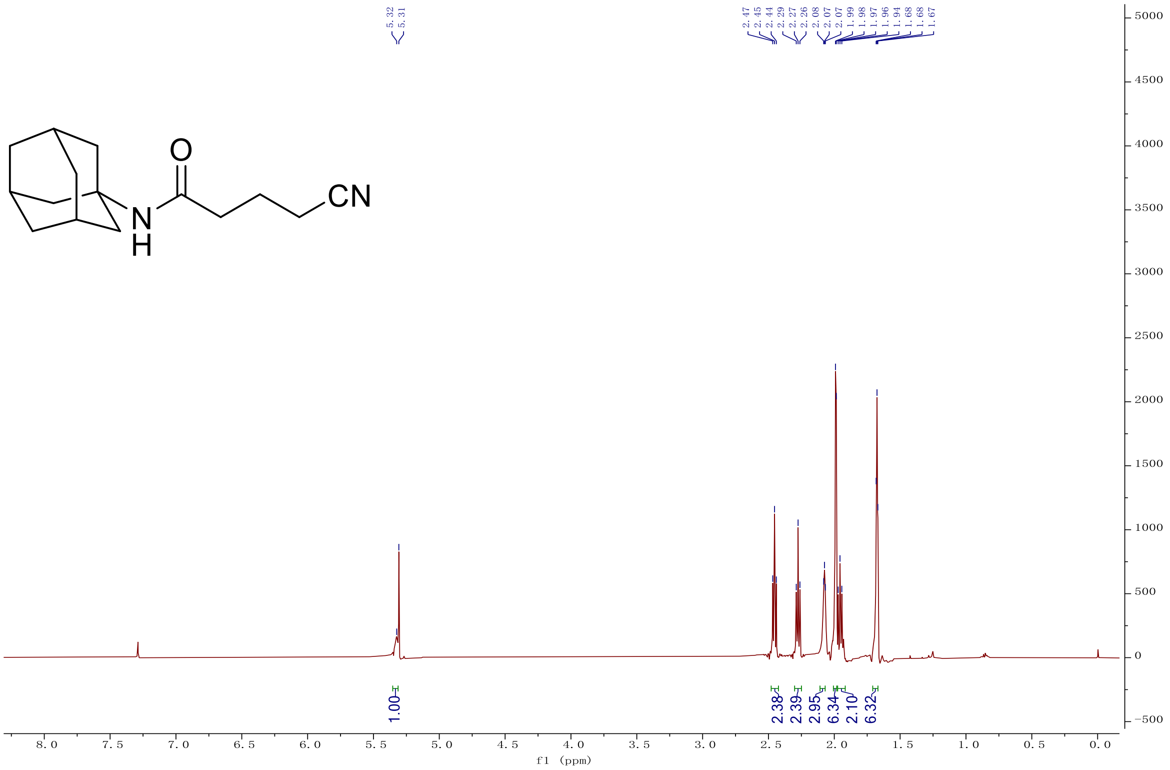

Supplementary Figure 42. ^{1}H NMR (400 MHz, $CDCl_3$) of compound **14a:**

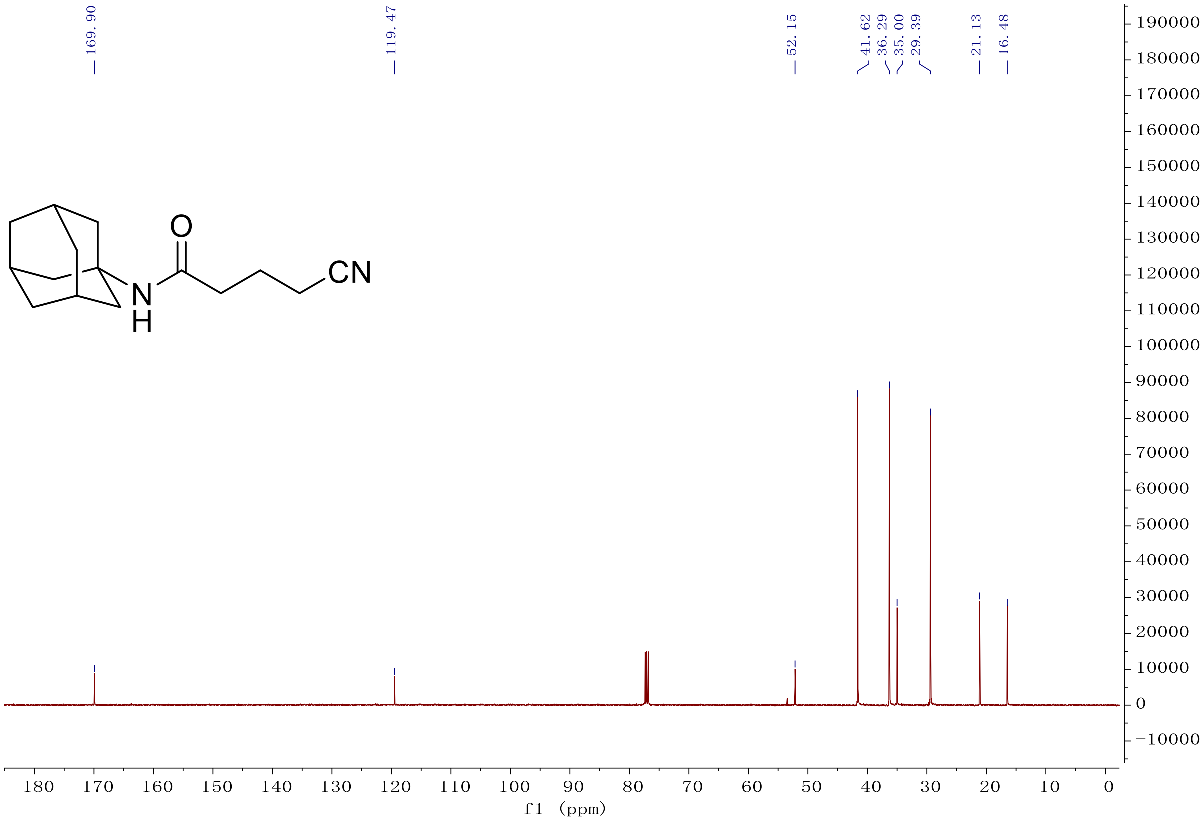

Supplementary Figure 43. ^{13}C NMR (126 MHz, $CDCl_3$) of compound **14a**

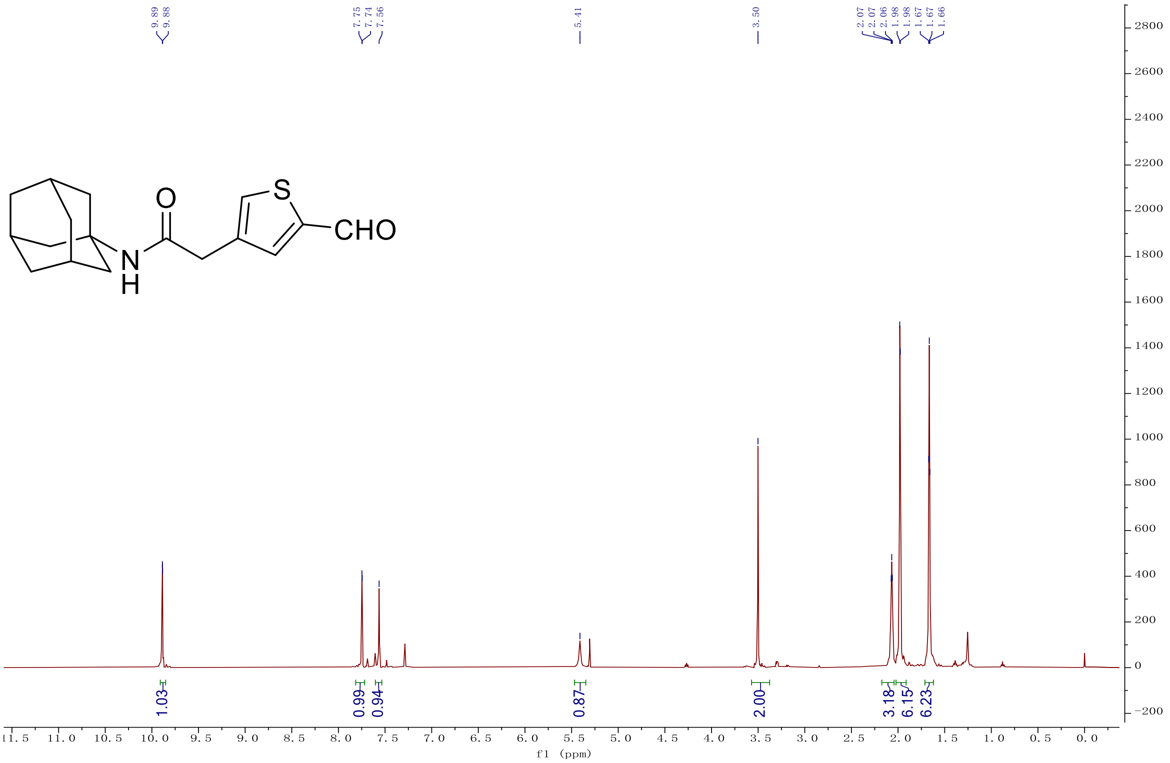

Supplementary Figure 44. ^{1}H NMR (400 MHz, $CDCl_3$) of compound **15a**

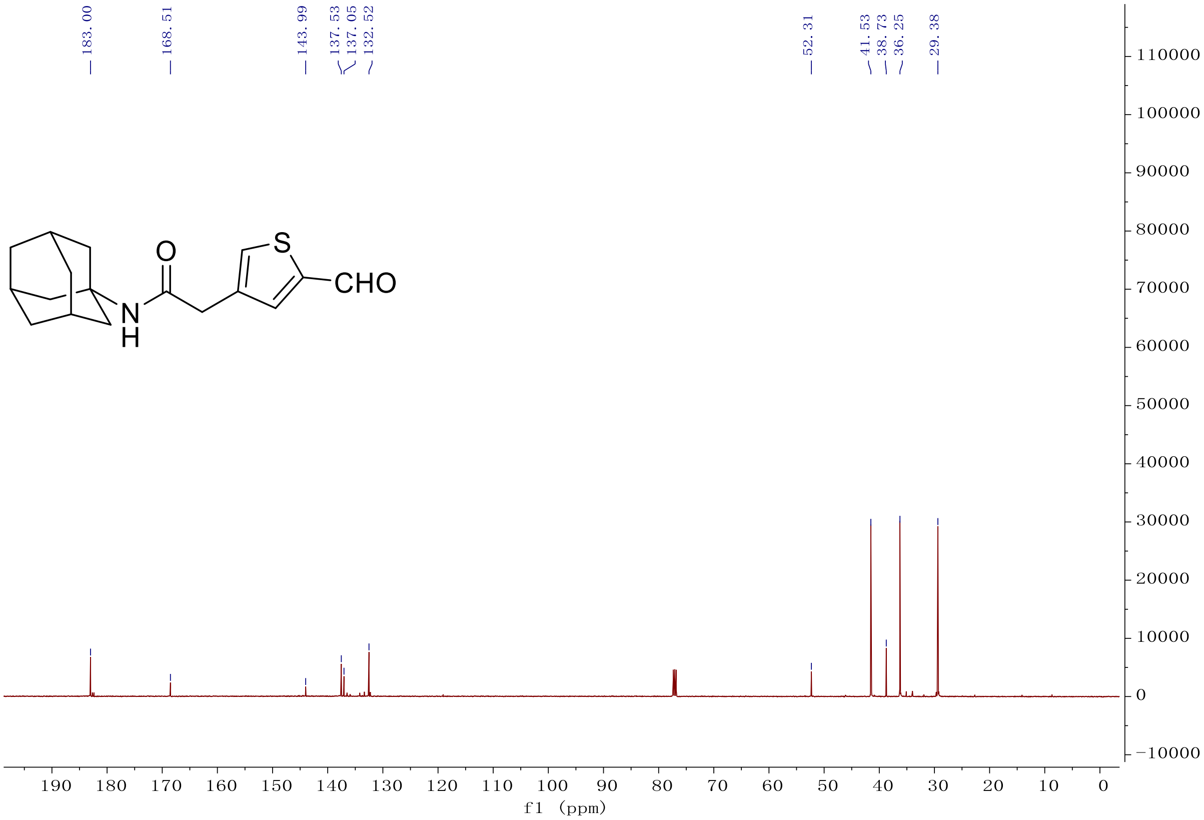

Supplementary Figure 45. ^{13}C NMR (126 MHz, $CDCl_3$) of compound **15a**

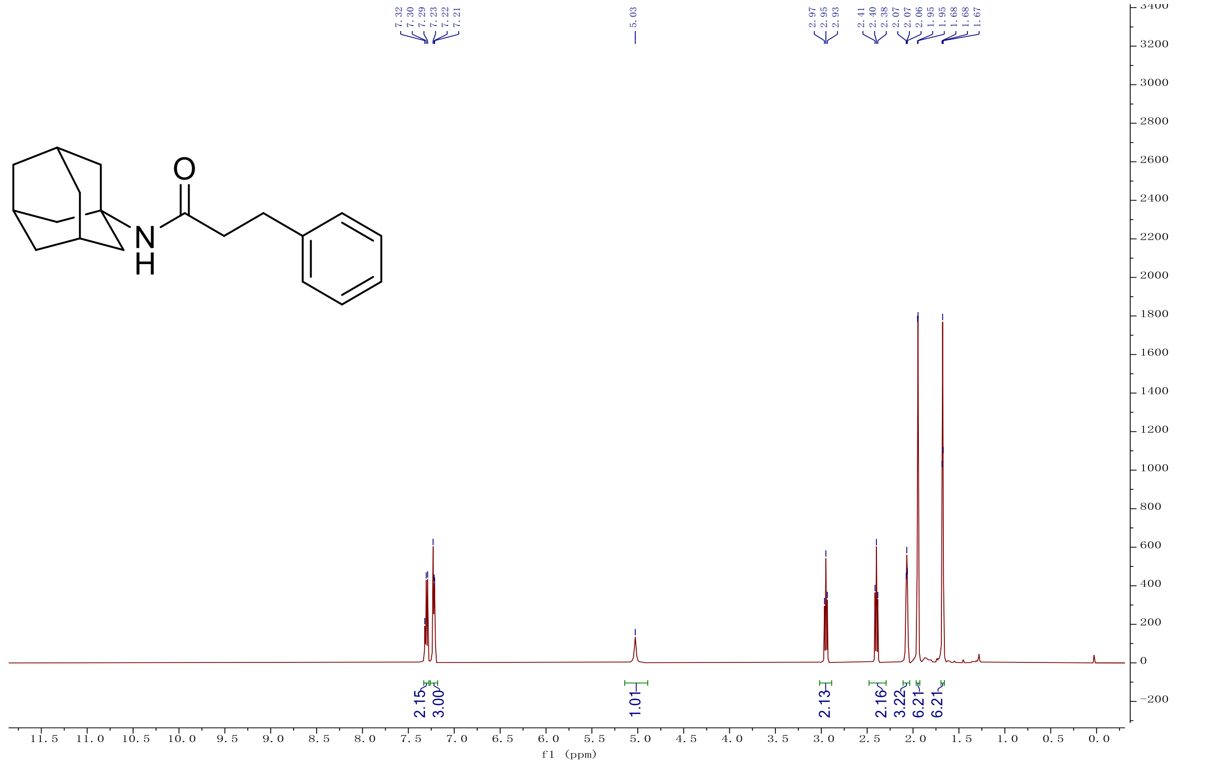

Supplementary Figure 46. ^{1}H NMR (400 MHz, $CDCl_3$) of compound **16a**

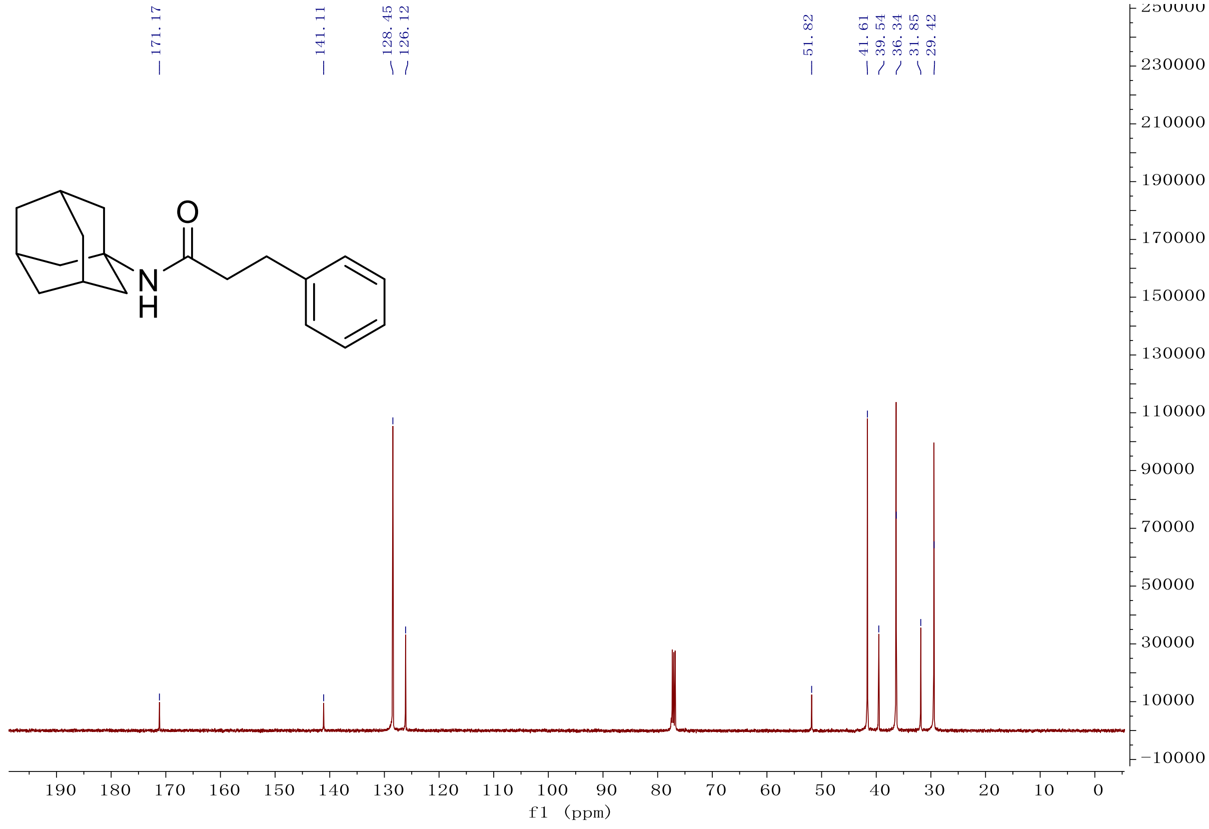

Supplementary Figure 47. ^{13}C NMR (126 MHz, $CDCl_3$) of compound **16a**

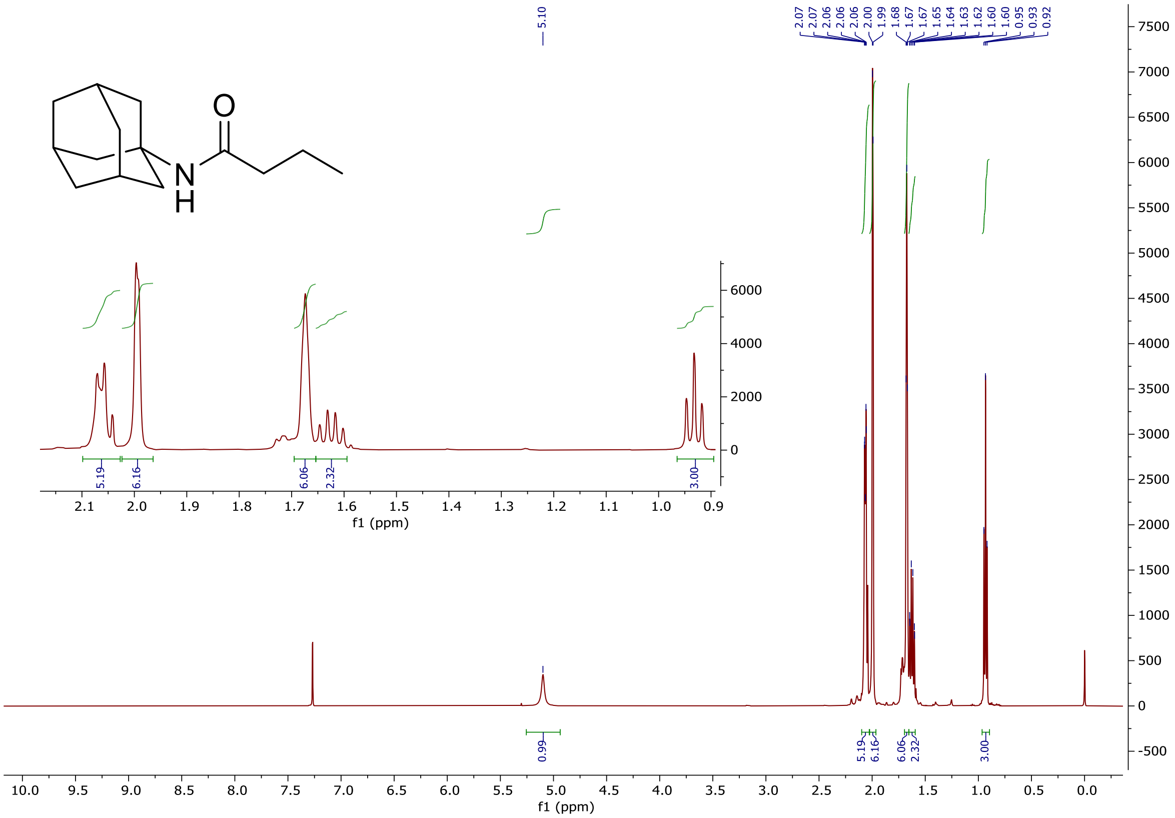

Supplementary Figure 48. ^{1}H NMR (400 MHz, $CDCl_3$) of compound **17a**

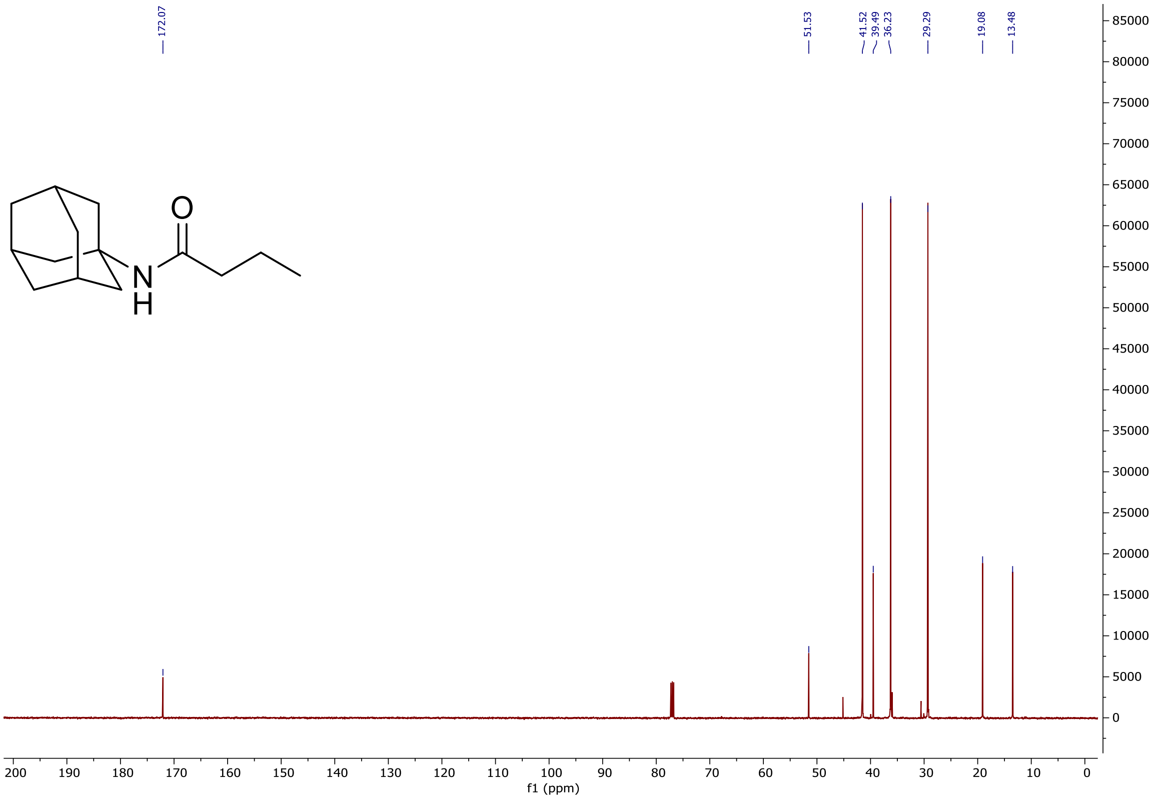

Supplementary Figure 49. ^{13}C NMR (126 MHz, $CDCl_3$) of compound **17a**

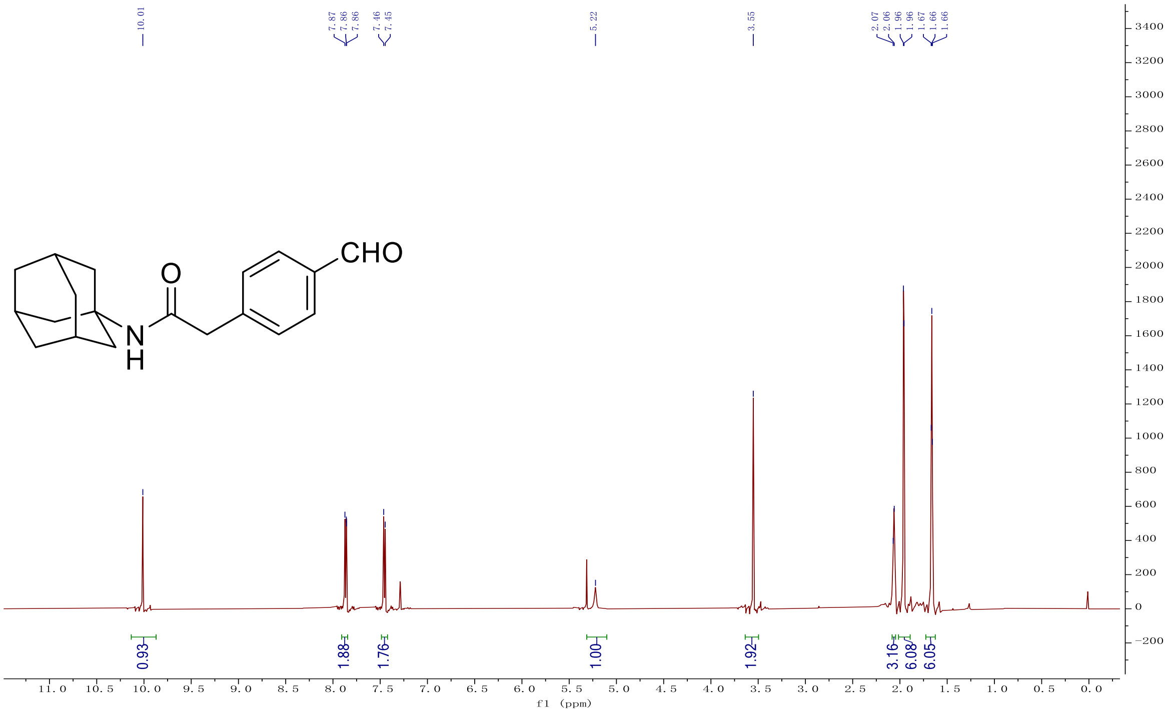

Supplementary Figure 50. ^{1}H NMR (400 MHz, $CDCl_3$) of compound**18a**

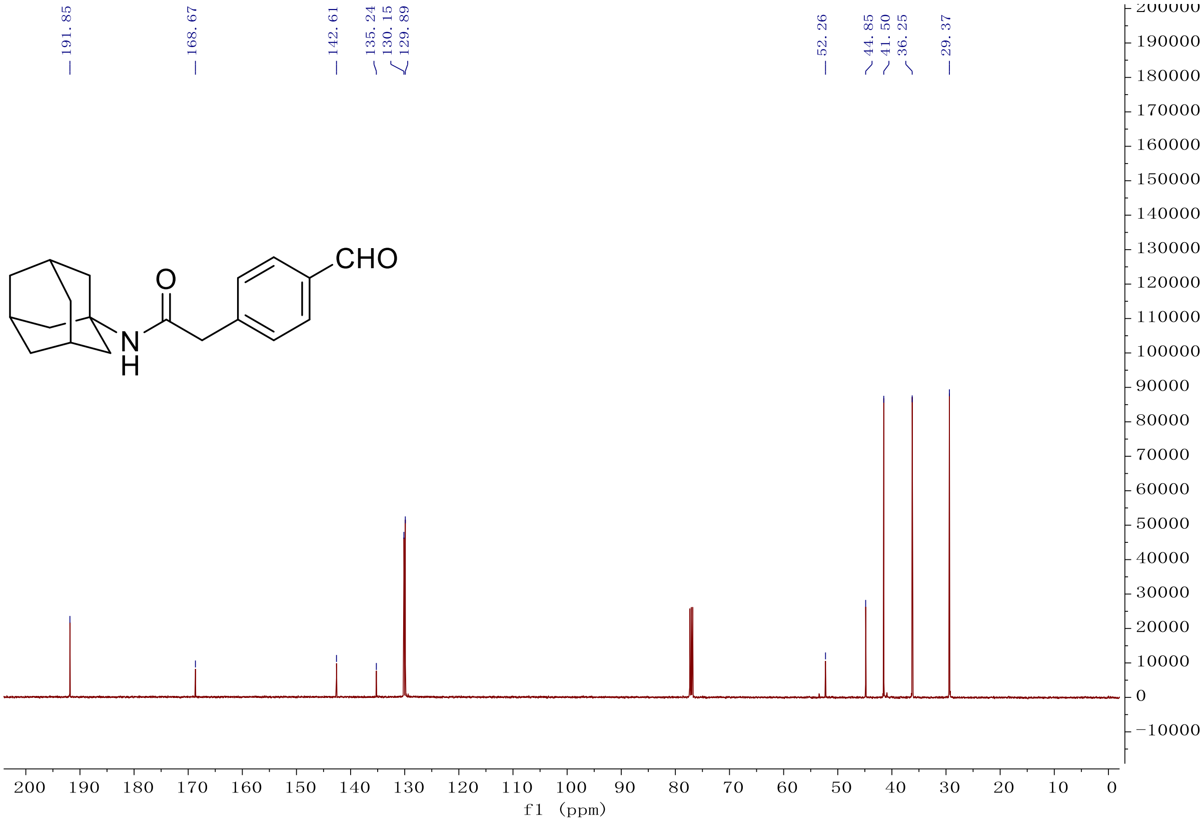

Supplementary Figure 51. ^{13}C NMR (126 MHz, $CDCl_3$) of compound **18a**

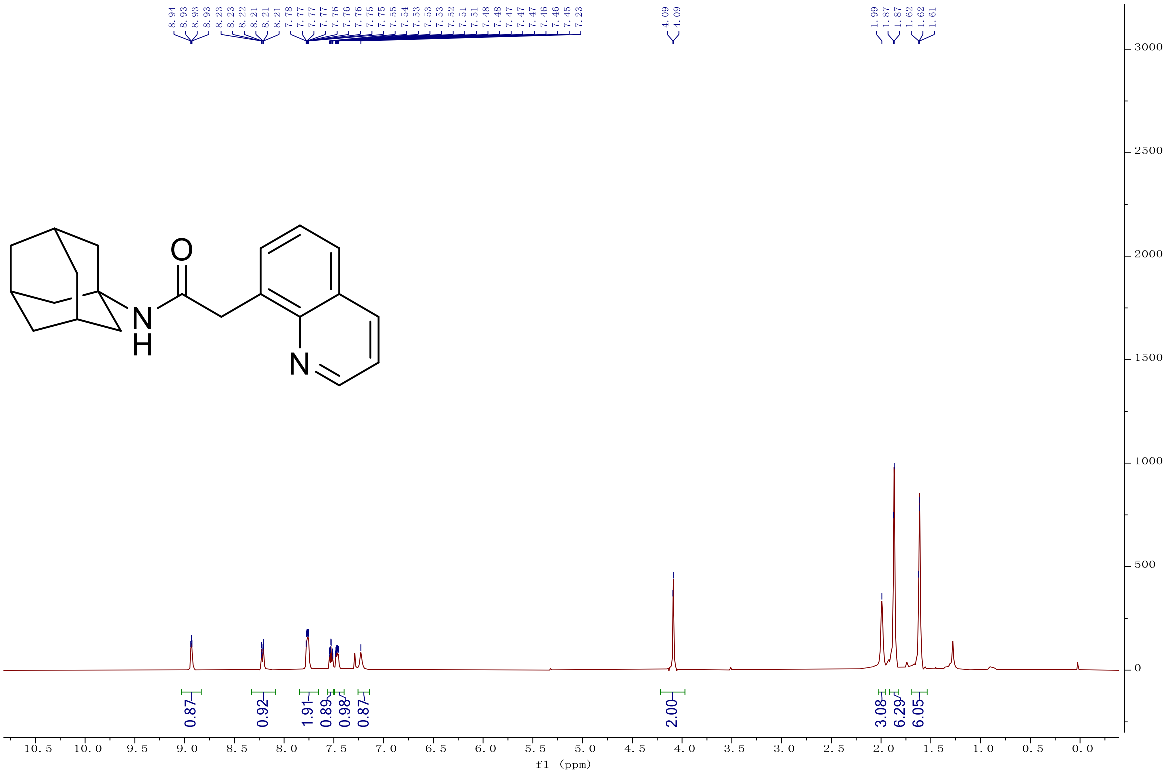

Supplementary Figure 52. ^{1}H NMR (400 MHz, $CDCl_3$) of compound **19a**

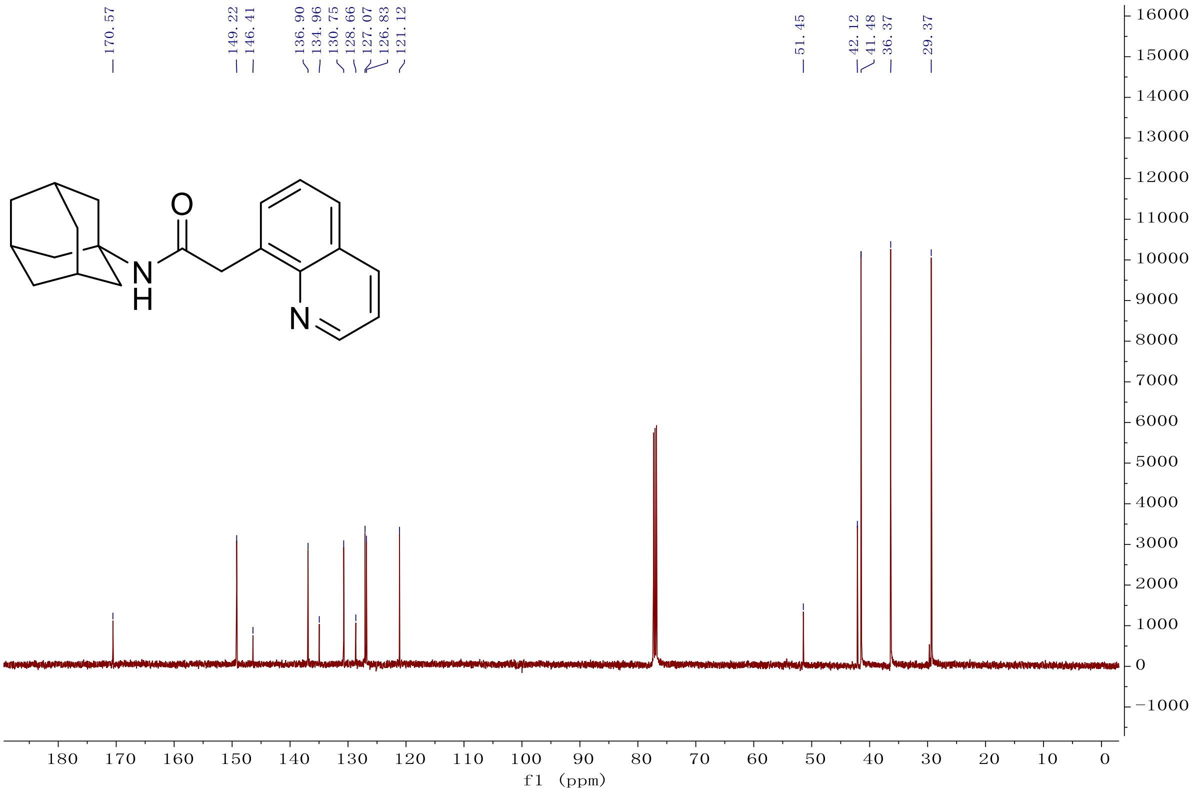

Supplementary Figure 53. ^{13}C NMR (126 MHz, $CDCl_3$) of compound **19a**

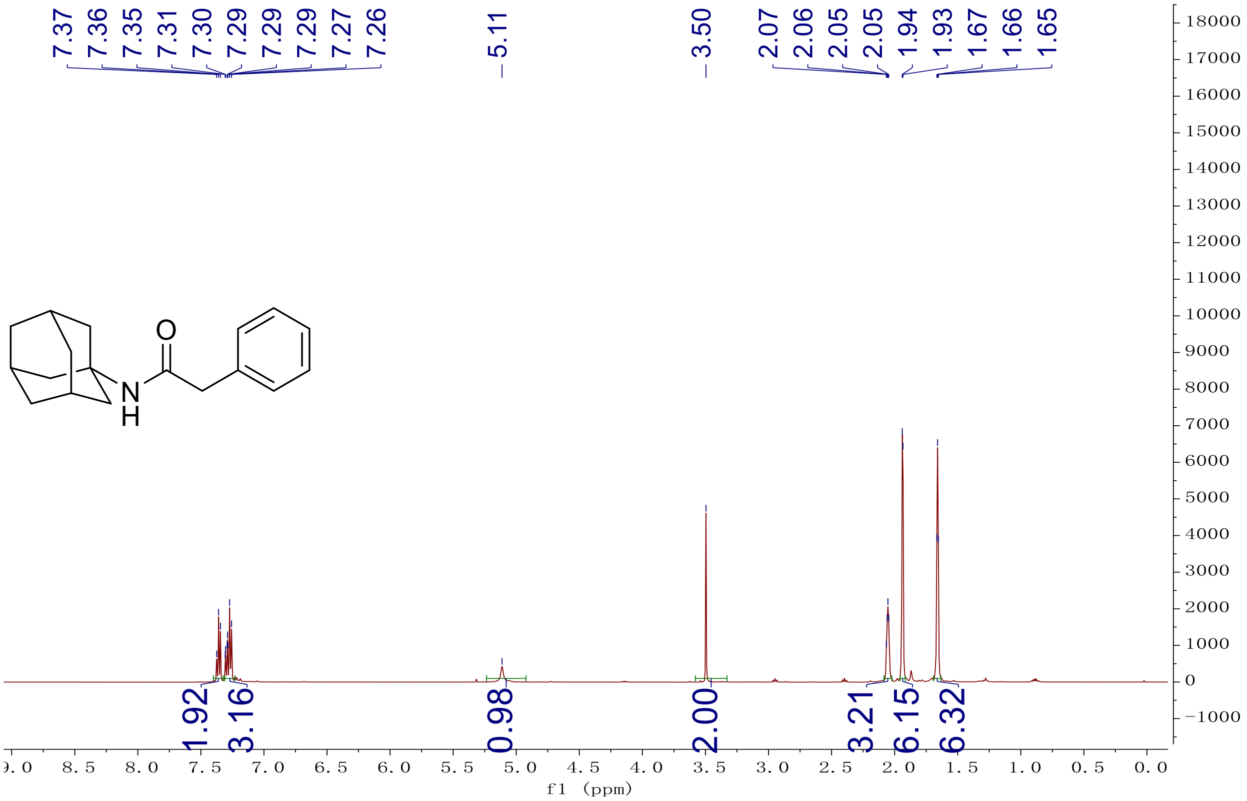

Supplementary Figure 54. ^{1}H NMR (400 MHz, $CDCl_3$) of compound **20a**

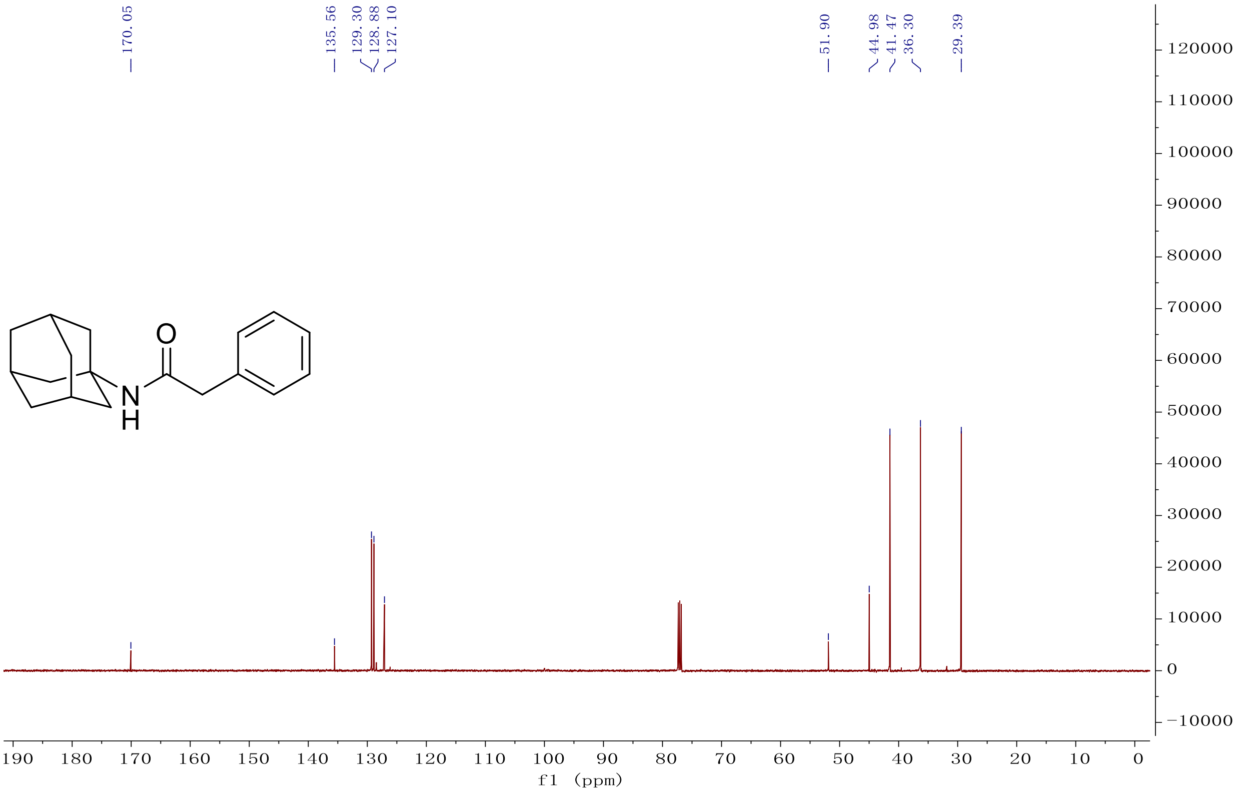

Supplementary Figure 55. ^{13}C NMR (126 MHz, $CDCl_3$) of compound **20a**

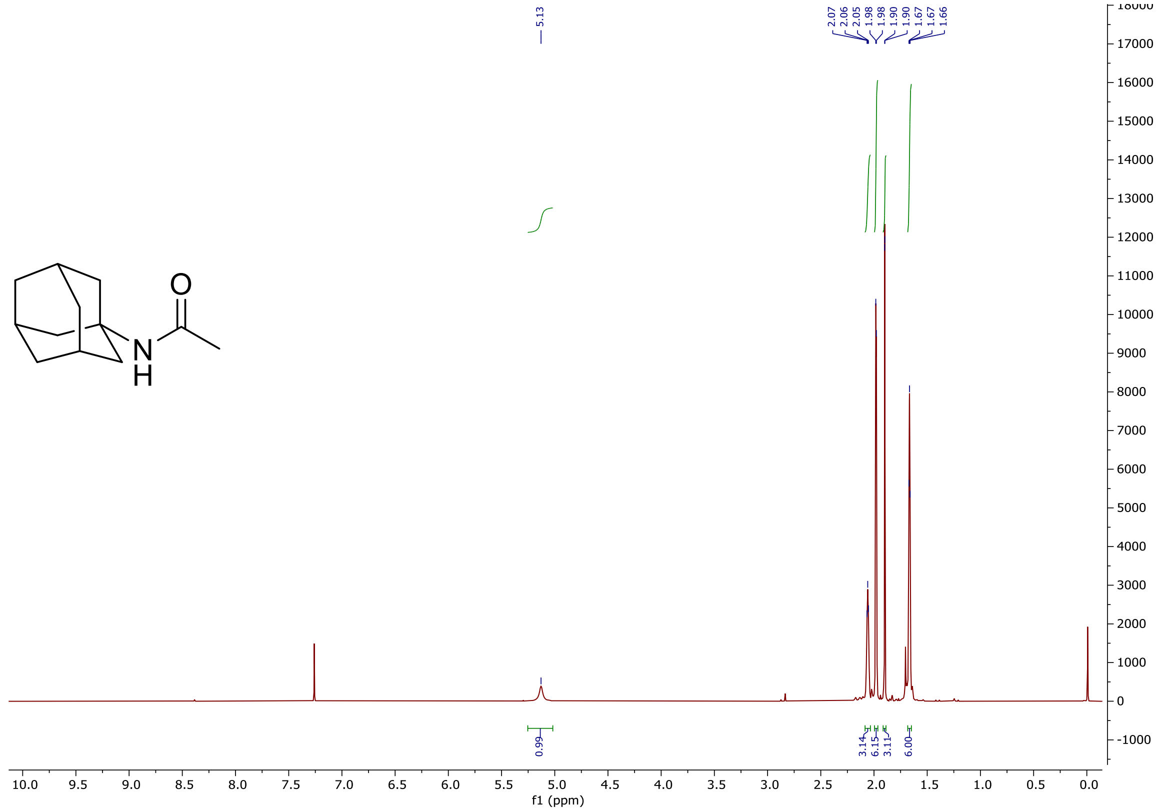

Supplementary Figure 56. ^{1}H NMR (400 MHz, $CDCl_3$) of compound **21a**

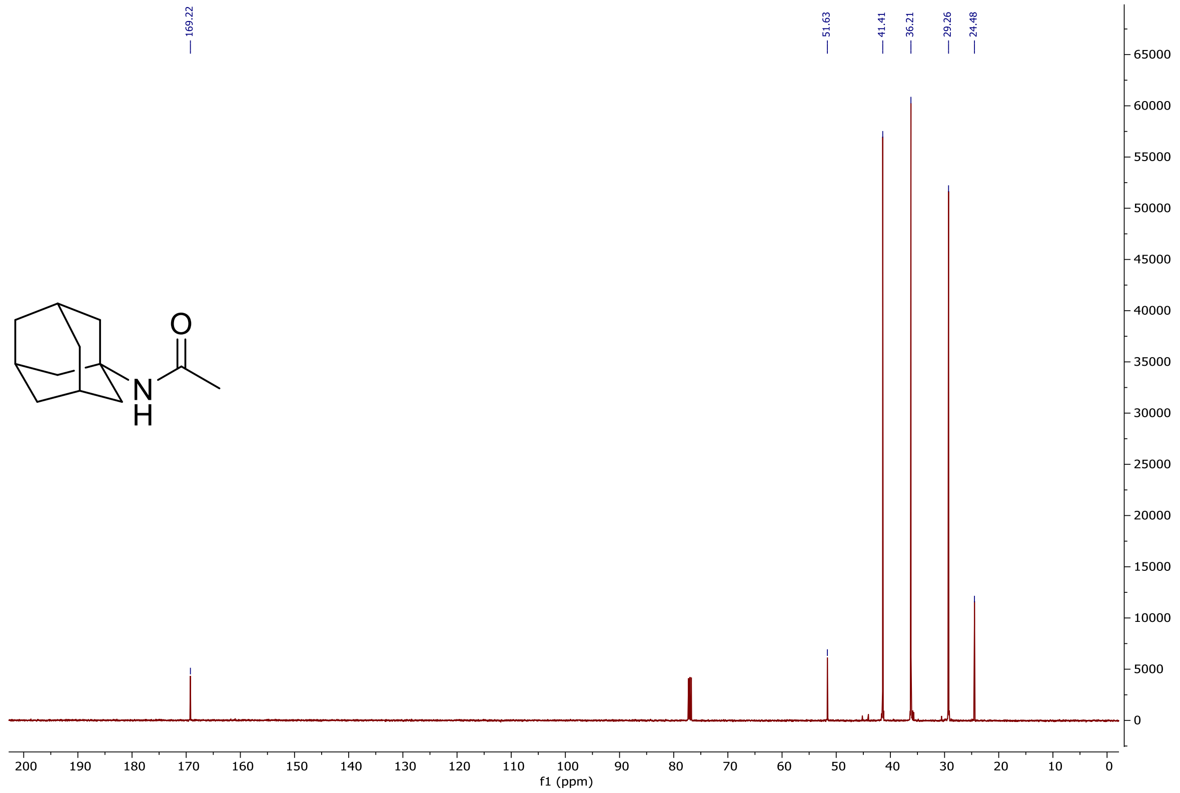

Supplementary Figure 57. ^{13}C NMR (126 MHz, $CDCl_3$) of compound **22a**

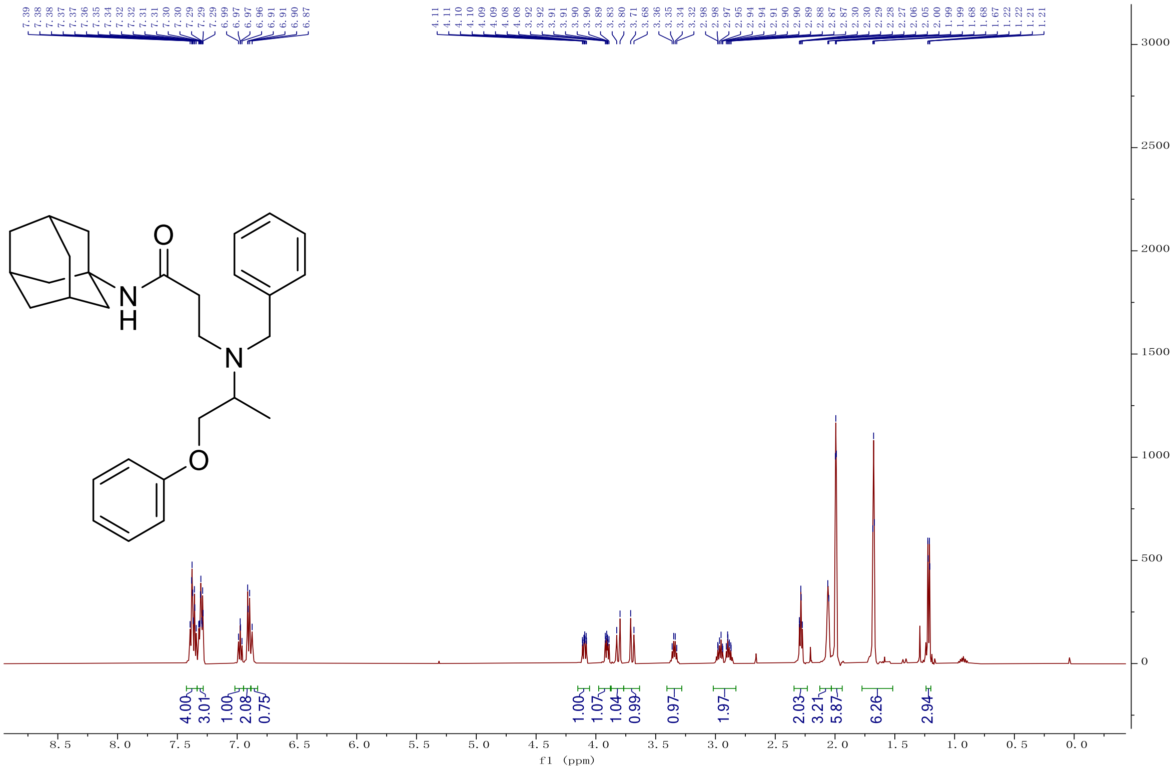

Supplementary Figure 58. ^{1}H NMR (400 MHz, $CDCl_3$) of compound **22a**

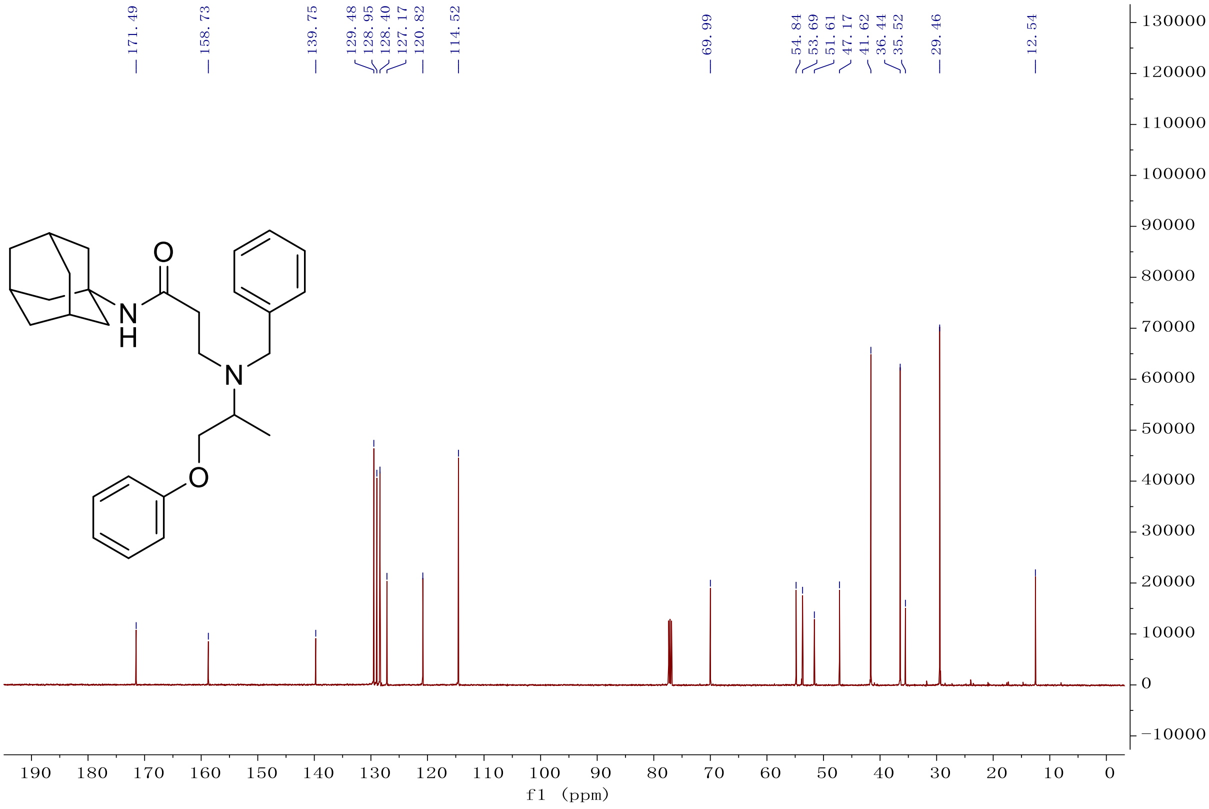

Supplementary Figure 59. ^{13}C NMR (126 MHz, $CDCl_3$) of compound **22a**

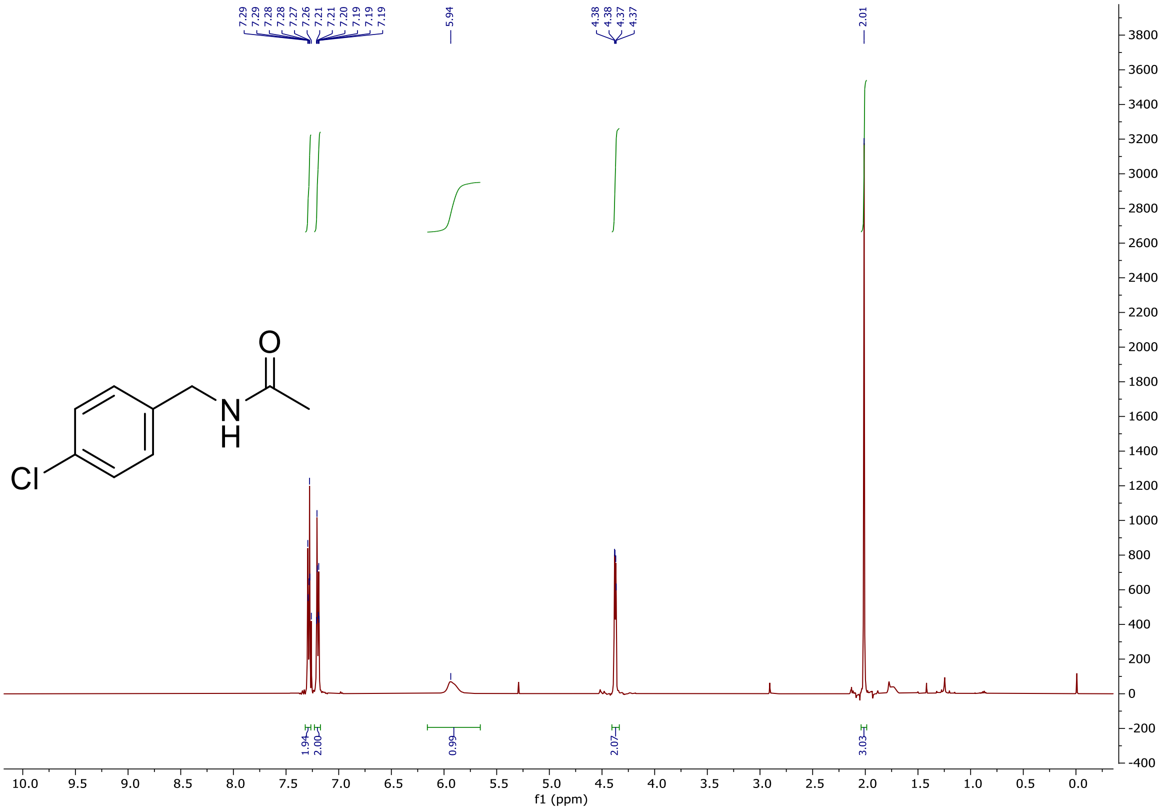

Supplementary Figure 60. ^{1}H NMR (400 MHz, $CDCl_3$) of compound **23a**

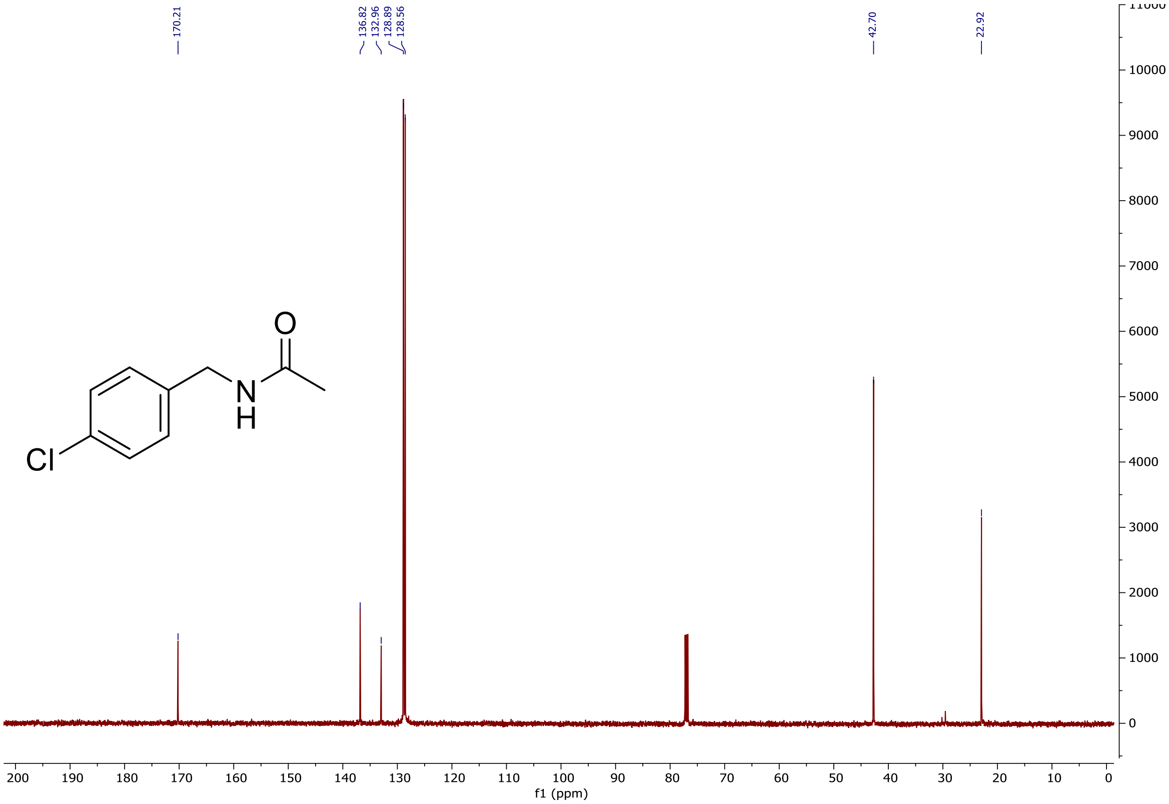

Supplementary Figure 61. ^{13}C NMR (126 MHz, $CDCl_3$) of compound **23a**

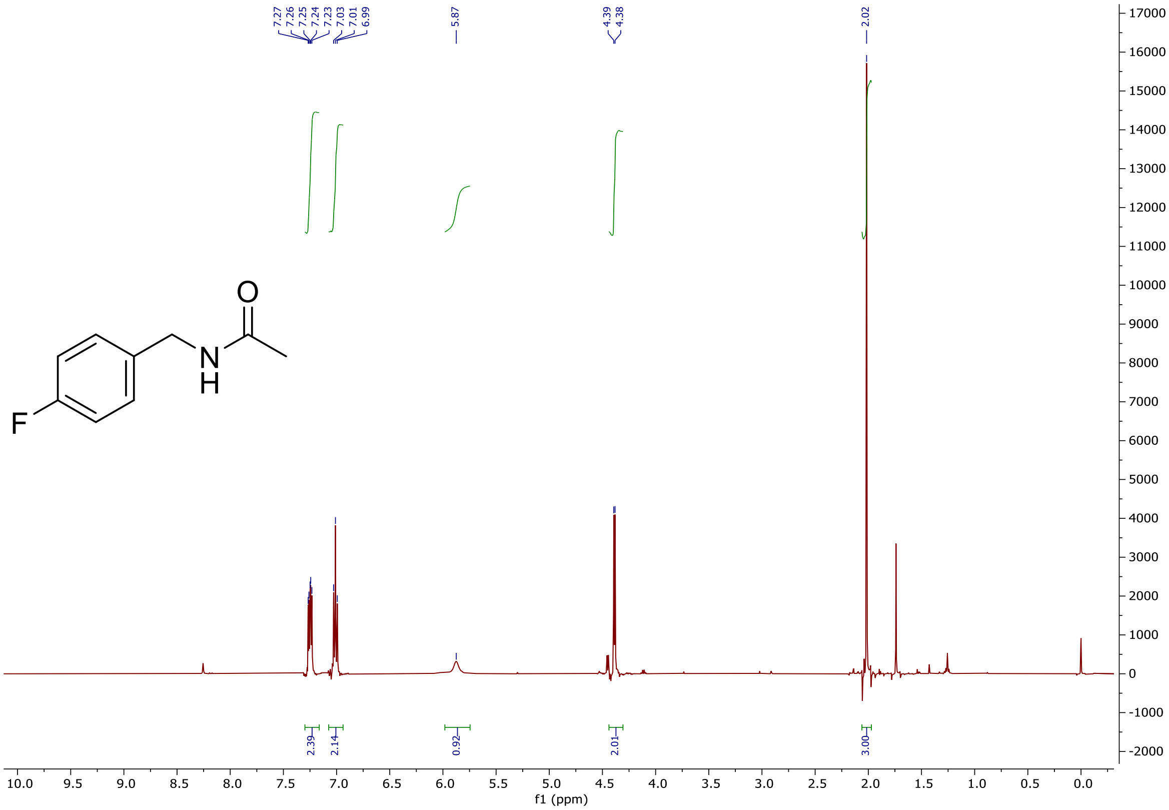

Supplementary Figure 62. ^{1}H NMR (400 MHz, $CDCl_3$) of compound **24a**

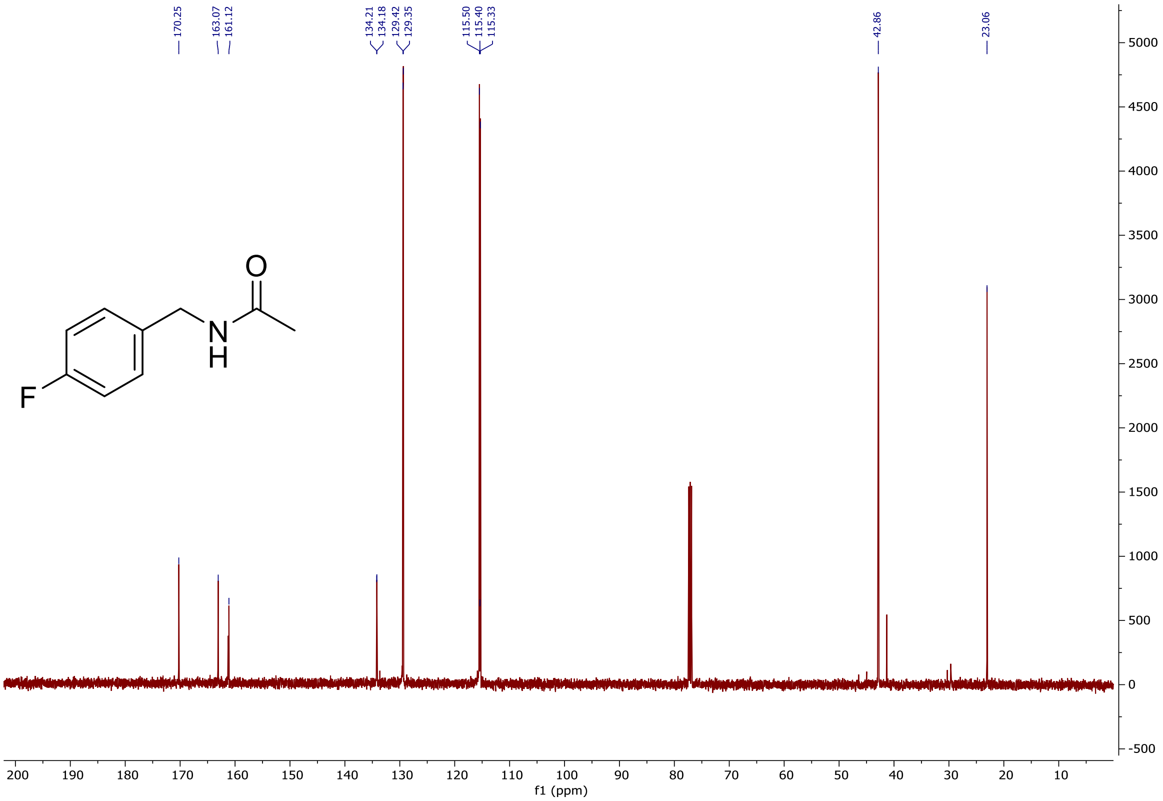

Supplementary Figure 63. ^{13}C NMR (126 MHz, $CDCl_3$) of compound **24a**

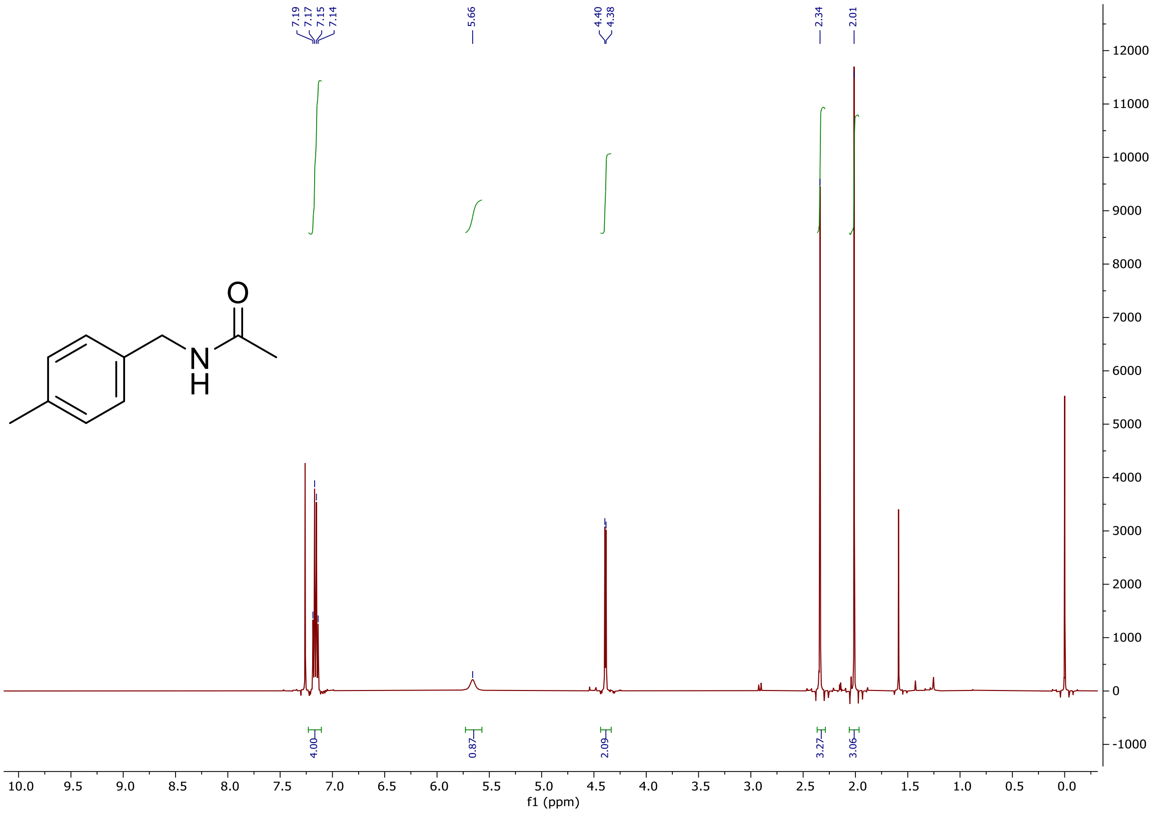

Supplementary Figure 64. ^{1}H NMR (400 MHz, $CDCl_3$) of compound **25a**

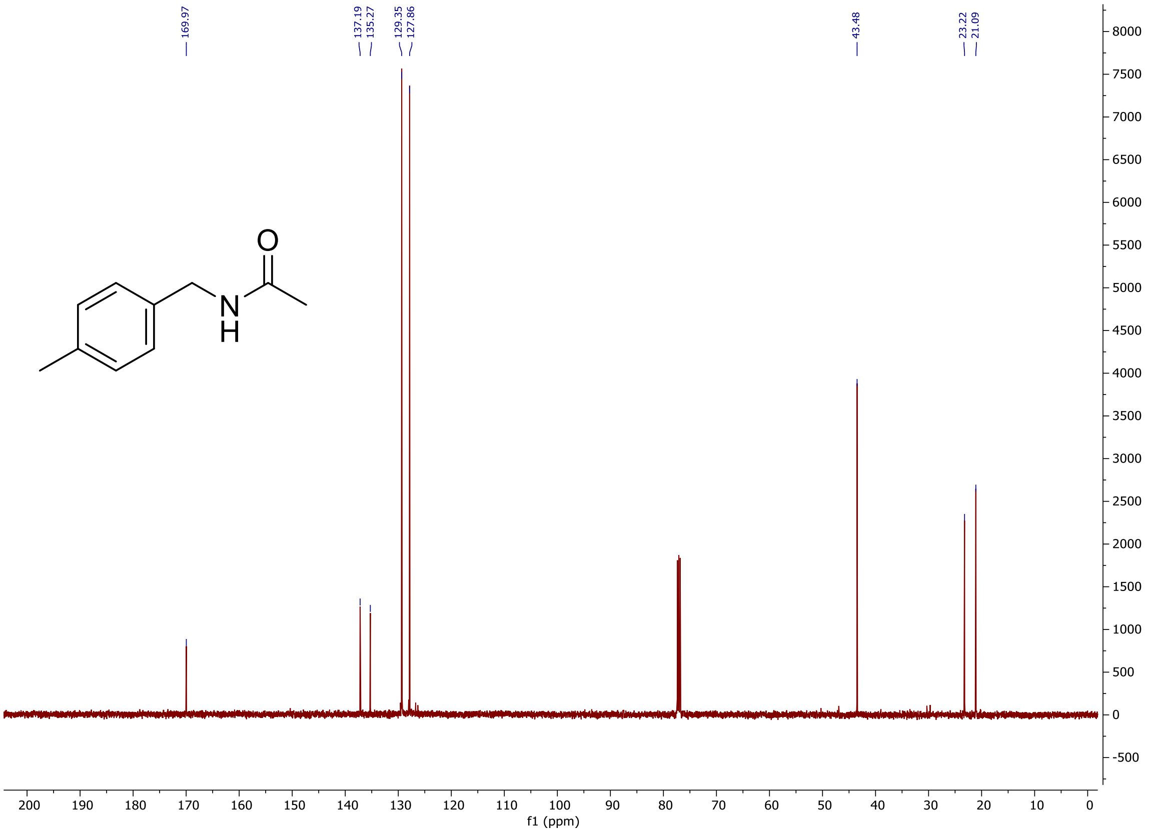

Supplementary Figure 65. ^{13}C NMR (126 MHz, $CDCl_3$) of compound **25a**

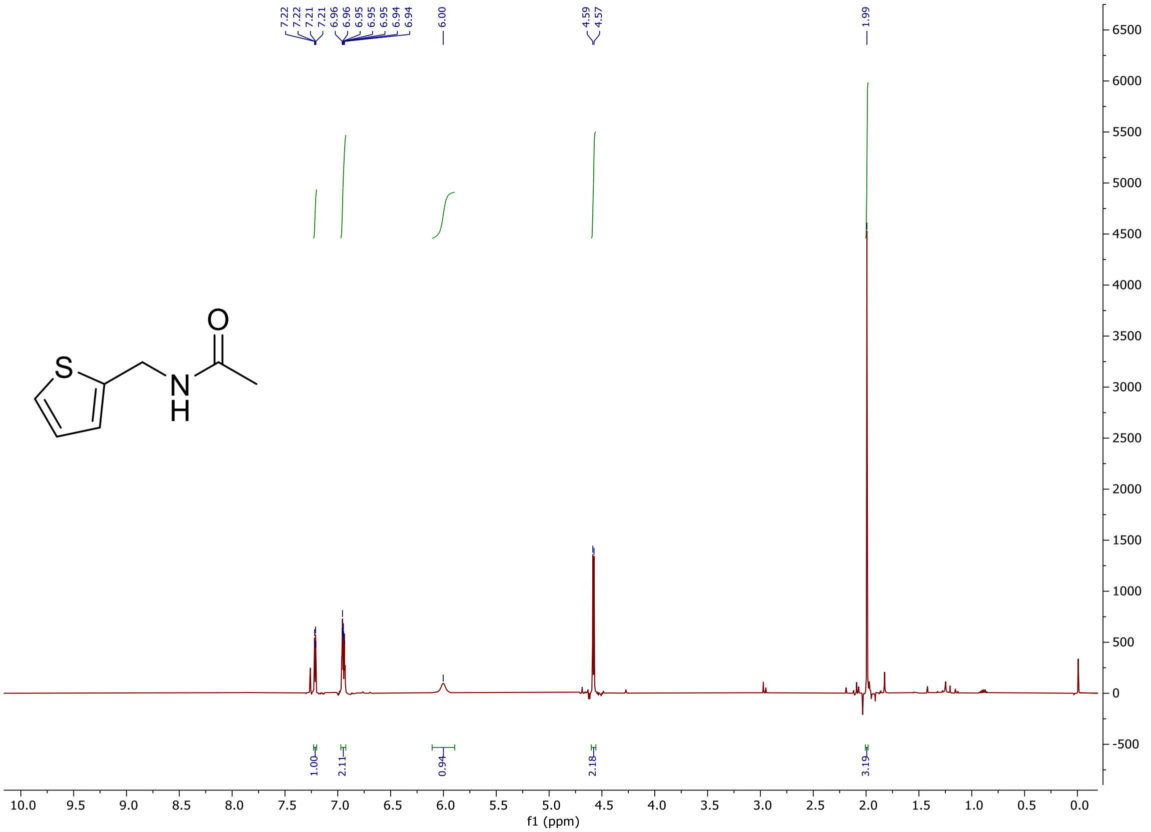

Supplementary Figure 66. ^{1}H NMR (400 MHz, $CDCl_3$) of compound **26a**

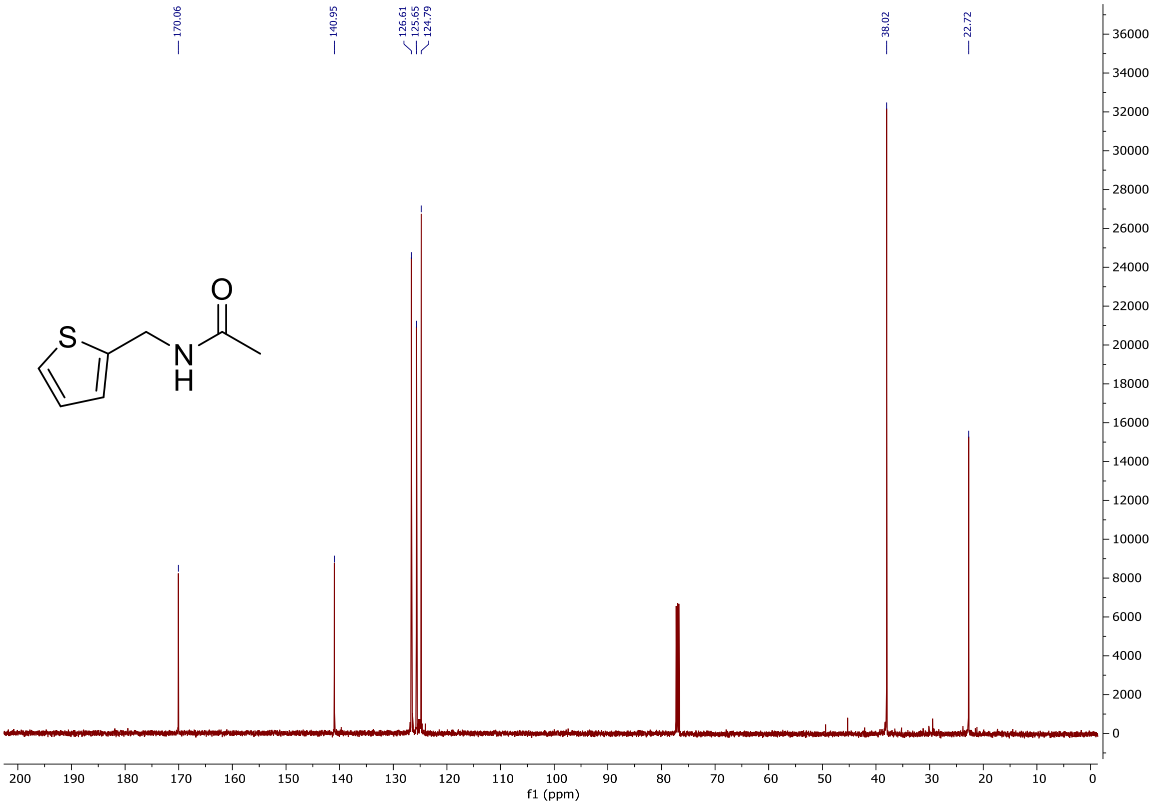

Supplementary Figure 67. ^{13}C NMR (126 MHz, $CDCl_3$) of compound **26a**

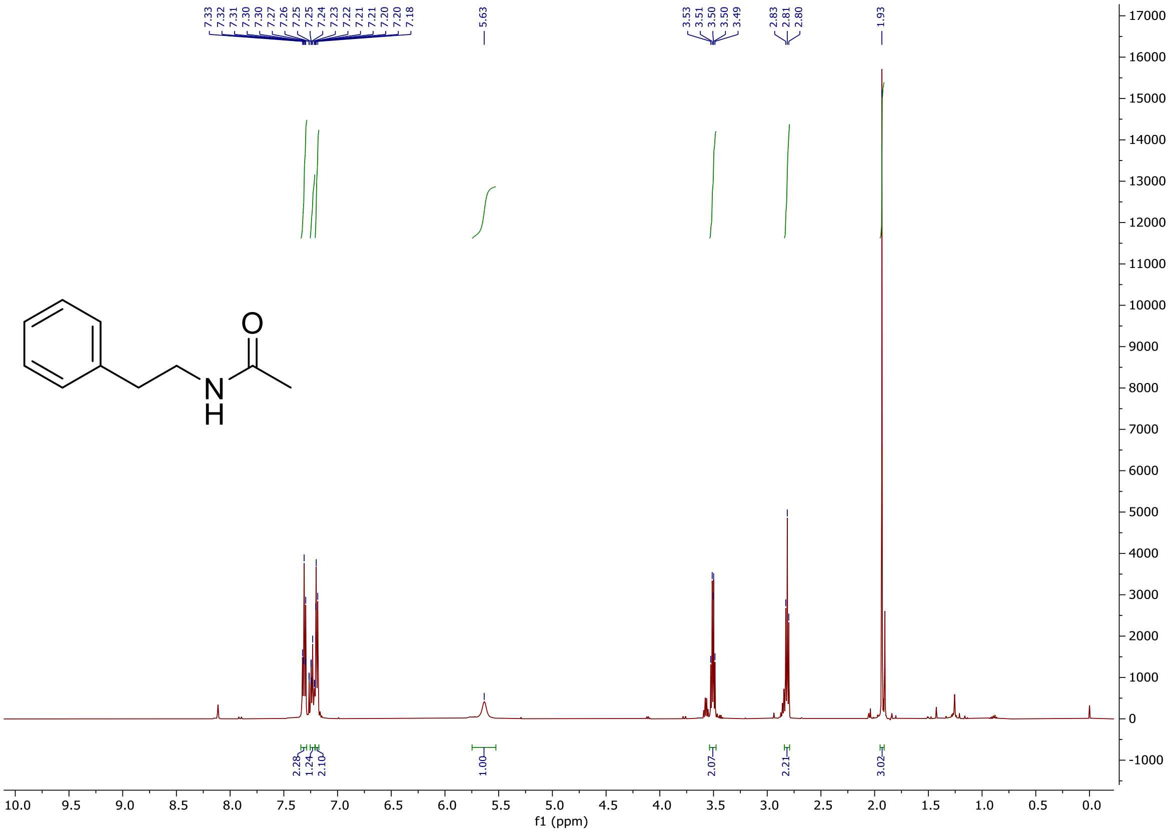

Supplementary Figure 68. ^{1}H NMR (400 MHz, $CDCl_3$) of compound **27a**

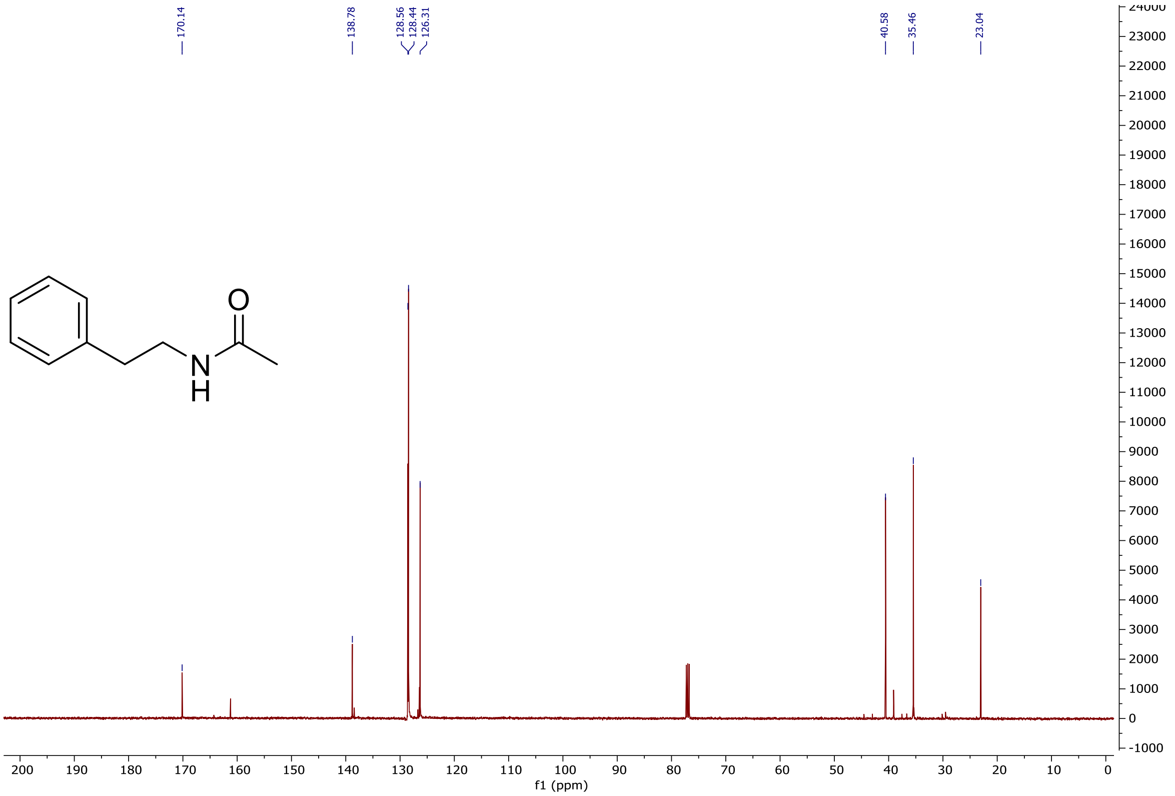

Supplementary Figure 69. ^{13}C NMR (126 MHz, $CDCl_3$) of compound **27a**

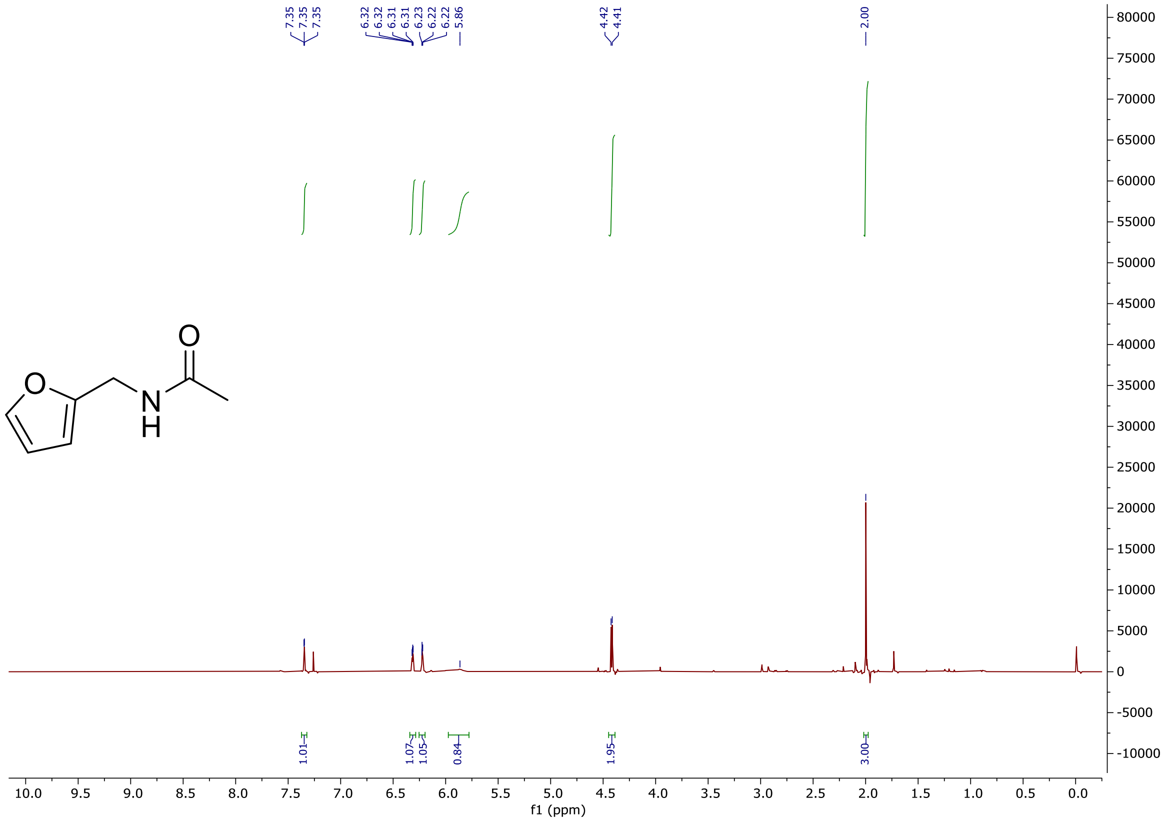

Supplementary Figure 70. ^{1}H NMR (400 MHz, $CDCl_3$) of compound **28a**

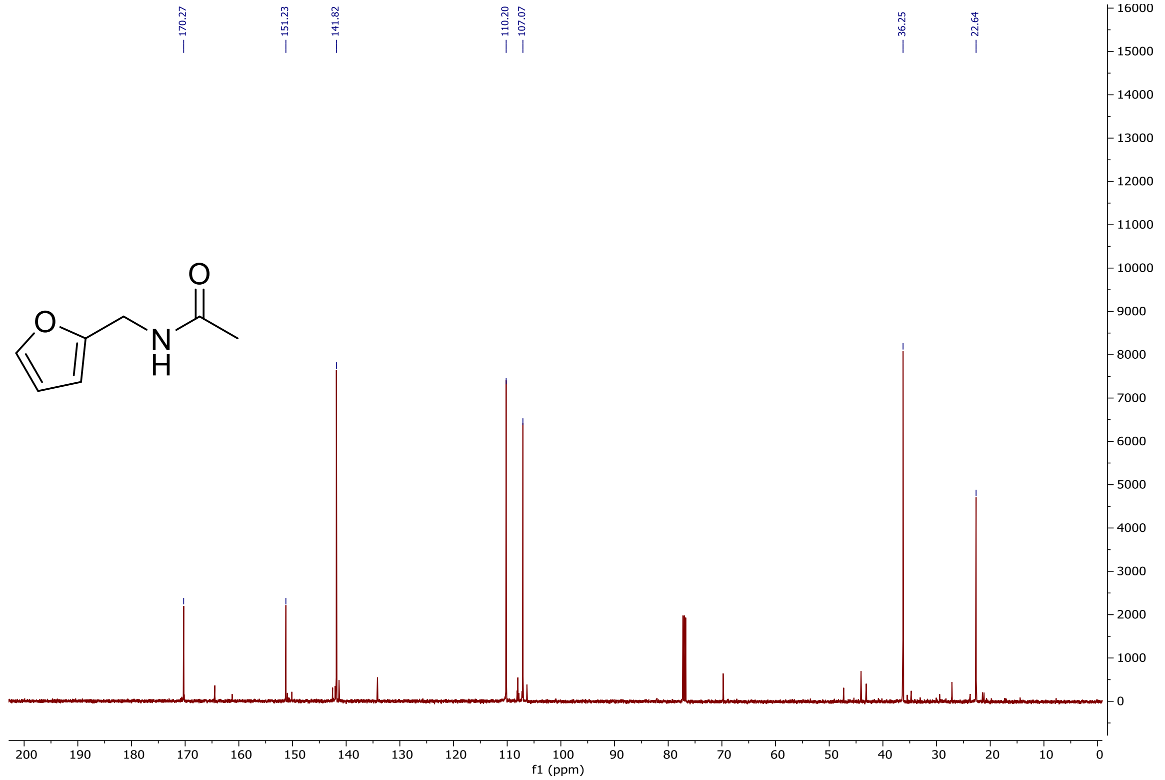

Supplementary Figure 71. ^{13}C NMR (126 MHz, $CDCl_3$) of compound **28a**

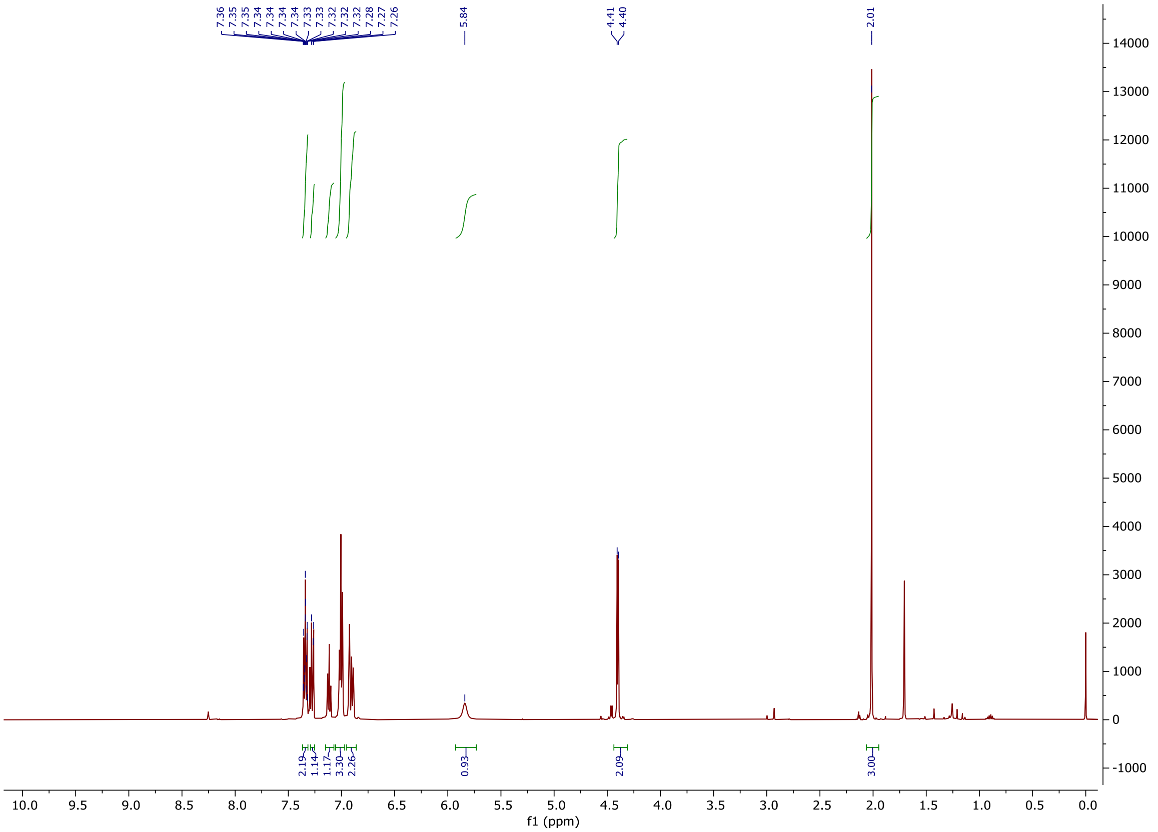

Supplementary Figure 72. ^{1}H NMR (400 MHz, $CDCl_3$) of compound **29a**

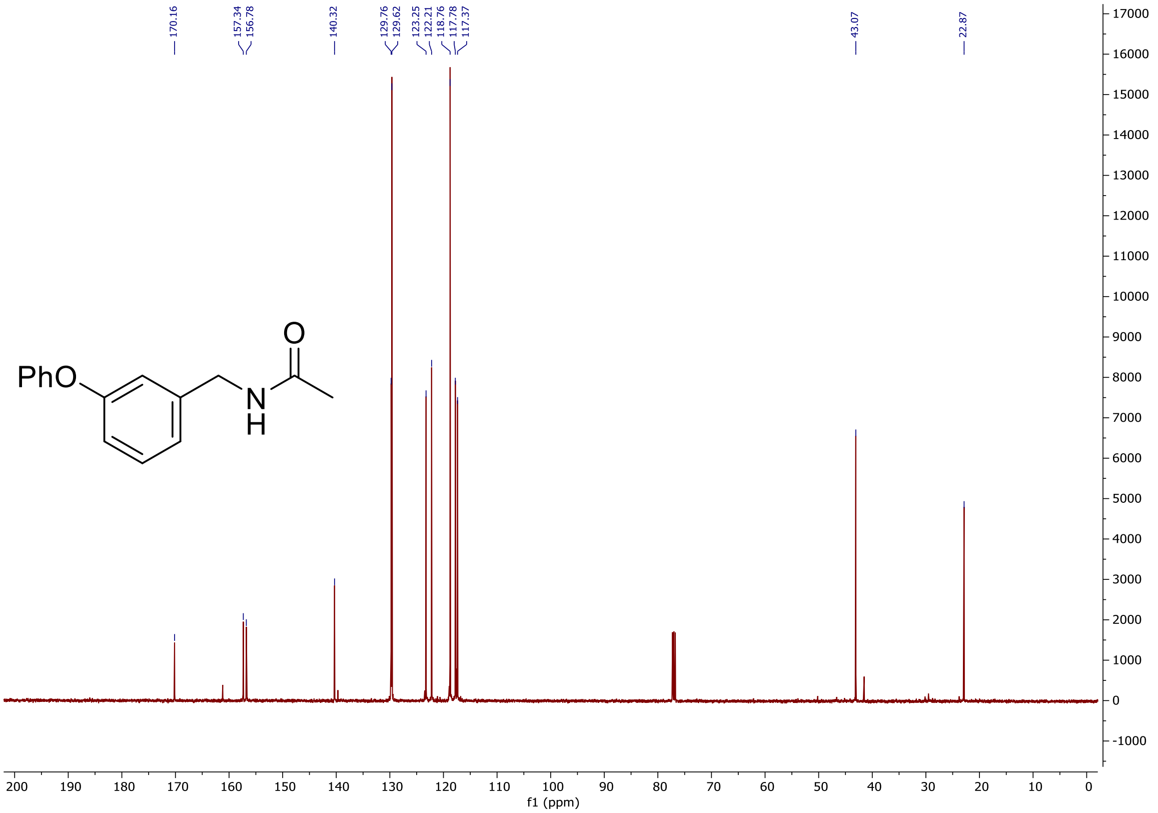

Supplementary Figure 73. ^{13}C NMR (126 MHz, $CDCl_3$) of compound **29a**

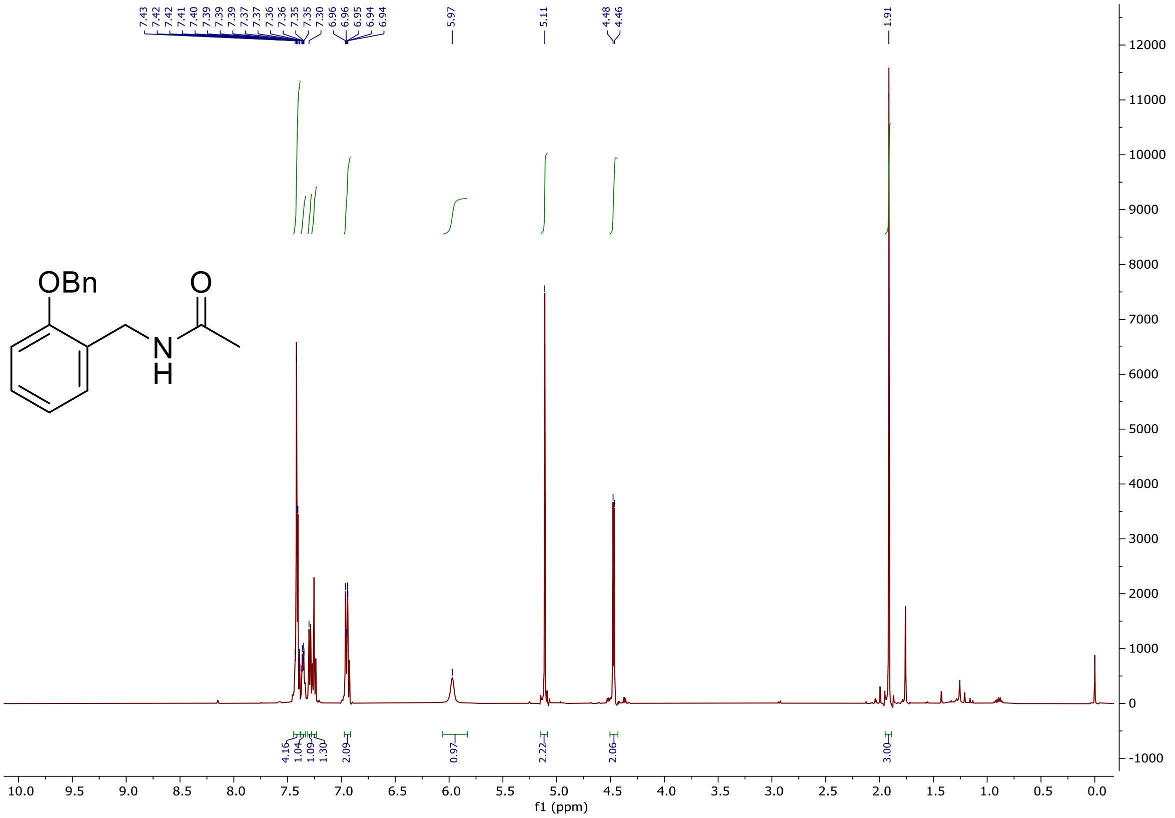

Supplementary Figure 74. ^{1}H NMR (400 MHz, $CDCl_3$) of compound **30a**

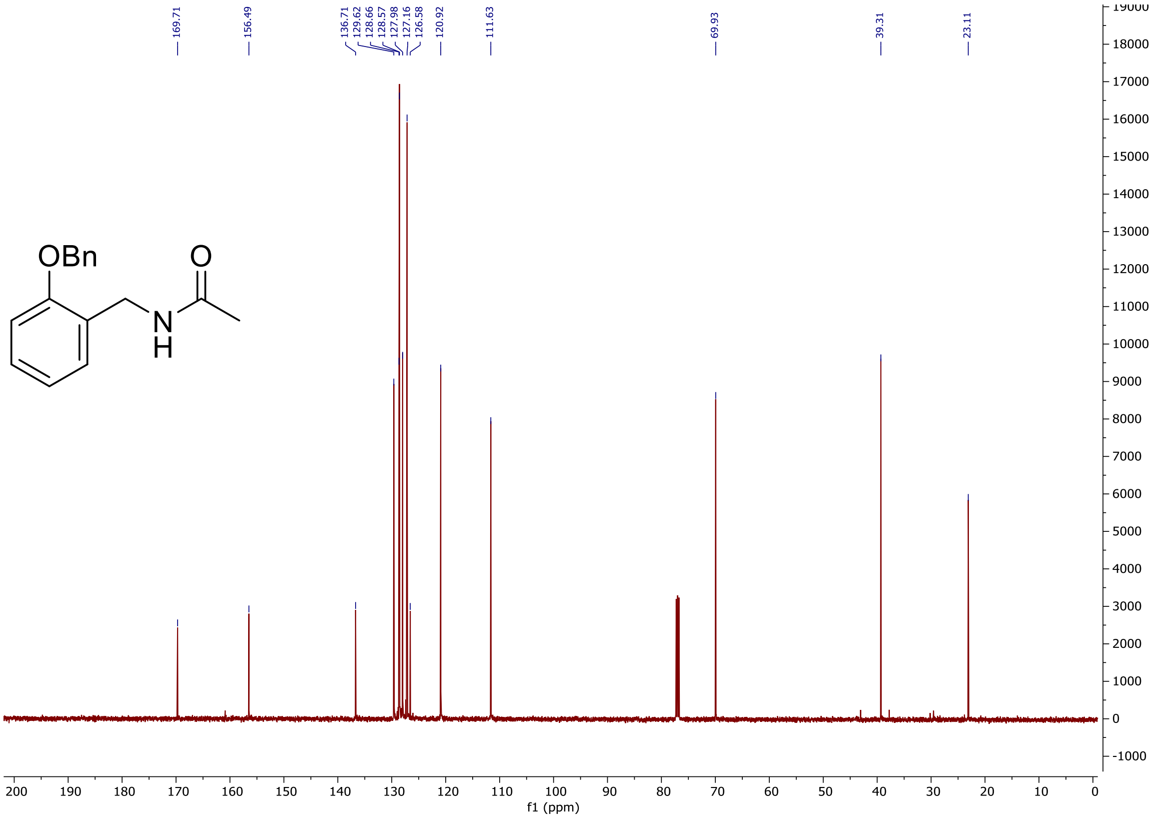

Supplementary Figure 75. ^{13}C NMR (126 MHz, $CDCl_3$) of compound **30a**

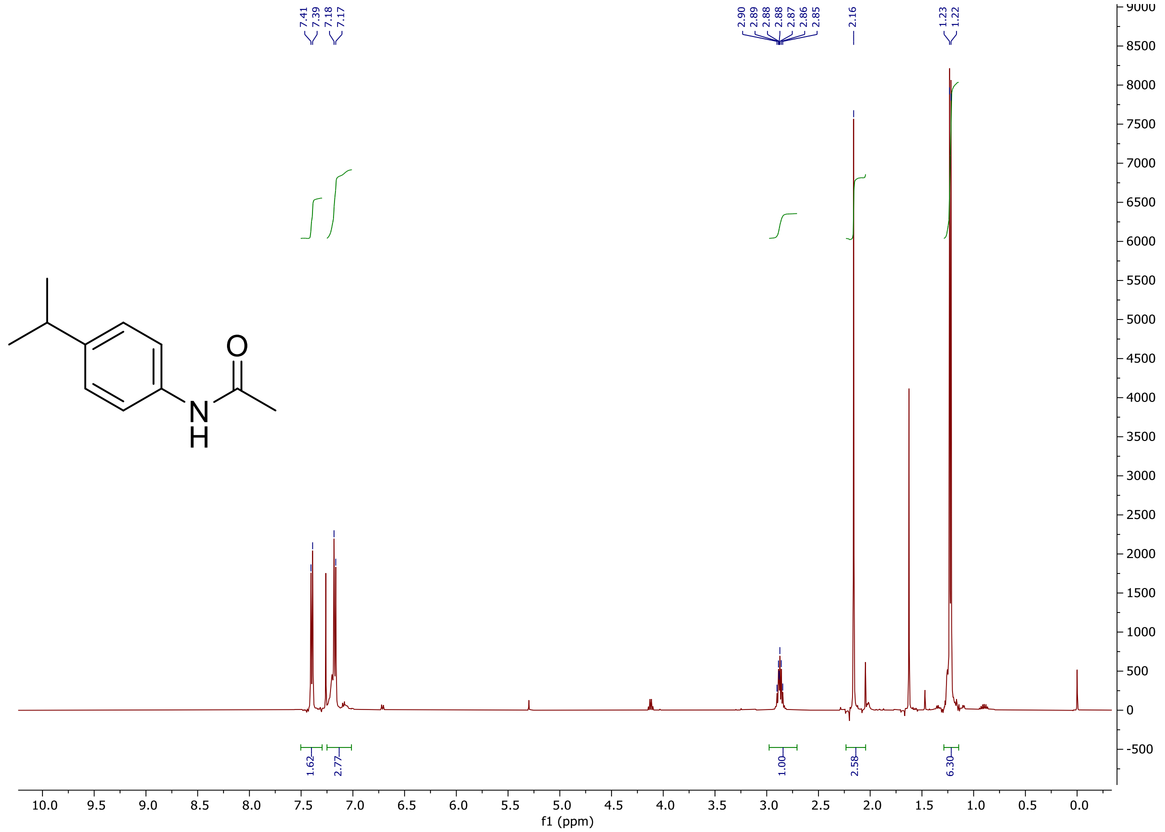

Supplementary Figure 76. ^{1}H NMR (400 MHz, $CDCl_3$) of compound **31a**

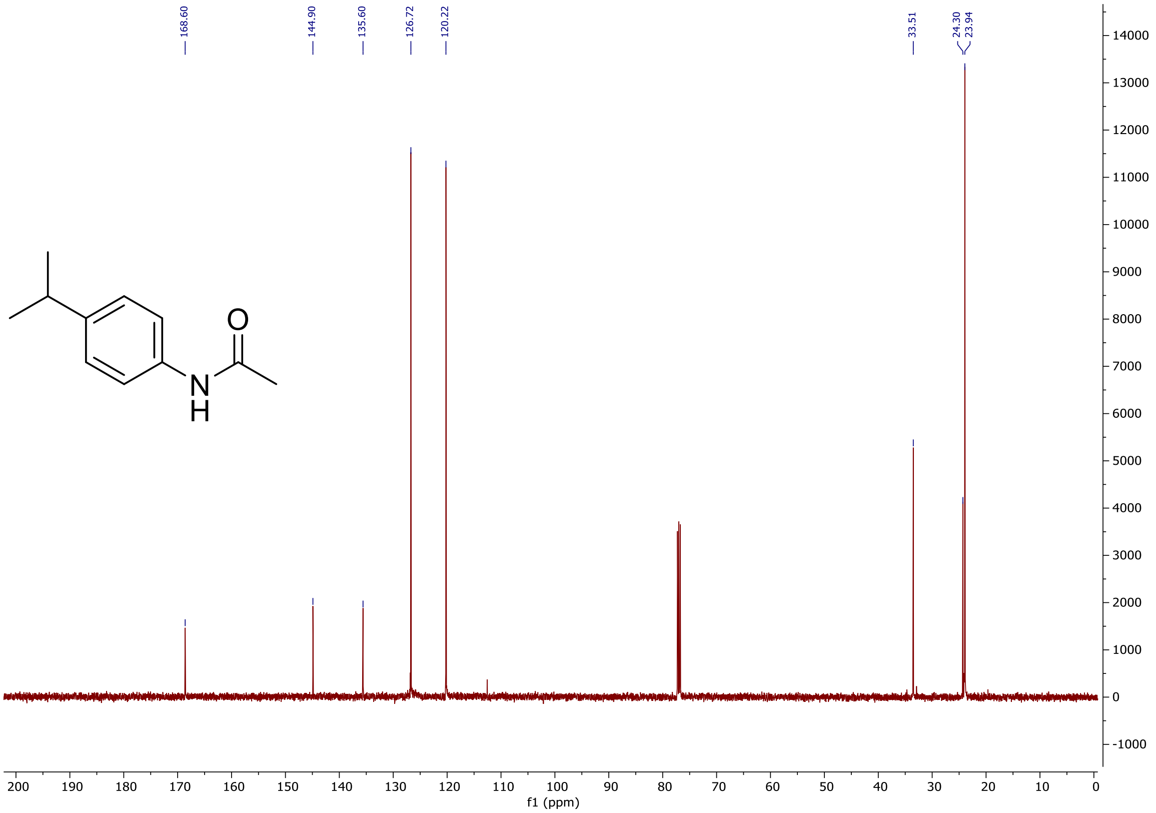

Supplementary Figure 77. ^{13}C NMR (126 MHz, $CDCl_3$) of compound **31a**

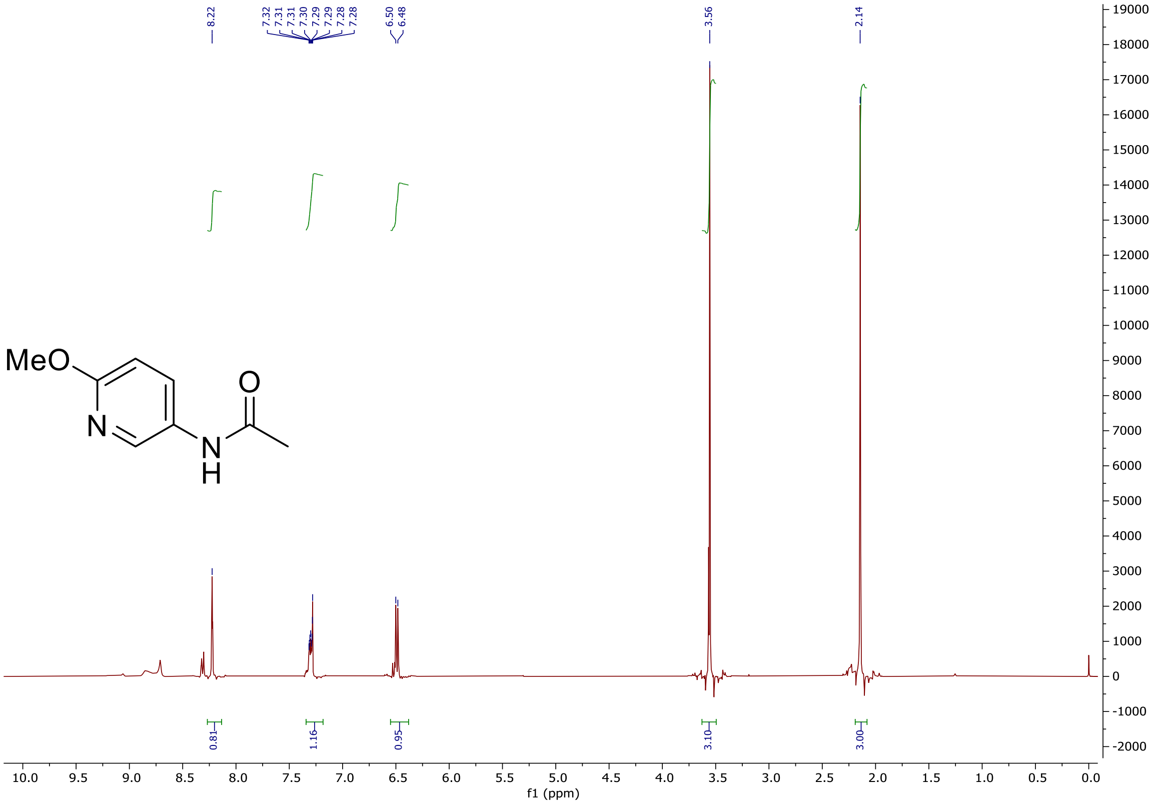

Supplementary Figure 78. ^{1}H NMR (400 MHz, $CDCl_3$) of compound **32a**

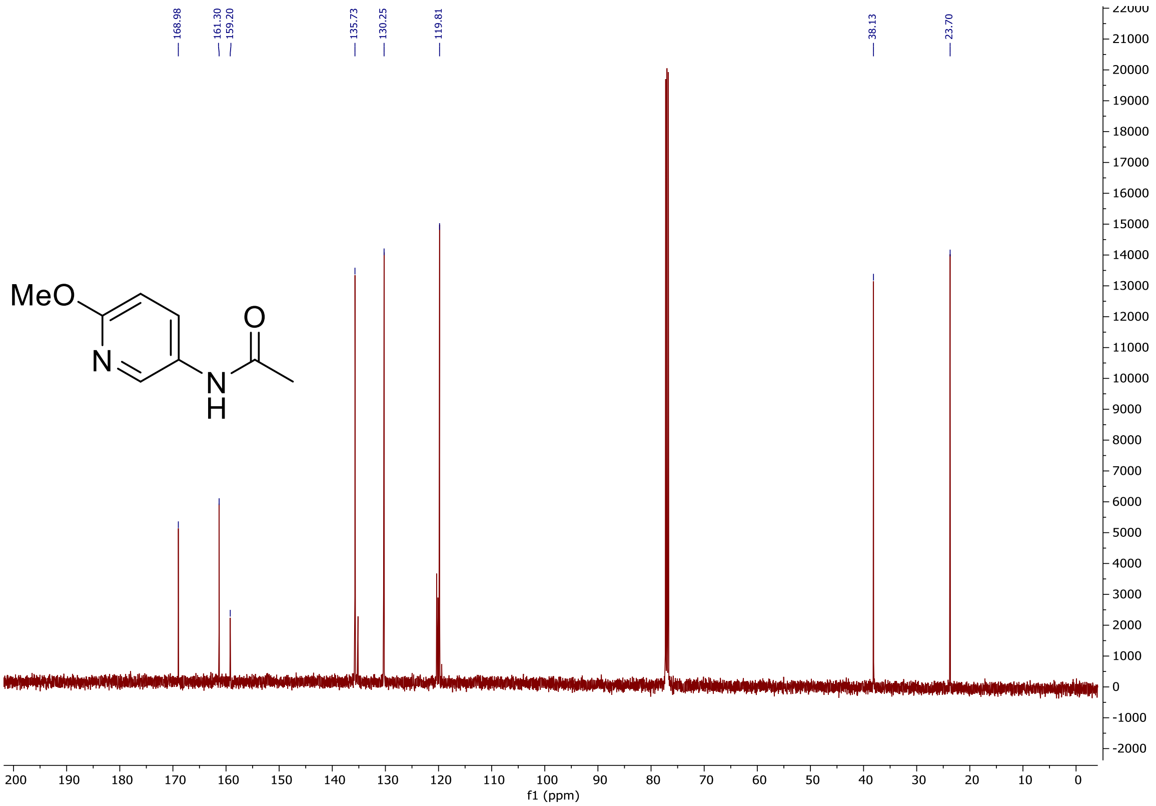

Supplementary Figure 79. ^{13}C NMR (126 MHz, $CDCl_3$) of compound **32a**

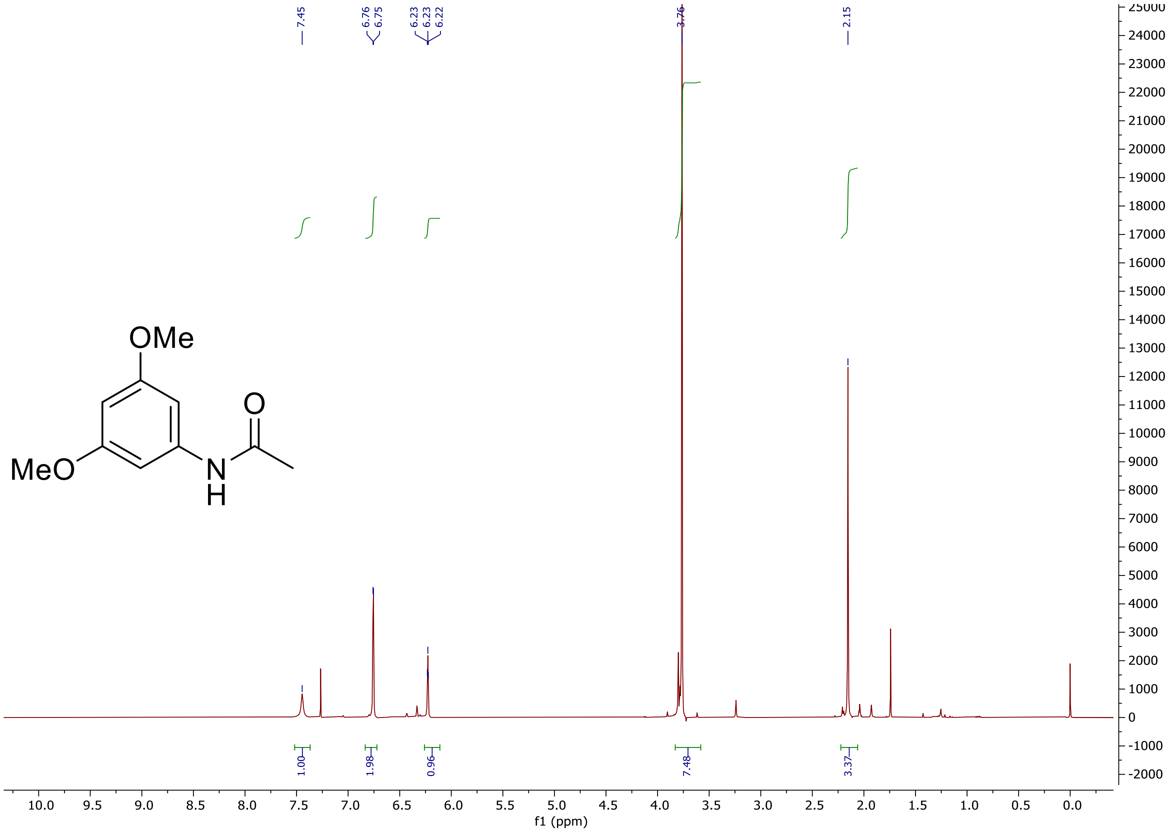

Supplementary Figure 80. ^{1}H NMR (400 MHz, $CDCl_3$) of compound **33a**

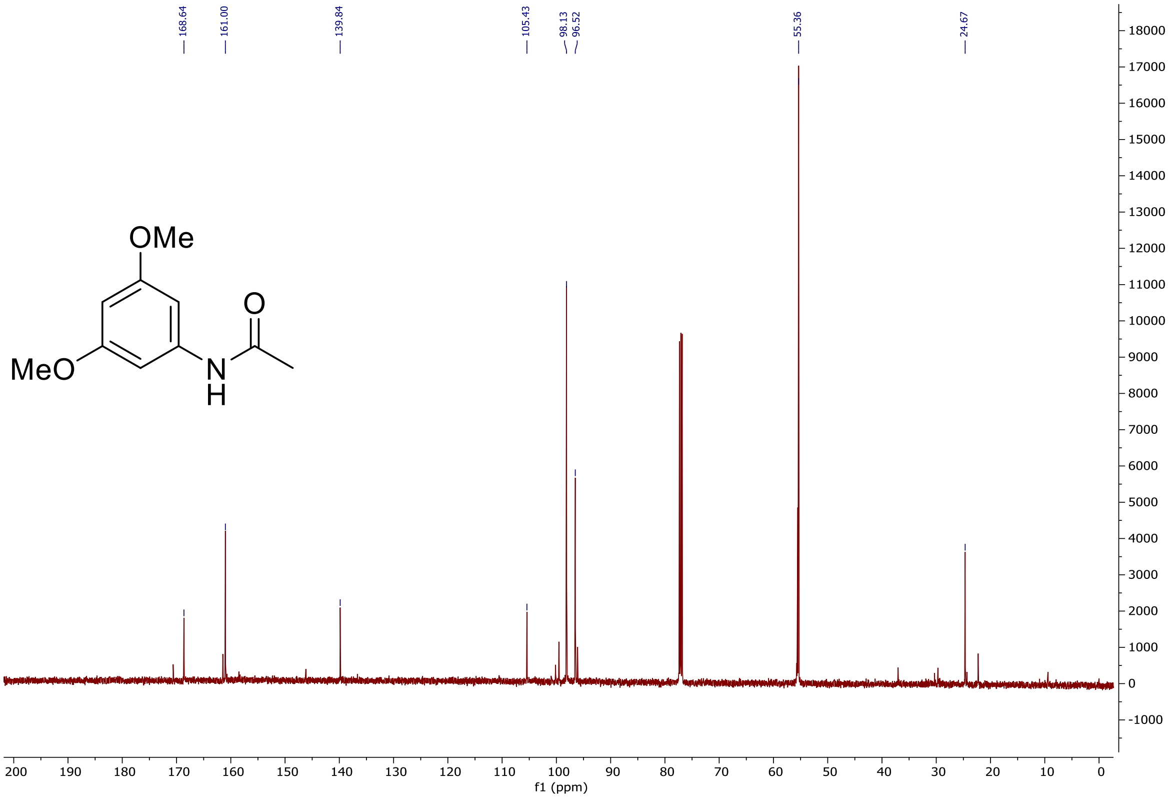

Supplementary Figure 81. ^{13}C NMR (126 MHz, $CDCl_3$) of compound **33a**

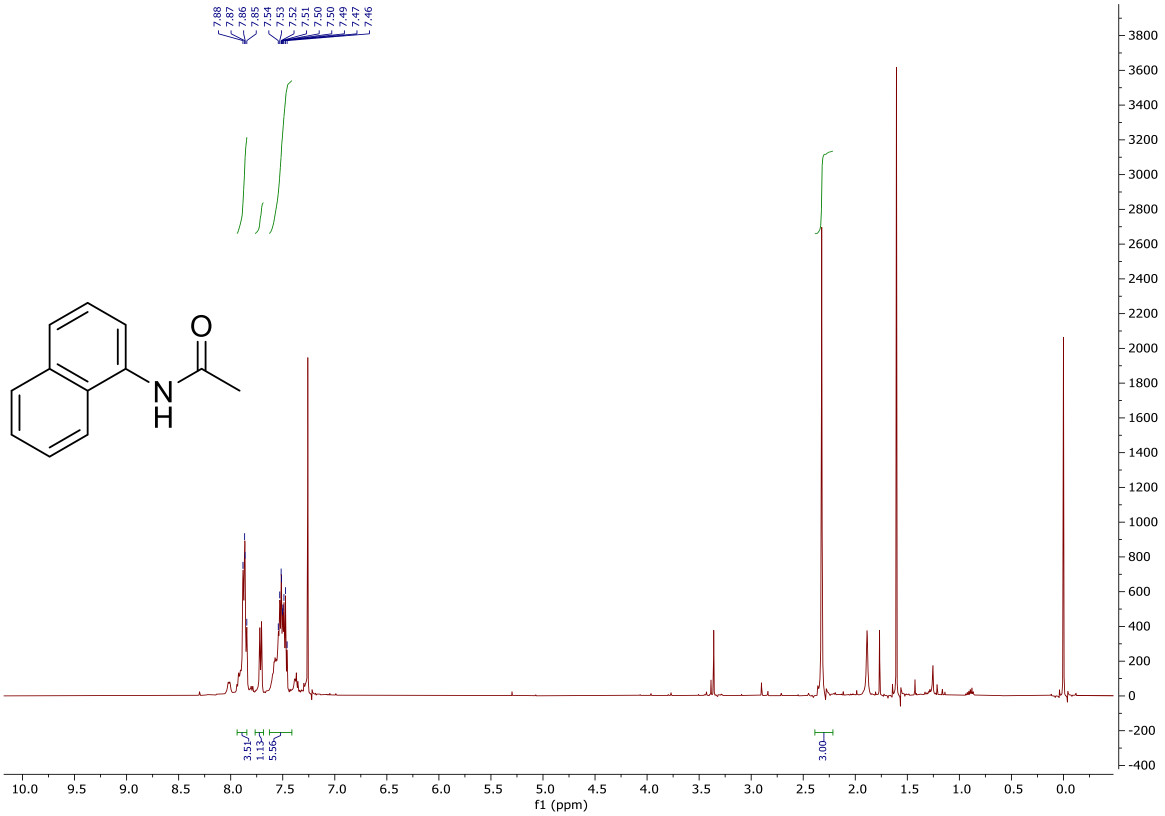

Supplementary Figure 82. ^{1}H NMR (400 MHz, $CDCl_3$) of compound **34a**

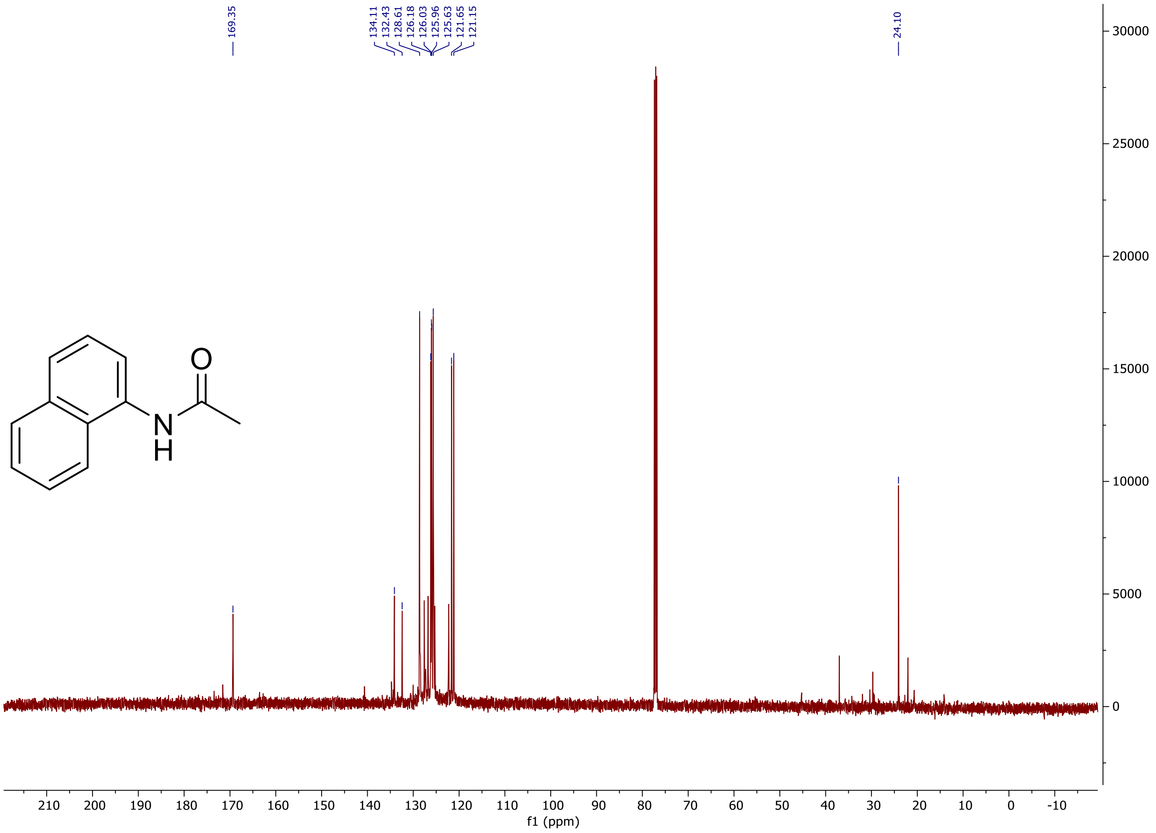

Supplementary Figure 83. ^{13}C NMR (126 MHz, $CDCl_3$) of compound **34a**

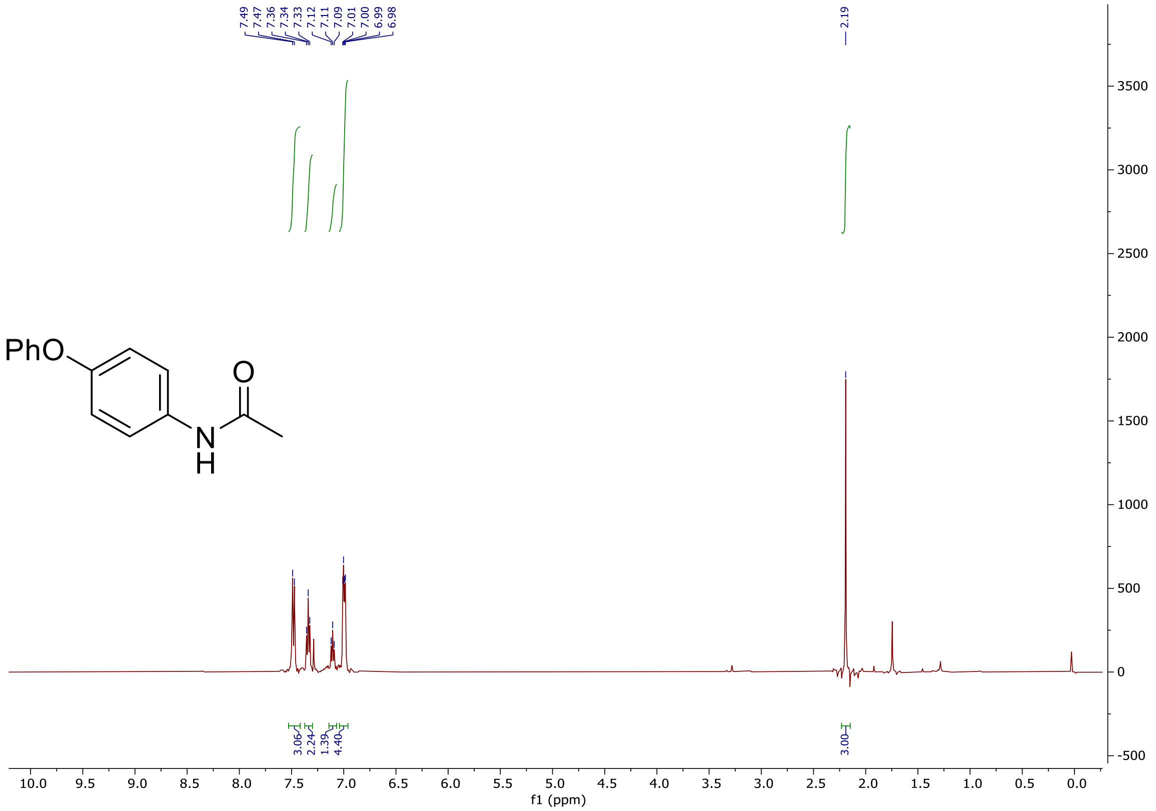

Supplementary Figure 84. ^{1}H NMR (400 MHz, $CDCl_3$) of compound **35a**

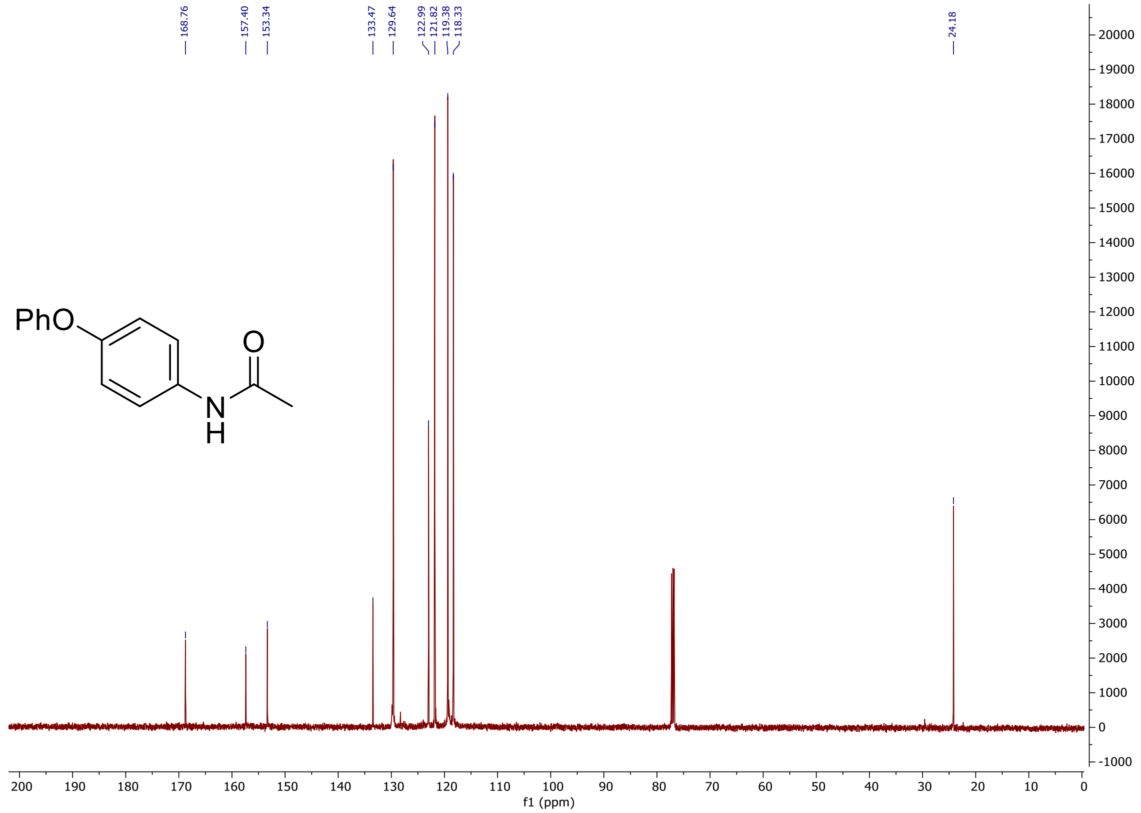

Supplementary Figure 85. ^{13}C NMR (126 MHz, $CDCl_3$) of compound **35a**

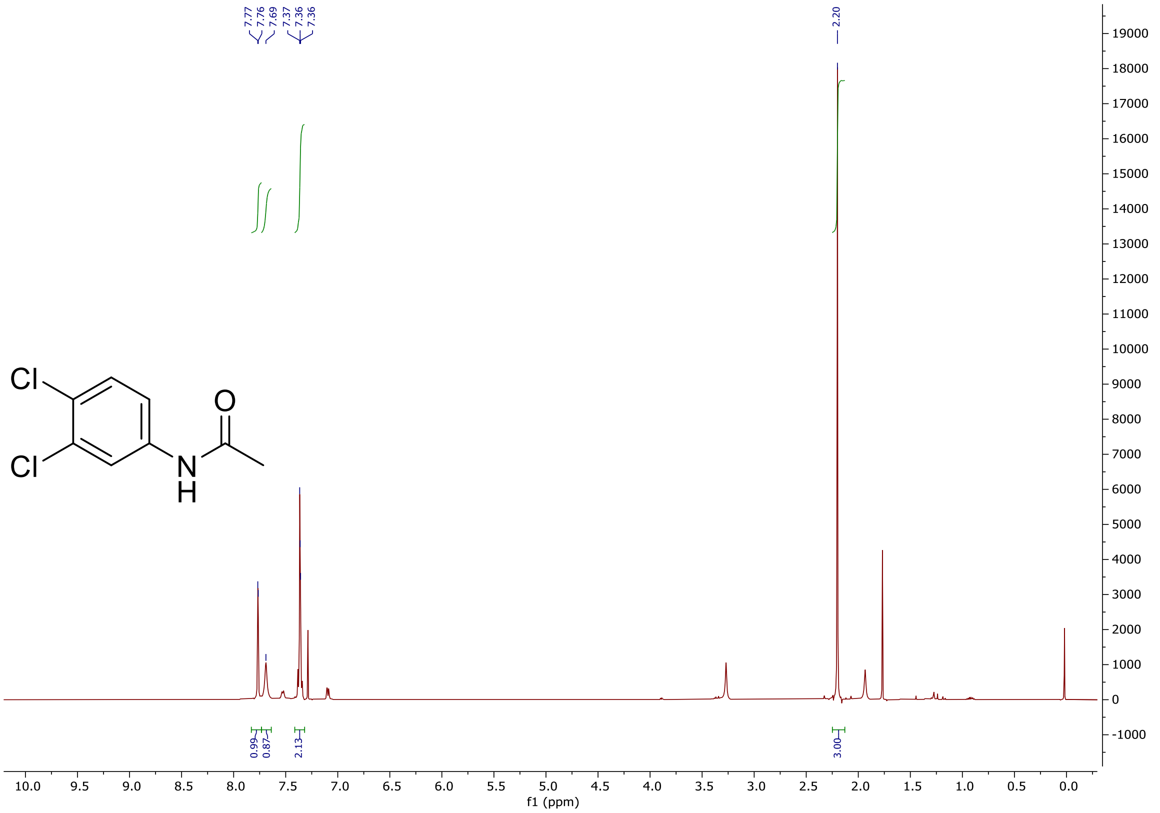

Supplementary Figure 86. ^{1}H NMR (400 MHz, $CDCl_3$) of compound **36a**

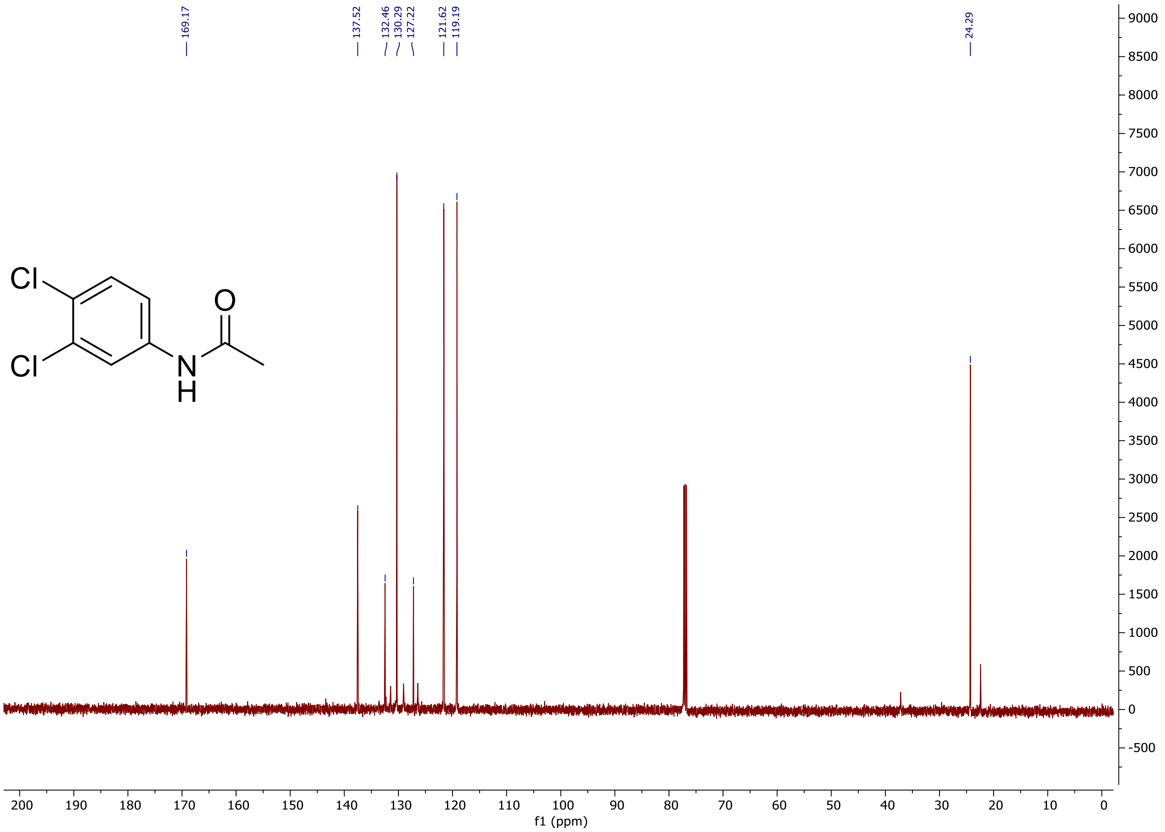

Supplementary Figure 87. ^{13}C NMR (126 MHz, $CDCl_3$) of compound **36a**

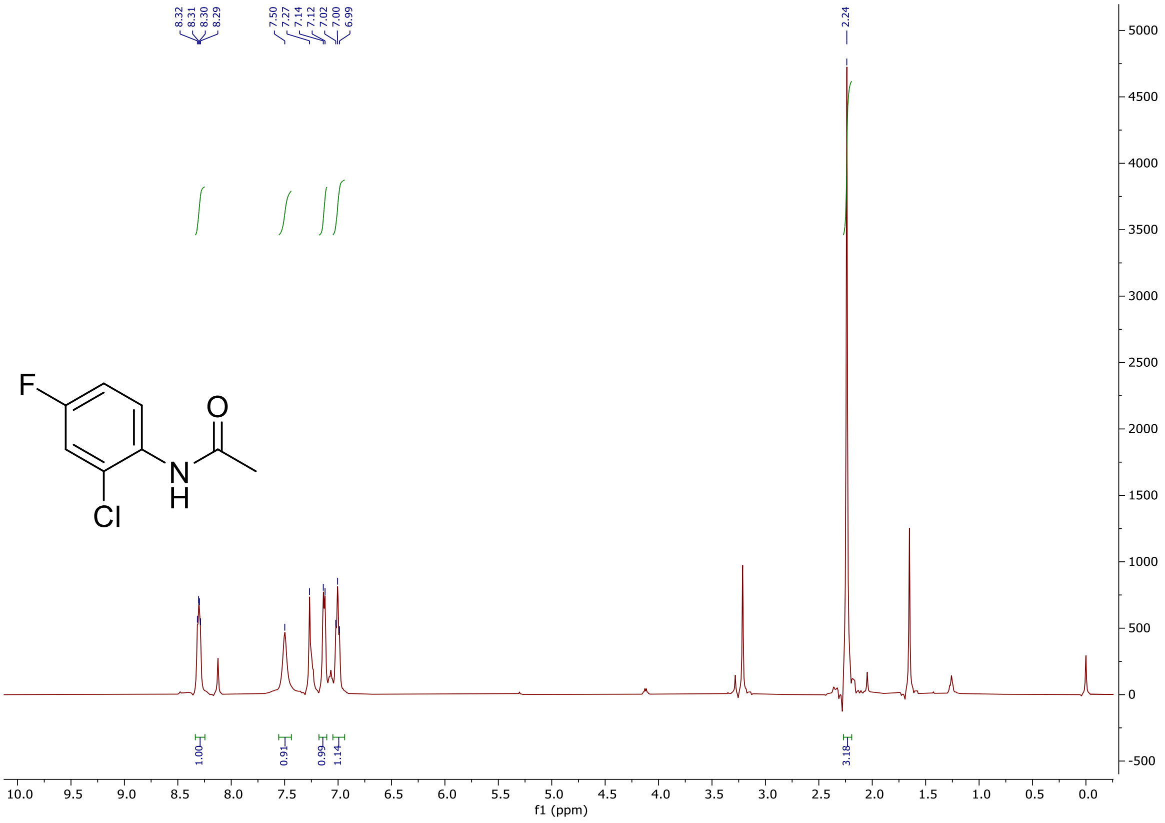

Supplementary Figure 88. ^{1}H NMR (400 MHz, $CDCl_3$) of compound **37a**

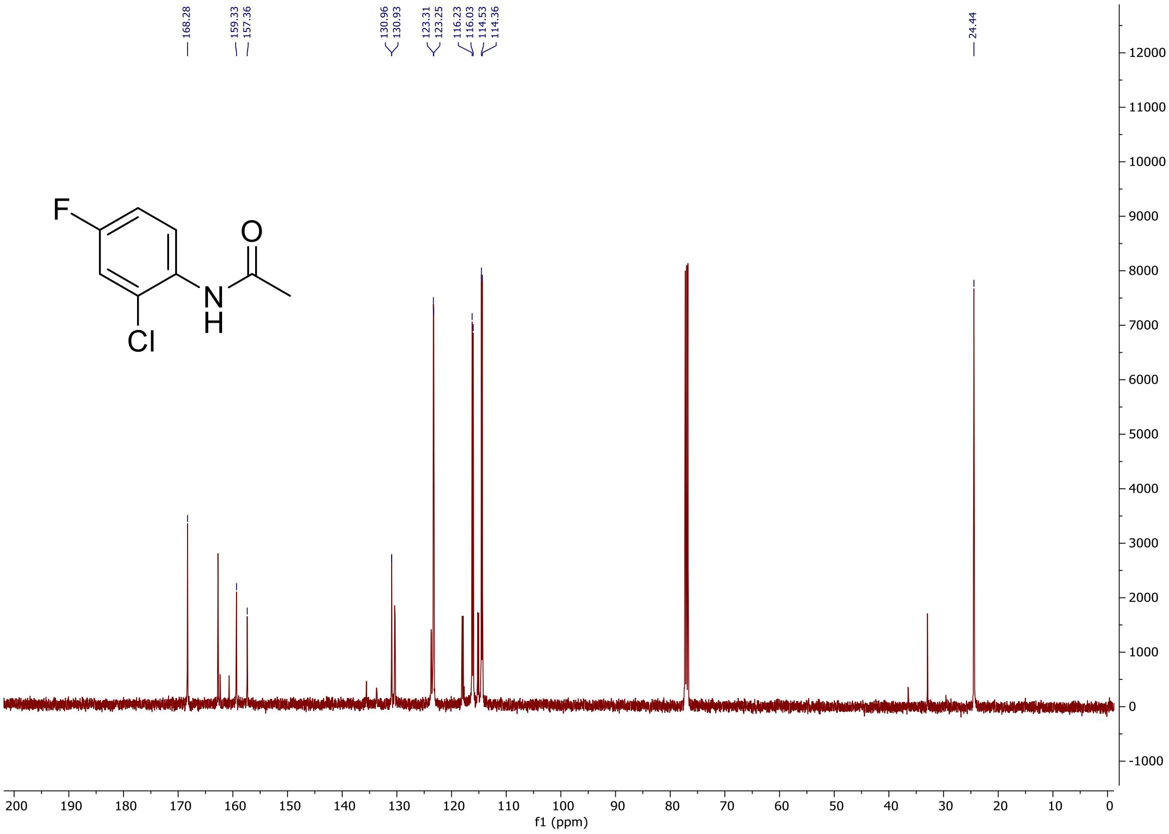

Supplementary Figure 89. ^{13}C NMR (126 MHz, $CDCl_3$) of compound **37a**

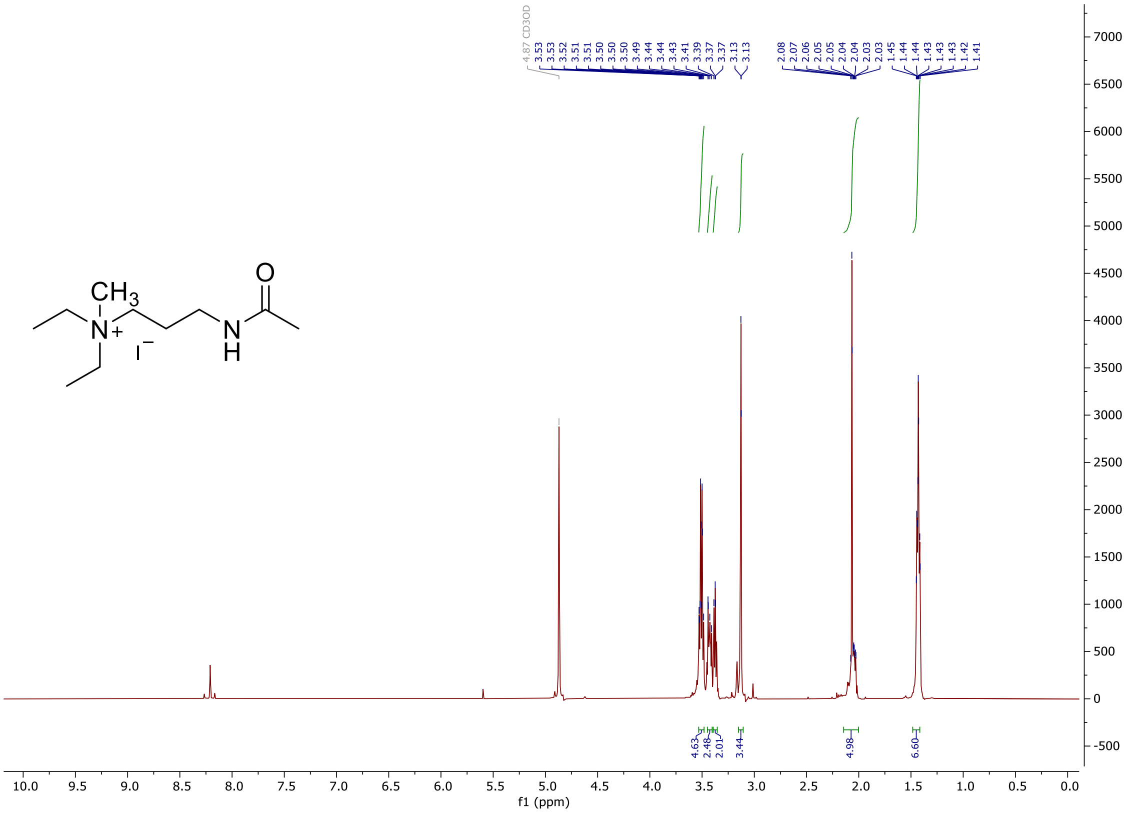

Supplementary Figure 90. ^{1}H NMR (400 MHz, MeOD) of compound **38a**

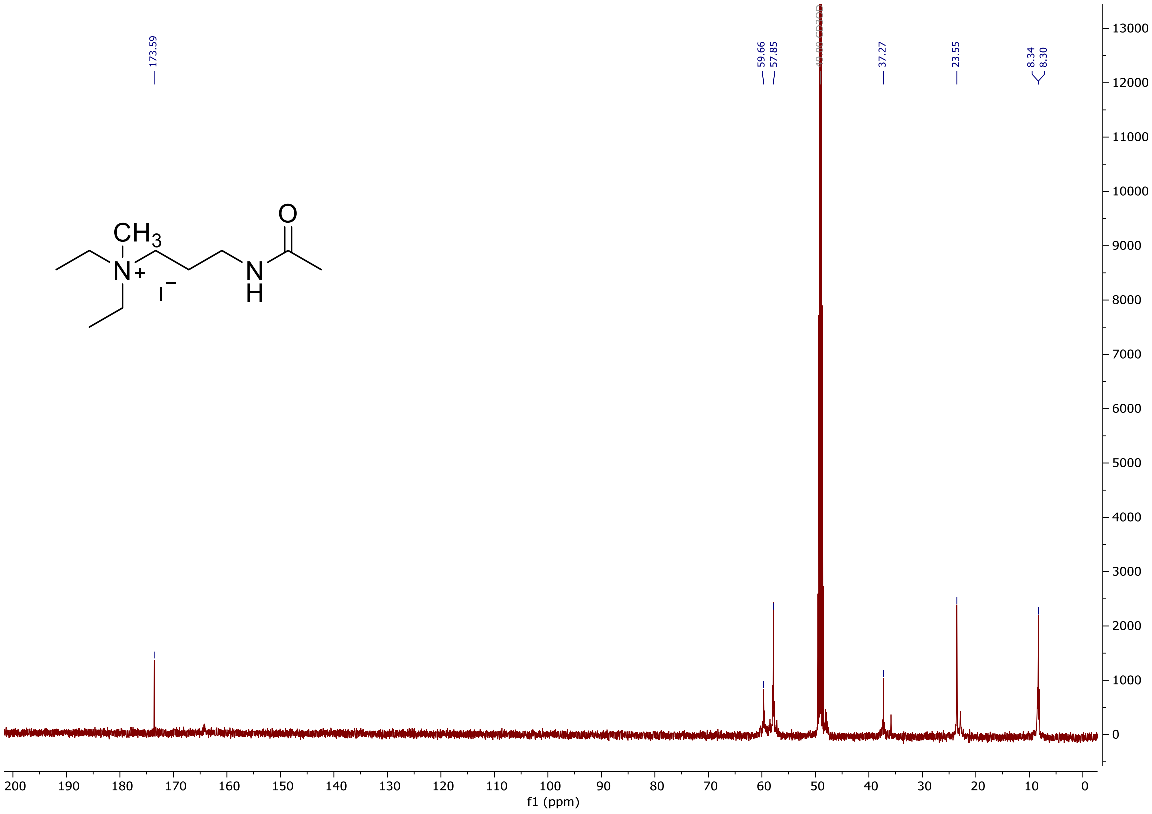

Supplementary Figure 91. ^{13}C NMR (126 MHz, MeOD) of compound **38a**

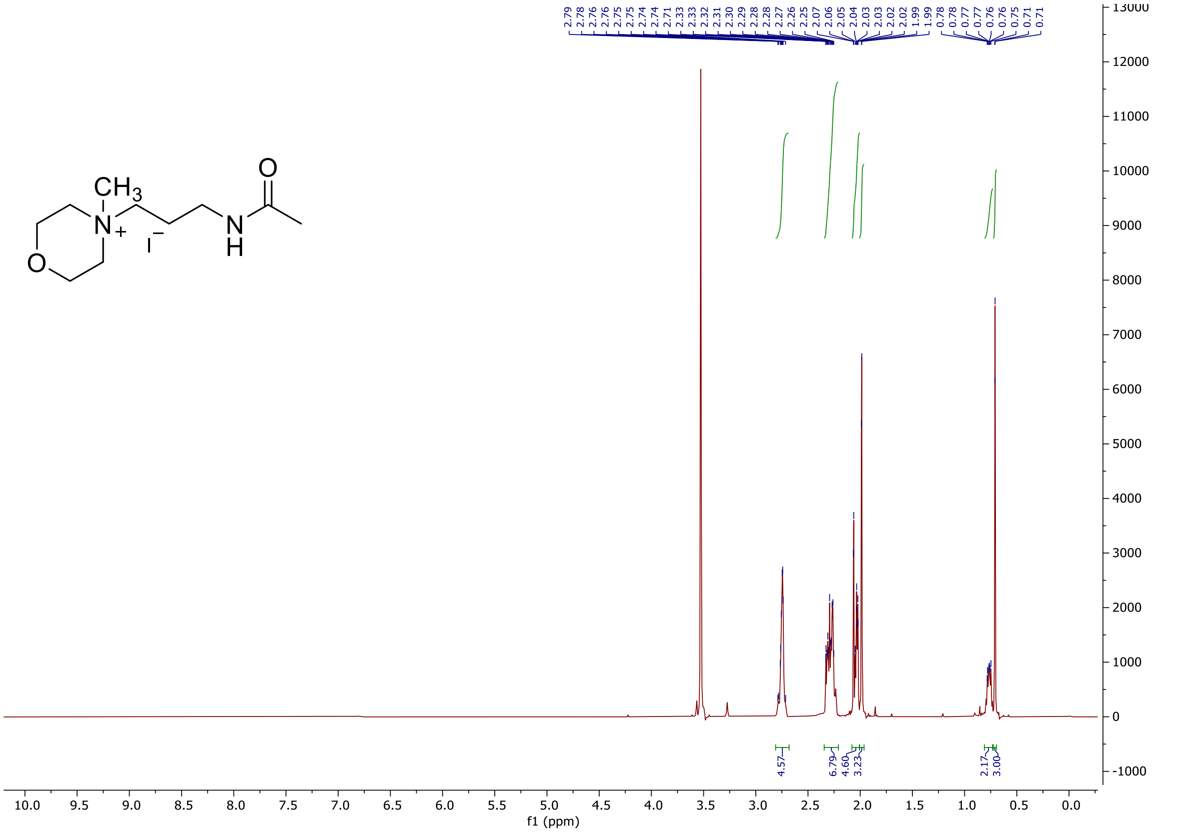

Supplementary Figure 92. ^{1}H NMR (400 MHz, MeOD) of compound **39a**

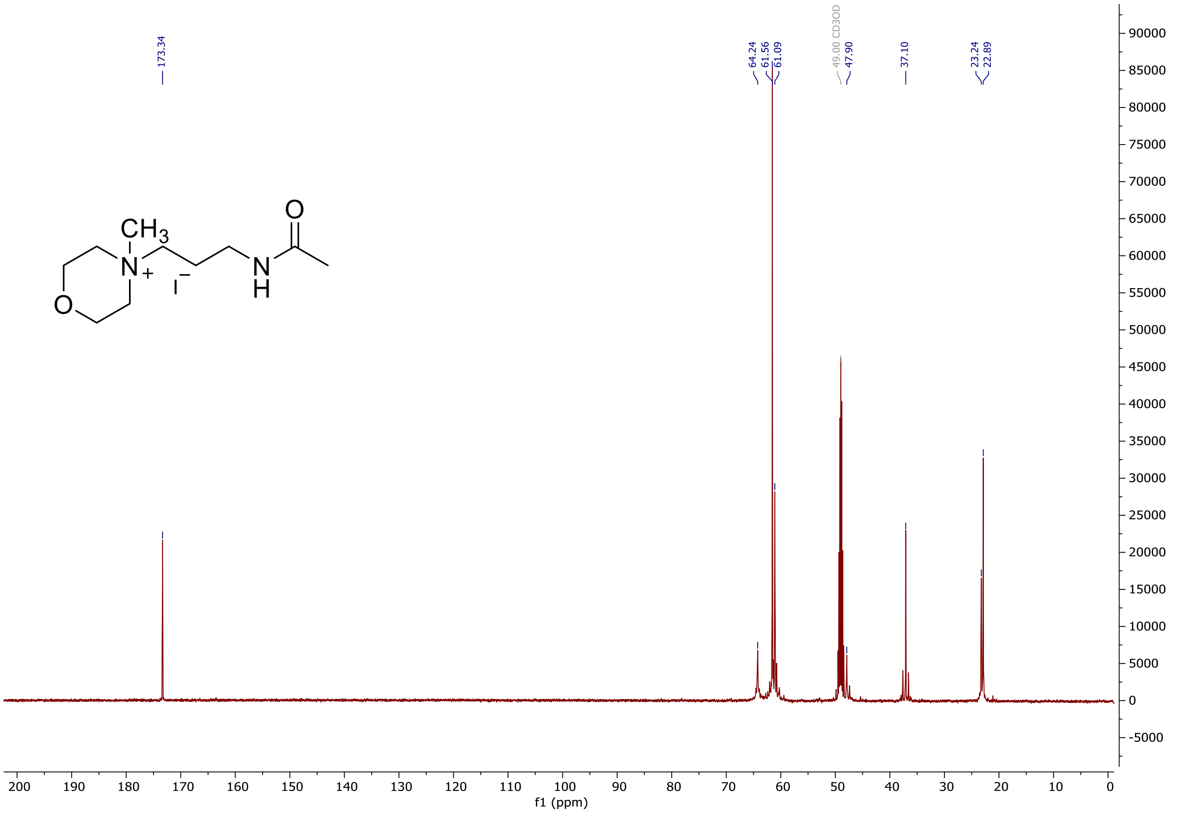

Supplementary Figure 93. ^{13}C NMR (126 MHz, MeOD) of compound **39a**

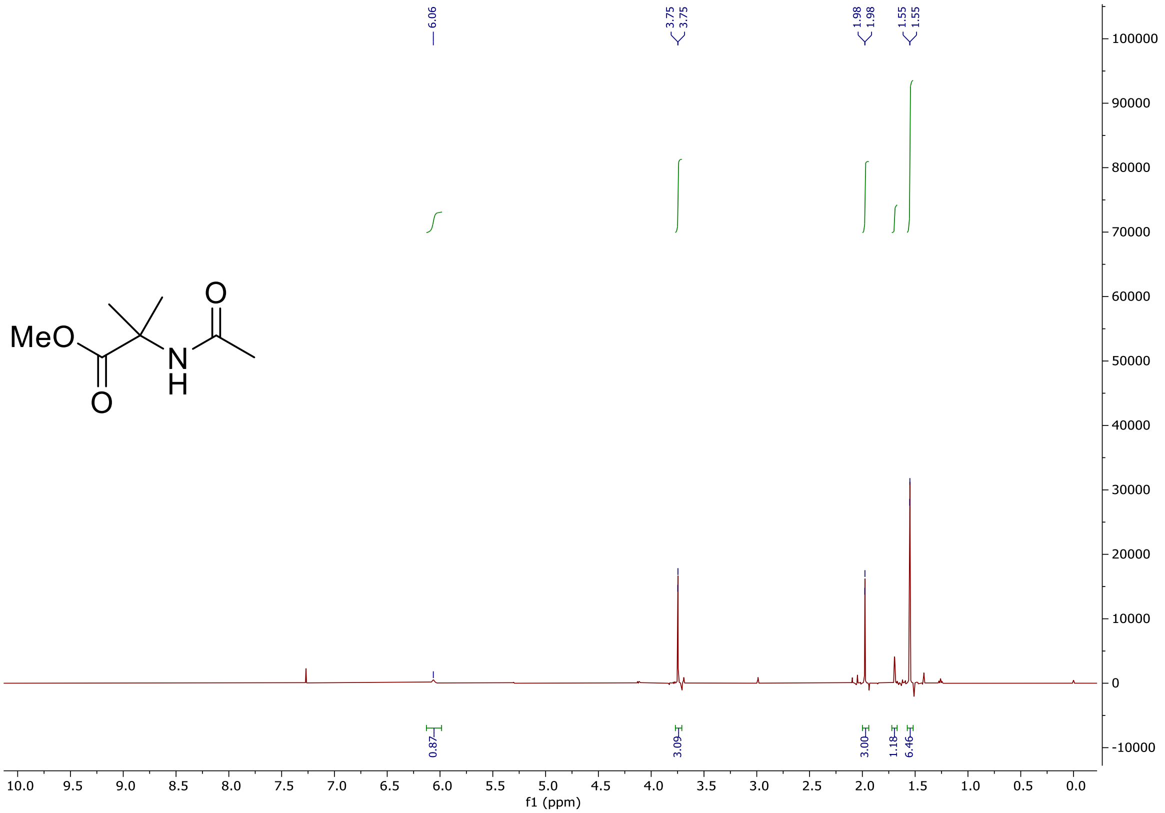

Supplementary Figure 94. ^{1}H NMR (400 MHz, $CDCl_3$) of compound **40a**

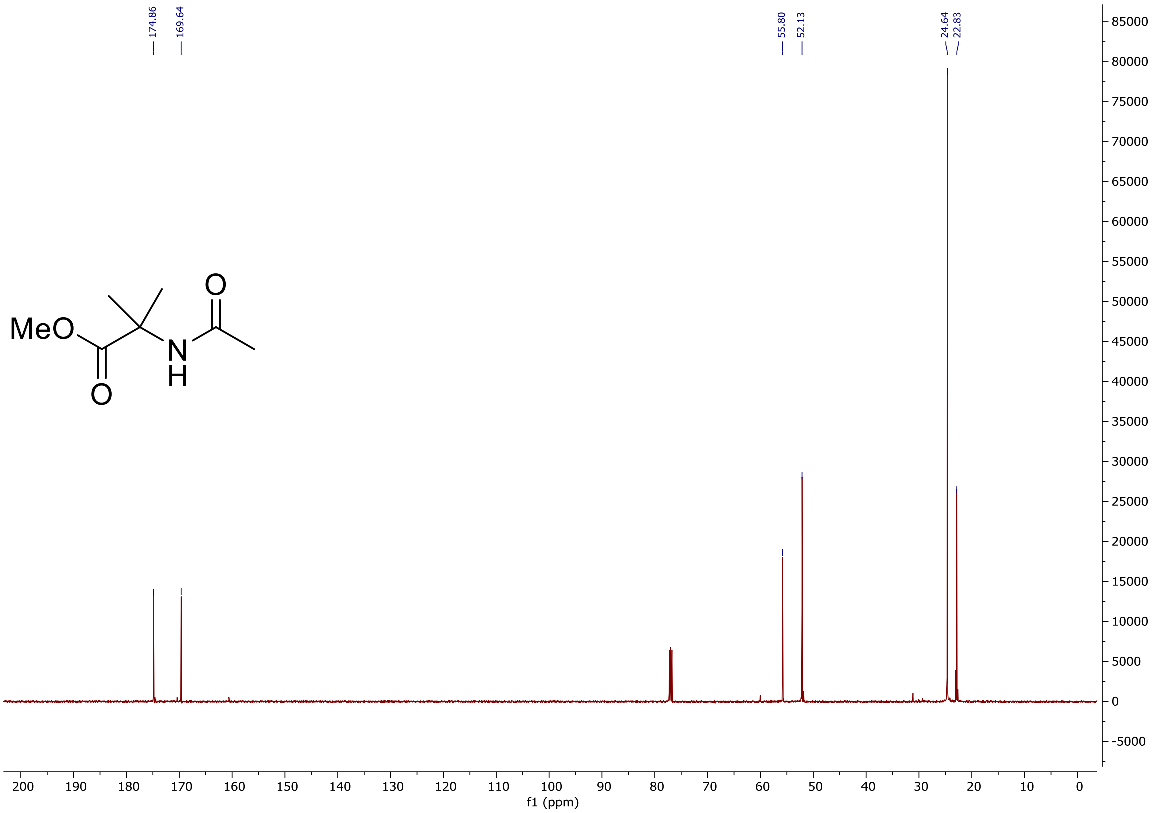

Supplementary Figure 95. ^{13}C NMR (126 MHz, $CDCl_3$) of compound **40a**

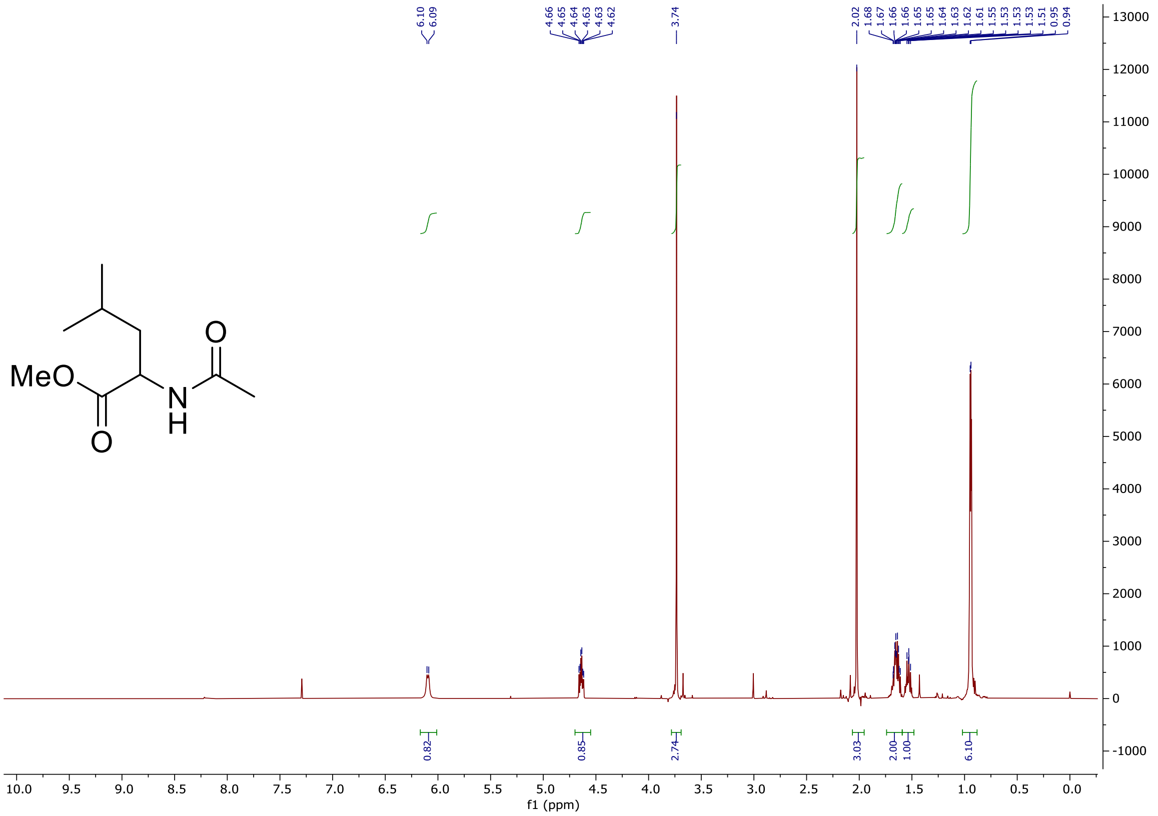

Supplementary Figure 96. ^{1}H NMR (400 MHz, $CDCl_3$) of compound **41a**

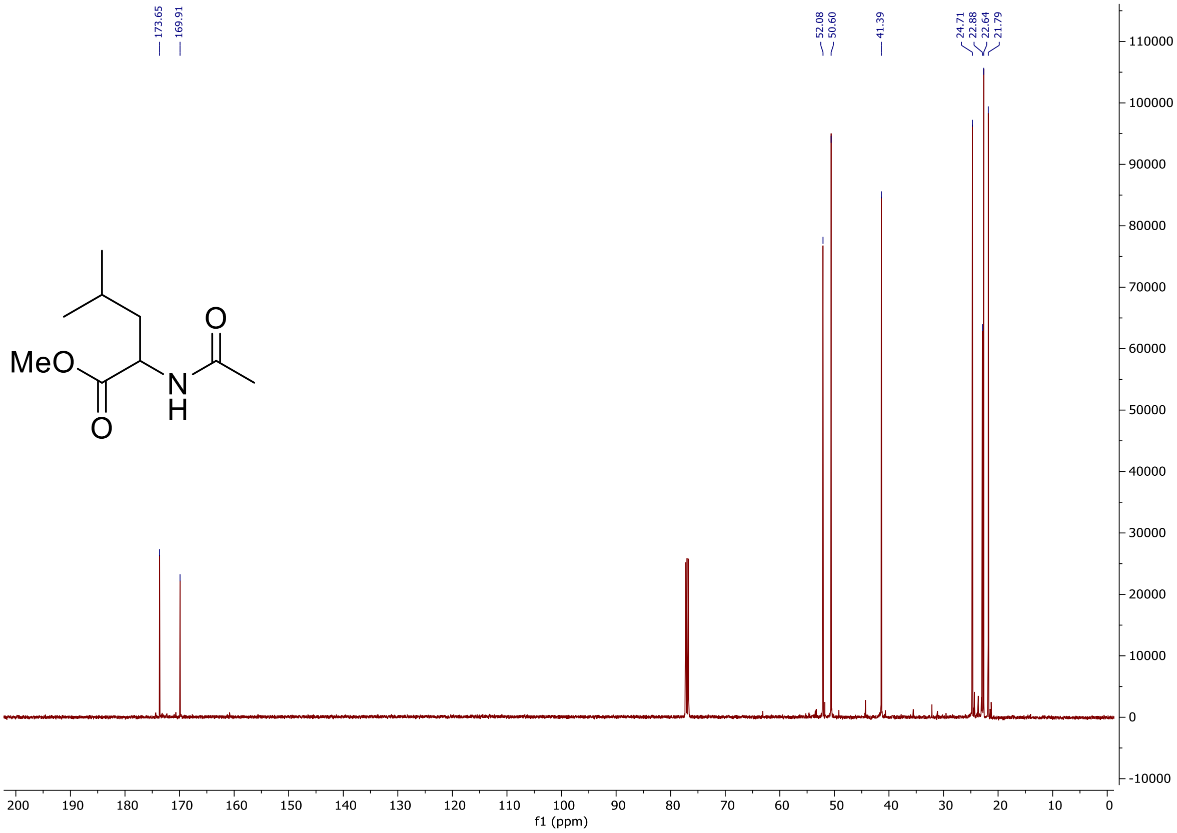

Supplementary Figure 97. ^{13}C NMR (126 MHz, CDCl3) of compound **41a**

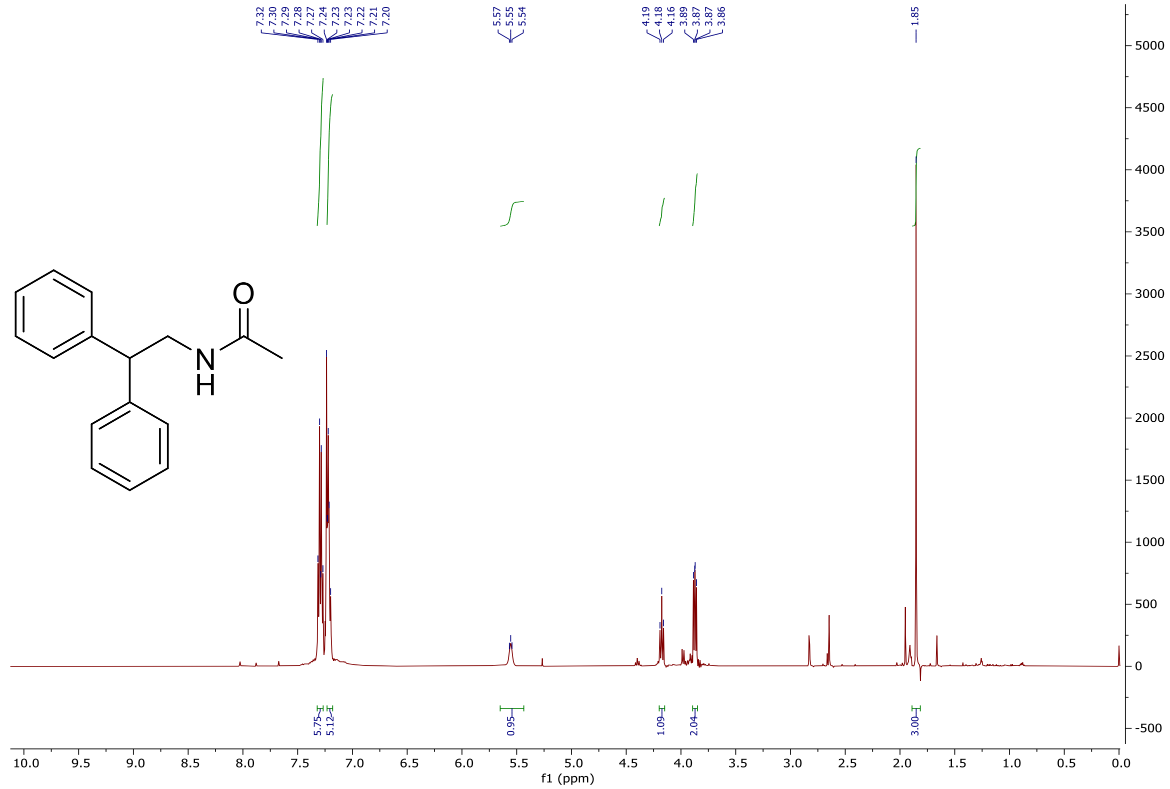

Supplementary Figure 98. ^{1}H NMR (400 MHz, $CDCl_3$) of compound **42a:**

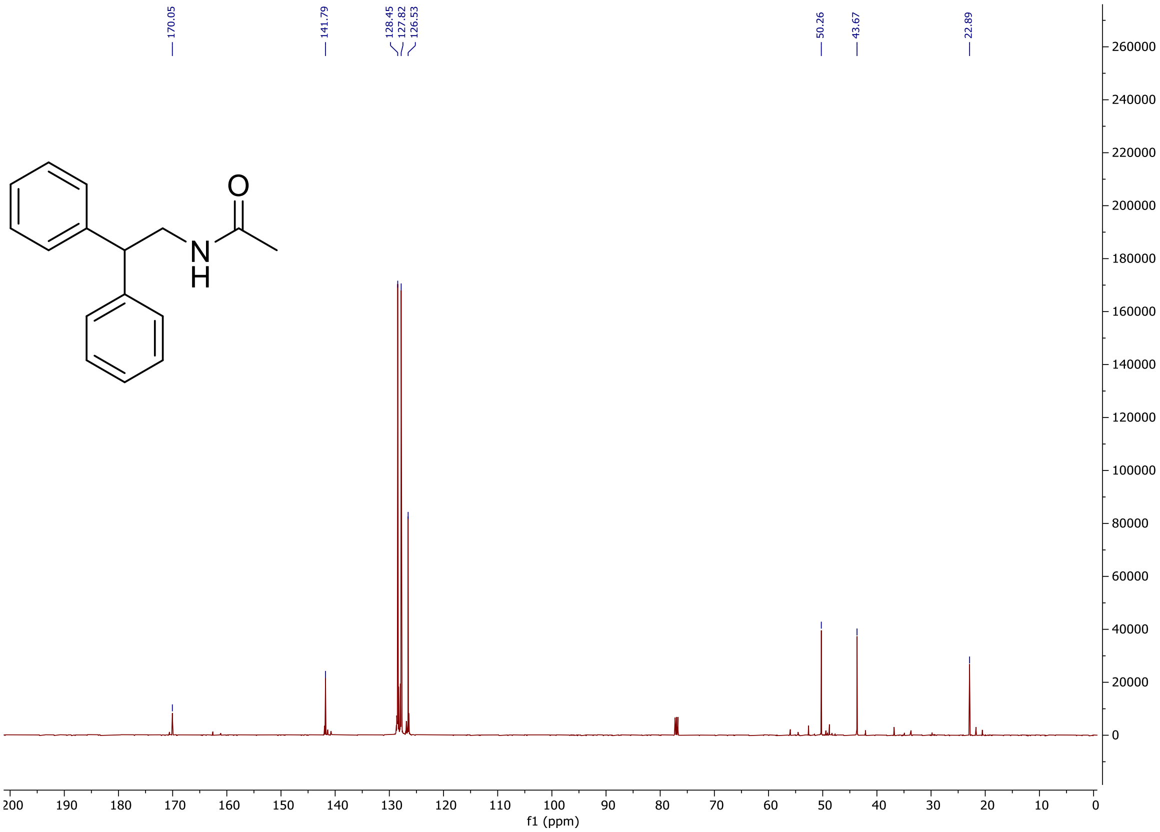

Supplementary Figure 99. ^{13}C NMR (126 MHz, $CDCl_3$) of compound **42a**

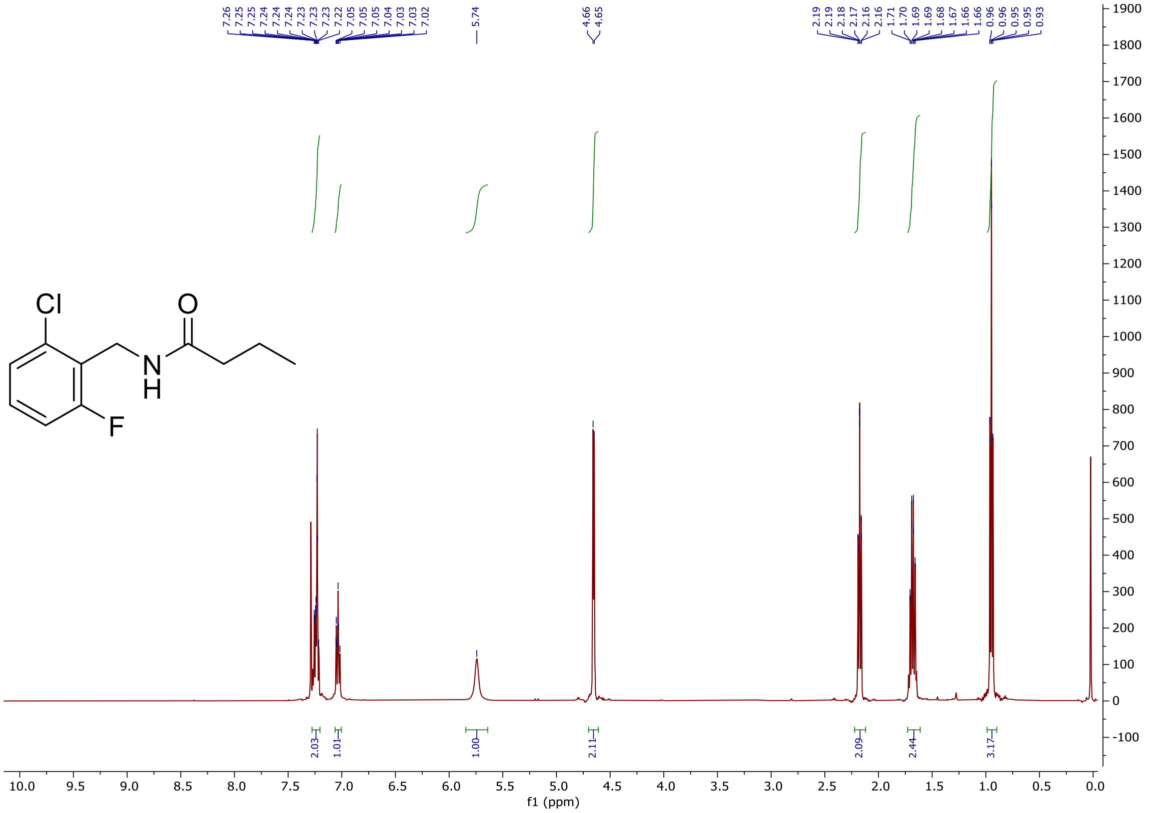

Supplementary Figure 100. ^{1}H NMR (400 MHz, $CDCl_3$) of compound **43a:**

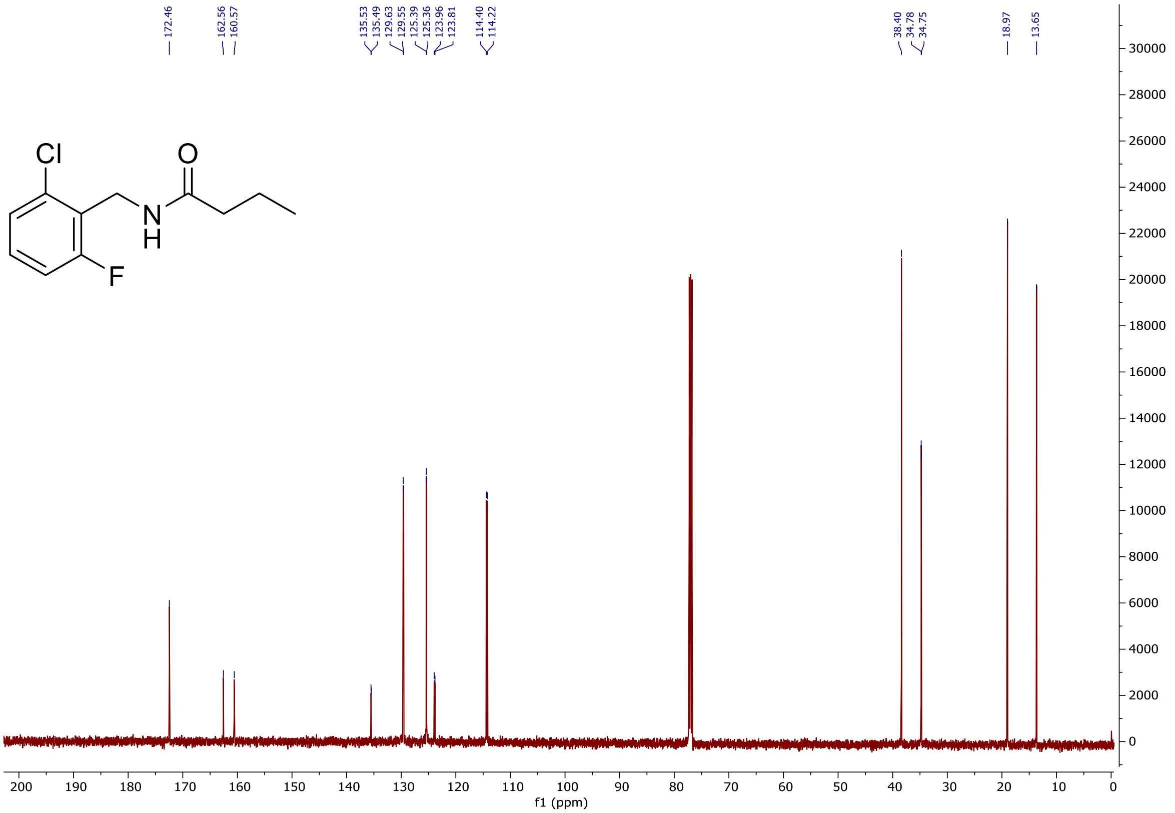

Supplementary Figure 101. ^{13}C NMR (126 MHz, $CDCl_3$) of compound **43a**

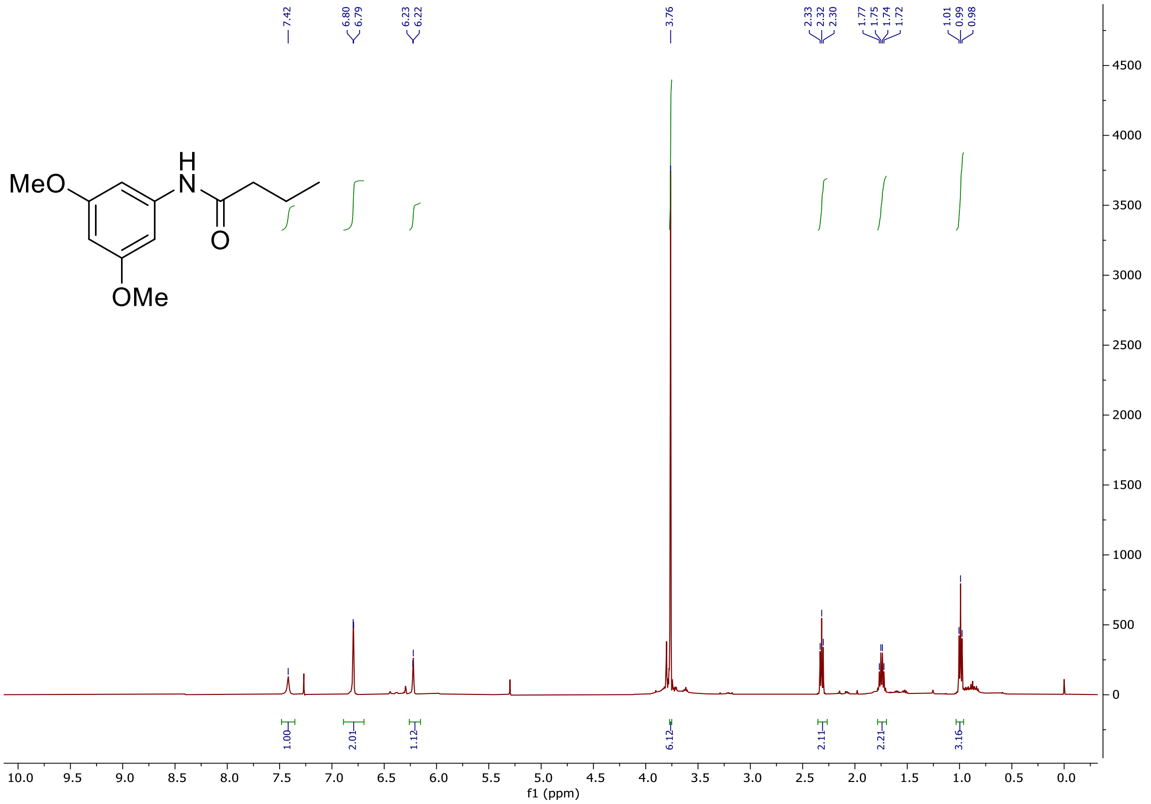

Supplementary Figure 102. ^{1}H NMR (400 MHz, $CDCl_3$) of compound **44a:**

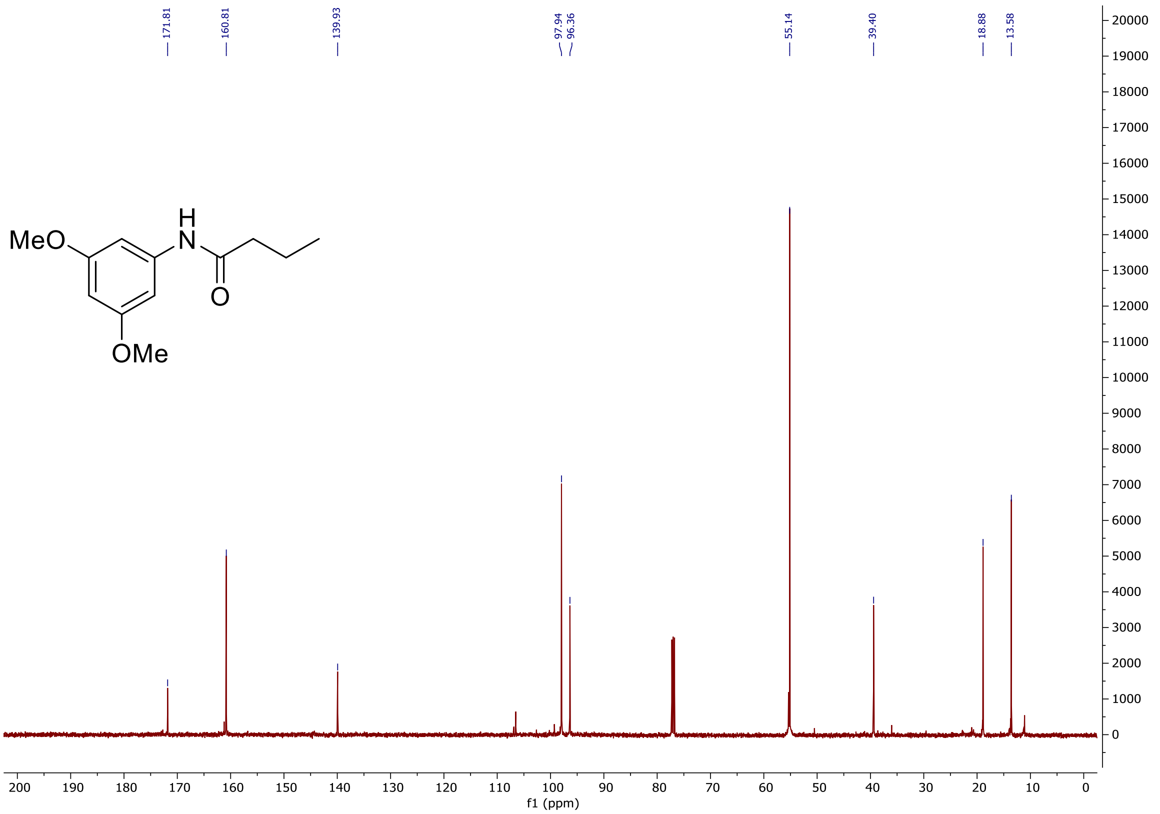

Supplementary Figure 103. ^{13}C NMR (126 MHz, $CDCl_3$) of compound **44a**

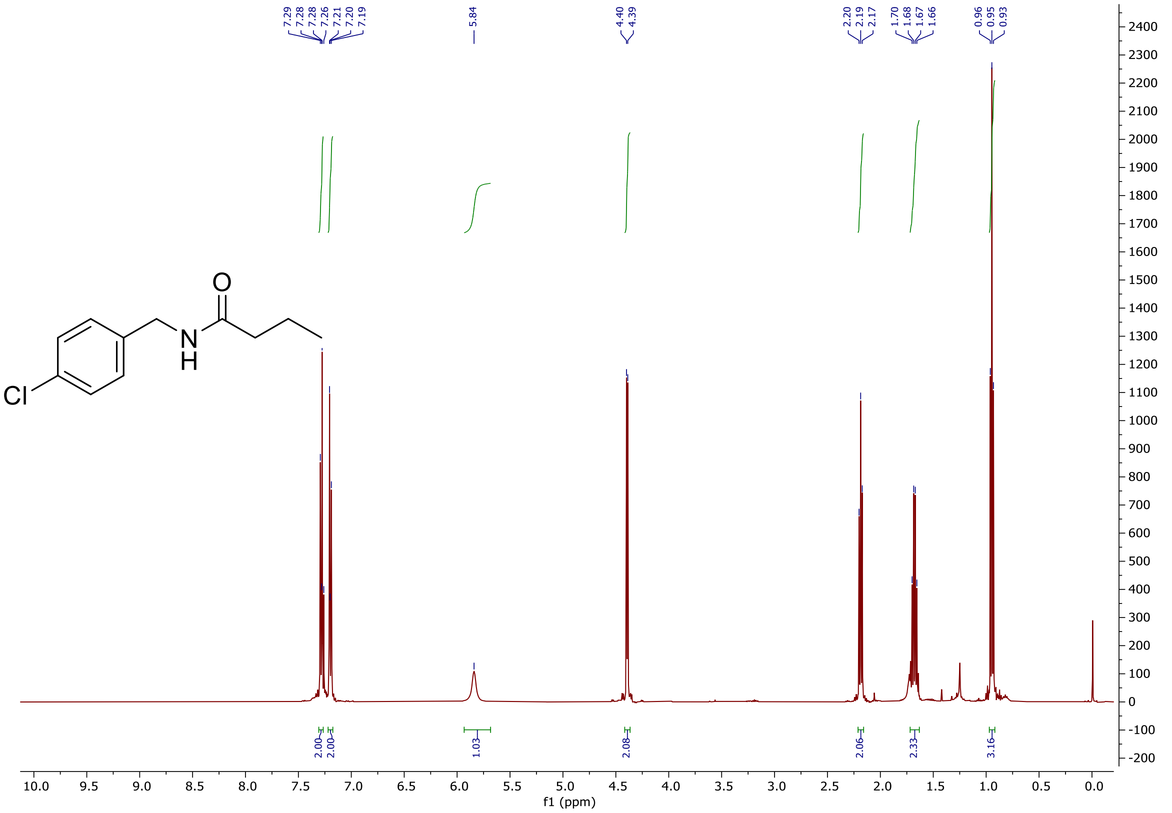

Supplementary Figure 104. ^{1}H NMR (400 MHz, $CDCl_3$) of compound **45a**

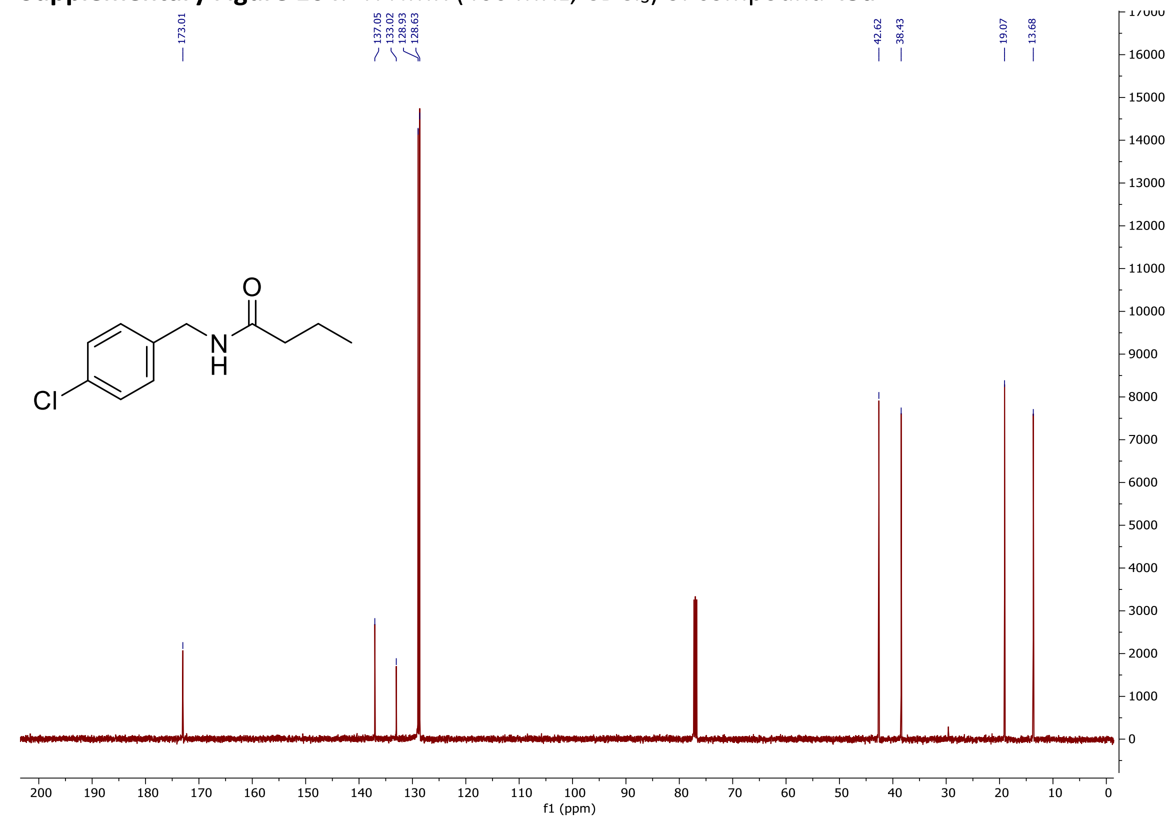

Supplementary Figure 105. ^{13}C NMR (126 MHz, $CDCl_3$) of compound **45a**

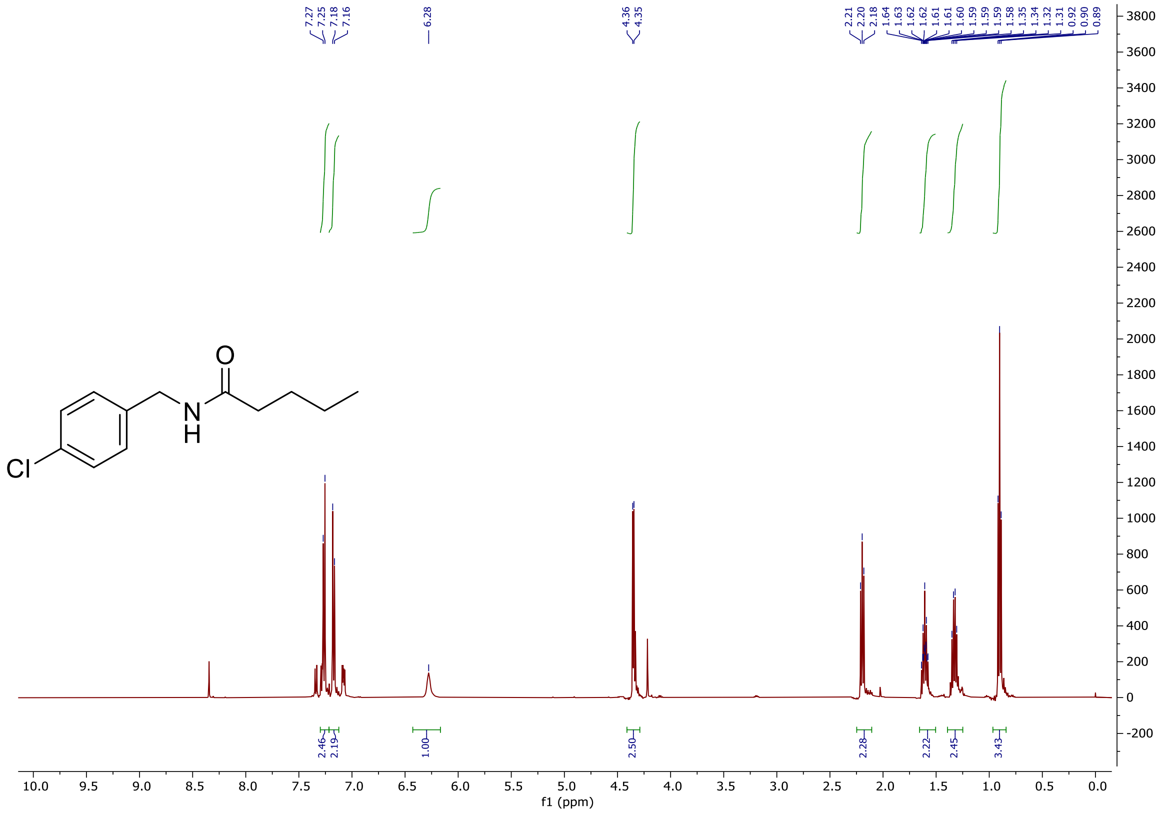

Supplementary Figure 106. ^{1}H NMR (400 MHz, $CDCl_3$) of compound **46a**

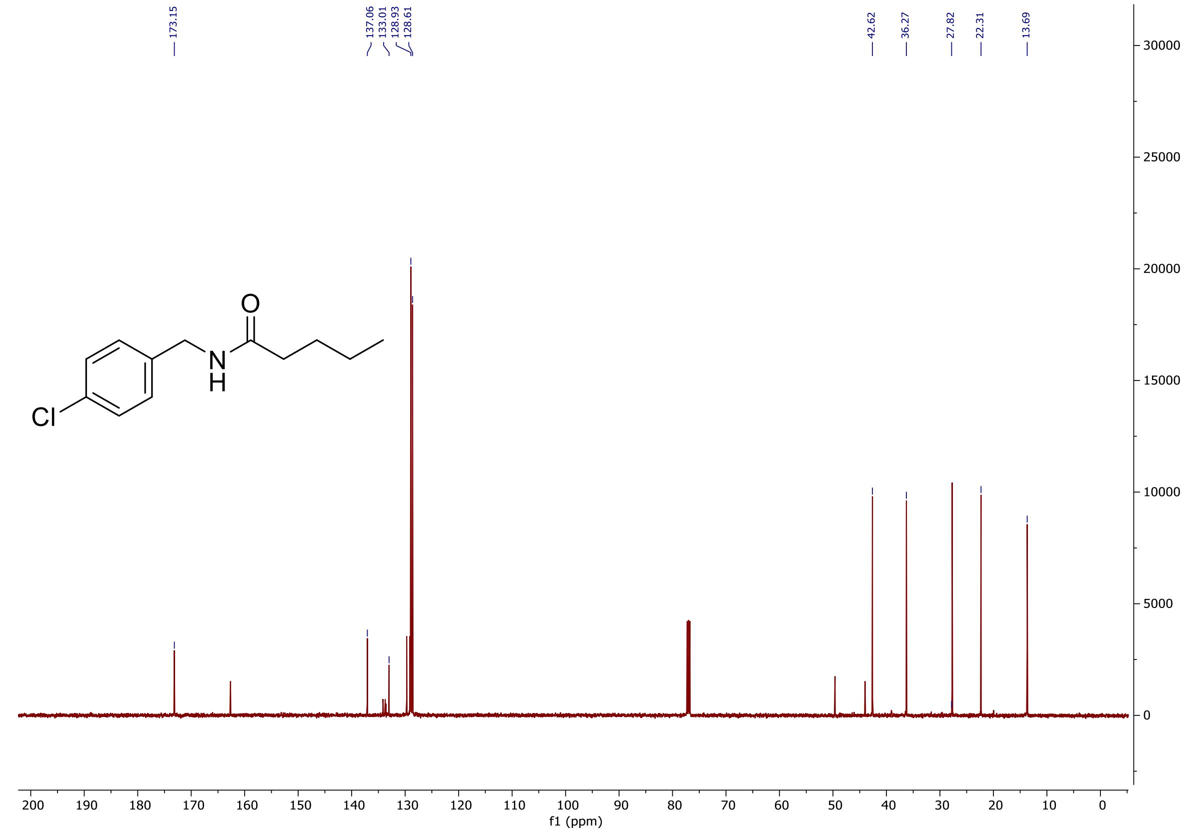

Supplementary Figure 107. ^{13}C NMR (126 MHz, $CDCl_3$) of compound **46a**

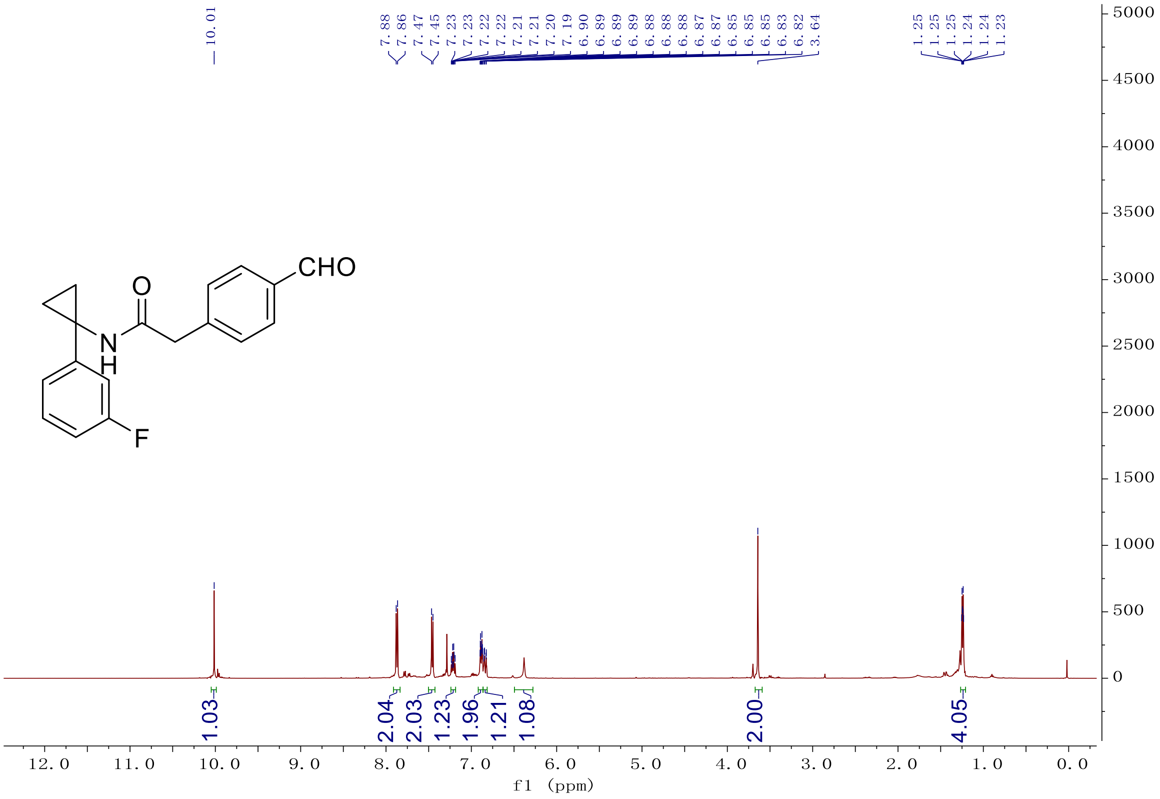

Supplementary Figure 108. ^{1}H NMR (400 MHz, $CDCl_3$) of compound **47a**

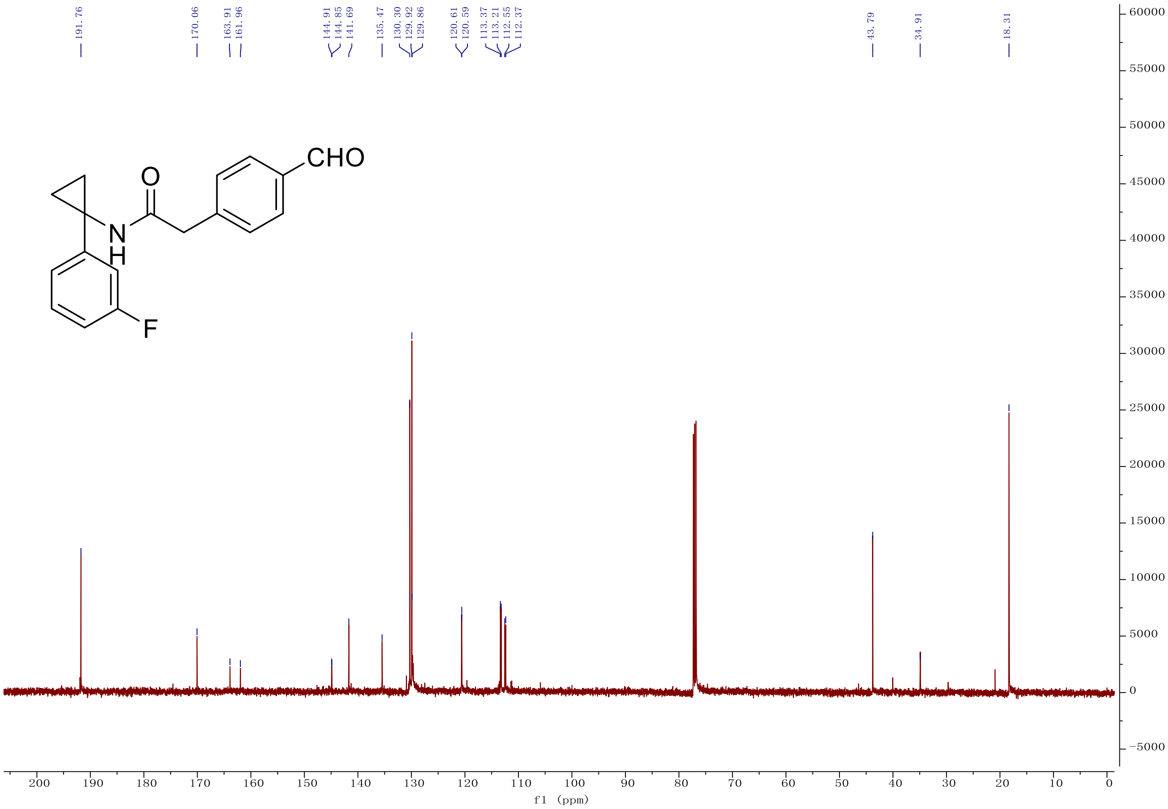

Supplementary Figure 109. ^{13}C NMR (126 MHz, $CDCl_3$) of compound **47a**

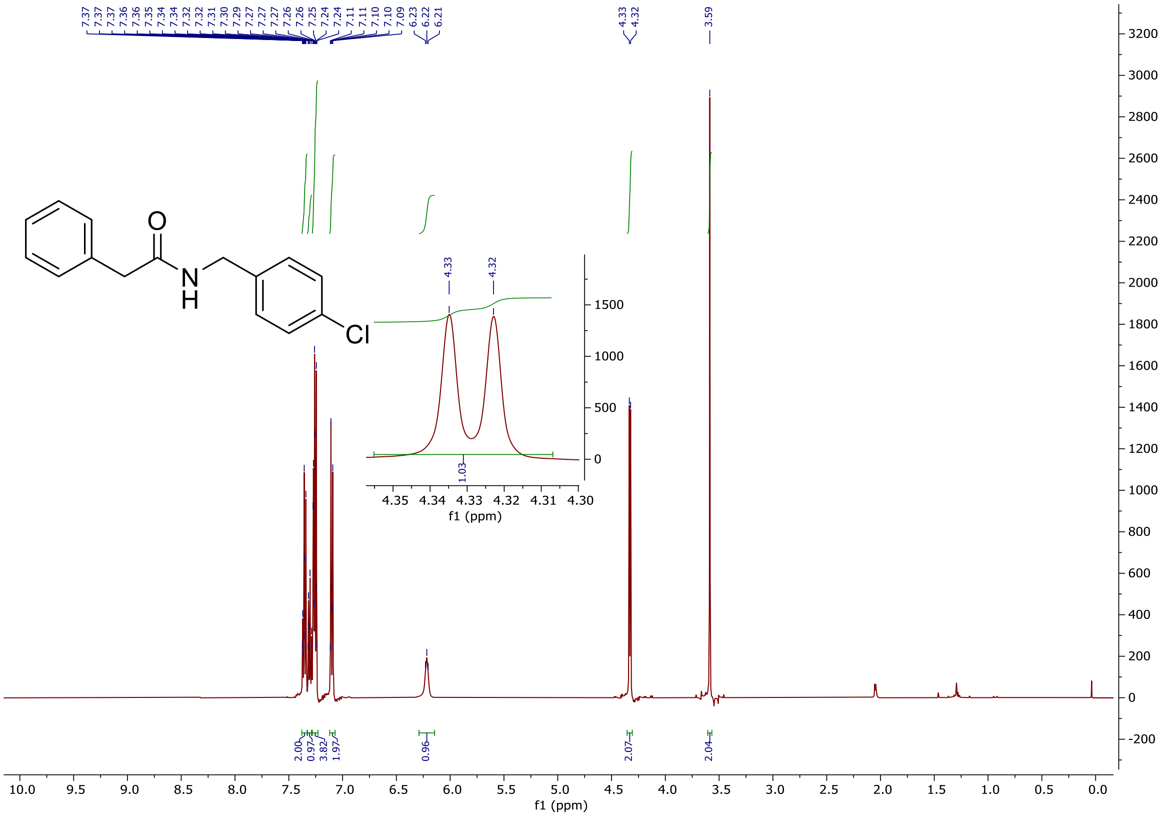

Supplementary Figure 110. ^{1}H NMR (400 MHz, $CDCl_3$) of compound **48a**

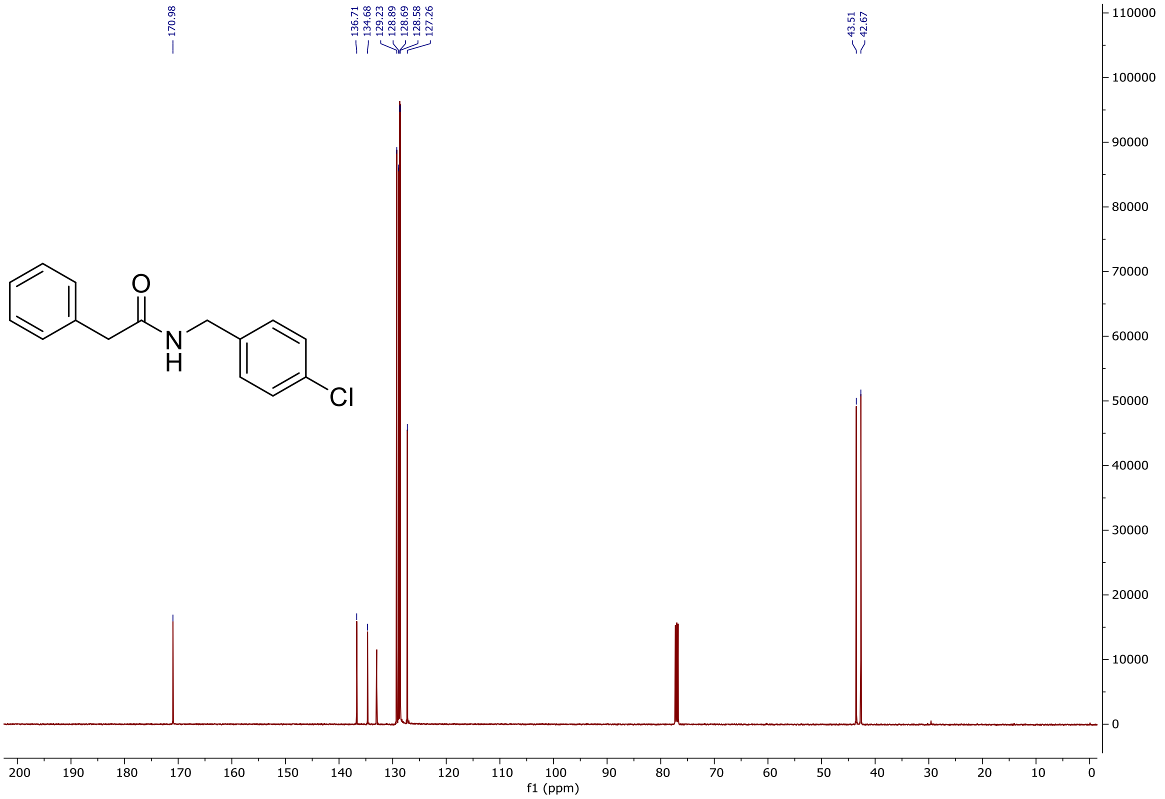

Supplementary Figure 111. ^{13}C NMR (126 MHz, $CDCl_3$) of compound **48a**

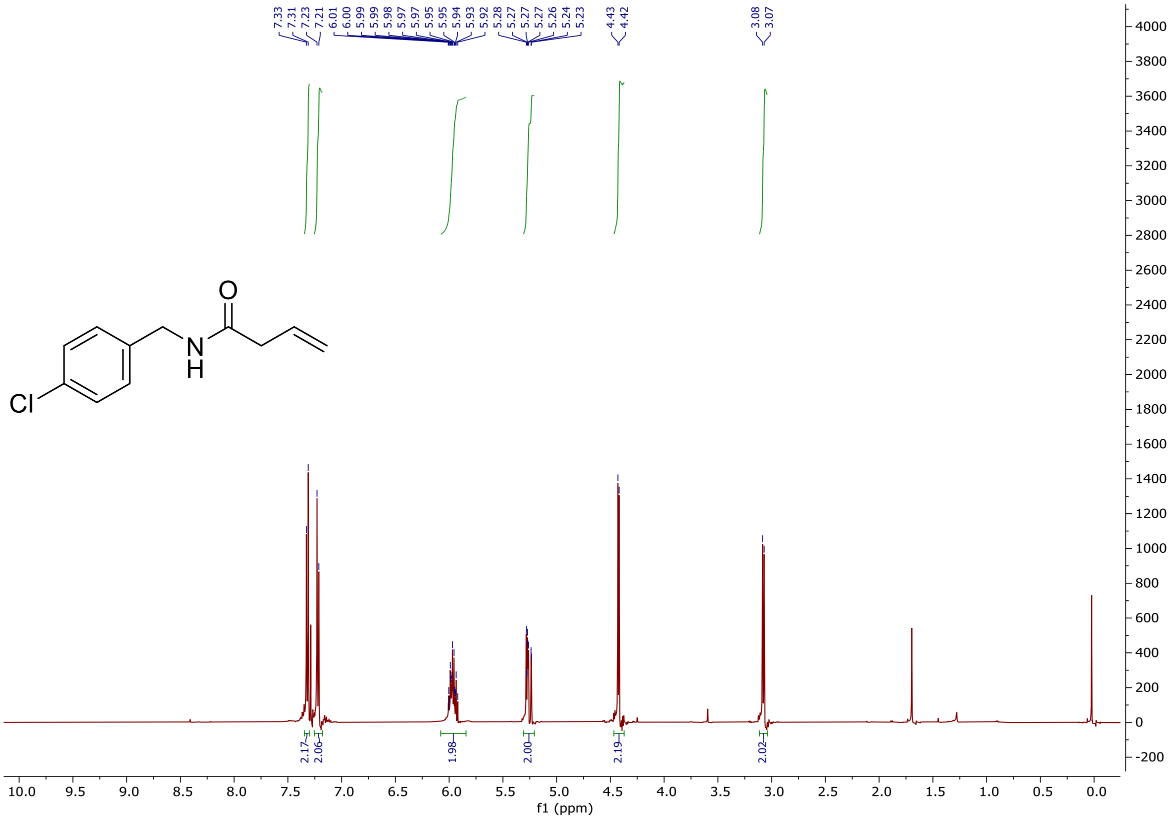

Supplementary Figure 112. ^1H NMR (400 MHz, $CDCl_3$) of compound **49a**

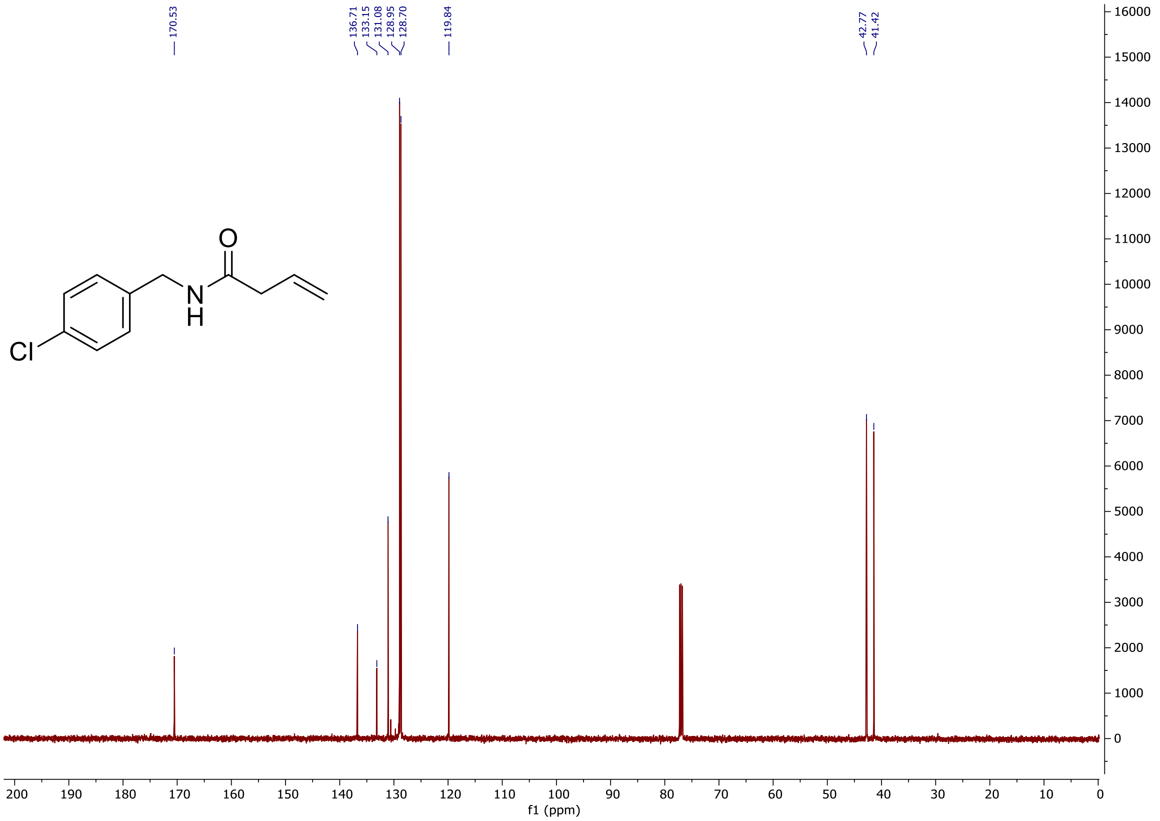

Supplementary Figure 113. ^{13}C NMR (126 MHz, $CDCl_3$) of compound **49a**

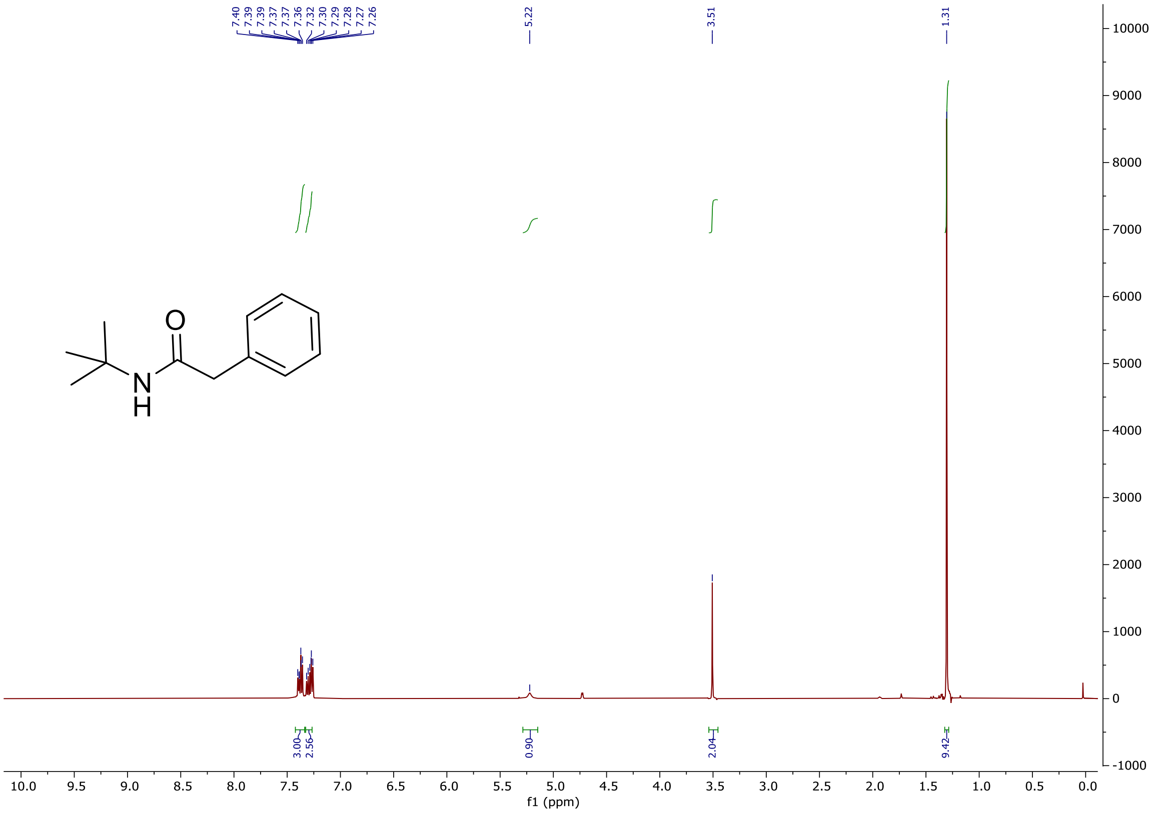

Supplementary Figure 114. ^{1}H NMR (400 MHz, $CDCl_3$) of compound **50a**

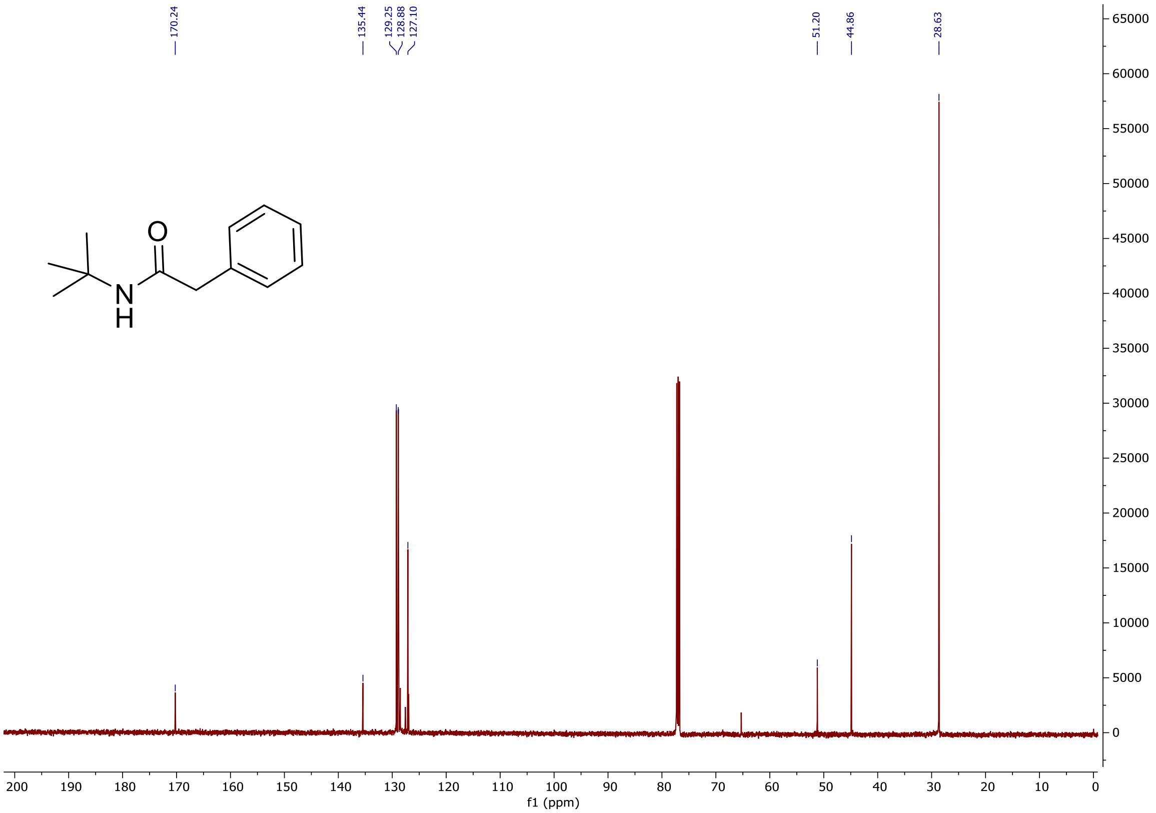

Supplementary Figure 115. ^{13}C NMR (126 MHz, $CDCl_3$) of compound **50a**

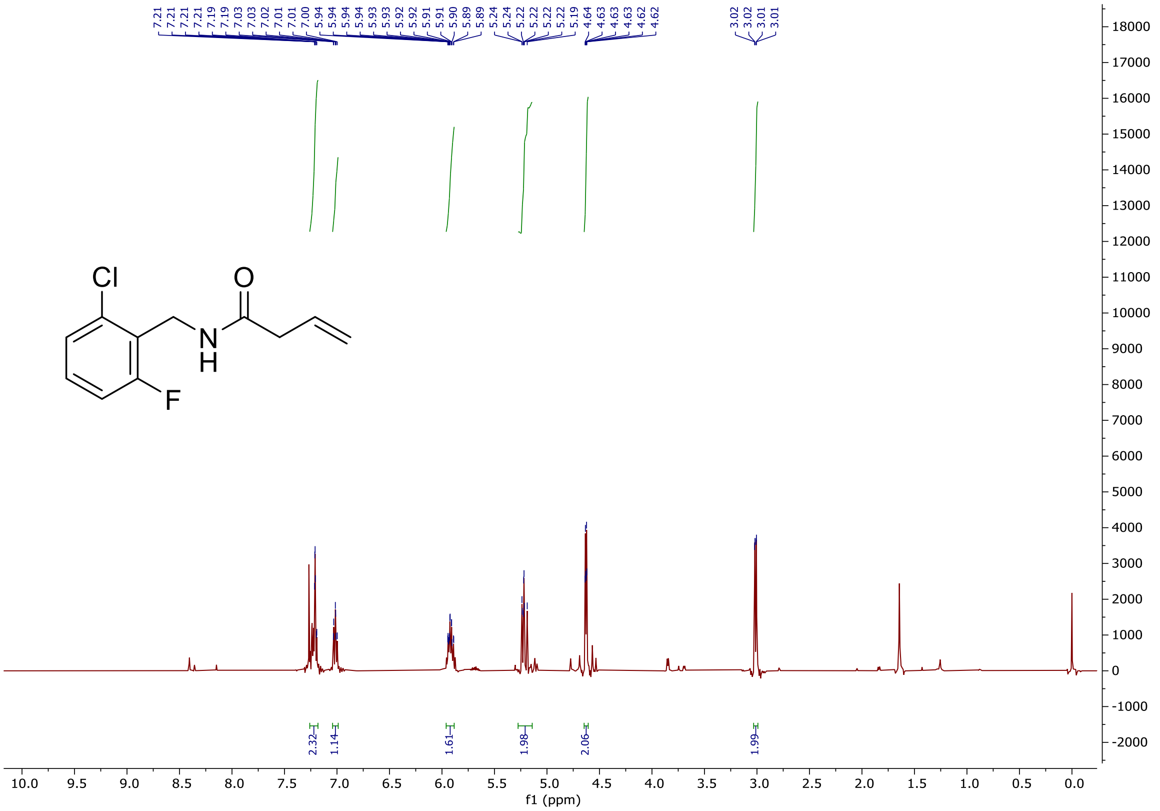

Supplementary Figure 116. ^{1}H NMR (400 MHz, $CDCl_3$) of compound **51a**

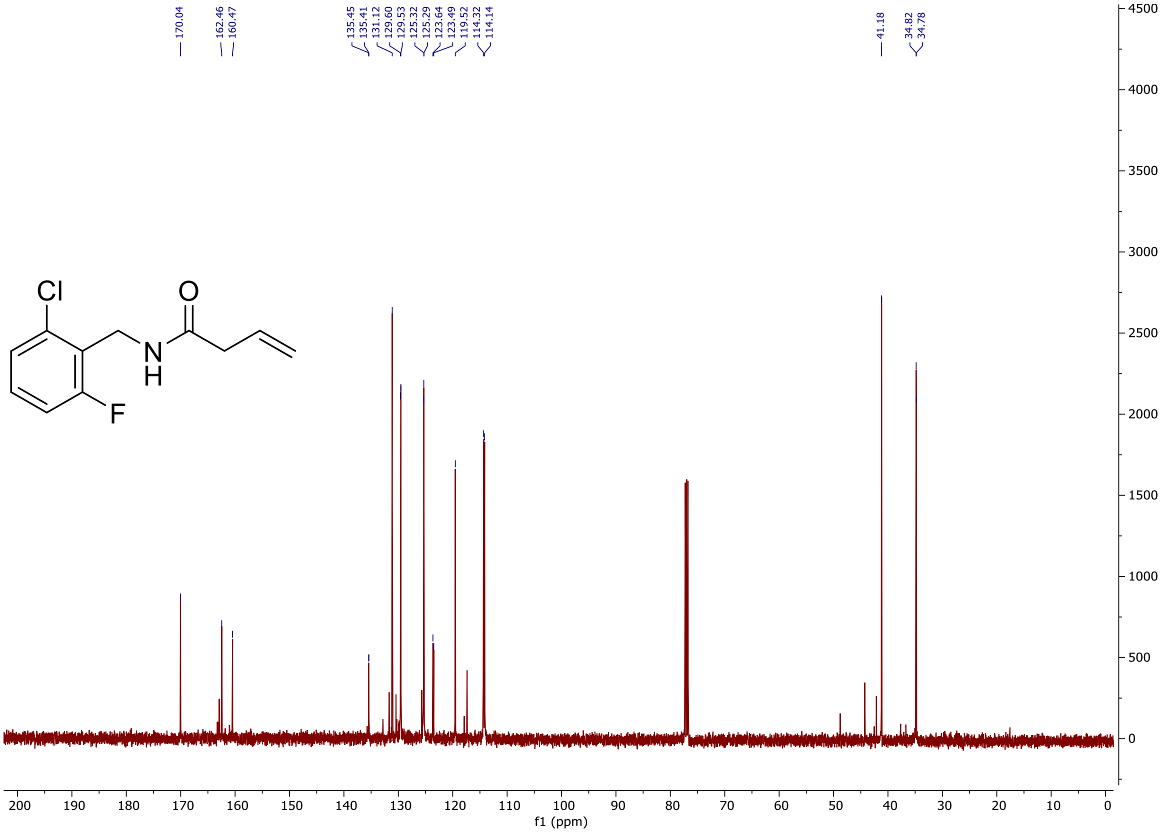

Supplementary Figure 117. ^{13}C NMR (126 MHz, $CDCl_3$) of compound **51a**

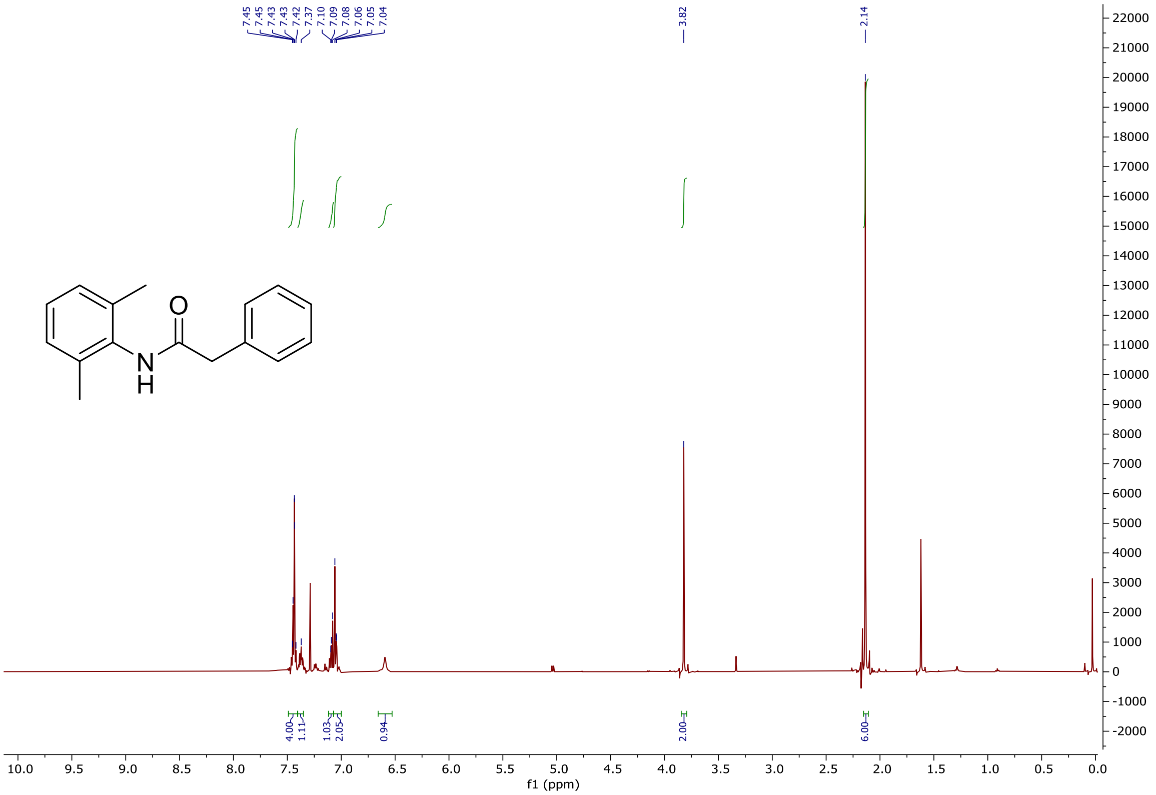

Supplementary Figure 118. ^{1}H NMR (400 MHz, $CDCl_3$) of compound **52a**

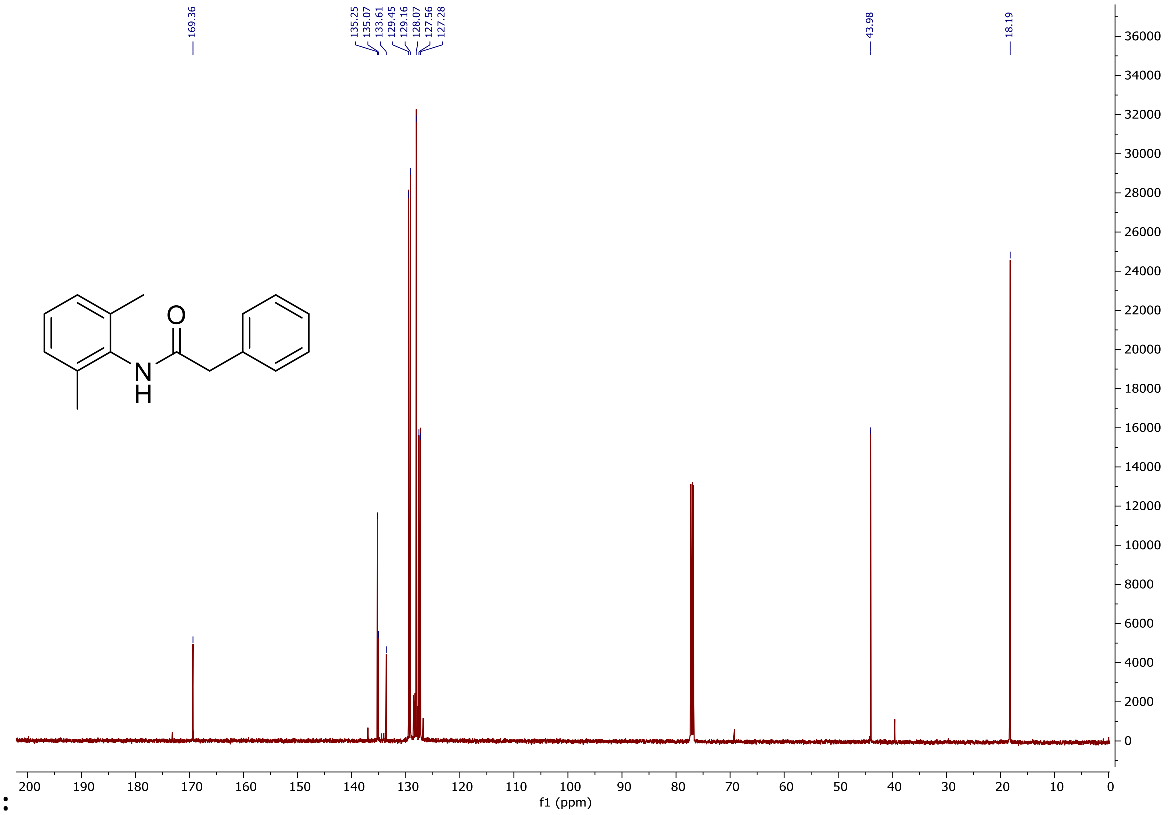

:

Supplementary Figure 119. ^{13}C NMR (126 MHz, $CDCl_3$) of compound **52a**

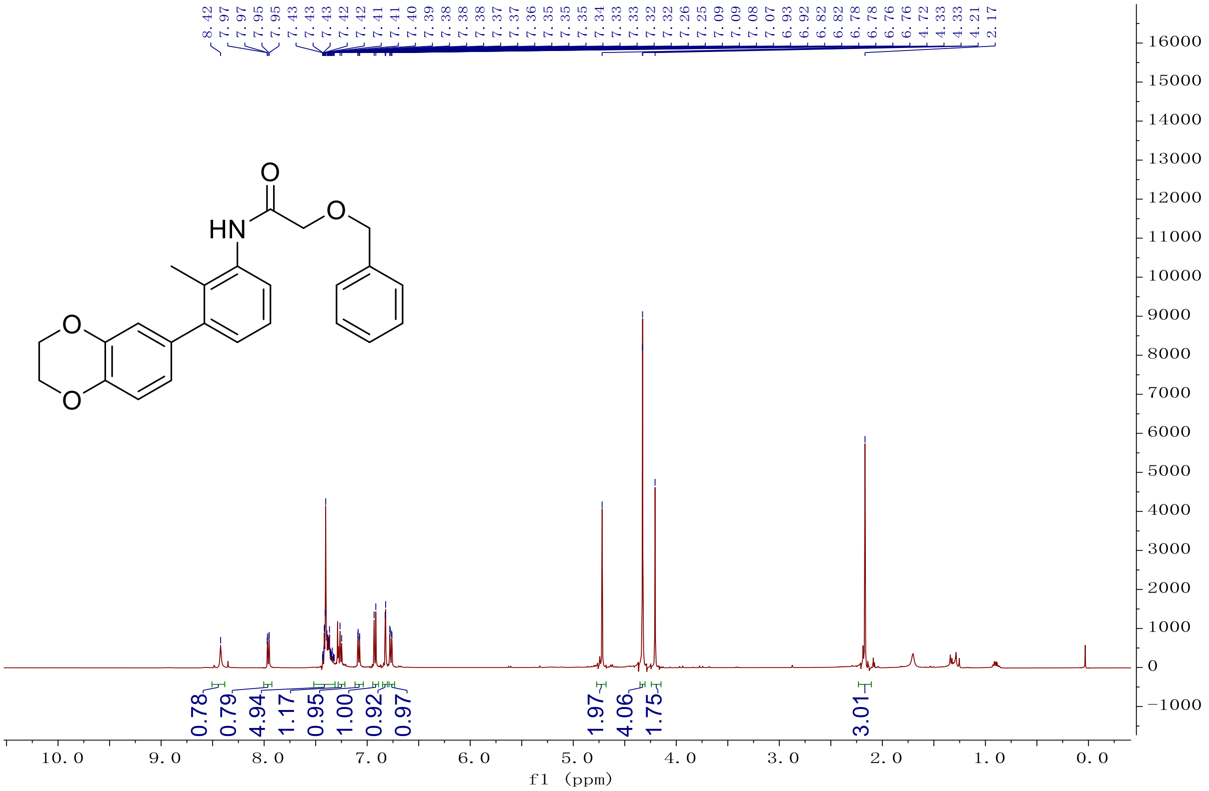

Supplementary Figure 120. ^{1}H NMR (400 MHz, $CDCl_3$) of compound **53a**

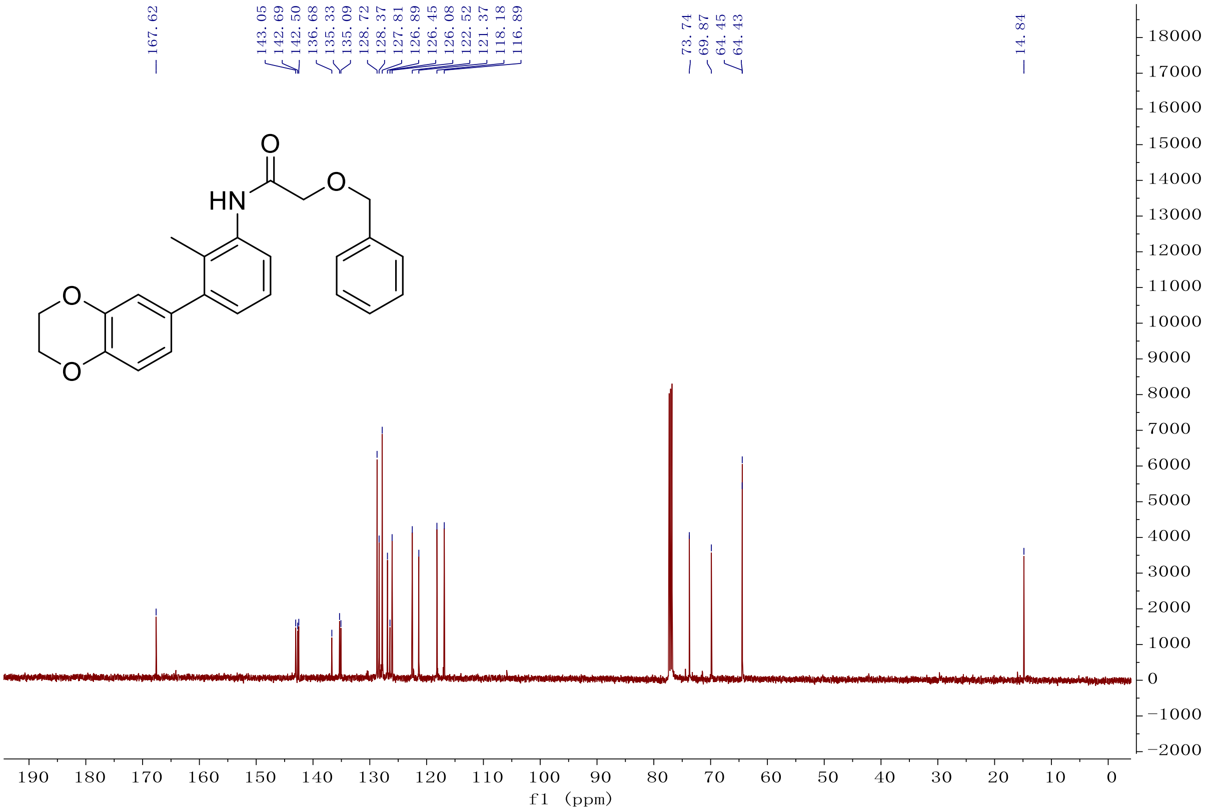

Supplementary Figure 121. ^{13}C NMR (126 MHz, $CDCl_3$) of compound **53a**

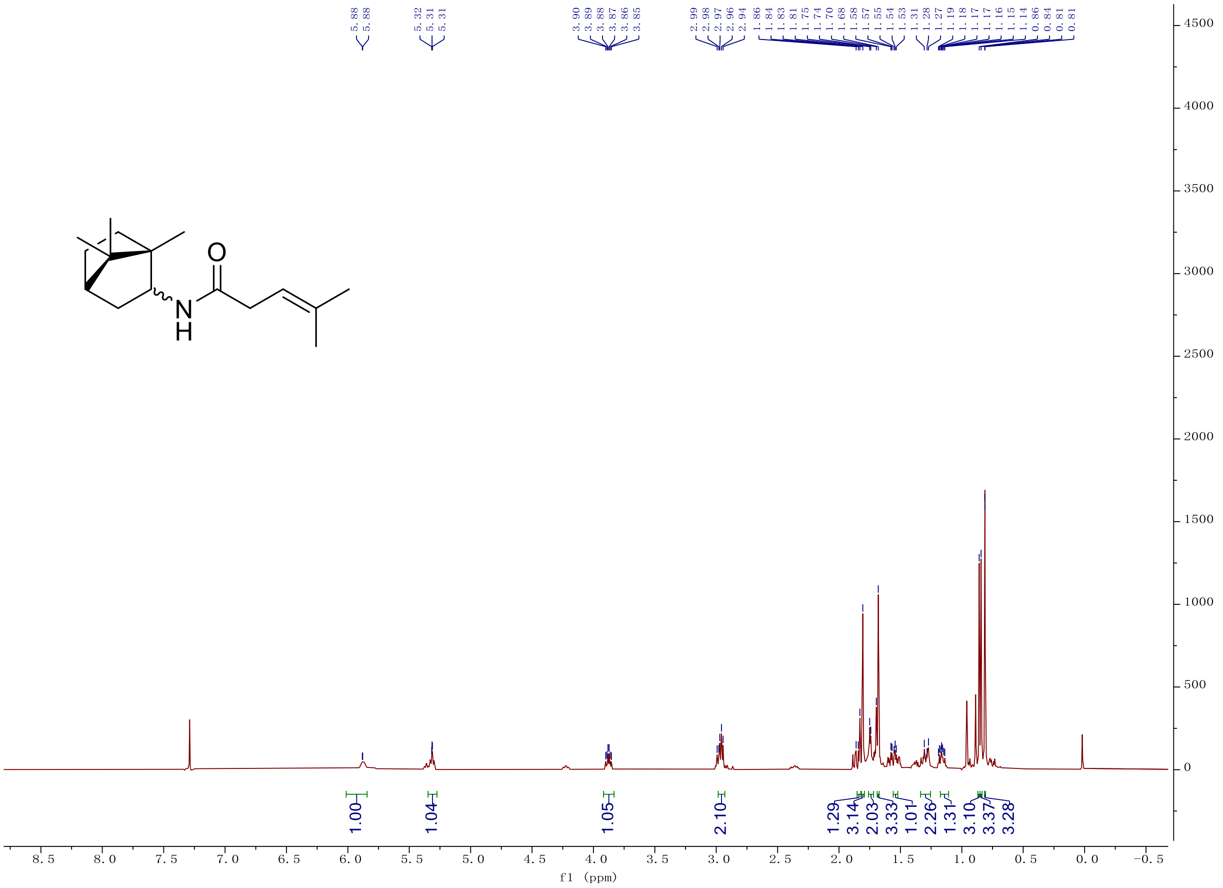

Supplementary Figure 122. ^{1}H NMR (400 MHz, $CDCl_3$) of compound **54a**

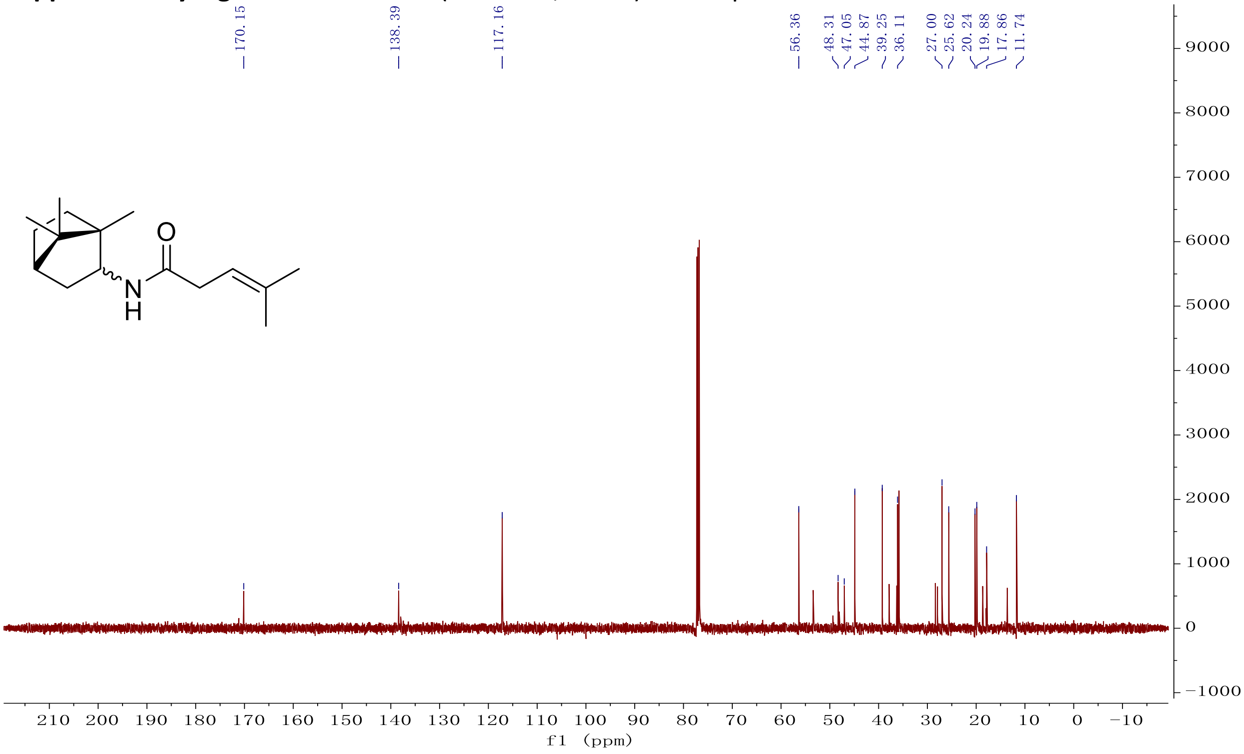

Supplementary Figure 123. ^{13}C NMR (126 MHz, $CDCl_3$) of compound **54a**

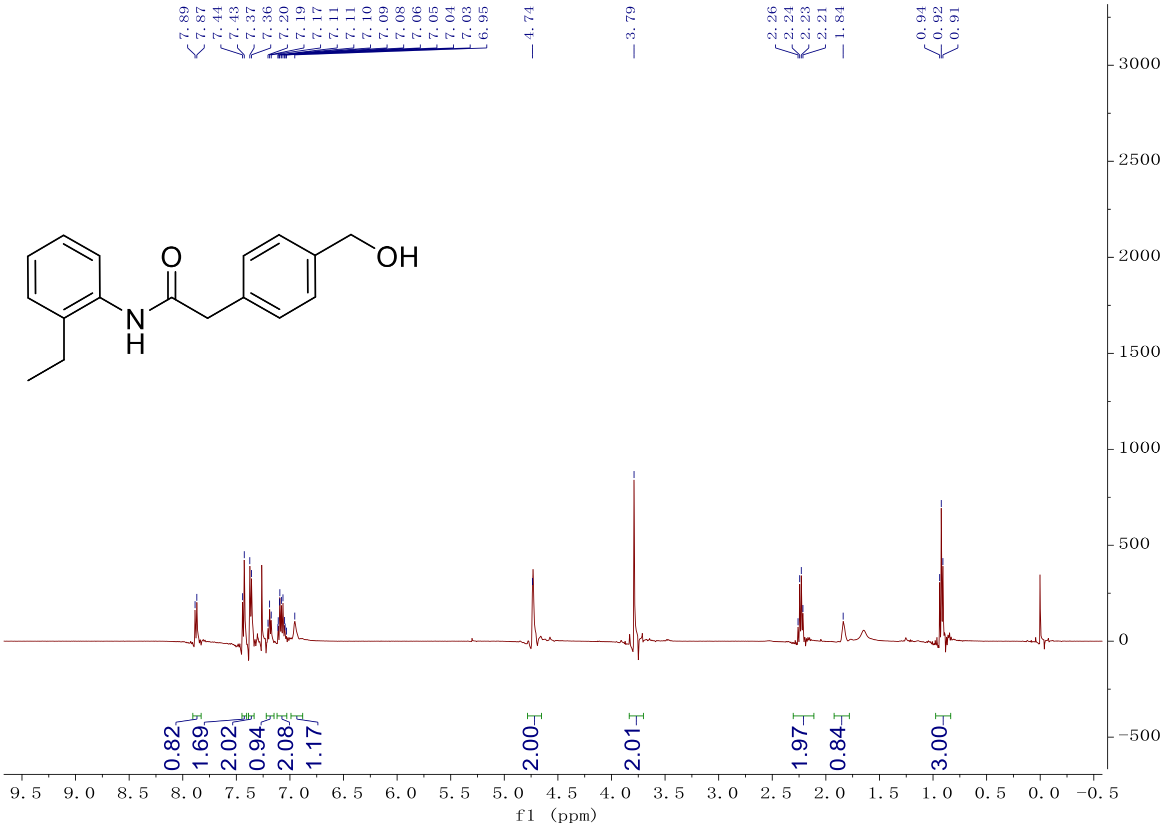

Supplementary Figure 124. ^{1}H NMR (400 MHz, $CDCl_3$) of compound **55a**

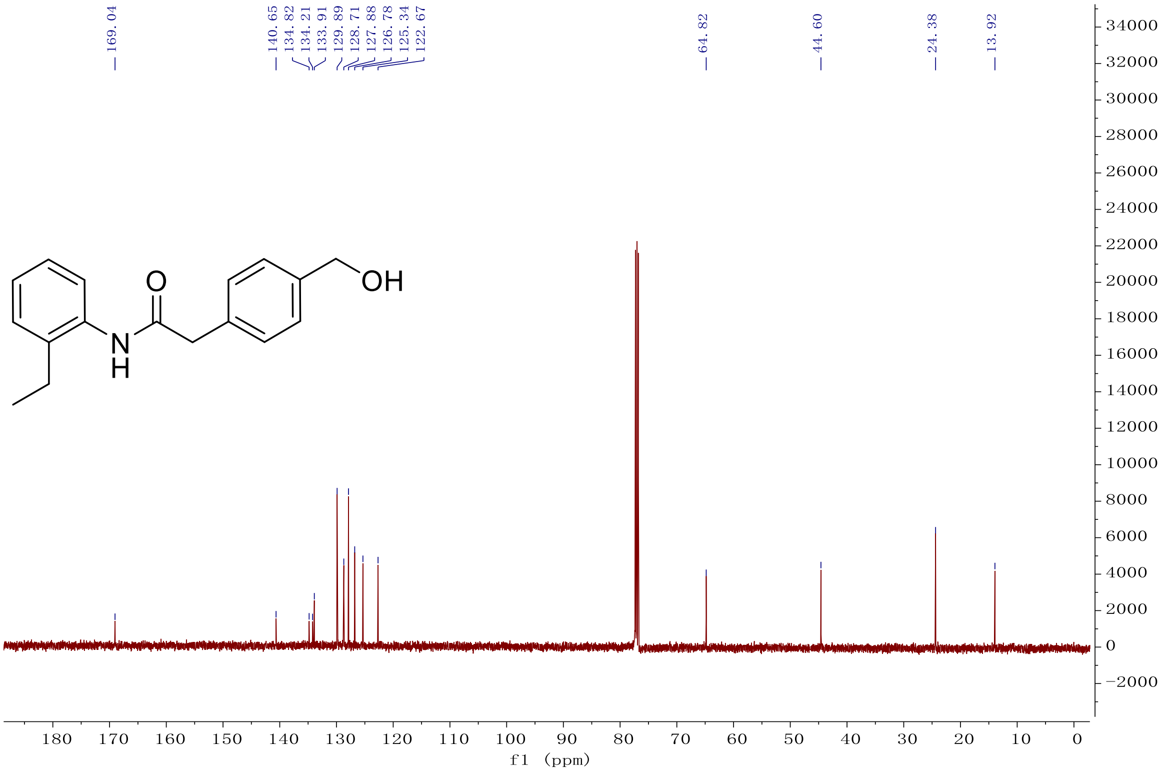

Supplementary Figure 125. ^{13}C NMR (126 MHz, $CDCl_3$) of compound **55a**

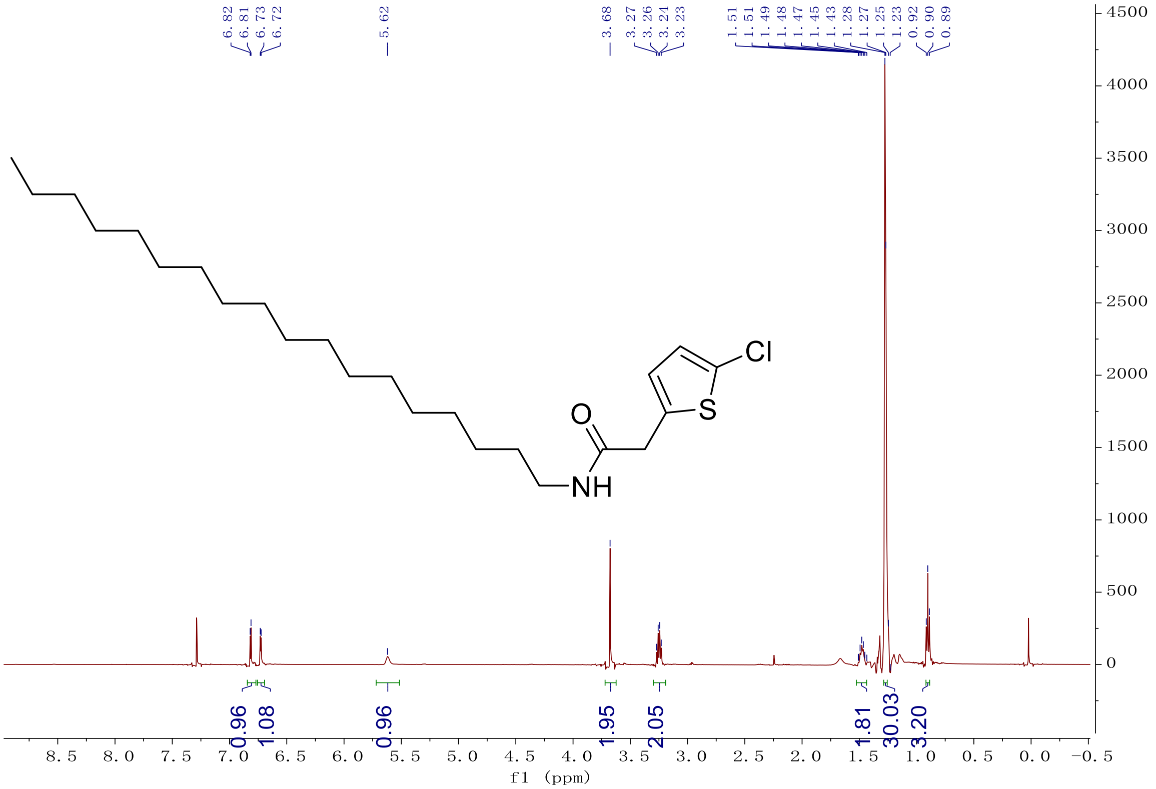

Supplementary Figure 126. ^{1}H NMR (400 MHz, $CDCl_3$) of compound **56a**

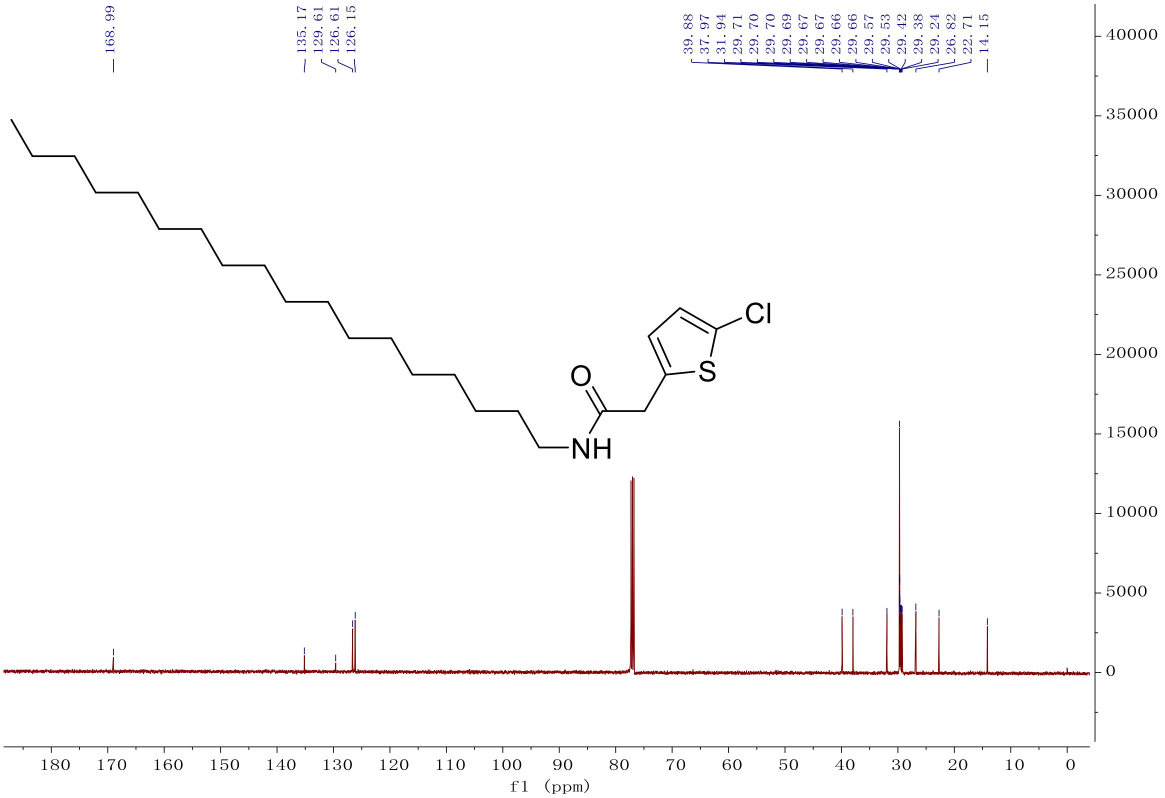

Supplementary Figure 127. ^{13}C NMR (126 MHz, $CDCl_3$) of compound **56a**

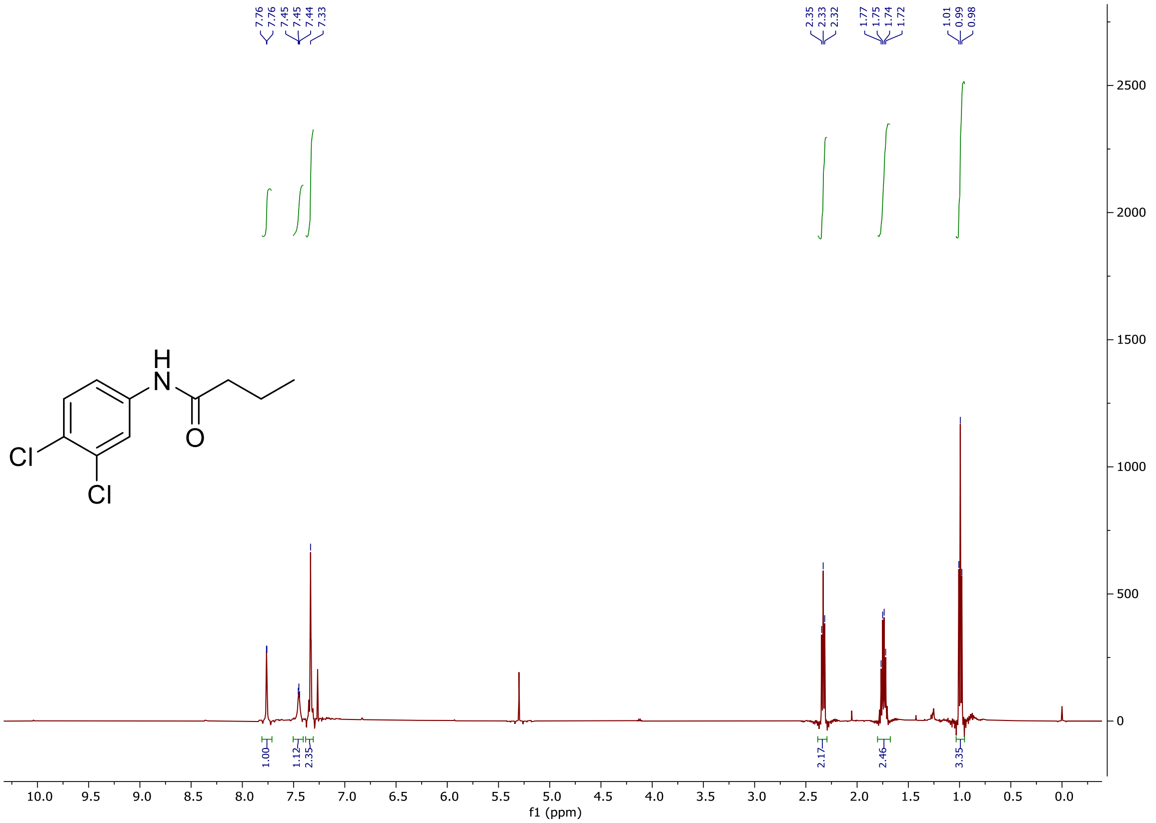

Supplementary Figure 128. ^{1}H NMR (400 MHz, $CDCl_3$) of compound **57a**

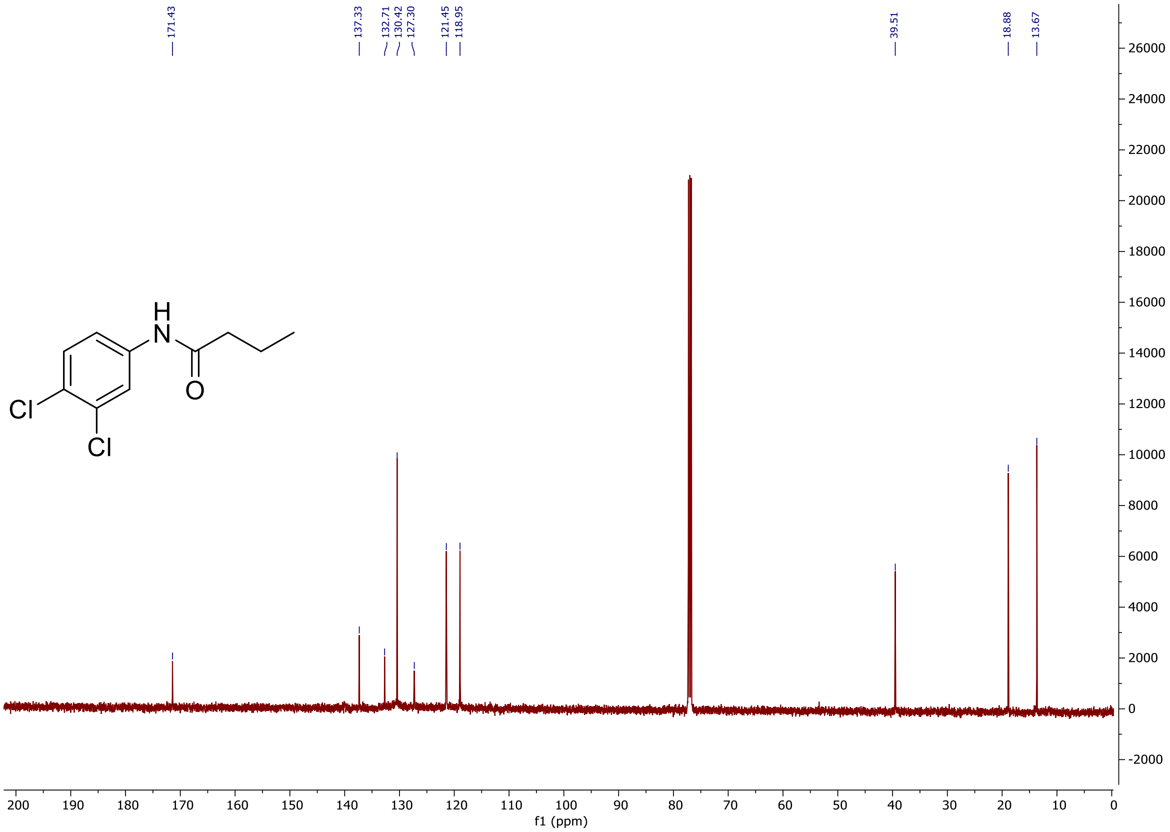

Supplementary Figure 129. ^{13}C NMR (126 MHz, $CDCl_3$) of compound **57a**

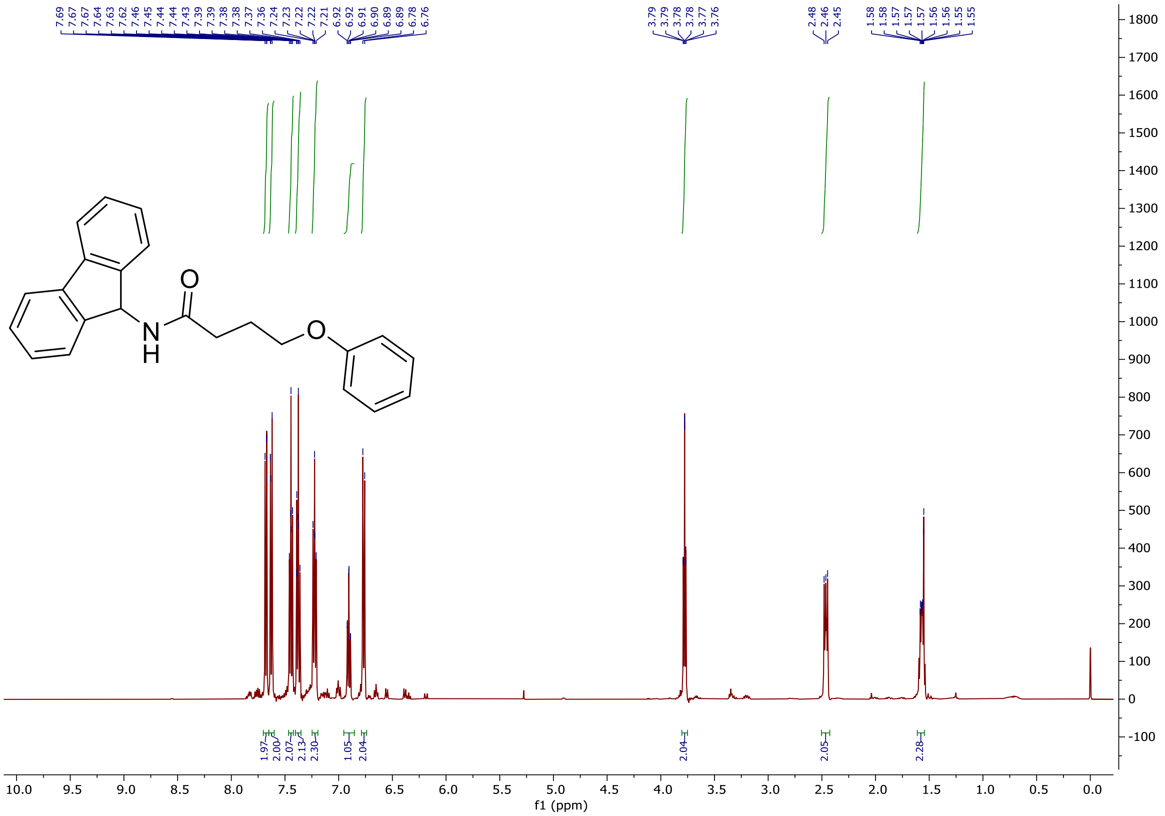

Supplementary Figure 130. ^{1}H NMR (400 MHz, $CDCl_3$) of compound **58a**

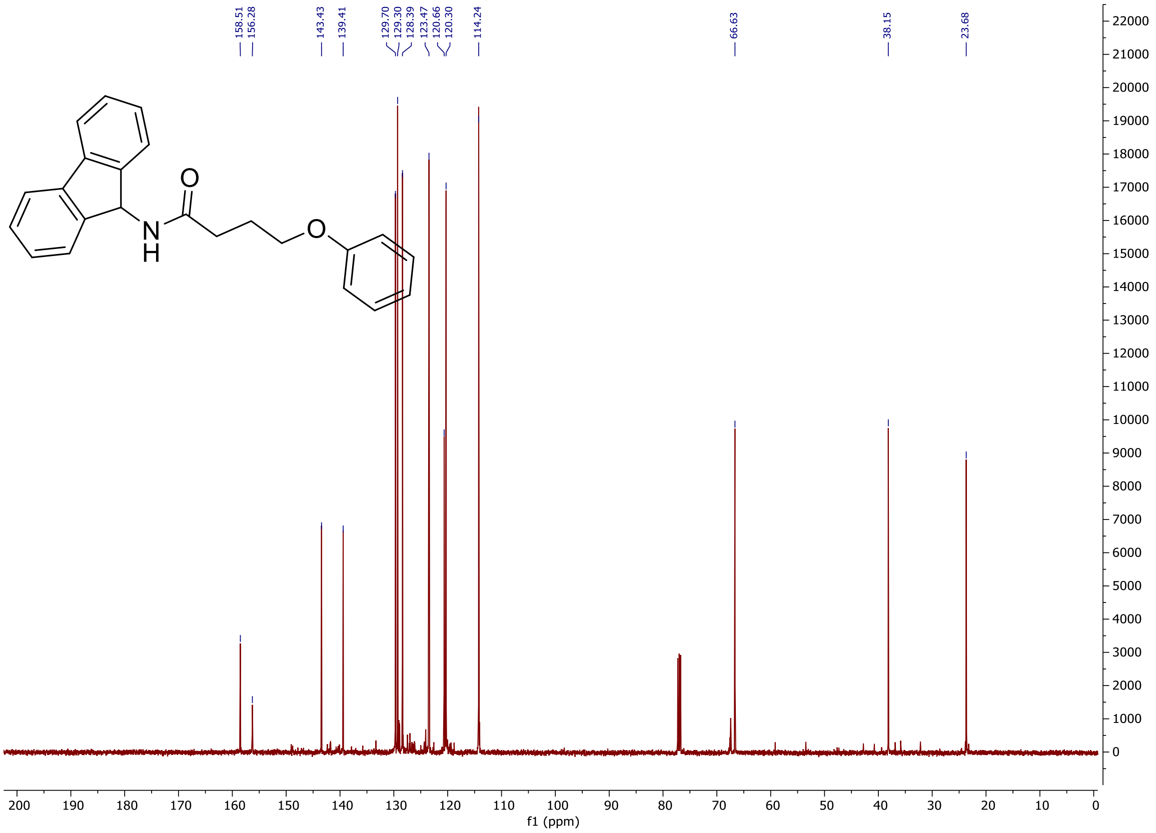

Supplementary Figure 131. ^{13}C NMR (126 MHz, $CDCl_3$) of compound **58a**

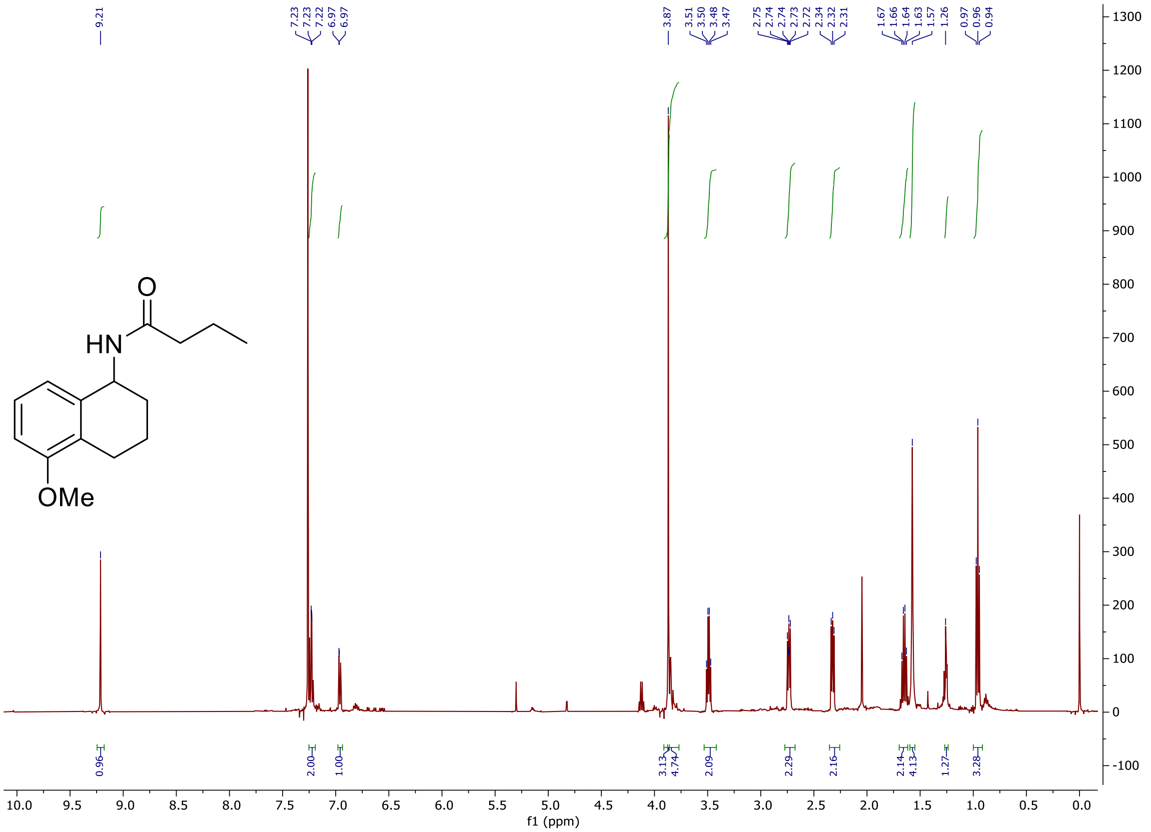

Supplementary Figure 132. ^{1}H NMR (400 MHz, $CDCl_3$) of compound **59a**

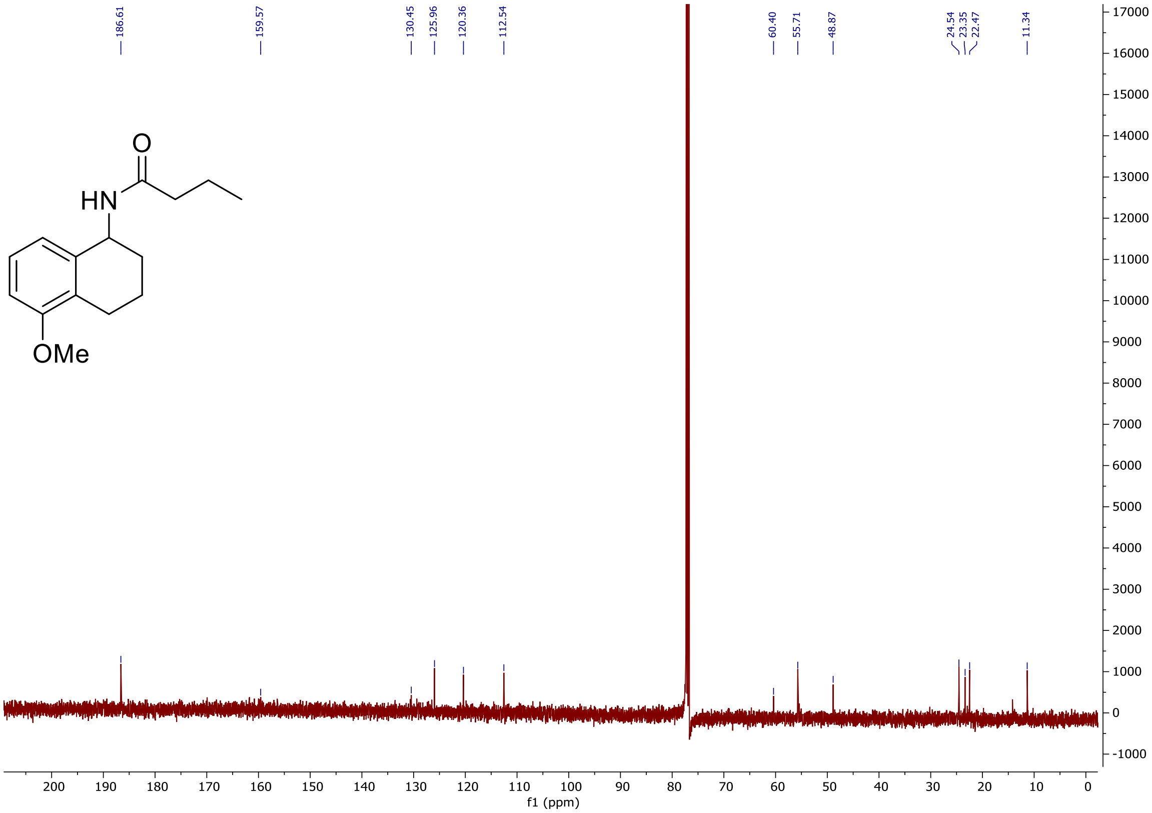

Supplementary Figure 133. ^{13}C NMR (126 MHz, $CDCl_3$) of compound **59a**

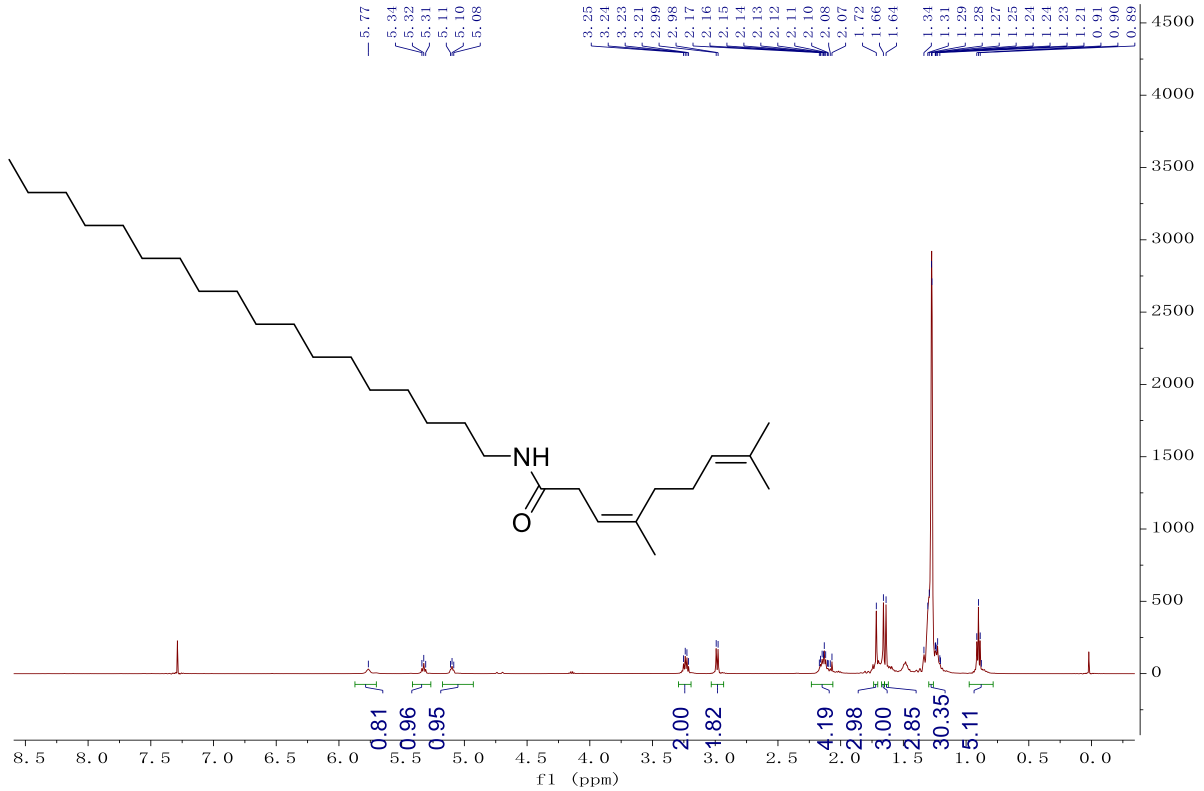

Supplementary Figure 134. ^{1}H NMR (400 MHz, $CDCl_3$) of compound **60a**

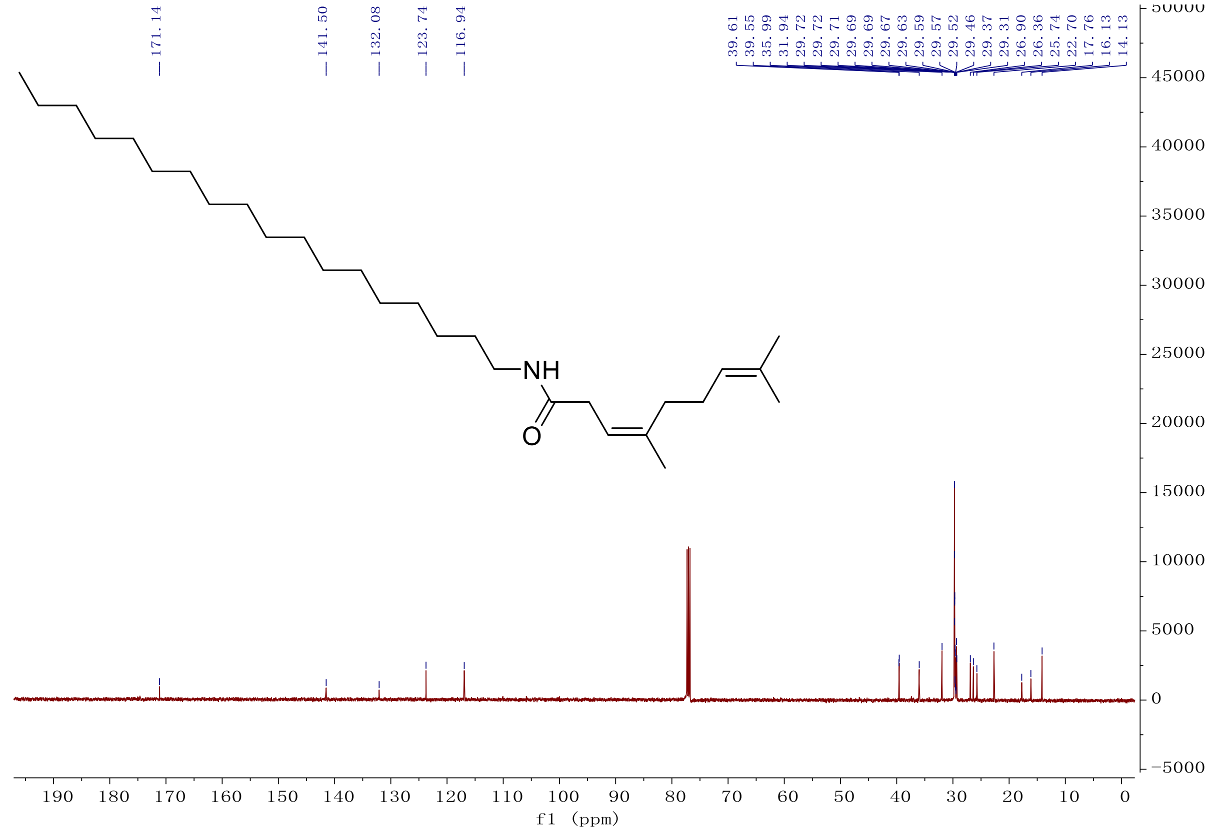

Supplementary Figure 135. ^{13}C NMR (126 MHz, $CDCl_3$) of compound **61a**

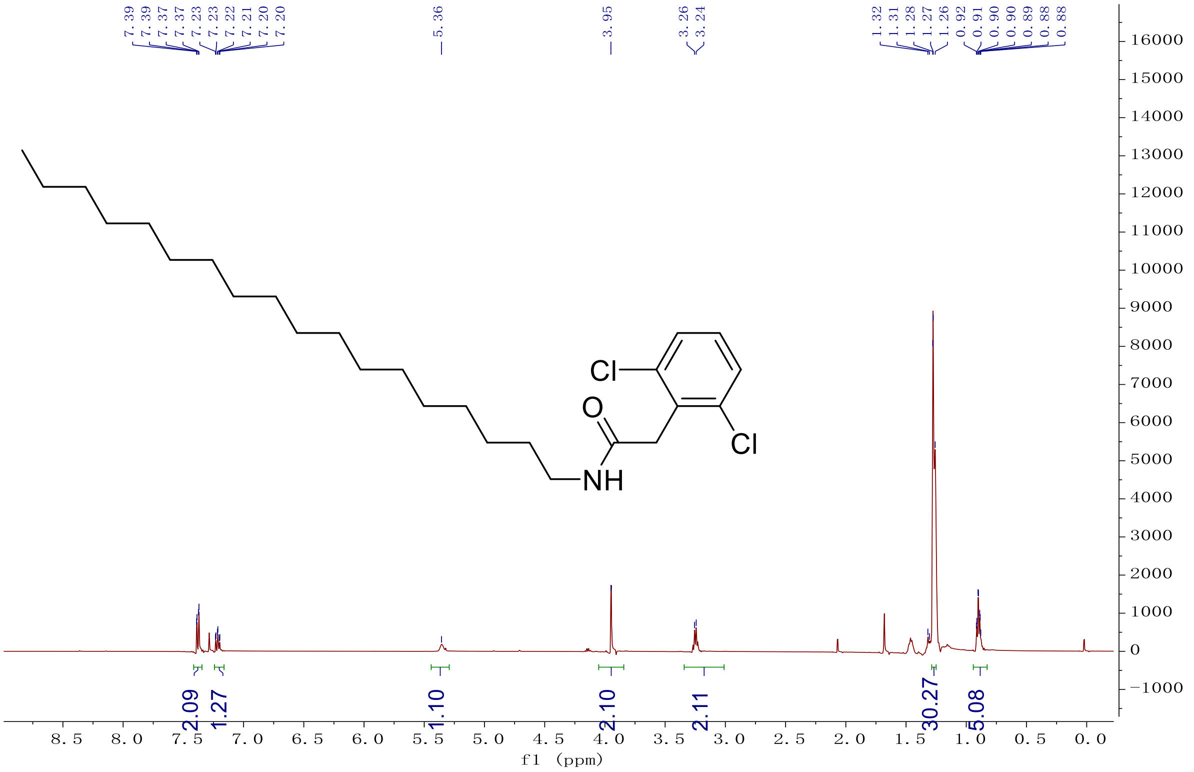

Supplementary Figure 136. ^{1}H NMR (400 MHz, $CDCl_3$) of compound **61a**

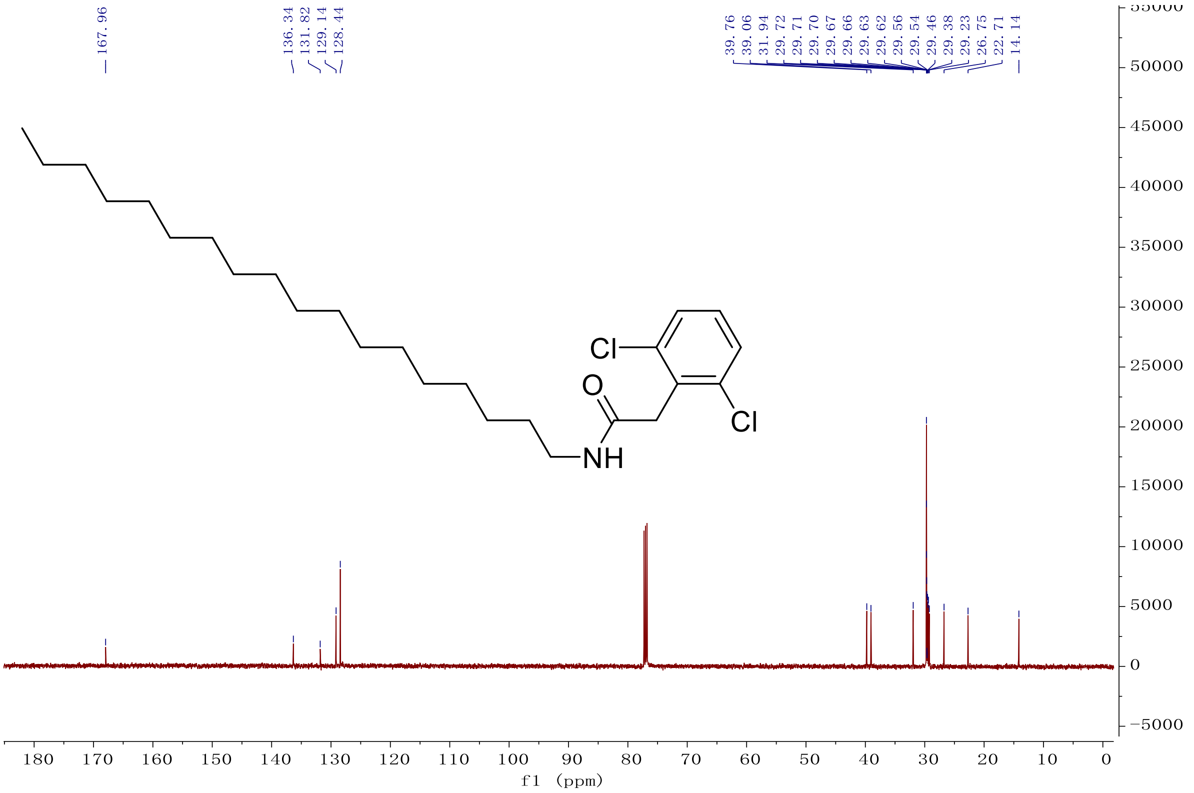

Supplementary Figure 137. ^{13}C NMR (126 MHz, $CDCl_3$) of compound **61a**

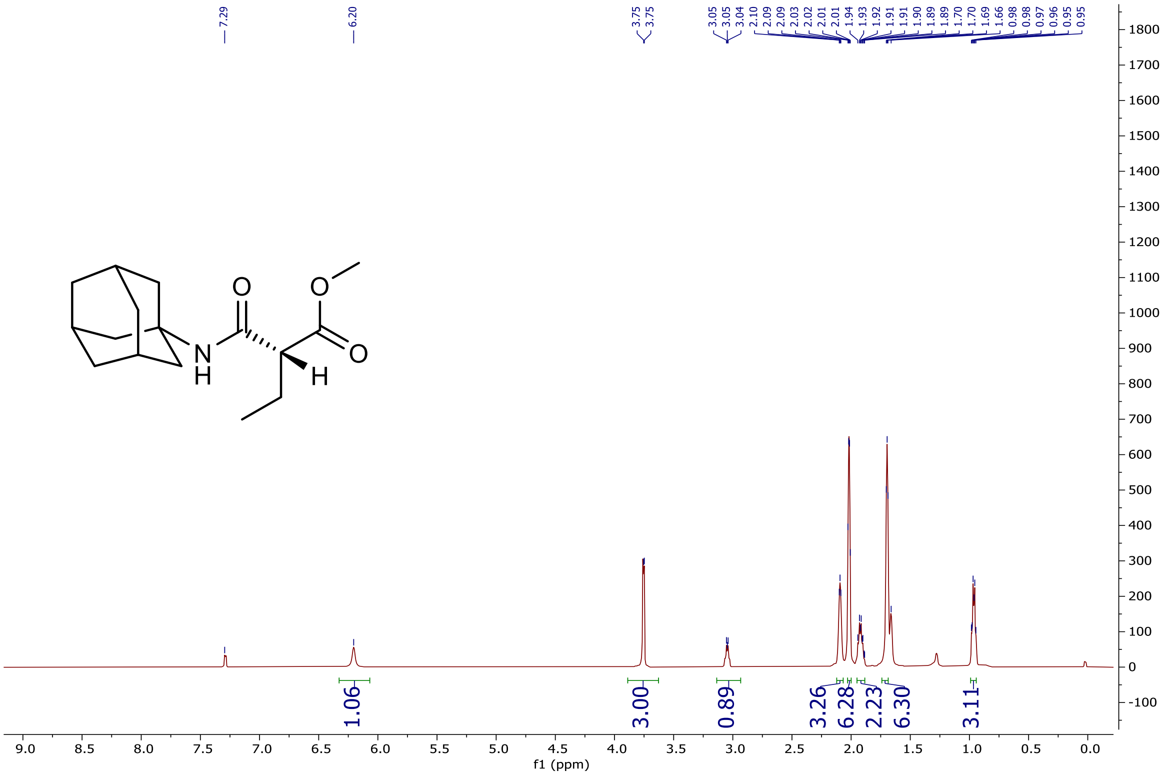

Supplementary Figure 138. ^{1}H NMR (400 MHz, $CDCl_3$) of compound **62a**

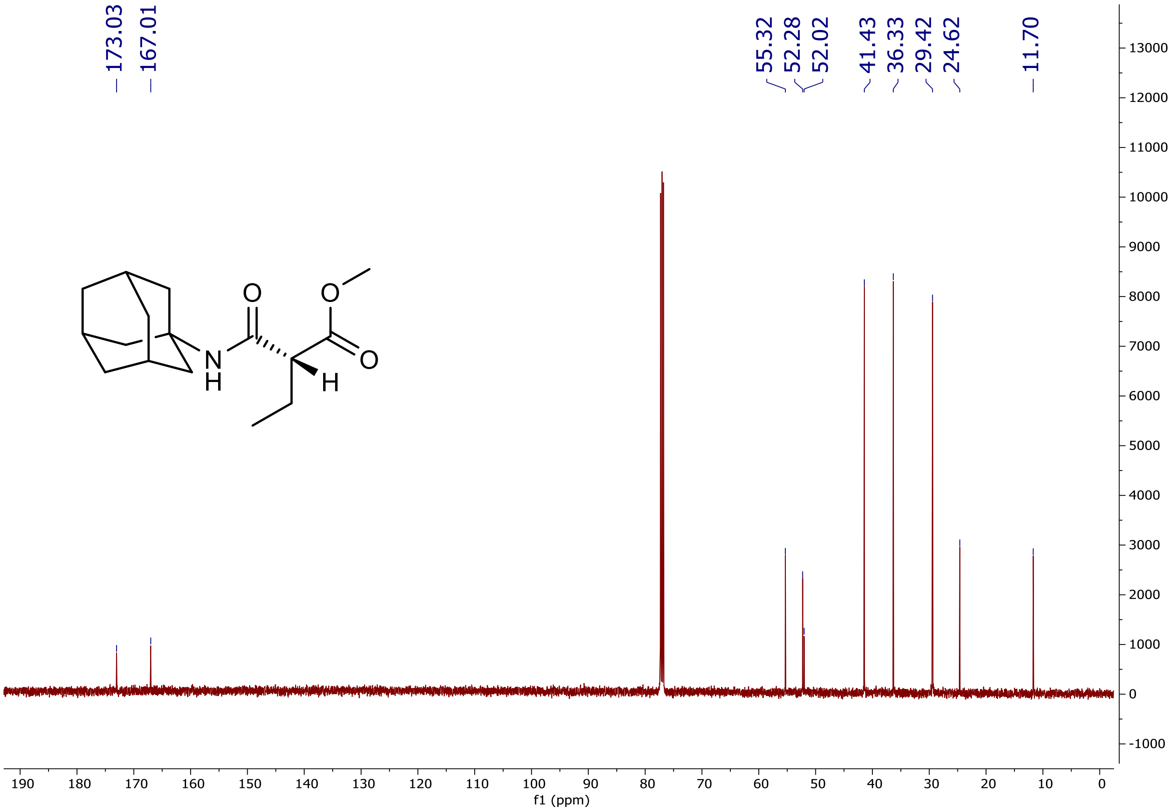

Supplementary Figure 139. ^{13}C NMR (126 MHz, $CDCl_3$) of compound **62a**

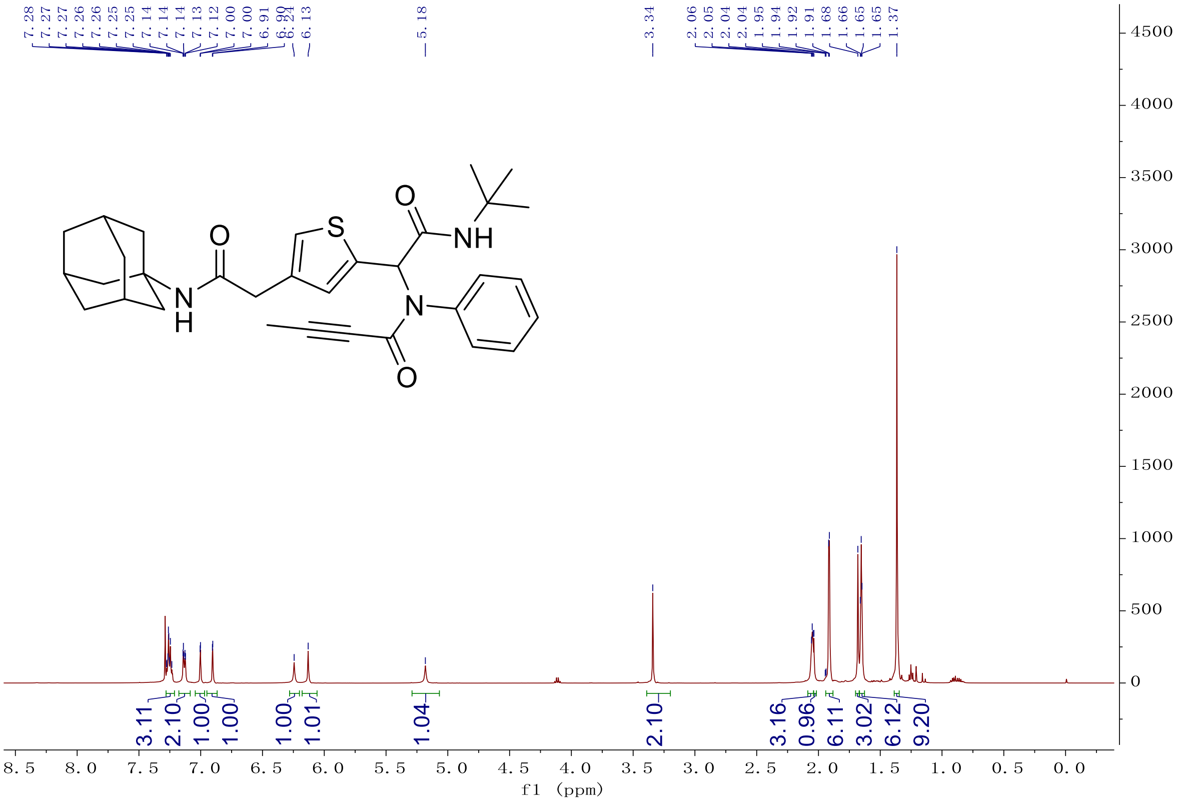

Supplementary Figure 140. ^{1}H NMR (400 MHz, $CDCl_3$) of compound **1b**

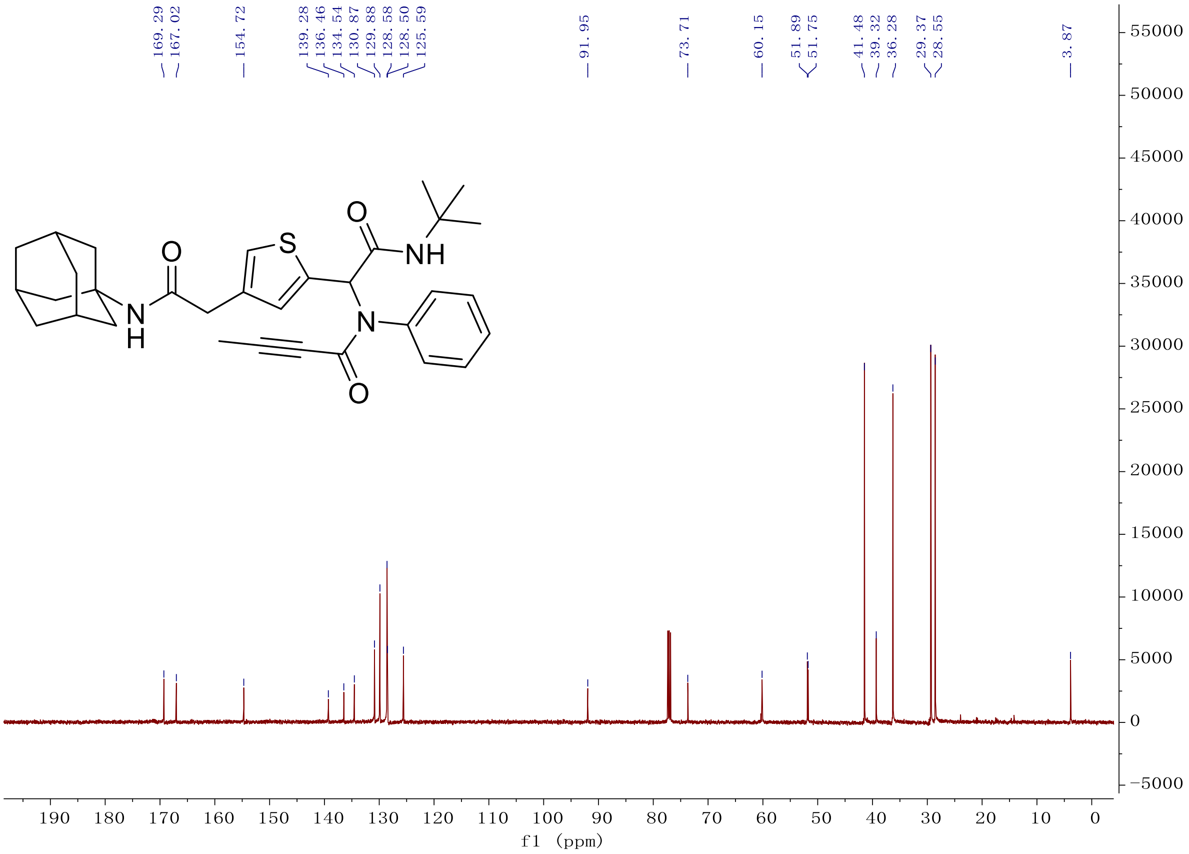

Supplementary Figure 141. ^{13}C NMR (126 MHz, $CDCl_3$) of compound **1b**

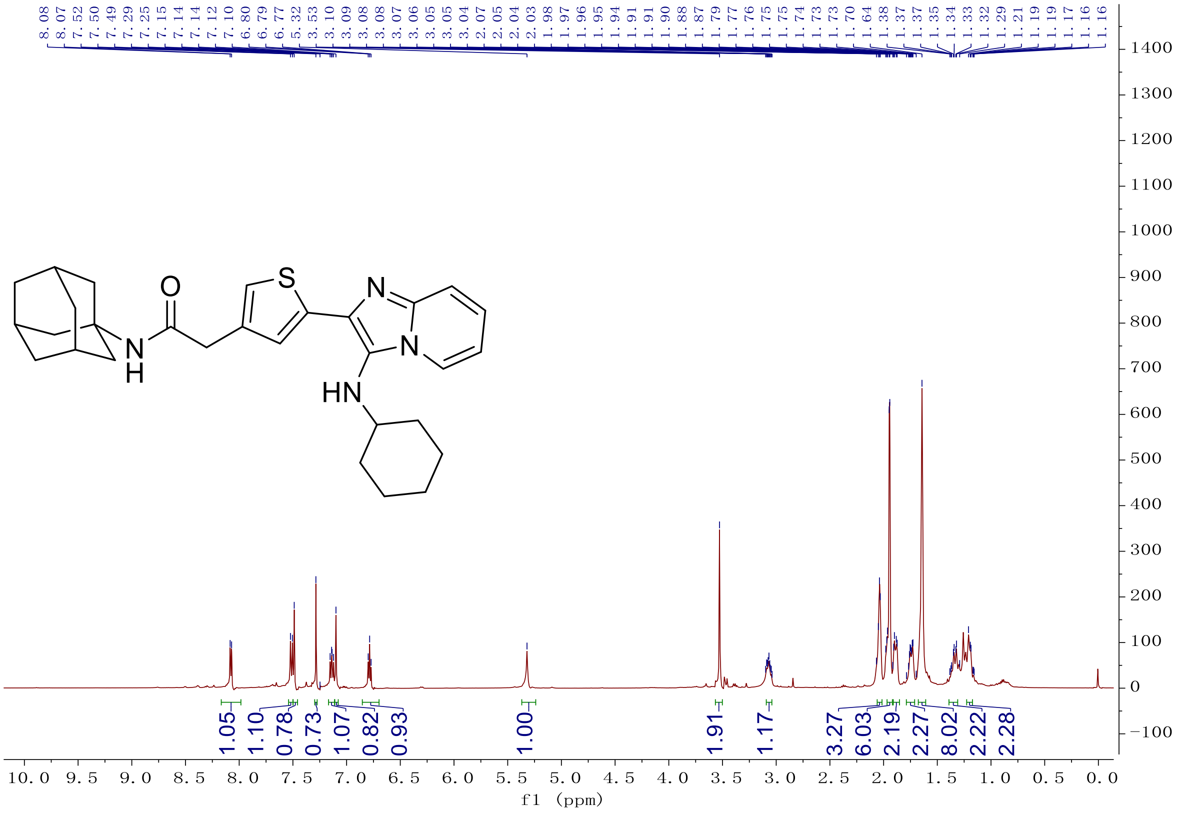

Supplementary Figure 142. ^{1}H NMR (400 MHz, $CDCl_3$) of compound **1c**

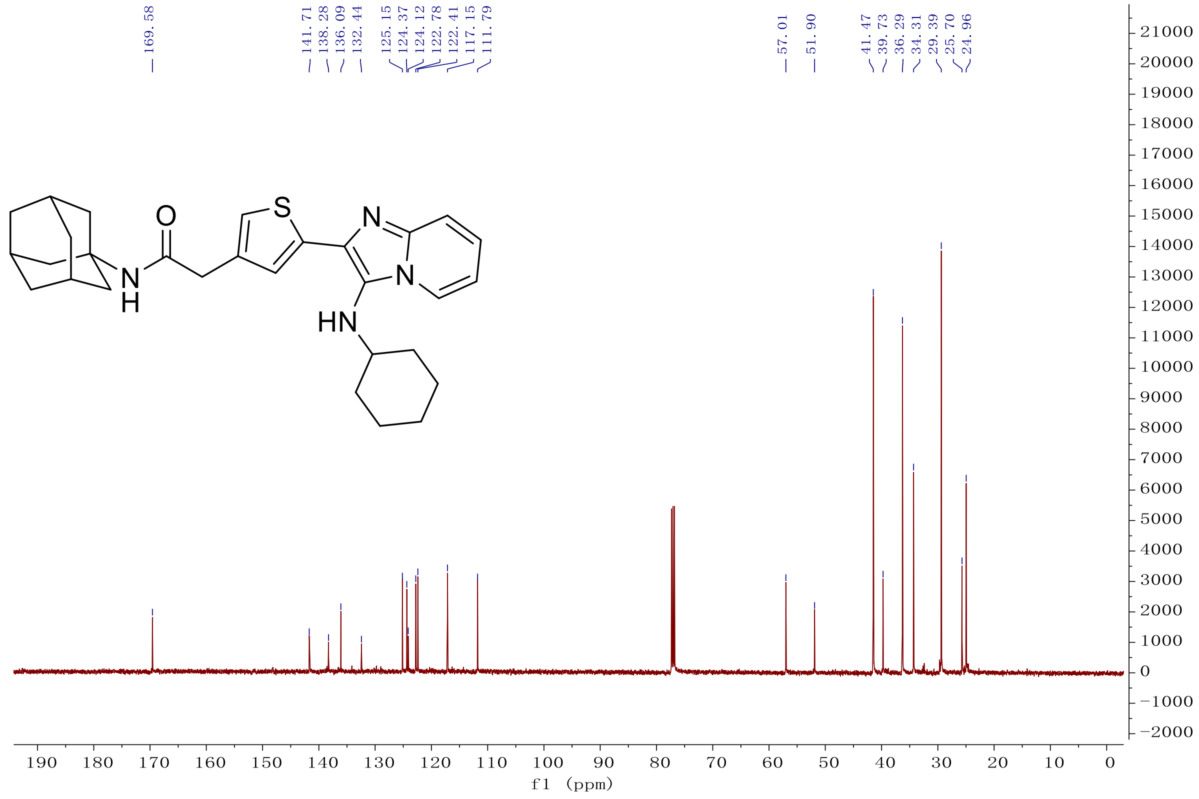

Supplementary Figure 143. ^{13}C NMR (126 MHz, $CDCl_3$) of compound **1c**

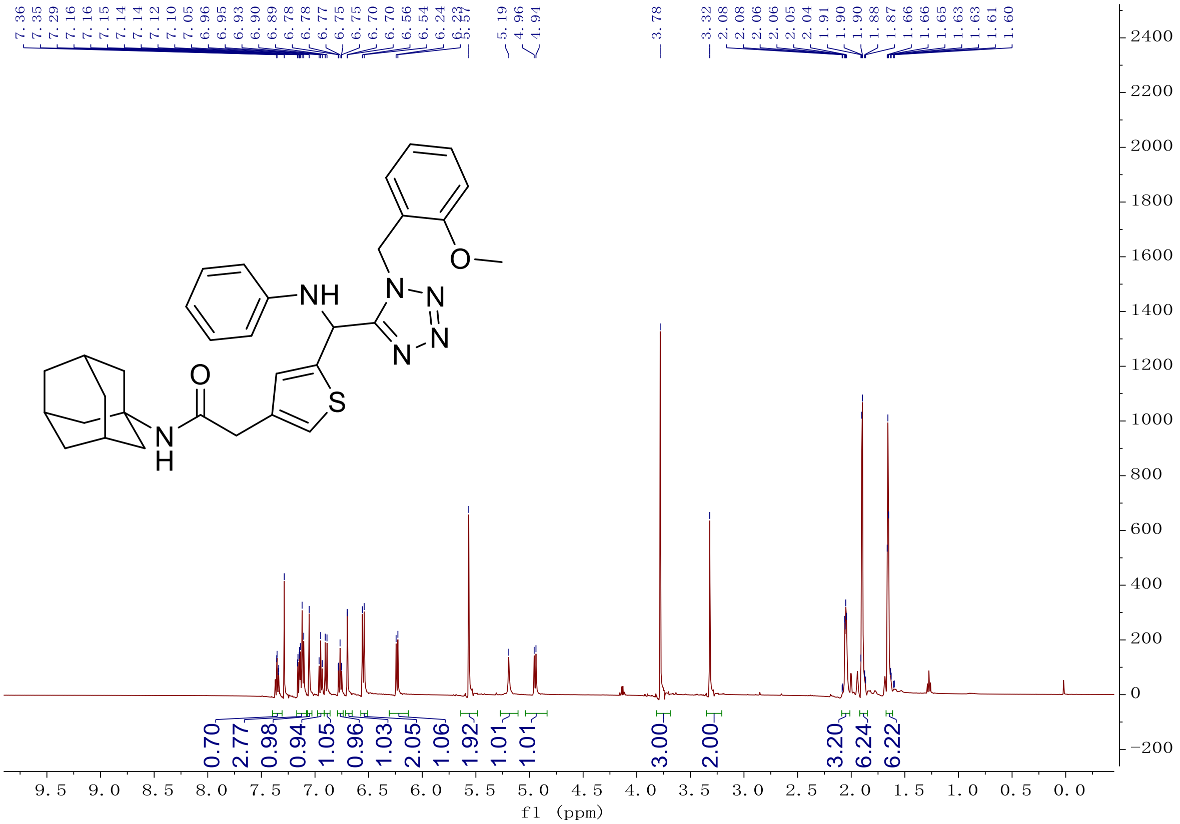

Supplementary Figure 144. ^{1}H NMR (400 MHz, $CDCl_3$) of compound **1d**

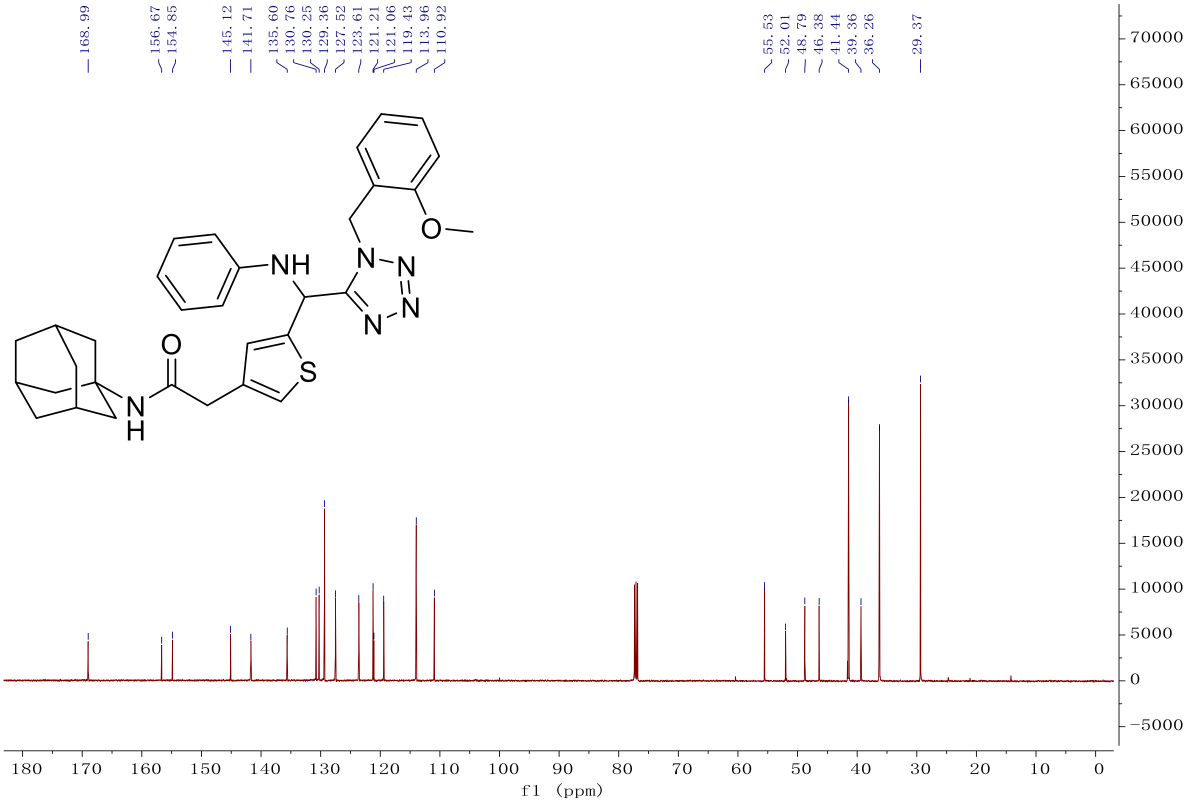

Supplementary Figure 145. ^{13}C NMR (126 MHz, $CDCl_3$) of compound **1d**

16. List of molecules which were ineffectively synthesized by this method:

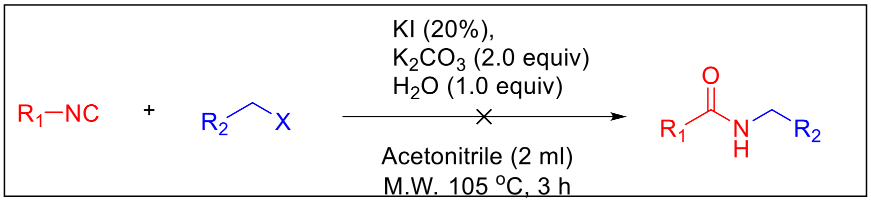

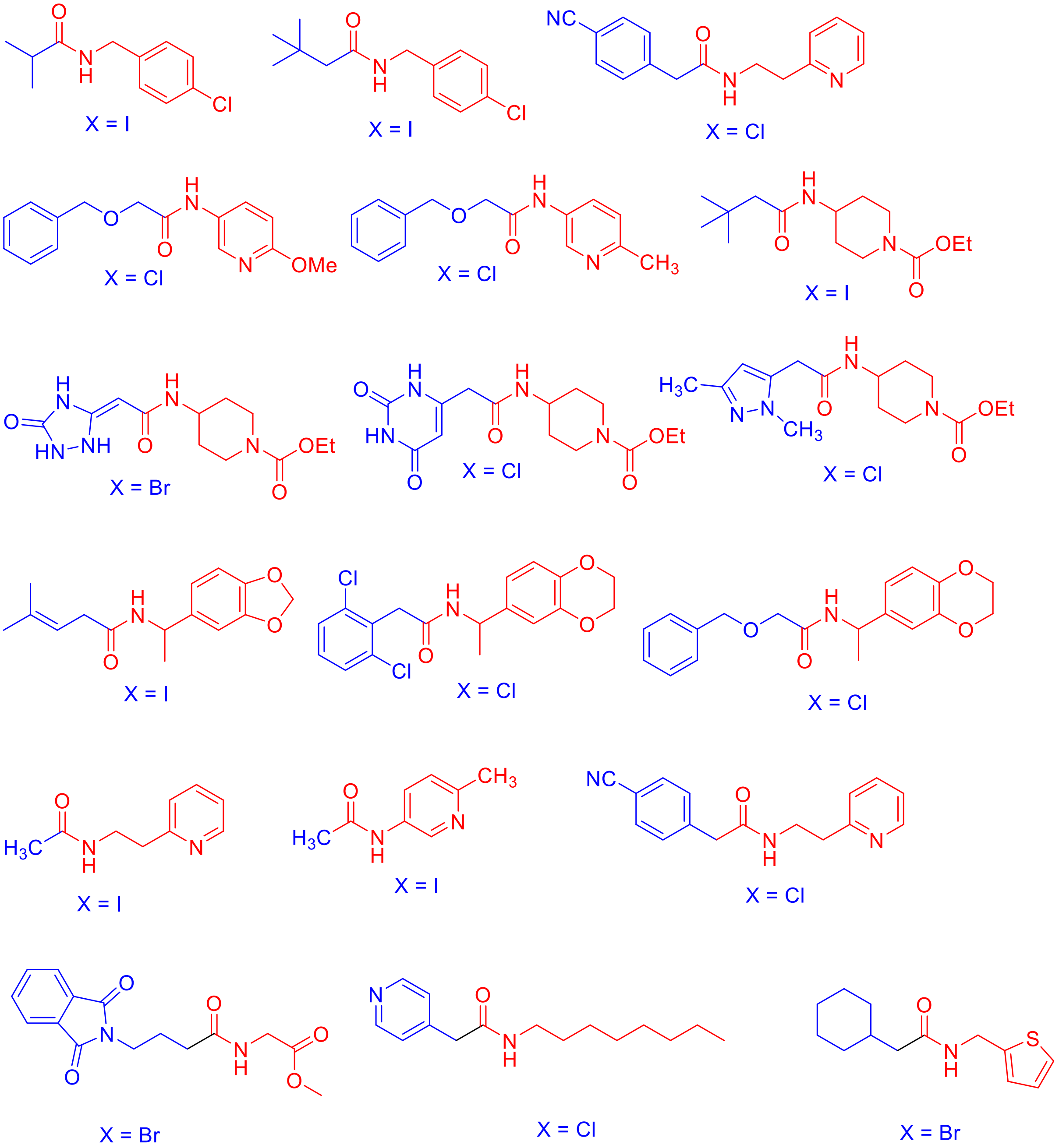

Supplementary Figure 146: List of molecules which were ineffectively synthesized by this method.

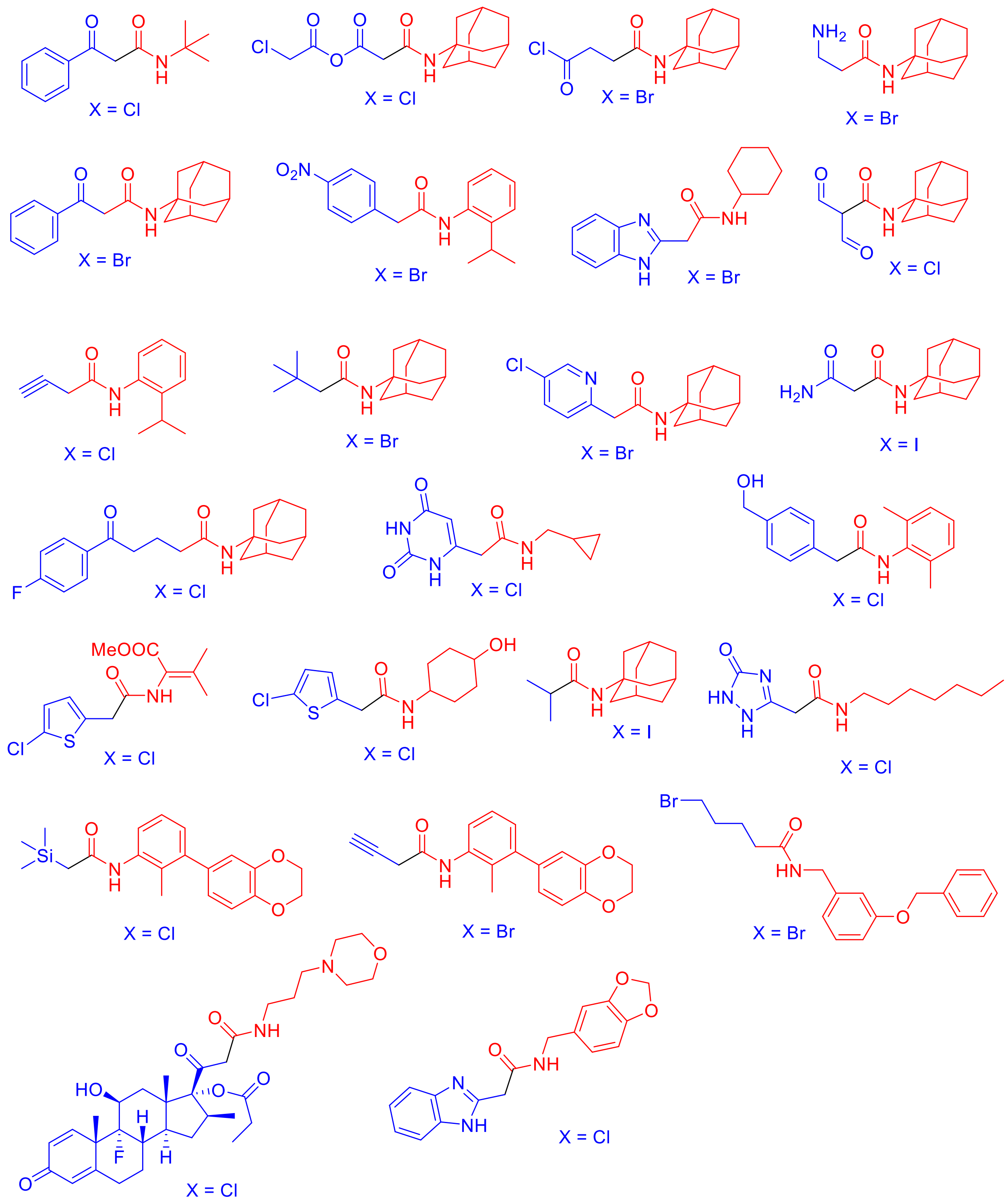

Supplementary Figure 147: List of molecules which were ineffectively synthesized by this method.

17. Crystal structure determination

X-ray diffraction data for single crystal of compounds **4a**, **16a**, **8a**, **12a** were collected using Rigaku XtaLAB Synergy S Dualflex diffractometer (four circle diffractometer with a mirror monochromator) with HyPix detector and a PhotonJet CuKα radiation source (λ = 1.54184 Å) for all collected data sets. Additionally, the diffractometer was equipped with a CryoJet HT cryostat system (Oxford Instruments) allowing low temperature experiments, performed at 100 (12) K. The obtained data sets were processed with CrysAlisPro software[4]. The phase problem was solved by direct methods using SUPERFLIP[5]. The parameters of obtained models were refined by full-matrix least-squares on F2 using SHELXL-2018/3[6]. Calculations were performed using WinGX integrated system (ver. 2021.2)[7]. The figure was prepared with Mercury 2020.3.0 software[8].

All non-hydrogen atoms were refined anisotropically. All hydrogen atoms attached to carbon atoms were positioned with the idealised geometry and refined using the riding model with the isotropic displacement parameter Uiso[H] = 1.2 (or 1.5 (methyl groups only)) Ueq[C]. Crystal data and structure refinement results for presented crystal structures are shown in supplementary table 3. The molecular geometry (asymmetric unit) observed in the crystal structures is shown in supplementary figure 148.

Crystallographic data have been deposited with the Cambridge Crystallographic Data Centre as supplementary publication no. CCDC 2213257 (**4a**), CCDC 2213258 (**16a**), CCDC 2213259 (**8a**), CCDC 2213260 (**12a**). Copies of the data can be obtained, free of charge, on application to CCDC, 12 Union Road, Cambridge CB2 1EZ, UK, (fax: +44-(0)1223-336033 or e-mail: deposit@ccdc.cam.ac.uk).

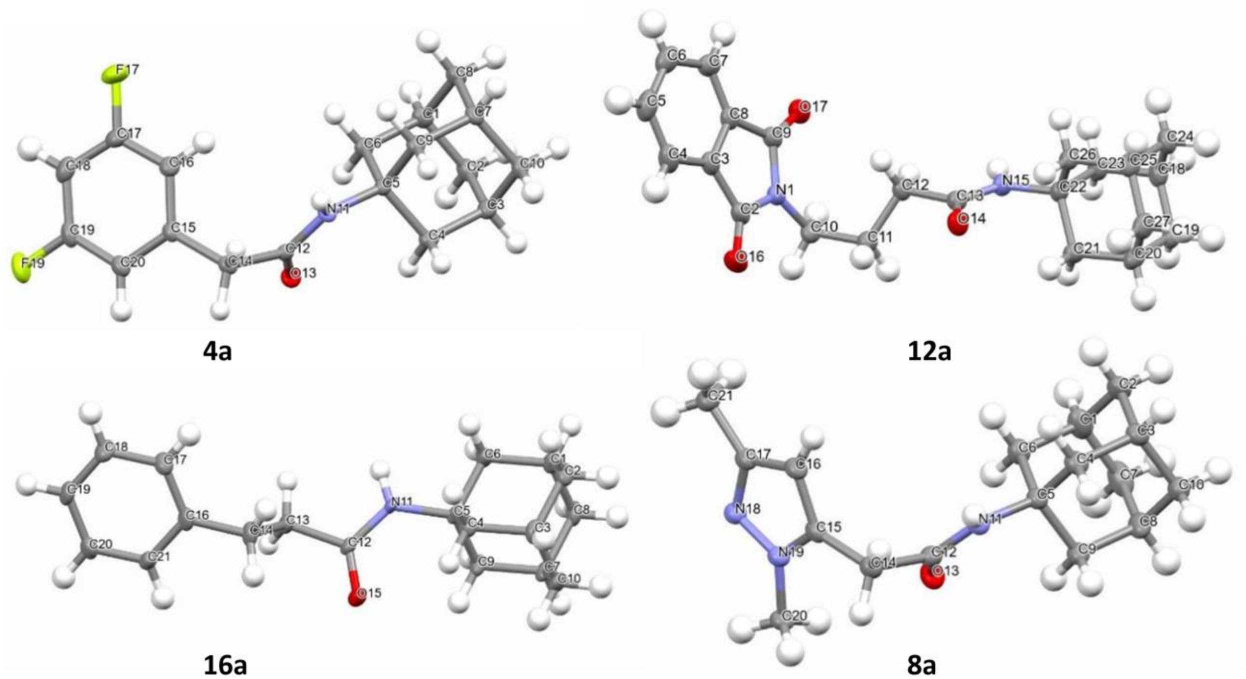

Supplementary Figure 148. Molecular geometry observed in the crystal structures of compounds **4a**, **16a, 8a, 12a** showing the atom labelling scheme. The positional disorder within the benzene ring is observed with equal site occupancy (50:50). Displacement ellipsoids of non-hydrogen atoms are drawn at the 30% probability level. H atoms are presented as small spheres with an arbitrary radius.

Supplementary Table 3: Crystal data and structure refinement results for compounds **4a**, **16a, 8a, 12a.**

	**5a**	**16a**	**8a**	**12a**
Empirical moiety formula	$C_{18}H_{21}F_{2}NO$	$C_{19}H_{25}NO$	$C_{17}H_{25}N_{3}O$	$C_{22}H_{26}N_{2}O_{3}$
Formula weight [g/mol]	305.36	283.40	287.4	366.45
Crystal system	Orthorhombic	Monoclinic	Monoclinic	Monoclinic
Space group	*P na2₁* → $P\,na2_1$	$I\,a$	$P\,2_1/n$	$I\,2/a$
Unite cell dimensions	a = 9.4933(3) Å<br>b = 12.0312(2) Å<br>c = 13.4052(5) Å<br>α=90°<br>β=90°<br>γ=90°	a = 9.3614(2) Å<br>b = 14.9273(3) Å<br>c = 11.7579(3) Å<br>α=90°<br>β= 102.562(2)°<br>γ=90°	a = 6.7262(2)Å<br>b = 13.7246(3)Å<br>c = 16.9017(4)Å<br>α=90°<br>β= 100.265(2)°<br>γ=90°	a = 9.6034(4) Å<br>b = 10.2099(4) Å<br>c = 39.5555(14) Å<br>α=90°<br>β= 95.026(3)°<br>γ=90°

Volume [Å^3]	1531.09(8)	1603.72(6)	1535.30(7)	3863.5(3)
Z	4	4	4	8
D_{calc} [Mg/m^3]	1.325	1.174	1.243	1.260
μ [mm^{-1}]	0.811	0.549	0.617	0.674
F(000)	648	616	624	1568
Crystal size [mm^3]	0.40 x 0.10 x 0.10	0.40 x 0.05 x 0.05	0.30 x 0.20 x 0.20	0.20 x 0.15 x 0.10
Θ range	4.93° to 75.18°	4.86° to 75.26°	4.17° to 75.04°	4.47° to 74.45°
Index ranges	-9 ≤ h ≤ 11, -11 ≤ k ≤ 15, -16 ≤ l ≤ 16	-11 ≤ h ≤ 11, -18 ≤ k ≤ 18, -12 ≤ l ≤ 14	-8 ≤ h ≤ 8, -17 ≤ k ≤ 15, -20 ≤ l ≤ 20	-12 ≤ h ≤ 11, -12 ≤ k ≤ 12, -49 ≤ l ≤ 47
Refl. collected	8071	8494	14395	18150
Independent reflections	2741 [R(int) = 0.0437]	2261 [R(int) = 0.0627]	3090 [R(int) = 0.0561]	3792 [R(int) = 0.1194]
Completeness [%] to Θ	99.9 (Θ 67.68°)	99.5 (Θ 67.68°)	99.5 (Θ 67.68°)	99.7 (Θ 67.68°)
Absorption correction	Multi-scan	Multi-scan	Multi-scan	Multi-scan
Tmin. and Tmax.	0.510 and 1.000	0.386 and 1.000	0.707 and 1.000	0.249 and 1.000
Data/ restraints/parameters	2741 / 200 / 1	2261 / 190 / 2	3090 / 192 / 0	3792 / 244 / 0
GooF on F2	1.070	1.139	1.040	1.036
Final R indices [I>2sigma(I)]	R1= 0.0353, wR2= 0.0877	R1= 0.0549, wR2= 0.1481	R1= 0.0544, wR2= 0.1339	R1= 0.0955, wR2= 0.2061
R indices (all data)	R1= 0.0342, wR2= 0.0866	R1= 0.0519, wR2= 0.1461	R1= 0.0485, wR2= 0.1294	R1= 0.0767, wR2= 0.1932
$\Delta\rho_{max}$, $\Delta\rho_{min}$ [e·Å^{-3}]	0.21 and -0.24	0.22 and -0.31	0.29 and -0.20	0.31 and -0.41

18. Supplementary References:

1. Reactor was used for enumeration and reaction modeling, JChem 22.16.0, 2022, ChemAxon (http://www.chemaxon.com).
2. Instant JChem was used for structure database management, search and prediction, Instant JChem 22.16.0, 2022, ChemAxon (http://www.chemaxon.com).
3. O'Boyle, N. M., Banck, M., James, C. A., Morley, C., Vandermeersch, T., Hutchison, G. R., Open Babel: An open chemical toolbox. *J. Cheminform*., **3**, 33 (2011).
4. Rigaku-Oxford Diffraction; CrysAlisPro Oxford Diffraction Ltd, Abingdon, England V 1. 171. 36. 2. (release 27-06-2012 CN (2006).
5. Palatinus, L. Chapuis, G., SUPERFLIP - A computer program for the solution of crystal structures by charge flipping in arbitrary dimensions. *J. Appl. Cryst.* **40**, 786-790 (2007).
6. Sheldrick, G. M., A short history of SHELX. *Acta Cryst*. A**64**, 112-122 (2008).
7. Farrugia, L.J., WinGX and ORTEP for Windows: an update. *J. Appl. Cryst*. **45**, 848-854 (2012).
8. Macrae C. F., Edgington P.R., McCabe P., Pidcock E., Shields G.P., Taylor R., Towler M., & van de Streek J., *Mercury*: visualization and analysis of crystal structures. *J. Appl. Cryst.* **39**, 453-457 (2006).
